# A DNA target-enrichment approach to detect mutations, copy number changes and immunoglobulin translocations in multiple myeloma

**DOI:** 10.1038/bcj.2016.72

**Published:** 2016-09-02

**Authors:** N Bolli, Y Li, V Sathiaseelan, K Raine, D Jones, P Ganly, F Cocito, G Bignell, M A Chapman, A S Sperling, K C Anderson, H Avet-Loiseau, S Minvielle, P J Campbell, N C Munshi

**Affiliations:** 1 Cancer Genome Project, Wellcome Trust Sanger Institute, Cambridge, UK; 2 Department of Oncology and Onco-Hematology, Fondazione IRCCS Istituto Nazionale dei Tumori, University of Milan, Milan, Italy; 3 Department of Pathology (UOC), University of Otago, Christchurch, New Zealand; 4 Division of Hematology, Fondazione IRCCS Policlinico San Matteo di Pavia, Pavia, Italy; 5 Department of Haematology, University of Cambridge, Cambridge, UK; 6 Jerome Lipper Multiple Myeloma Center, Department of Medical Oncology, Dana-Farber Cancer Institute, Harvard Medical School, Boston, MA, USA; 7 Unité de Genomique du Myelome, Centre Hospitalier Universitaire Rangueil, Toulouse, France; 8 Centre de Recherche en Cancerologie de Toulouse, INSERM U1037, Toulouse, France; 9 Hematology Department, Centre de Recherche en Cancerologie Nantes-Angers Unité Mixte de Recherche INSERM 892–Centre National de la Recherche Scientifique 6299 and Institut de Recherche Therapeutique de l'Université de Nantes, Nantes, France

## Abstract

Genomic lesions are not investigated during routine diagnostic workup for multiple myeloma (MM). Cytogenetic studies are performed to assess prognosis but with limited impact on therapeutic decisions. Recently, several recurrently mutated genes have been described, but their clinical value remains to be defined. Therefore, clinical-grade strategies to investigate the genomic landscape of myeloma samples are needed to integrate new and old prognostic markers. We developed a target-enrichment strategy followed by next-generation sequencing (NGS) to streamline simultaneous analysis of gene mutations, copy number changes and immunoglobulin heavy chain (IGH) translocations in MM in a high-throughput manner, and validated it in a panel of cell lines. We identified 548 likely oncogenic mutations in 182 genes. By integrating published data sets of NGS in MM, we retrieved a list of genes with significant relevance to myeloma and found that the mutational spectrum of primary samples and MM cell lines is partially overlapping. Gains and losses of chromosomes, chromosomal segments and gene loci were identified with accuracy comparable to conventional arrays, allowing identification of lesions with known prognostic significance. Furthermore, we identified IGH translocations with high positive and negative predictive value. Our approach could allow the identification of novel biomarkers with clinical relevance in myeloma.

## Introduction

Multiple myeloma (MM) is a hematological neoplasm that arises from transformation and clonal proliferation of plasma cells.^1^ Virtually every case of MM is characterized by gross chromosomal rearrangements in the form of either hyperdiploidy or translocations predominantly involving the immunoglobulin locus^2^ that can be tracked along the typical multi-step disease progression from the preclinical stages of monoclonal gammopathy of unknown significance to the final setting of relapsed-refractory MM.^3^ Identification of cytogenetic abnormalities using conventional karyotyping and fluorescence *in situ* hybridization is a standard part of the initial workup and risk stratification^4^ and may guide clinical practice in some circumstances. Patients with del17p, t(4;14) and t(14;16) are considered to have high risk disease^5, 6^ and the ability of bortezomib-based treatments to overcome the adverse prognosis associated with t(4;14)^7^ helps in making treatment decisions. Similarly, clinical and genetic features associated with good response to lenalidomide have recently been described.^8^ The ever-increasing availability of new drugs targeting recurrent genetic lesions^9^ and better understanding of the biological features of myeloma has prompted a need for updated risk stratification and a rational approach to the use of new agents alone or in combination. In fact, attempts at delivering risk-adapted therapy have already been performed in the context of clinical trials.^10, 11^

Molecular studies are not routinely performed in myeloma outside of investigational trials. However, recent next-generation sequencing (NGS) studies have added considerable resolution to the landscape of genomic abnormalities of myeloma, highlighting how it behaves as a heterogeneous admixture of subclones evolving dynamically over time based on differential chemosensitivity and intrinsic genomic instability.^12, 13, 14, 15^ Nevertheless, myeloma is a disease driven by an intricate and heterogeneous interplay of genetic events and these data have failed so far to provide a unifying view of its pathogenesis and clinical behavior. If advances in genomics are to be used in the future to define prognosis and to inform therapy, integration of even larger studies and clinical data sets will be required. Initial efforts to incorporate these new findings into standard risk models are currently underway.^16^ Targeted NGS has significant advantages over whole-genome or whole-exome sequencing as it allows high-throughput, robust and easy analysis of chromosomal and gene lesions of large cohorts of patients by reducing the footprint of the genome to be sequenced in each case. Such studies have already been performed in acute myeloid leukemia,^17, 18^ myelodysplastic syndrome^19, 20^ and myeloma to detect recurrent gene lesions^21, 22^ or characterize immunoglobulin heavy chain (IGH) translocations,^23^ but their full potential to comprehensively annotate the extended spectrum of genomic lesions with prognostic significance in myeloma has not been exploited so far.

In this study, we developed and validated a novel target-enrichment strategy based on DNA pull-down followed by NGS to streamline simultaneous high-throughput analysis of gene mutations, copy number alterations, immunoglobulin translocations and tumor-specific V(D)J rearrangements in MM that could be applied to patient samples even by laboratories with limited NGS and analytic expertise.

## Materials and methods

### Samples, DNA target enrichment, sequencing and alignment

Native DNA at 500 ng was extracted from 24 hematopoietic cells lines: 14 MM lines and 10 control myeloid and lymphoid lines (Supplementary Table S1). For 5 primary patient samples banked for ⩾4 years, 10 ng of DNA was whole-genome amplified using the REPLI-g mini kit (Qiagen, Manchester, UK) and 500 ng of whole-genome amplified DNA was used for library construction and sequencing. Samples and data were obtained and managed in accordance with the Declaration of Helsinki under protocol 08/H0308/303: somatic molecular genetics of human cancers, Melanoma and Myeloma (Dana Farber Cancer Institute, Boston, MA, USA). The same protocol was approved by RES Committee East of England–Cambridge Central.

We designed a target-enrichment design based on DNA pull-down by cRNA baits (SureSelect, Agilent Technologies, Santa Clara, CA, USA). We selected 246 genes implicated in myeloma and/or cancer in general based on previous literature to establish the prevalence of the recurrent mutations and identify novel cancer gene mutations not described before in myeloma. We also selected 2538 single-nucleotide polymorphisms (SNPs), evenly spaced across the genome and highly polymorphic based on the 1000 Genomes project^24^ to detect copy number and allelic frequency changes at the single-gene and whole-genome level. In known regions of copy number abnormality we tiled SNPs more densely to improve resolution. Finally, we targeted the whole IGH locus to detect translocations and V(D)J rearrangements. The final design was created using the online SureDesign tool (Agilent Technologies), using no repeat masking for exonic regions, moderate masking for intronic regions and minimum masking for the IGH locus. The total size of our design reached a total footprint of 2.992 Mbp (Supplementary Table S2). DNA and library preparation was carried out as described previously.^14^ A total of 16 and 8 samples were pooled and target DNA was subsequently enriched using one reaction tube each from the SureSelect kit. All 24 samples were sequenced in one lane of HiSeq2000 with a 75-bp paired-end protocol. FastQ files were aligned to the human genome (NCBI build 37) using BWAmem that improves detection of insertions, deletions and rearrangements by clipping and mapping of chimeric reads spanning breakpoints that would otherwise get discarded by BWAaln.^25^ Unmapped, duplicate or off-target reads were excluded from analysis. Aligned BAM files are available at the URL https://www.ebi.ac.uk/ega/studies/EGAS00001000743.

### On-target copy number and B-allele frequency analysis

Probes could be designed for >99% of the input design after excluding highly repetitive regions. We used Bedtools (v2.1523)^26^ to retrieve depth of coverage of the target region. We then normalized coverage in each sample by dividing the read count at each position by the total number of on-target uniquely mapped bases for that sample. Copy number data were then generated as a LogR ratio between the normalized depth of each sample and the mean depth of three cell lines with normal karyotype—as shown by the Genome-Wide Human SNP Array 6.0 (Affimetryx, Inc., Santa Clara, CA, USA)—used as ‘normal controls'. We then derived the average LogR copy number of the subchromosomal regions and genes that are affected by recurrent abnormalities to detect gains and losses. To generate B-allele frequency plots, calls from our list of 2538 SNPs were analyzed to remove SNPs that gave inconsistent results, and for the remaining ones the percentage of reads supporting the minor allele was plotted. These analyses were performed with bespoke R scripts (R v3.0.3)^27^ available on request. Results were validated with copy number data generated by Genome-Wide Human SNP Array 6.0 (Affymetrix, Inc.).

### Mutation calling algorithms

Substitutions and insertions/deletions were detected using CaVEMan and Pindel as previously described.^14^ To evaluate the accuracy of mutation calling, we used results from the ‘catalogue of somatic mutation in cancer (COSMIC) cell line whole exome sequencing (WES) project',^28^ limiting analysis to calls within the target region of our study. Variants were cross-referenced with the following databases: exome sequencing project v0.0.18, 1000 Genomes, NCBI dbSNP build 137 and COSMIC v68.

### IGH translocations and rearrangements

For IGH translocations, we manually ran Brass^29^ to find clusters of read pairs where one read maps to the IGH locus and the other in proximity to a known partner of translocation (Supplementary Table S3).

To detect V(D)J rearrangements, we first segmented the sequencing depth of the IGH region using the R package ‘changepoint' in order to find positions of copy number change indicative of loss of copy number secondary to deletions in the locus. We then retrieved clusters of read pairs where at least one end mapped to a segmentation breakpoint (max distance: 2 kb) such that the read pair orientations and sequencing depth changes were consistent with a deletion event. To filter out mismapping artifacts and only focus on high-confidence events, we also analyzed a panel of 48 normal samples targeted with the same design (as well as the nonlymphoid lines within our cohort). We only retained events that (1) were supported by ⩾10 reads in the test sample and none in the controls; (2) were supported by reads mapped with a quality of ⩾27 on one end and >0 on the other; (3) were around a deletion of at least 8 kb of length; and (4) whose start and end coordinates were not duplicated in the control cohort, suggestive of potential artifacts.

## Results

### Sequencing metrics of the study

**Figure 1:**
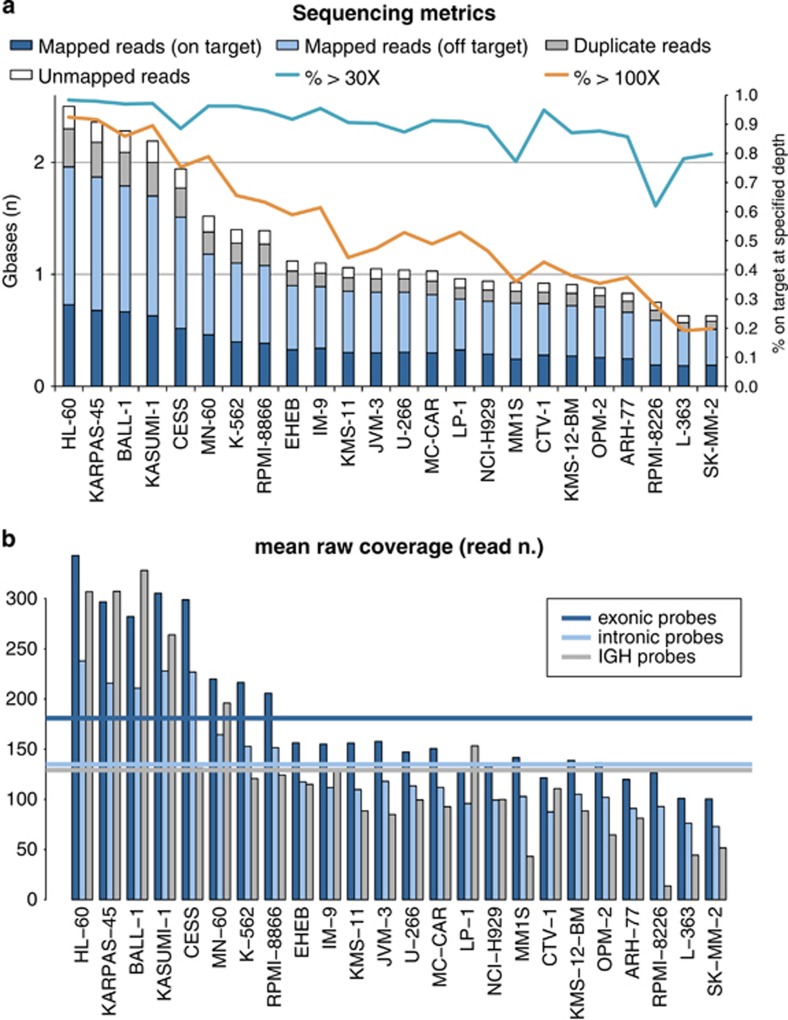
Sequencing metrics of the study. (**a**) Bars represent the absolute number of gigabases sequenced (left y axis). Lines represent the percentage of the target region sequenced above the threshold of 30 × (light blue) or 100 × (orange), referenced to the right y axis. (**b**) Bars represent the mean coverage of exonic regions (dark blue), intronic regions (light blue) and the IGH region (gray) in each sample. Lines represent median coverage of each region across the whole cohort of samples.

We sequenced a total of 30.36 Gb for this study, with an average of 1.26 Gb per sample (range, 0.63–2.5). The mean coverage of the target region was 155.48 ×, resulting from a rather low average on-target efficiency of 36.3% (Figure 1a). We believe that this low on-target efficiency depended in part on the intronic probes designed to capture SNPs, whose performance was significantly lower than that of exonic probes for most samples (Figure 1b). We also investigated whether capture of the IGH region, which harbors repetitive regions and was rearranged in B-lymphoid cell lines, contributed to this low efficiency. We found that the mean coverage of the IGH region was 130 × across the whole cohort, not different from that of the intronic regions (133 ×). Nevertheless, this was closer to exonic probes in samples without IGH rearrangements, suggesting that the IGH locus can be targeted with good efficiency. In samples with IGH rearrangements instead, coverage of the locus dropped as expected because of the multiple deletions in the region, thus indicating that our on-target performance was in part artifactually low owing to the actual IGH region being smaller than annotated in the reference genome (Figure 1b). Overall, 90% and 63% of the target region was covered on average at more than 10 × or 30 ×, respectively. In particular, exons in the study were covered at an average depth of 181 ×, with 99 and 71% of bases covered at more than 30 × and 100 ×. We therefore conclude that our design, despite its large size and the presence of challenging regions, performed well in target enrichment returning enough coverage—for the amount of sequencing performed—for reliable interpretation and quantitation of the data.

### Validation of variants in the study

**Figure 2:**
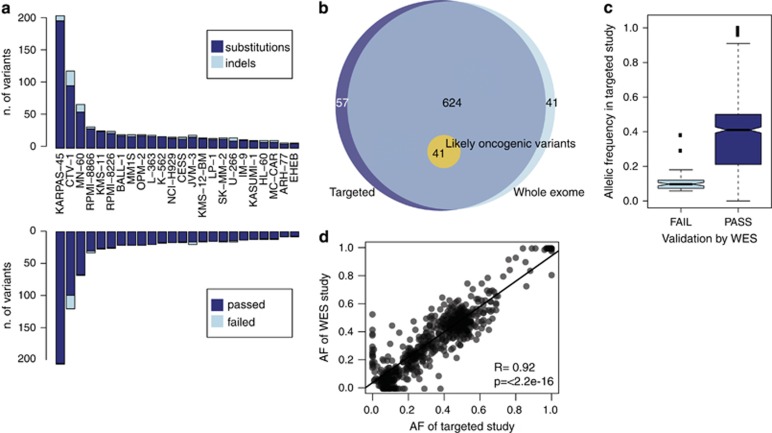
Validation of variants in the study. (**a**) Histogram showing the absolute number of variants broken down by type (top) or by validation from a previous whole-exome sequencing study (bottom). (**b**) Venn diagram showing the overlap of variants as called by a whole-exome sequencing study (light blue), our targeted sequencing study (dark blue) and the accuracy in reporting ‘likely oncogenic variants'. (**c**) Boxplot representing the allelic ratio of variants in our targeted study that were confirmed by whole exome (dark blue) or not confirmed (light blue). (**d**) Scatter plot confirming a strong correlation of the allelic frequency of variants reported by whole exome or by our target-enrichment study.

We used CaVEMan and Pindel to identify 831 coding nonsynonymous variants in our 24 samples, composed of 712 substitutions and 119 indels (Figure 2a). 722/831 positions were adequately covered (>20 ×) in both our study and the WES used as control, and were therefore used for analysis. 665/722 variants in our study were confirmed in the WES data^28^ (Figure 2b), resulting in an apparent specificity of 92% for our custom pull-down approach. Notably, the average allelic frequency was 37.91% for the validated variants but only 10.63% for the 57 that were not confirmed (Figure 2c), suggesting they probably represent a mixture of artifacts (false positives) and subclonal variants (true positives) that the WES study did not identify because of lower coverage. Conversely, despite a higher coverage our targeted study did not identify 41 variants reported by WES (Figure 2b). This underscores the difficulty of establishing the sensitivity of a given sequencing platform, as the full catalog of true mutations for any given sample is for the most part unknown. We therefore sought a measure of sensitivity of our study by creating a shortlist of 41 ‘likely oncogenic' variants from the WES study. These variants were selected because they were present in genes classed as likely to drive oncogenesis in a large study of primary tumors.^30^ Of these variants, 21 were in genes identified as drivers in MM, and the remainder were in genes identified as drivers in other tissues. Selected variants also had to be recurrent in the COSMIC database, with substitutions residing in codons that were mutated in >2 primary tumor samples and protein terminating variants (nonsense or frameshift indels) occurring in genes containing >10 such variants in primary tumor samples. Our targeted study identified all 41 variants, thus showing 100% sensitivity and a similarly absolute positive predictive value with respect to this highly relevant subset of variants (Figure 2b). Furthermore, the significant concordance of allelic fraction of shared variants (Figure 2d) suggested that our targeted study retained quantitative value similar to WES data.

Cell line DNA may not be representative of real-life settings, where patient DNA may be of poor quality, limited amount and contaminated with normal cells. Therefore, to test the robustness of our target-enrichment strategy, we tested its performance on whole-genome amplified DNA from five patients previously analyzed by WES.^14^ Our targeted design showed a sensitivity of 100% and a specificity of 91.67%, as it identified two lesions not reported by the WES study because of lower or absent coverage of the region (Supplementary Figure S1). Furthermore, the correlation between allelic frequencies of the two sequencing experiments was almost perfect, confirming that our design and analysis strategy retain quantitative value even in samples of lower quality subject to DNA amplification.

### Identification of likely somatic mutations

**Figure 3:**
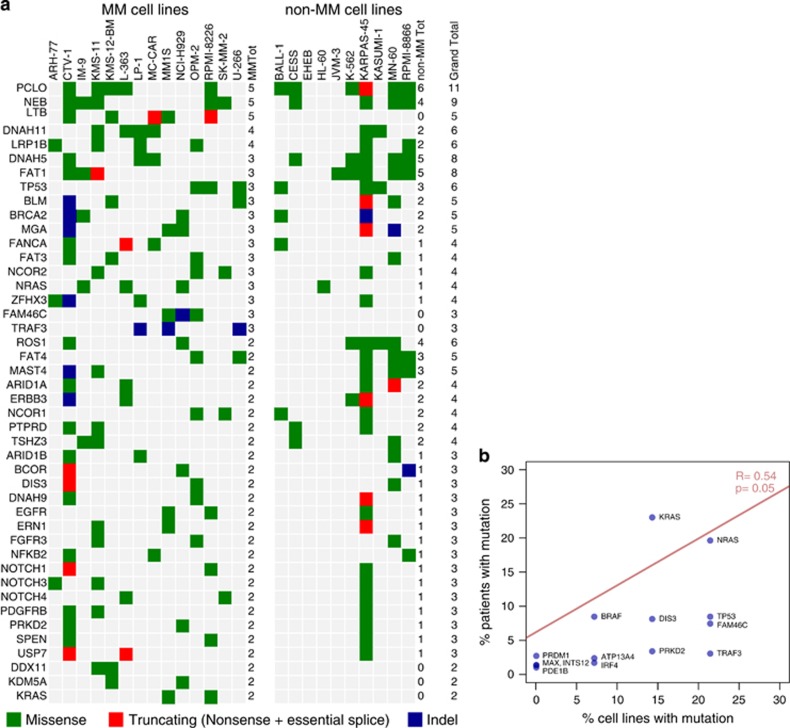
Identification of variants in the cohort. (**a**) Variants in genes hit at least twice in the study are reported based on the type of mutation (missense in green, truncating in red and indel in blue) and based on the tissue of origin of the cell line where the variant was identified (left for MM cell lines and right for non-MM cell lines). (**b**) Scatter plot showing that the frequency of myeloma driver gene mutations in cell lines has a loose but significant correlation with data from primary patient samples.

After establishing a measure of accuracy for the identification of real variants, we focused on the identification of likely somatic variants, a particularly hard task in cell lines as they lack a matched normal control and carry numerous private polymorphisms. We used known human variation databases and a panel of 317 normal samples internal to the Sanger Institute to exclude variants that were reported as known constitutional polymorphisms with a frequency of >1% in the general adult population (roughly estimating the prevalence of monoclonal gammopathies in the adult population). We thus filtered our data and obtained a list of 182 mutated genes and 548 ‘likely somatic' variants (Supplementary Table S4) in our cell lines. Our list (Figure 3a) returned a number of known mutated oncogenes and tumor suppressors like *TP53*, *KRAS* and *NRAS* across cell types. Furthermore, known myeloma genes such as *FAM46C*, *LTB* and *TRAF3* were specifically mutated in MM cell lines only, suggesting that cell lines retain a spectrum of mutation that resembles that of the tissue of origin, and that tissue restriction could be used as a way to filter genes with likely functional role in MM from cell line data.

Nevertheless, looking at MM cell lines, the spectrum of variants differed quite substantially from that published in primary human MM samples. The most commonly mutated gene was *PCLO*, encoding a presynaptic cytoskeletal protein involved in neurotransmitter release.^31^ *PLCO* was previously reported as frequently mutated in diffuse large cell lymphoma but with an extremely high synonymous to nonsynonymous ratio, suggestive of an unusually high local rate of passenger mutations without functional consequences in B-cell malignancies.^32^ A similar phenomenon likely explains the presence of *NEB*, a giant cytoskeletal protein^33^ with no known role in B-cell biology, as the second mutated gene in the rank. This hypothesis is further reinforced by the observation that these genes are the most frequently mutated across all cell lines irrespective of their tissue of origin, likely as a result of ongoing local mutational processes. This highlights the need for a list of significant candidate driver genes in myeloma, corrected for more sophisticated variables than their crude recurrence rate. As the small number of cell lines in our study would not allow for a robust statistical analysis, we used the mutation data sets of three large MM sequencing papers^13, 14, 15^ (*n*= 292 samples) and applied the MutSigCV2 algorithm^34^ to establish the spectrum of driver genes mutated in myeloma at a significant rate. We found 14 significantly mutated genes in MM (Table 1). Out of 14 genes, 10 were also mutated in our MM cell lines, showing correlation of their recurrence rate with that found in primary samples (Figure 3b). Although the number of cell lines is small when compared with the number of patients, these findings suggest that MM cell lines can partially recapitulate the genomic features of primary MM samples.

### Identification of copy number changes

**Figure 4:**
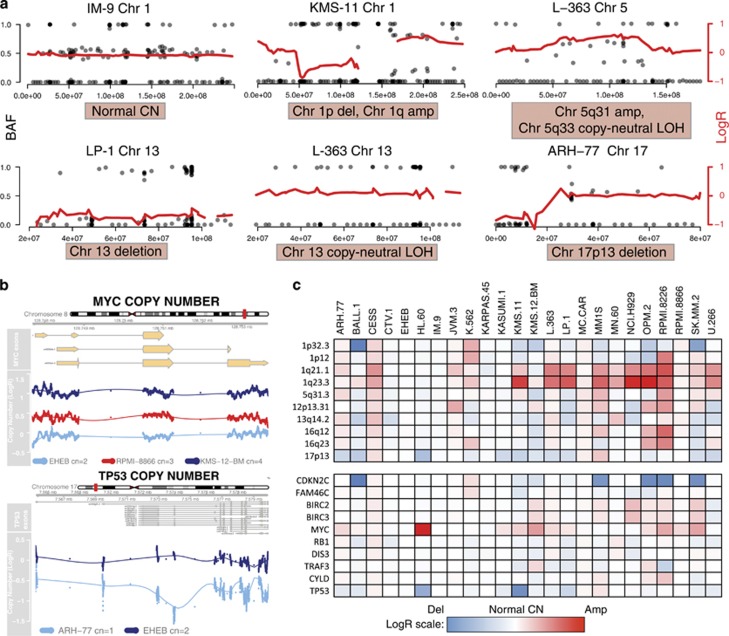
Copy number (CN) and loss of heterozygosity (LOH) analysis. (**a**) Representative examples of CN and LOH analysis in cell line samples. Each plot represents a chromosome in a cell line. The red line represents an interpolation of the log ratio (LogR) of coverage depth between the cell line in study and that of three cell lines with normal karyotype, referenced to the right y axis where 0 means the ploidy of the region is the same as the average ploidy of the control cell lines. Black dots represent the B-allelic frequency of the SNPs we targeted for that chromosome. Heterozygous regions show SNPs at either 0, 0.5 or 1 (referenced to the left y axis). Losses show SNPs at either 0 or 1. In region of gains, B allele frequency (BAF) shows a tetramodal distribution, typically 0, 0.33, 0.66 and 1 for trisomies. Genomic coordinates of the chromosome are in the x axis. (**b**) The same interpolated LogR analysis was applied for single gene loci, showing gains for MYC and losses for TP53. Dots represent the LogR coverage of individual bases in the target region. (**c**) Heatmap summarizing the LogR copy number status of regions and genes with recurrent copy number abnormalities in the cell lines studied. Maximum (red) and minimum (blue) are calculated on the highest and lowest value of the data set.

MM is a disease characterized by recurrent copy number abnormalities, many of which have prognostic value.^35^ We therefore used normalized coverage data from our exonic probes and from intronic SNPs to infer the copy number status (relative to each sample's total ploidy) and allelic frequencies of chromosome segments and genes. Figure 4a shows examples of how we could use this approach to detect deletions in chr1p, chr13 and chr17p13, as well as amplifications in chr1q and chr5q. Combined analysis of copy number and B-allele frequency of SNPs allowed identification of copy neutral loss of heterozygosity events such as the one affecting the whole chromosome 13 in L-363 (Figure 4a). Furthermore, we could extend this analysis to the single-gene level and we correctly identified for example, gains in MYC and losses in TP53 (Figure 4b). All of these copy number changes were validated using previously generated data from the genome-wide human SNP array 6.0 (Affymetrix, Inc.). We applied this analysis more systematically to known regions and genes affected by recurrent copy number changes in myeloma. We built a heatmap showing the average LogR for copy number of each locus, relative to the average ploidy of normal samples, demonstrating our ability to detect gains and losses carrying prognostic value in myeloma. For example, losses in 1p and gains in 1q were quite common and specific for myeloma cell lines, whereas TP53 deletions and MYC amplification were more widespread in the cohort irrespective of the origin of the cell line (Figure 4c). Overall, these results confirm that NGS data have quantitative value similar to clinical-grade platforms, a feature that can be exploited in future diagnostic applications.

### Identification of translocations involving the IGH locus

**Figure 5:**
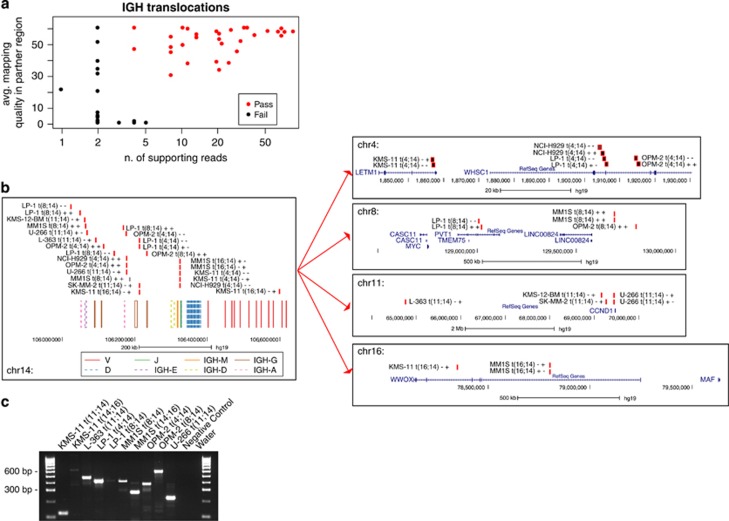
Detection of IGH translocations. (**a**) Scatter plot of IGH translocation events detected by our pipeline, plotted by number of supporting reads (x axis) and average quality of mapping in the partner chromosome region (y axis). In red, events that passed our filters. (**b**) Schematic representation of translocations involving the IGH locus. (**b**, left) Mapping of the breakpoints on chromosome 14 is represented along with annotation of the variable and constant regions of the immunoglobulin locus, showing that most events are located in the class switch region. Annotation of the variable regions of the immunoglobulin locus: V, D and J segments are in solid red (V), dashed blue (D) and solid green (J). The constant regions are in solid orange (M), dashed yellow (D), solid brown (G) and dashed pink (A). (**b**, right) Mapping of the breakpoint on the partner chromosomes, along with annotation of the genes involved. Breakpoint sites are represented as black boxes with red borders, and are annotated with the cell line name, the chromosomes involved and the chromosomal strands involved. (**c**) Genomic PCR validating the events represented in (**b**).

A substantial fraction of MM patients harbor translocations between the IGH locus and oncogenes that have pathogenic, diagnostic and prognostic value.^2^ In our design, we tiled the whole IGH locus to identify such translocations through pull-down of the genomic DNA surrounding the breakpoints and identification of the translocation partner by paired-end sequencing. As ours was not a discovery effort, we focused on known translocations to evaluate the performance of our approach. We analyzed 14 MM and 2 acute lymphoblastic leukemia cell lines. With a simple filter on mapping quality and depth of the supporting reads we shortlisted 13 translocations (Figure 5a, Table 2 and Supplementary Table S5). Interestingly, only in some cases did we find evidence of balanced, reciprocal translocations, supported by two groups of paired reads in opposite directions. Rather, in many cases only one read group supported the translocation, often with the two read pairs in concordant orientations (Supplementary Table S5). This suggests that the latter are part of rearrangements involving other partners and complex inversion–translocation events, and our approach was able to capture such translocations as well. Furthermore, in cases where both derivative chromosome breakpoints were captured, they often covered two different switch regions (see, for example, t(4;14) in NCI-H929, Figure 5b), suggesting that the translocation has developed during or after the class switch recombination process, with deletion of the intervening region on chromosome 14. More rarely, the breakpoint fell in the variable region, indicating that the translocation resulted from non-class switch recombination mechanisms (see, for example, t(14;16) in KMS-11, Figure 5b).^23^

In MM cell lines we collectively identified and mapped four cases each of t(11;14) and t(4;14), three cases of t(8;14) and two cases of t(14;16) (Figure 5b). Interestingly, 50% of the MM cell lines showed two translocations, one often involving the MYC locus. MYC translocations are described as late events in MM,^36^ associated with aggressive progression, and often co-exist with other IGH translocations, and therefore are not surprisingly enriched in myeloma cell lines.

All these events were manually inspected in IGV, and to further validate our effort we performed PCR in the 9/11 cell lines for which DNA was available. This validation effort confirmed the presence of the translocation in all but two cases in which the breakpoint on the IGH locus was located in a highly repetitive region not amenable to priming (Table 2). In our small cohort, we could identify IGH translocations with high accuracy and we therefore believe that our platform can reliably detect IGH translocations in primary MM samples.

## Discussion

The incorporation of the spectrum of genomic lesions in prognostic and therapeutic models of MM requires the development of new methods and technologies to gather all the inputs required for a unified predictive tool. The ever-growing landscape of treatment options in MM suggests that molecular analysis will be a part of routine clinical practice in the near future to aid prognostic and therapeutic stratification. Here we present a novel sequencing approach and analysis pipeline that could be implemented in diagnostic laboratories. It allows multiplexing of patient samples without the need for a matched normal sample for high-throughput analysis. It returns analysis of mutations, copy number changes and IGH translocations that are currently only performed in reference centers through integration of fluorescence *in situ* hybridization, SNP arrays and PCR data at a total cost per sample that in aggregate could be higher than with the method described here. Furthermore, as our design can be refined further to exclude regions of scarce clinical relevance and as sequencing technologies continue to improve, we anticipate costs and turnaround times will decrease further in the future, making NGS a viable option for routine clinical practice.

The use of cell lines to validate our design has the advantage of providing high-quality and abundant DNA for analysis. We anticipated that the clonal nature of the sample would limit the performance of our platform on subclonal variants that are quite frequent in MM.^14, 15^ Surprisingly though, looking at the allelic fraction of our variants, we found instead that most cell lines harbored subclonal variants. All cell lines were tested with short tandem repeat analysis to exclude mix-ups and contaminations, and most of our variants were confirmed by the WES study on the same cells performed within the COSMIC cell line project.^28^ These subclonal variants are therefore likely to be real and indicate subclonal evolution of the cell line, a finding that has been observed before in clonogenic studies.^37^ Our platform therefore performed well at the identification of variants at low allelic frequency, likely aided by the high depth of sequencing, showing a very low rate of false positives (<8%). We believe that our sensitivity was also high, but the evaluation of false negatives is always more difficult than that of false positives because no method can reliably report a complete list of mutations in a sample with absolute accuracy. Although the fraction of missed mutations is therefore hard to evaluate for any given mutation caller, our effort was limited to the identification of likely oncogenic variants in known cancer genes, and we showed absolute sensitivity for those. Furthermore, when applied to actual patient samples, we showed 100% sensitivity for detection of variants of any allelic fraction, again stressing the robustness of our platform across a range of sample quality and purity.

The mutational spectrum of the MM cell lines that we describe only partially recapitulates that of previous studies of primary MM samples.^12, 13, 14, 15, 38^ This is primarily because of the selection bias introduced by our targeted design that included all driver events identified by previous studies but excluded nonrecurrent or infrequent events that account for a large section of mutations described by NGS studies. Interestingly, MM cell lines share with primary samples of lymphoid malignancies a subset of recurrently mutated genes with questionable functional relevance.^32^ These findings highlight the need to curate any list of mutated genes accounting for, among other variables, the local mutation rate and synonymous-to-nonsynonymous ratio to identify real cancer driver genes that should be included in future clinical applications. To this end, here we combined the published data sets of mutations in primary MM samples to identify 14 potential recurrent driver genes with a rigorous statistical analysis^34^ that could be useful for future studies.

Our platform also identified relative copy number changes in regions with prognostic significance. A limitation of our analysis was that a targeted study does not allow automated and solid determination of the absolute ploidy of each cell line. Therefore, we were not able to identify the absolute copy number of each gene or chromosome segment, but only gains or losses relative to the average ploidy of normal cell lines. Nevertheless, we were able to identify changes in copy number of regions with prognostic significance with good accuracy, comparable to that of a SNP array.

Among our objectives was that of describing events involving the IGH locus. Translocations and rearrangements involving this locus are of clinical value as they can inform prognosis and minimal residual disease. Currently, fluorescence *in situ* hybridization and PCR techniques are used for this purpose with variable sensitivity.^39, 40^ Our strategy based on DNA pull-down and NGS performed extremely well in the detection of translocations, but less so for V(D)J rearrangement. In fact, we tried to reconstruct the pattern of V(D)J recombination in our samples looking for paired reads mapping on both sides of regions of decreased coverage (indicative of genomic deletions). We were able to identify, with high-confidence, cell-specific rearrangements in 12/14 MM cell lines (Supplementary Figure S2 and Supplementary Table S6), although visual inspection of the coverage plots suggests that our sensitivity was rather low and many deletions were missed. The presence of repetitive regions, the high homology of many segments and the incomplete annotation of the locus are all factors that can influence the efficiency of pull-down and mapping of sequences belonging to the IGH locus.

Although better mapping could be facilitated by newer iterations of the reference genome, our strategy already offers a list of clonal and subclonal markers in the form of either gene mutations, copy number changes, IGH translocations or rearrangements that can be used to track tumor evolution over time in clinical settings. Furthermore, parallel RNA-sequencing approaches could add relevant information on expression of mutated genes and overall transcriptional dysregulation of the disease.^41^ Given the high heterogeneity of myeloma samples, such NGS strategies designed to identify different tumor subpopulations followed by appropriate treatment selection are needed if disease eradication is to be the next goal of novel therapies.

In conclusion, we describe target-enrichment, sequencing techniques and analysis tools that can be implemented in diagnostic and research laboratories and can be deployed in the study of myeloma pathogenesis, diagnosis and prognosis. Application of this panel to large cohorts of clinically annotated patient samples will potentially allow discovery of novel biomarkers with clinical significance.

## Figures and Tables

**Table 1:** Significant genes in myeloma from the MutSigCV2 algorithm

*Gene*	P-*value*	q*-Value*	*% Of patients*	*% Of MM cell lines*
*KRAS*	1.00E−16	6.23E−13	23.3	14.3
*NRAS*	1.00E−16	6.23E−13	19.9	21.4
*TP53*	1.00E−16	6.23E−13	8.6	21.4
*BRAF*	1.18E−10	3.68E−07	8.6	7.1
*DIS3*	2.55E−12	1.19E−08	8.2	14.3
*FAM46C*	7.34E−12	2.75E−08	7.5	21.4
*PRKD2*	3.67E−05	5.26E−02	3.4	14.3
*TRAF3*	1.17E−06	3.11E−03	3.1	21.4
*PRDM1*	1.42E−05	2.65E−02	2.7	0.0
*ATP13A4*	1.33E−06	3.11E−03	2.4	7.1
*IRF4*	3.83E−06	7.96E−03	1.7	7.1
*INTS12*	3.06E−05	4.77E−02	1.4	0.0
*MAX*	3.94E−05	5.26E−02	1.4	0.0
*PDE1B*	1.66E−05	2.82E−02	1.0	0.0

Abbreviation: MM, multiple myeloma.

**Table 2:** Translocations as identified by NGS analysis

*Cell line*	*Translocation(s)*	*Validated*
ARH-77	No	NA
CTV-1	No	NA
IM-9	No	NA
KMS-11	t(4;14); t(14;16)	Yes, yes
KMS-12-BM	t(11;14)	Failed
L-363	t(11;14)	Yes
LP-1	t(4;14), t(8;14)	Yes, yes
MC-CAR	No	NA
MM1S	t(8;14); t(14;16)	Yes, yes
NCI-H929	t(4;14)	No DNA
OPM-2	t(4;14), t(8;14)	Yes, yes
SK-MM-2	t(11;14)	Failed
U-266	t(11;14)	Yes
RPMI-8226	No	NA

Abbreviations: NA, not applicable; NGS, next-generation sequencing.
