# Supplementary material for: A DNA target-enrichment approach to detect mutations, copy number changes and immunoglobulin translocations in multiple myeloma
Source: Blood Cancer J. 2016 Sep 2;6(9):e467–. doi: 10.1038/bcj.2016.72 (PMC5056967; doi:10.1038/bcj.2016.72)
Supplement: Supplementary Figure Legends [file bcj201672x1.docx]

### Legend to Supplementary Figure 1. Comparison between native and WGA DNA

Scatter plot of allelic frequencies of 24 variants identified in native DNA in a WES study (X axis) and in WGA DNA in the custom targeted study (Y axis). Substitutions are represented by circles and indels by triangles. Variants identified in both studies are solid, whereas blank circles identify variants reported by the targeted study only. The line represents the correlation between the allelic frequencies of the two studies: r is the Pearson's product moment correlation coefficient, p is the statistical significance of the test.

### Legend to Supplementary Figure 2. Comparison between native and WGA DNA

Schematic representation of the IGH locus in 12 myeloma cell lines. Annotation of the IgH exons as in Figure 5. Above, solid line connectors identify deletions reported by our algorithm, and breakpoints are projected on the X-axis (genomic coordinated of chr14) by grey dashed lines. Scatterplot represents coverage of individual base-pairs of the IgH locus in absolute values (Y-axis), with most deletions occurring at sites of coverage change.
