# Supplementary material for: A DNA target-enrichment approach to detect mutations, copy number changes and immunoglobulin translocations in multiple myeloma
Source: Blood Cancer J. 2016 Sep 2;6(9):e467–. doi: 10.1038/bcj.2016.72 (PMC5056967; doi:10.1038/bcj.2016.72)
Supplement: Supplementary Table 1 [file bcj201672x4.pdf]

Name	Tissue
ARH-77	MM
KMS-12-BM	MM
L-363	MM
LP-1	MM
OPM-2	MM
RPMI-8226	MM
SK-MM-2	MM
U-266	MM
CTV-1	MM
IM-9	MM
KMS-11	MM
MC-CAR	MM
MM1S	MM
NCI-H929	MM
EHEB	CLL
JVM-3	CLL
HL-60	AML
KASUMI-1	AML
CESS	ALL
MN-60	ALL
BALL-1	ALL
KARPAS-45	ALL
K-562	CML
RPMI-8866	CML
