# Supplementary material for: A DNA target-enrichment approach to detect mutations, copy number changes and immunoglobulin translocations in multiple myeloma
Source: Blood Cancer J. 2016 Sep 2;6(9):e467–. doi: 10.1038/bcj.2016.72 (PMC5056967; doi:10.1038/bcj.2016.72)
Supplement: Supplementary Table 2 [file bcj201672x5.pdf]

Chromosome	Start	End
chr1	776486	776606
chr1	3786185	3786305
chr1	6790886	6791006
chr1	9795659	9795779
chr1	12806136	12806256
chr1	15808707	15808827
chr1	16174503	16174703
chr1	16199290	16199650
chr1	16200589	16200909
chr1	16202674	16203194
chr1	16235755	16236035
chr1	16237575	16237815
chr1	16242558	16242838
chr1	16245363	16245603
chr1	16245835	16246075
chr1	16247301	16247541
chr1	16248673	16248913
chr1	16254556	16262476
chr1	16262507	16262787
chr1	16263638	16264158
chr1	16264283	16264523
chr1	16265151	16265431
chr1	16265736	16265976
chr1	18815274	18815394
chr1	21821697	21821817
chr1	23885400	23885560
chr1	23885607	23885927
chr1	24018216	24018416
chr1	24019033	24019313
chr1	24020229	24020469
chr1	24021098	24021338
chr1	24022234	24022474
chr1	24022768	24022928
chr1	24827769	24827889
chr1	27019951	27020071
chr1	27021634	27021754
chr1	27022882	27024042
chr1	27029490	27029610
chr1	27030958	27031078
chr1	27041570	27041795
chr1	27041956	27042076
chr1	27049760	27049880

chr1	27051995	27052227
chr1	27054768	27054888
chr1	27056127	27056367
chr1	27057628	27058108
chr1	27059124	27059324
chr1	27067356	27067476
chr1	27068141	27068261
chr1	27068960	27069080
chr1	27087326	27087606
chr1	27087819	27088019
chr1	27088586	27088866
chr1	27089459	27089779
chr1	27092664	27093102
chr1	27093596	27093716
chr1	27094265	27094505
chr1	27097593	27097833
chr1	27098936	27099176
chr1	27099250	27099530
chr1	27099791	27100440
chr1	27100805	27101725
chr1	27102012	27102526
chr1	27105500	27107260
chr1	27853399	27853519
chr1	30861795	30861915
chr1	33895794	33895914
chr1	36897743	36897863
chr1	39908666	39908786
chr1	42908788	42908908
chr1	43803478	43803638
chr1	43803715	43803955
chr1	43804161	43804441
chr1	43804930	43805250
chr1	43805575	43805855
chr1	43806000	43806240
chr1	43812067	43812347
chr1	43812413	43812653
chr1	43814473	43814713
chr1	43814884	43815164
chr1	43817830	43818030
chr1	43818175	43818455
chr1	45794923	45795163
chr1	45796128	45796288
chr1	45796789	45797279

chr1	45797306	45797546
chr1	45797626	45798690
chr1	45798705	45799056
chr1	45799059	45799299
chr1	45800002	45800242
chr1	45805518	45805678
chr1	45805828	45805988
chr1	45953973	45954093
chr1	48986485	48986605
chr1	50088758	50088878
chr1	50595922	50596042
chr1	51103221	51103341
chr1	51427504	51427624
chr1	51435984	51436224
chr1	51437186	51437306
chr1	51438304	51438424
chr1	51438820	51438940
chr1	51439553	51439953
chr1	51440032	51440152
chr1	51441900	51442020
chr1	51443380	51443500
chr1	51448098	51448218
chr1	51449033	51449153
chr1	51449514	51449634
chr1	51450419	51450581
chr1	51450659	51450779
chr1	51452502	51452622
chr1	51457954	51458074
chr1	51632903	51633023
chr1	52016663	52016783
chr1	52034752	52034872
chr1	52431751	52431871
chr1	52836078	52836198
chr1	53320213	53320333
chr1	53808597	53808717
chr1	54320322	54320442
chr1	54831740	54831860
chr1	55046332	55046452
chr1	55333392	55333512
chr1	55828579	55828699
chr1	56325896	56326016
chr1	56826158	56826278
chr1	58068059	58068179

chr1	61140956	61141076
chr1	64153592	64153712
chr1	65885508	65885628
chr1	67159598	67159718
chr1	70176185	70176305
chr1	73239110	73239230
chr1	74941232	74941352
chr1	76251951	76252071
chr1	79264668	79264788
chr1	82276526	82276646
chr1	84722609	84722729
chr1	85298051	85298171
chr1	88327700	88327820
chr1	91444132	91444252
chr1	93297572	93297772
chr1	93298880	93299279
chr1	93300282	93300522
chr1	93301729	93301969
chr1	93302961	93303241
chr1	93306051	93306251
chr1	93307272	93307472
chr1	94465401	94465521
chr1	95633916	95634036
chr1	97543704	97543824
chr1	99127237	99127517
chr1	99128216	99128416
chr1	99129442	99129562
chr1	99141387	99141507
chr1	99144415	99144535
chr1	99150391	99150671
chr1	99156585	99156785
chr1	99157032	99157312
chr1	99161032	99161312
chr1	99164221	99164501
chr1	99167315	99167515
chr1	99173620	99173740
chr1	99179877	99179997
chr1	99180117	99180237
chr1	99180794	99181017
chr1	99196658	99196890
chr1	99198928	99199048
chr1	99199893	99200013
chr1	99202461	99202581

chr1	99203748	99203988
chr1	99207517	99207637
chr1	99209480	99209728
chr1	99209935	99210055
chr1	99210697	99210817
chr1	99225007	99225127
chr1	99225568	99225728
chr1	100592593	100592713
chr1	103612009	103612129
chr1	106687889	106688009
chr1	109714343	109714463
chr1	112721855	112721975
chr1	115241795	115241915
chr1	115242376	115242496
chr1	115251115	115251378
chr1	115251548	115251668
chr1	115252149	115252389
chr1	115254346	115254466
chr1	115255656	115255776
chr1	115256369	115256728
chr1	115257460	115257580
chr1	115258625	115258825
chr1	115259707	115259827
chr1	115262063	115262183
chr1	115263617	115263882
chr1	115264002	115264122
chr1	115266245	115266365
chr1	115737159	115737279
chr1	116717495	116717721
chr1	117273516	117273636
chr1	117746150	117746270
chr1	118155992	118156112
chr1	118158026	118158146
chr1	118161999	118162119
chr1	118164733	118164853
chr1	118165152	118165312
chr1	118165392	118166678
chr1	118166689	118166809
chr1	118166816	118166936
chr1	118167138	118167258
chr1	118172160	118172280
chr1	118172477	118172597
chr1	118174161	118174281

chr1	118242803	118242923
chr1	118744163	118744283
chr1	118760110	118760230
chr1	119277778	119277898
chr1	119799604	119799724
chr1	120309380	120309500
chr1	120457922	120459322
chr1	120460236	120460436
chr1	120460982	120461222
chr1	120461925	120462245
chr1	120462795	120463075
chr1	120464283	120464483
chr1	120464843	120465083
chr1	120465209	120465449
chr1	120466253	120466613
chr1	120467920	120468440
chr1	120469077	120469277
chr1	120471596	120471836
chr1	120478040	120478280
chr1	120479856	120480136
chr1	120480436	120480676
chr1	120483158	120483398
chr1	120484142	120484382
chr1	120490992	120491232
chr1	120491589	120491789
chr1	120493303	120493503
chr1	120496118	120496358
chr1	120497618	120497898
chr1	120501969	120502169
chr1	120506193	120506433
chr1	120508032	120508232
chr1	120508955	120509155
chr1	120510009	120510289
chr1	120510657	120510897
chr1	120512130	120512370
chr1	120529523	120529763
chr1	120539607	120539967
chr1	120547941	120548221
chr1	120572469	120572669
chr1	120611903	120612063
chr1	121164546	121164666
chr1	121184837	121184957
chr1	144474481	144474602

chr1	145092905	145093025
chr1	145588475	145588595
chr1	145736399	145736519
chr1	146535341	146535461
chr1	147048539	147048659
chr1	147375904	147376024
chr1	147743557	147743677
chr1	150000104	150000224
chr1	150749853	150749973
chr1	153753665	153753785
chr1	154554813	154554973
chr1	154557260	154557540
chr1	154557636	154557876
chr1	154558164	154558404
chr1	154558627	154558867
chr1	154560547	154560787
chr1	154560967	154561207
chr1	154561791	154561991
chr1	154562178	154562458
chr1	154562632	154562912
chr1	154569255	154569495
chr1	154569530	154569810
chr1	154570237	154570517
chr1	154570849	154571089
chr1	154573489	154575129
chr1	154580374	154580574
chr1	154600262	154600542
chr1	156754405	156754525
chr1	159802567	159802687
chr1	160129424	160129544
chr1	160626322	160626442
chr1	161162112	161162232
chr1	162148638	162148758
chr1	162640588	162640708
chr1	162813591	162813711
chr1	163126356	163126476
chr1	163587294	163587414
chr1	164081994	164082114
chr1	164568545	164568665
chr1	165080972	165081092
chr1	165589476	165589596
chr1	165835304	165835424
chr1	166037848	166037968

chr1	168849913	168850033
chr1	169676425	169676545
chr1	171794847	171794967
chr1	171901745	171901865
chr1	174971622	174971742
chr1	177989267	177989387
chr1	181002419	181002539
chr1	184002814	184002934
chr1	187005250	187005370
chr1	190010185	190010305
chr1	193054094	193054214
chr1	193091282	193091522
chr1	193094165	193094405
chr1	193099227	193099427
chr1	193104451	193104772
chr1	193107158	193107358
chr1	193110967	193111207
chr1	193116945	193117145
chr1	193119372	193119572
chr1	193121441	193121641
chr1	193172873	193173033
chr1	193181132	193181292
chr1	193181463	193181663
chr1	193202063	193202343
chr1	193205315	193205555
chr1	193218790	193219070
chr1	193219743	193219903
chr1	196061651	196061771
chr1	199102550	199102670
chr1	202116178	202116298
chr1	202698112	202698352
chr1	202698814	202699174
chr1	202699993	202700233
chr1	202700911	202701111
chr1	202702511	202703031
chr1	202704515	202704755
chr1	202705290	202705570
chr1	202709750	202709990
chr1	202710492	202710852
chr1	202711475	202711675
chr1	202711737	202711977
chr1	202714927	202715167
chr1	202715220	202715500

chr1	202718029	202718309
chr1	202719746	202719946
chr1	202721973	202722253
chr1	202724349	202724629
chr1	202725444	202725684
chr1	202727478	202727678
chr1	202729501	202729741
chr1	202731781	202731981
chr1	202733124	202733324
chr1	202735478	202735678
chr1	202736000	202736240
chr1	202742190	202742470
chr1	202743681	202743921
chr1	202746103	202746263
chr1	202777211	202777451
chr1	204452719	204452839
chr1	204494575	204494775
chr1	204495405	204495605
chr1	204499758	204499998
chr1	204501266	204501426
chr1	204501861	204502021
chr1	204505946	204506146
chr1	204506491	204506691
chr1	204506980	204507180
chr1	204507286	204507486
chr1	204511851	204512131
chr1	204513597	204513877
chr1	204515864	204516064
chr1	204518225	204518825
chr1	204522429	204522549
chr1	204527708	204527868
chr1	204551770	204551890
chr1	204566939	204567059
chr1	204586987	204587107
chr1	205127999	205128119
chr1	208130567	208130687
chr1	211187603	211187723
chr1	214192634	214192754
chr1	217199081	217199201
chr1	220218406	220218526
chr1	223240251	223240371
chr1	226242712	226242832
chr1	229260583	229260703

chr1	232269174	232269294
chr1	235324537	235324777
chr1	235331783	235332023
chr1	235335885	235336125
chr1	235338486	235338726
chr1	235340111	235340311
chr1	235341071	235341271
chr1	235344883	235346123
chr1	235349047	235349167
chr1	235354457	235354577
chr1	235357286	235357566
chr1	235359287	235359487
chr1	235359721	235359841
chr1	235362899	235363019
chr1	235363847	235363967
chr1	235371484	235371604
chr1	235375758	235375878
chr1	235377072	235377352
chr1	235383055	235383335
chr1	235383598	235383878
chr1	235384053	235384173
chr1	235384777	235385028
chr1	235386427	235386627
chr1	235387793	235387913
chr1	235387973	235388133
chr1	235392502	235392742
chr1	235394385	235394545
chr1	235397687	235397847
chr1	235403576	235403816
chr1	235409680	235409880
chr1	235416004	235416164
chr1	235418919	235419119
chr1	235420427	235420587
chr1	235423911	235424111
chr1	235429846	235429966
chr1	235438626	235438746
chr1	235466523	235466643
chr1	235478751	235478871
chr1	235489123	235489243
chr1	235490131	235490331
chr1	237048439	237048559
chr1	238388862	238388982
chr1	240492673	240492793

chr1	241399944	241400064
chr1	244404774	244404894
chr1	247404851	247404971
chr2	78943	79063
chr2	3078961	3079081
chr2	6087010	6087130
chr2	9133509	9133629
chr2	9347176	9347416
chr2	9419401	9419561
chr2	9437381	9437621
chr2	9458609	9458769
chr2	9460361	9460521
chr2	9463194	9463434
chr2	9467897	9468097
chr2	9474824	9474984
chr2	9475164	9475364
chr2	9484612	9484812
chr2	9484814	9484974
chr2	9490880	9491080
chr2	9496119	9496530
chr2	9498831	9499071
chr2	9508500	9508700
chr2	9514838	9515118
chr2	9516979	9517179
chr2	9519030	9519230
chr2	9520823	9520983
chr2	9525330	9525530
chr2	9528408	9528688
chr2	9531137	9531377
chr2	9533553	9533833
chr2	9540096	9540256
chr2	9540814	9541014
chr2	9541343	9541583
chr2	9543338	9543498
chr2	9796730	9796850
chr2	12145952	12146072
chr2	15148278	15148398
chr2	16036308	16036428
chr2	16082171	16082691
chr2	16082729	16082849
chr2	16082867	16083067
chr2	16084357	16084477
chr2	16085431	16085631

chr2	16085640	16086240
chr2	16088820	16088940
chr2	16098333	16098453
chr2	16111244	16111364
chr2	16112768	16112888
chr2	16127862	16127982
chr2	16136862	16136982
chr2	18214397	18214517
chr2	21218941	21219061
chr2	24288715	24288835
chr2	25457078	25457358
chr2	25458514	25458754
chr2	25459739	25459939
chr2	25461941	25462141
chr2	25462290	25462450
chr2	25463104	25463384
chr2	25463453	25463653
chr2	25464363	25464643
chr2	25466708	25466908
chr2	25466995	25467235
chr2	25467344	25467584
chr2	25468061	25468261
chr2	25468830	25469243
chr2	25469426	25469706
chr2	25469853	25470093
chr2	25470398	25470678
chr2	25470893	25471133
chr2	25472459	25472659
chr2	25474964	25475164
chr2	25497742	25498022
chr2	25498310	25498470
chr2	25505238	25505598
chr2	25522939	25523179
chr2	25536717	25536917
chr2	27290465	27290585
chr2	29416078	29416798
chr2	29419531	29419780
chr2	29420255	29420594
chr2	29429967	29430207
chr2	29430810	29431010
chr2	29432597	29432797
chr2	29436798	29436998
chr2	29443516	29443756

chr2	29443838	29444118
chr2	29445141	29445527
chr2	29446180	29446420
chr2	29448258	29448498
chr2	29449723	29450003
chr2	29450388	29450588
chr2	29451720	29451960
chr2	29453775	29453895
chr2	29455101	29455381
chr2	29456376	29456616
chr2	29462480	29462760
chr2	29473911	29474191
chr2	29497908	29498148
chr2	29498214	29498414
chr2	29512615	29512735
chr2	29519698	29519978
chr2	29541099	29541339
chr2	29543562	29543802
chr2	29551161	29551401
chr2	29606541	29606781
chr2	29672880	29673000
chr2	29754761	29755001
chr2	29791781	29791901
chr2	29917657	29917937
chr2	29940381	29940621
chr2	29966810	29966930
chr2	29986514	29986634
chr2	30026905	30027025
chr2	30035542	30035662
chr2	30048950	30049070
chr2	30111780	30111900
chr2	30142831	30143551
chr2	30299976	30300096
chr2	33300675	33300795
chr2	36304569	36304689
chr2	39403950	39404070
chr2	42418523	42418643
chr2	45420022	45420142
chr2	48423838	48423958
chr2	49189860	49189980
chr2	51488365	51488485
chr2	54502852	54502972
chr2	57515988	57516108

chr2	60518290	60518410
chr2	61705907	61706147
chr2	61708267	61708467
chr2	61709474	61709714
chr2	61710038	61710278
chr2	61711015	61711295
chr2	61712859	61713139
chr2	61713468	61713588
chr2	61715252	61715452
chr2	61715674	61715954
chr2	61717723	61717963
chr2	61719111	61719391
chr2	61719417	61719932
chr2	61719998	61720238
chr2	61720987	61721267
chr2	61722548	61722788
chr2	61723957	61724197
chr2	61725767	61726103
chr2	61726798	61727078
chr2	61727154	61727274
chr2	61729072	61729317
chr2	61729334	61729494
chr2	61731417	61731537
chr2	61732043	61732163
chr2	61735385	61735505
chr2	61747062	61747182
chr2	61749467	61749587
chr2	61749629	61749861
chr2	61752093	61752213
chr2	61753474	61753714
chr2	61756842	61756962
chr2	61757660	61757780
chr2	61760849	61761089
chr2	63518412	63518532
chr2	66567339	66567459
chr2	68166019	68166139
chr2	69578232	69578352
chr2	72789492	72789612
chr2	75802091	75802211
chr2	78805771	78805891
chr2	81832646	81832766
chr2	84833205	84833325
chr2	88319664	88319784

chr2	91812774	91812894
chr2	95536940	95537060
chr2	97193274	97193394
chr2	97197204	97197324
chr2	97198725	97198845
chr2	97199616	97199736
chr2	97201621	97201741
chr2	97202182	97202302
chr2	97202465	97202665
chr2	97202978	97203218
chr2	97206348	97206468
chr2	97207260	97207380
chr2	97208244	97208364
chr2	97212603	97212843
chr2	97213096	97213296
chr2	97215006	97215246
chr2	97215435	97215595
chr2	97215873	97216073
chr2	97216270	97216510
chr2	97216822	97218062
chr2	97218306	97218426
chr2	98543502	98543622
chr2	101546656	101546776
chr2	102314490	102314650
chr2	102314887	102315047
chr2	102320579	102320699
chr2	102322708	102322828
chr2	102325192	102325312
chr2	102331110	102331230
chr2	102336845	102336965
chr2	102343001	102343121
chr2	102348616	102348736
chr2	102350969	102351089
chr2	102359862	102359982
chr2	102367501	102367621
chr2	102374898	102375018
chr2	102378473	102378593
chr2	102391515	102391635
chr2	102396902	102397022
chr2	102403105	102403225
chr2	102407129	102407289
chr2	102412525	102412645
chr2	102413685	102413885

chr2	102415076	102415196
chr2	102421917	102422037
chr2	102429588	102429708
chr2	102438785	102438905
chr2	102440105	102440225
chr2	102440332	102440572
chr2	102441735	102441935
chr2	102443760	102443880
chr2	102443981	102444101
chr2	102445910	102446110
chr2	102447159	102447279
chr2	102448127	102448367
chr2	102450817	102450977
chr2	102452320	102452480
chr2	102456228	102456508
chr2	102457029	102457149
chr2	102459026	102459186
chr2	102460547	102460787
chr2	102462513	102462633
chr2	102467562	102467682
chr2	102472379	102472659
chr2	102475400	102475600
chr2	102476095	102476335
chr2	102477227	102477507
chr2	102478968	102479088
chr2	102480277	102480517
chr2	102481344	102481544
chr2	102482845	102483085
chr2	102483626	102483826
chr2	102486031	102486311
chr2	102486696	102486936
chr2	102487911	102488191
chr2	102490067	102490267
chr2	102490488	102490768
chr2	102493416	102493656
chr2	102495686	102495806
chr2	102498959	102499199
chr2	102500275	102500395
chr2	102501598	102501798
chr2	102503496	102503776
chr2	102504199	102504439
chr2	102505207	102505447
chr2	102507569	102507769

chr2	104563834	104563954
chr2	107641838	107641958
chr2	110859612	110859732
chr2	113862113	113862233
chr2	116867536	116867656
chr2	119886858	119886978
chr2	122966097	122966217
chr2	126042807	126042927
chr2	129052292	129052412
chr2	132077319	132077439
chr2	135080509	135080629
chr2	138080635	138080755
chr2	140990704	140990944
chr2	140992303	140992503
chr2	140995672	140995912
chr2	140996955	140997155
chr2	141004612	141004772
chr2	141026793	141026993
chr2	141027762	141027962
chr2	141031939	141032219
chr2	141055317	141055597
chr2	141072445	141072725
chr2	141079474	141079714
chr2	141081408	141081688
chr2	141083288	141083488
chr2	141091974	141092174
chr2	141093175	141093415
chr2	141108346	141108626
chr2	141110481	141110737
chr2	141113857	141114097
chr2	141115496	141115736
chr2	141116332	141116572
chr2	141122170	141122410
chr2	141128224	141128464
chr2	141128700	141128900
chr2	141130521	141130761
chr2	141135701	141135901
chr2	141143419	141143619
chr2	141200032	141200232
chr2	141201858	141202058
chr2	141202089	141202289
chr2	141208083	141208283
chr2	141213977	141214217

chr2	141214985	141215265
chr2	141232666	141232946
chr2	141242862	141243142
chr2	141245126	141245366
chr2	141250119	141250319
chr2	141253085	141253365
chr2	141259209	141259489
chr2	141260481	141260721
chr2	141264305	141264545
chr2	141267440	141267680
chr2	141272181	141272381
chr2	141274398	141274638
chr2	141283367	141283607
chr2	141283762	141283962
chr2	141291549	141291749
chr2	141294093	141294333
chr2	141298484	141298724
chr2	141299303	141299583
chr2	141356181	141356421
chr2	141358973	141359253
chr2	141457804	141458204
chr2	141459231	141459471
chr2	141459665	141459905
chr2	141459938	141460178
chr2	141473486	141473726
chr2	141474197	141474437
chr2	141526727	141526967
chr2	141528392	141528632
chr2	141533616	141533856
chr2	141571180	141571420
chr2	141597506	141597706
chr2	141598433	141598713
chr2	141607664	141607904
chr2	141609166	141609406
chr2	141625140	141625420
chr2	141625609	141625889
chr2	141641367	141641607
chr2	141643665	141643945
chr2	141660471	141660751
chr2	141665425	141665665
chr2	141680484	141680764
chr2	141707747	141708027
chr2	141709368	141709568

chr2	141739686	141739886
chr2	141747043	141747283
chr2	141751513	141751753
chr2	141762844	141763084
chr2	141771079	141771359
chr2	141773254	141773494
chr2	141777440	141777720
chr2	141806552	141806792
chr2	141812636	141812876
chr2	141816397	141816677
chr2	141819610	141819850
chr2	141945930	141946210
chr2	141986740	141987020
chr2	142004738	142004978
chr2	142012050	142012250
chr2	142237913	142238153
chr2	142567788	142568028
chr2	142888157	142888357
chr2	144139478	144139598
chr2	147155523	147155643
chr2	150204318	150204438
chr2	152342220	152342500
chr2	152346417	152346657
chr2	152346818	152347098
chr2	152348142	152348342
chr2	152348583	152348823
chr2	152348834	152349074
chr2	152349809	152350009
chr2	152350234	152350434
chr2	152350620	152350820
chr2	152352735	152352935
chr2	152353400	152353600
chr2	152354085	152354285
chr2	152354681	152354921
chr2	152355791	152355991
chr2	152357850	152358050
chr2	152359253	152359453
chr2	152359808	152360008
chr2	152361937	152362137
chr2	152362625	152362825
chr2	152363354	152363594
chr2	152364450	152364690
chr2	152369178	152369418

chr2	152370030	152370270
chr2	152370766	152371006
chr2	152371266	152371506
chr2	152372904	152373144
chr2	152374763	152375003
chr2	152375410	152375650
chr2	152376139	152376379
chr2	152380756	152381187
chr2	152381611	152381851
chr2	152382406	152382843
chr2	152383363	152383603
chr2	152383926	152384166
chr2	152385655	152385895
chr2	152387441	152387681
chr2	152388237	152388477
chr2	152389887	152390127
chr2	152390660	152390900
chr2	152392136	152392376
chr2	152393577	152393817
chr2	152394317	152394557
chr2	152394585	152394825
chr2	152396789	152397029
chr2	152397142	152397382
chr2	152397892	152398132
chr2	152402336	152402576
chr2	152402807	152403007
chr2	152403874	152404319
chr2	152404755	152404995
chr2	152406081	152406321
chr2	152408185	152408425
chr2	152409117	152409357
chr2	152409843	152410083
chr2	152410320	152410560
chr2	152411397	152411637
chr2	152417039	152417279
chr2	152417449	152417893
chr2	152418555	152418795
chr2	152419103	152419343
chr2	152420056	152420510
chr2	152421462	152421702
chr2	152421946	152422401
chr2	152423657	152424017
chr2	152424518	152424758

chr2	152424760	152425000
chr2	152425080	152425280
chr2	152425713	152425953
chr2	152426570	152426930
chr2	152426942	152427182
chr2	152432140	152432380
chr2	152432646	152432886
chr2	152435827	152436187
chr2	152437244	152437484
chr2	152437928	152438168
chr2	152438959	152439199
chr2	152439944	152440304
chr2	152441954	152442194
chr2	152442850	152443090
chr2	152443869	152444109
chr2	152446378	152446738
chr2	152447795	152448035
chr2	152448479	152448719
chr2	152449511	152449751
chr2	152450496	152450856
chr2	152452507	152452747
chr2	152453403	152453643
chr2	152454422	152454662
chr2	152456931	152457291
chr2	152458348	152458588
chr2	152459032	152459272
chr2	152460064	152460304
chr2	152461049	152461409
chr2	152463053	152463293
chr2	152463949	152464189
chr2	152464968	152465208
chr2	152466298	152466658
chr2	152466963	152467203
chr2	152467207	152467447
chr2	152468681	152468921
chr2	152470765	152471125
chr2	152472449	152472689
chr2	152473809	152474049
chr2	152474770	152475010
chr2	152475940	152476300
chr2	152477366	152477606
chr2	152481979	152482219
chr2	152483497	152483737

chr2	152484012	152484372
chr2	152485986	152486226
chr2	152487160	152487400
chr2	152487638	152487878
chr2	152490143	152490503
chr2	152492674	152492914
chr2	152495725	152495965
chr2	152496352	152496592
chr2	152496844	152497204
chr2	152499021	152499450
chr2	152499645	152499885
chr2	152500307	152500667
chr2	152500915	152501155
chr2	152502575	152502815
chr2	152506671	152506911
chr2	152507063	152507423
chr2	152510439	152510679
chr2	152511763	152511963
chr2	152512313	152512553
chr2	152512642	152513002
chr2	152514430	152514670
chr2	152515510	152515750
chr2	152518631	152518871
chr2	152520037	152520397
chr2	152520948	152521188
chr2	152521204	152521444
chr2	152521829	152522069
chr2	152522579	152522939
chr2	152524254	152524494
chr2	152525472	152525712
chr2	152527519	152527759
chr2	152528858	152529218
chr2	152530924	152531164
chr2	152531732	152531972
chr2	152534061	152534301
chr2	152534365	152534725
chr2	152536168	152536603
chr2	152537192	152537392
chr2	152539109	152539349
chr2	152541270	152541510
chr2	152543869	152544313
chr2	152544737	152544977
chr2	152547189	152547389

chr2	152548309	152549054
chr2	152550773	152551209
chr2	152552023	152552263
chr2	152553099	152553299
chr2	152553593	152554229
chr2	152563332	152563572
chr2	152566103	152566343
chr2	152566879	152567119
chr2	152572460	152572660
chr2	152573861	152574101
chr2	152579827	152580067
chr2	152580705	152580945
chr2	152581302	152581542
chr2	152581900	152582140
chr2	152584192	152584432
chr2	152586069	152586229
chr2	152589572	152589732
chr2	153211224	153211344
chr2	156312058	156312178
chr2	159316523	159316643
chr2	159694688	159694808
chr2	162551658	162551778
chr2	165558192	165558312
chr2	168559935	168560055
chr2	171564989	171565109
chr2	174584557	174584677
chr2	177595802	177595922
chr2	178095484	178096764
chr2	178097095	178097335
chr2	178097846	178098126
chr2	178098705	178099025
chr2	178129201	178129361
chr2	179871111	179871231
chr2	180603977	180604097
chr2	182543394	182543514
chr2	183679361	183679481
chr2	186807208	186807328
chr2	189849713	189849833
chr2	192965879	192965999
chr2	195974303	195974423
chr2	198256985	198257225
chr2	198257683	198257923
chr2	198260775	198261055

chr2	198262654	198262894
chr2	198263124	198263364
chr2	198263642	198263802
chr2	198264734	198265206
chr2	198265429	198265669
chr2	198266066	198266306
chr2	198266418	198266658
chr2	198266661	198266901
chr2	198267274	198267554
chr2	198267615	198267815
chr2	198268258	198268538
chr2	198269750	198269950
chr2	198269957	198270237
chr2	198272662	198272902
chr2	198273078	198273318
chr2	198274492	198274732
chr2	198281409	198281689
chr2	198283192	198283352
chr2	198283565	198283765
chr2	198285043	198285323
chr2	198285704	198285904
chr2	198288474	198288754
chr2	198299609	198299809
chr2	198977761	198977881
chr2	201983367	201983487
chr2	202122909	202123149
chr2	202123369	202123489
chr2	202124942	202125062
chr2	202126877	202126997
chr2	202127656	202127776
chr2	202127802	202127922
chr2	202128557	202128677
chr2	202131168	202131528
chr2	202132046	202132166
chr2	202134180	202134380
chr2	202136191	202136391
chr2	202137309	202137549
chr2	202137562	202137722
chr2	202138301	202138421
chr2	202139563	202139723
chr2	202141500	202141740
chr2	202142971	202143091
chr2	202143155	202143275

chr2	202143867	202143987
chr2	202149529	202150049
chr2	202151129	202151369
chr2	204988972	204989092
chr2	207992095	207992215
chr2	209101747	209101947
chr2	209103735	209104015
chr2	209104516	209104796
chr2	209106653	209106933
chr2	209108099	209108379
chr2	209109975	209110215
chr2	209113076	209113396
chr2	209116094	209116334
chr2	211059989	211060109
chr2	211138749	211138869
chr2	214156325	214156445
chr2	217184655	217184775
chr2	220191801	220191921
chr2	223251404	223251524
chr2	226273430	226273550
chr2	229296905	229297025
chr2	231090508	231090668
chr2	231097062	231097182
chr2	231101746	231102026
chr2	231102871	231103156
chr2	231103172	231103292
chr2	231103431	231103631
chr2	231106060	231106260
chr2	231107084	231107204
chr2	231107768	231107888
chr2	231107892	231108012
chr2	231108067	231108187
chr2	231108385	231108585
chr2	231109648	231109848
chr2	231110536	231110696
chr2	231111361	231111481
chr2	231112585	231112825
chr2	231113541	231113741
chr2	231115635	231115835
chr2	231117981	231118181
chr2	231120106	231120306
chr2	231130826	231130946
chr2	231134189	231134389

chr2	231134509	231134709
chr2	231135247	231135407
chr2	231149013	231149173
chr2	231150406	231150606
chr2	231152563	231152723
chr2	231155126	231155326
chr2	231155643	231155763
chr2	231157311	231157551
chr2	231158929	231159089
chr2	231162076	231162236
chr2	231174596	231174796
chr2	231175411	231175611
chr2	231175827	231175987
chr2	231176118	231176358
chr2	231177249	231177449
chr2	231181021	231181141
chr2	231183260	231183380
chr2	231183725	231183845
chr2	231188990	231189110
chr2	231222434	231222634
chr2	232301610	232301730
chr2	234601608	234601728
chr2	235303121	235303241
chr2	238313136	238313256
chr2	241328689	241328809
chr3	66834	66954
chr3	3067790	3067910
chr3	3192498	3193007
chr3	3193327	3193447
chr3	3193615	3193735
chr3	3194085	3194325
chr3	3195059	3195219
chr3	3195601	3195801
chr3	3196372	3196572
chr3	3197001	3197121
chr3	3197853	3198013
chr3	3198319	3198439
chr3	3198452	3198572
chr3	3199504	3199731
chr3	3201719	3201839
chr3	3204743	3204863
chr3	3205371	3205491
chr3	3209251	3209531

chr3	3211494	3211614
chr3	3214414	3214654
chr3	3215723	3215963
chr3	3216799	3216999
chr3	3220428	3220548
chr3	3220573	3220693
chr3	3221257	3221417
chr3	6081767	6081887
chr3	8817200	8817400
chr3	8922971	8923211
chr3	8932001	8932201
chr3	8940514	8940794
chr3	8943993	8944273
chr3	8953925	8954125
chr3	8955265	8955465
chr3	8977526	8977766
chr3	8981187	8981387
chr3	8983139	8983499
chr3	8988838	8989038
chr3	8990123	8990323
chr3	9000544	9000744
chr3	9004963	9005123
chr3	9098363	9098483
chr3	10070273	10070473
chr3	10074445	10074725
chr3	10076086	10076286
chr3	10076350	10076590
chr3	10076786	10076986
chr3	10077916	10078076
chr3	10080901	10081101
chr3	10081342	10081622
chr3	10082161	10082361
chr3	10083250	10083450
chr3	10084174	10084414
chr3	10084663	10084903
chr3	10085101	10085341
chr3	10085450	10085610
chr3	10088195	10088475
chr3	10089547	10089787
chr3	10091003	10091243
chr3	10093985	10094225
chr3	10101912	10102152
chr3	10105415	10105655

chr3	10105976	10106176
chr3	10106345	10106625
chr3	10107007	10107247
chr3	10107485	10107725
chr3	10108826	10109066
chr3	10114489	10114729
chr3	10114871	10115111
chr3	10116145	10116425
chr3	10119702	10119942
chr3	10122727	10122967
chr3	10122968	10123208
chr3	10127430	10127670
chr3	10128744	10128984
chr3	10130079	10130279
chr3	10130452	10130692
chr3	10131922	10132122
chr3	10133800	10134000
chr3	10134907	10135067
chr3	10135909	10136109
chr3	10136820	10137020
chr3	10137942	10138222
chr3	10140378	10140658
chr3	10142808	10143008
chr3	12117160	12117280
chr3	15152702	15152822
chr3	18179927	18180047
chr3	21251259	21251379
chr3	24263678	24263798
chr3	27336153	27336273
chr3	30342106	30342226
chr3	30648322	30648522
chr3	30664694	30664854
chr3	30686182	30686462
chr3	30691736	30691976
chr3	30702949	30703189
chr3	30713109	30713949
chr3	30715527	30715807
chr3	30729819	30730059
chr3	30732861	30733141
chr3	33419362	33419482
chr3	36425476	36425596
chr3	37034971	37035251
chr3	37038054	37038254

chr3	37042394	37042594
chr3	37045828	37046028
chr3	37048417	37048617
chr3	37050250	37050450
chr3	37053251	37053411
chr3	37053445	37053645
chr3	37055858	37056098
chr3	37058943	37059143
chr3	37061737	37062017
chr3	37067114	37067514
chr3	37070208	37070488
chr3	37081610	37081850
chr3	37083687	37083887
chr3	37088951	37089231
chr3	37089953	37090153
chr3	37090331	37090571
chr3	37091920	37092200
chr3	37107008	37107208
chr3	38180135	38180535
chr3	38181301	38181541
chr3	38181860	38182100
chr3	38182193	38182393
chr3	38182579	38182819
chr3	39448521	39448641
chr3	42492453	42492573
chr3	45649357	45649477
chr3	47058523	47058803
chr3	47059078	47059278
chr3	47061189	47061389
chr3	47064375	47064495
chr3	47070435	47070555
chr3	47079111	47079311
chr3	47084000	47084240
chr3	47087923	47088163
chr3	47093577	47093697
chr3	47098305	47098985
chr3	47103604	47103884
chr3	47106011	47106211
chr3	47108503	47108663
chr3	47110661	47110781
chr3	47114843	47114963
chr3	47121333	47121453
chr3	47125200	47125880

chr3	47127644	47127844
chr3	47129549	47129789
chr3	47138620	47138740
chr3	47139387	47139627
chr3	47142896	47143096
chr3	47144794	47144954
chr3	47147428	47147668
chr3	47155309	47155549
chr3	47158058	47158298
chr3	47161156	47161356
chr3	47161654	47166054
chr3	47168045	47168245
chr3	47170546	47170666
chr3	47177327	47177447
chr3	47205298	47205458
chr3	49212970	49213090
chr3	52236702	52236822
chr3	52540713	52540833
chr3	52582024	52582304
chr3	52584424	52584664
chr3	52584697	52584897
chr3	52588677	52588957
chr3	52592206	52592486
chr3	52595763	52596003
chr3	52597283	52597523
chr3	52598037	52598277
chr3	52610495	52610775
chr3	52613002	52613282
chr3	52620412	52620732
chr3	52621307	52621587
chr3	52623058	52623298
chr3	52637522	52637762
chr3	52643309	52643989
chr3	52649299	52649539
chr3	52651255	52651575
chr3	52658854	52659014
chr3	52661237	52661437
chr3	52662840	52663120
chr3	52668604	52668844
chr3	52675915	52676115
chr3	52677211	52677411
chr3	52682333	52682533
chr3	52685691	52685891

chr3	52692152	52692392
chr3	52696080	52696360
chr3	52702403	52702683
chr3	52712464	52712664
chr3	52713538	52713778
chr3	55263411	55263531
chr3	58269652	58269772
chr3	59736523	59736643
chr3	61315290	61315410
chr3	64327965	64328085
chr3	67356917	67357037
chr3	68434542	68434662
chr3	70374130	70374250
chr3	71007364	71007524
chr3	71008329	71008609
chr3	71014983	71015263
chr3	71019821	71020021
chr3	71021106	71021386
chr3	71021646	71021886
chr3	71026022	71026262
chr3	71026733	71026933
chr3	71026959	71027199
chr3	71037086	71037286
chr3	71050066	71050266
chr3	71064631	71064871
chr3	71090460	71090700
chr3	71096026	71096306
chr3	71101632	71101832
chr3	71102735	71102975
chr3	71161617	71161857
chr3	71179621	71179861
chr3	71246960	71247240
chr3	71247302	71247582
chr3	73411610	73411730
chr3	75986598	75986798
chr3	76126106	76126226
chr3	76224979	76225099
chr3	76439689	76439810
chr3	76467134	76467254
chr3	76469521	76469641
chr3	76480995	76481115
chr3	76489969	76490089
chr3	76735732	76735852

chr3	76763429	76763549
chr3	76780430	76780550
chr3	77089886	77090046
chr3	77147147	77147507
chr3	77235849	77235969
chr3	77246668	77246788
chr3	77526523	77526763
chr3	77530189	77530429
chr3	77531865	77531985
chr3	77542343	77542583
chr3	77571869	77572109
chr3	77595430	77595670
chr3	77598882	77599002
chr3	77599914	77600194
chr3	77607077	77607317
chr3	77611742	77611942
chr3	77612258	77612538
chr3	77613506	77613706
chr3	77614047	77614327
chr3	77617404	77617644
chr3	77623645	77623885
chr3	77626582	77626822
chr3	77629043	77629323
chr3	77637852	77638132
chr3	77644233	77644393
chr3	77645717	77645957
chr3	77651341	77651661
chr3	77652028	77652268
chr3	77656906	77657146
chr3	77666655	77666935
chr3	77671360	77671600
chr3	77675577	77675697
chr3	77681575	77681855
chr3	77683967	77684247
chr3	77691817	77692097
chr3	77693834	77694074
chr3	77695106	77695306
chr3	78647969	78648169
chr3	78649220	78649500
chr3	78655876	78656196
chr3	78663753	78664015
chr3	78666767	78667207
chr3	78676361	78676734

chr3	78680262	78680502
chr3	78683038	78683238
chr3	78683533	78683773
chr3	78684910	78685270
chr3	78688850	78689090
chr3	78695238	78695398
chr3	78696691	78696891
chr3	78700840	78701120
chr3	78706193	78706473
chr3	78708776	78709016
chr3	78710175	78710415
chr3	78711083	78711323
chr3	78716975	78717507
chr3	78717568	78717768
chr3	78719228	78719468
chr3	78734841	78735121
chr3	78737739	78737979
chr3	78742401	78742601
chr3	78744145	78744265
chr3	78763490	78763730
chr3	78766373	78766613
chr3	78766852	78767092
chr3	78775506	78775626
chr3	78795851	78796091
chr3	78877156	78877276
chr3	78985737	78985857
chr3	78987733	78988093
chr3	79067503	79067663
chr3	79174547	79174747
chr3	79190850	79190970
chr3	79192683	79192803
chr3	79192923	79193043
chr3	79412131	79412251
chr3	79429749	79429869
chr3	79522042	79522350
chr3	79522398	79522518
chr3	79533034	79533155
chr3	79569497	79569617
chr3	79582140	79582340
chr3	79585097	79585217
chr3	79634005	79634125
chr3	79638917	79639117
chr3	80549196	80549316

chr3	82545930	82546050
chr3	85570501	85570621
chr3	88585021	88585141
chr3	89156842	89157042
chr3	89176290	89176490
chr3	89258979	89259699
chr3	89390003	89390283
chr3	89390892	89391252
chr3	89444928	89445168
chr3	89448441	89448681
chr3	89456349	89456589
chr3	89457148	89457348
chr3	89462233	89462473
chr3	89468327	89468567
chr3	89478186	89478386
chr3	89480284	89480524
chr3	89498309	89498589
chr3	89499303	89499543
chr3	89521551	89521831
chr3	89528479	89528719
chr3	93641473	93641593
chr3	96664184	96664304
chr3	99679401	99679521
chr3	102827890	102828010
chr3	105840951	105841071
chr3	108867068	108867188
chr3	111873936	111874056
chr3	114957075	114957195
chr3	117800484	117800604
chr3	117958339	117958459
chr3	121015764	121015884
chr3	124017936	124018056
chr3	127037617	127037737
chr3	130060660	130060780
chr3	133088302	133088422
chr3	136094161	136094281
chr3	138374179	138374419
chr3	138374929	138375169
chr3	138376464	138376744
chr3	138382689	138382929
chr3	138383821	138384101
chr3	138400755	138400955
chr3	138402454	138402694

chr3	138403415	138403695
chr3	138407666	138407866
chr3	138409773	138410053
chr3	138413568	138413808
chr3	138417722	138417962
chr3	138423229	138423389
chr3	138425945	138426185
chr3	138430998	138431198
chr3	138433295	138433575
chr3	138452141	138452341
chr3	138453420	138453700
chr3	138456498	138456778
chr3	138461393	138461633
chr3	138474592	138474832
chr3	138477959	138478239
chr3	139101717	139101837
chr3	141205871	141206111
chr3	141207415	141207645
chr3	141212457	141212577
chr3	141213526	141213646
chr3	141217996	141218116
chr3	141230963	141231163
chr3	141235121	141235321
chr3	141240121	141240241
chr3	141244717	141244837
chr3	141248496	141248696
chr3	141259332	141259492
chr3	141272144	141272264
chr3	141272640	141272840
chr3	141274637	141274797
chr3	141277577	141277845
chr3	141278686	141278886
chr3	141289711	141289951
chr3	141290201	141290441
chr3	141291407	141291607
chr3	141291945	141292105
chr3	141292727	141292967
chr3	141294989	141295109
chr3	141295794	141295994
chr3	141299151	141299351
chr3	141299900	141300060
chr3	141304823	141304983
chr3	141305442	141305642

chr3	141309243	141309363
chr3	141324495	141324615
chr3	141326460	141326660
chr3	141327314	141327554
chr3	141328213	141328413
chr3	141328673	141328953
chr3	141331039	141331239
chr3	142164675	142164795
chr3	142168217	142168497
chr3	142169309	142169509
chr3	142171846	142172126
chr3	142176381	142176661
chr3	142177736	142178289
chr3	142180716	142180996
chr3	142183902	142184182
chr3	142185150	142185390
chr3	142186722	142186962
chr3	142188154	142188434
chr3	142188876	142189076
chr3	142203912	142204192
chr3	142211923	142212203
chr3	142215160	142215440
chr3	142215804	142216084
chr3	142217387	142217667
chr3	142218414	142218614
chr3	142222118	142222318
chr3	142223922	142224202
chr3	142226721	142227001
chr3	142231086	142231326
chr3	142232291	142232531
chr3	142234176	142234416
chr3	142238481	142238721
chr3	142241506	142241746
chr3	142242817	142243057
chr3	142253864	142254104
chr3	142254896	142255096
chr3	142257255	142257535
chr3	142259709	142259949
chr3	142261452	142261652
chr3	142266539	142266779
chr3	142268299	142268539
chr3	142268918	142269198
chr3	142272008	142272288

chr3	142272411	142272881
chr3	142274689	142275009
chr3	142275200	142275440
chr3	142277413	142277693
chr3	142278067	142278307
chr3	142279080	142279320
chr3	142280033	142280313
chr3	142281052	142281972
chr3	142284892	142285172
chr3	142286850	142287050
chr3	142297436	142297596
chr3	145168818	145168938
chr3	148171188	148171308
chr3	151187230	151187350
chr3	154454469	154454589
chr3	157475041	157475161
chr3	160519658	160519778
chr3	160534164	160534284
chr3	163525663	163525783
chr3	166585119	166585239
chr3	168802643	168802883
chr3	168804244	168804444
chr3	168806739	168807019
chr3	168807785	168808025
chr3	168810697	168810937
chr3	168812808	168813088
chr3	168815038	168815158
chr3	168818631	168818791
chr3	168819790	168820070
chr3	168825626	168825826
chr3	168830518	168830718
chr3	168833168	168834528
chr3	168838801	168839041
chr3	168840321	168840561
chr3	168845619	168845859
chr3	168849167	168849367
chr3	168861432	168861672
chr3	168862685	168862885
chr3	168954713	168954833
chr3	169017410	169017530
chr3	169033421	169033541
chr3	169079132	169079252
chr3	169098963	169099323

chr3	169128213	169128333
chr3	169146436	169146556
chr3	169177863	169177983
chr3	169191979	169192099
chr3	169197963	169198083
chr3	169243963	169244083
chr3	169249684	169249804
chr3	169283712	169283872
chr3	169322879	169322999
chr3	169330583	169330703
chr3	169347771	169347891
chr3	169366475	169366595
chr3	169381041	169381241
chr3	169595425	169595545
chr3	170781611	170781811
chr3	170783925	170784165
chr3	170784324	170784564
chr3	170786591	170786831
chr3	170788961	170789161
chr3	170797253	170797493
chr3	170799991	170800231
chr3	170801888	170802168
chr3	170802862	170803062
chr3	170805072	170805352
chr3	170811572	170811812
chr3	170819189	170819469
chr3	170824883	170825083
chr3	170825759	170825999
chr3	170828444	170828724
chr3	170841337	170841537
chr3	170843700	170843940
chr3	170846444	170846724
chr3	170855933	170856213
chr3	170857201	170857401
chr3	170858142	170858342
chr3	170875230	170875470
chr3	170879011	170879171
chr3	170884831	170885111
chr3	170892999	170893159
chr3	170895061	170895221
chr3	170906437	170906677
chr3	170908430	170908630
chr3	170912268	170912468

chr3	170928847	170929087
chr3	170943273	170943473
chr3	170945901	170946061
chr3	171064899	171065099
chr3	171087361	171087521
chr3	171177743	171177903
chr3	172603622	172603742
chr3	175632041	175632161
chr3	178794737	178794857
chr3	178916610	178916970
chr3	178917462	178917702
chr3	178919062	178919342
chr3	178921314	178921594
chr3	178922233	178922433
chr3	178927335	178927535
chr3	178927929	178928405
chr3	178935939	178936179
chr3	178936924	178937124
chr3	178937300	178937580
chr3	178937688	178937888
chr3	178938719	178938999
chr3	178941821	178942021
chr3	178942428	178942668
chr3	178943708	178943868
chr3	178947004	178947284
chr3	178947750	178947950
chr3	178947968	178948208
chr3	178951876	178952156
chr3	181801076	181801196
chr3	183818355	183818475
chr3	184844647	184844767
chr3	187440177	187440457
chr3	187442677	187442917
chr3	187443265	187443505
chr3	187444462	187444742
chr3	187446119	187446359
chr3	187446823	187447823
chr3	187449467	187449747
chr3	187451260	187451540
chr3	187859511	187859631
chr3	190877051	190877171
chr3	193120426	193120666
chr3	193125041	193125241

chr3	193128714	193128914
chr3	193129943	193130183
chr3	193132313	193132593
chr3	193151569	193151769
chr3	193153384	193153584
chr3	193156198	193156438
chr3	193156752	193156912
chr3	193158284	193158484
chr3	193159195	193159435
chr3	193160123	193160403
chr3	193165936	193166176
chr3	193171825	193172065
chr3	193174725	193175005
chr3	193175110	193175310
chr3	193176796	193177036
chr3	193180472	193180672
chr3	193182702	193182942
chr3	193183752	193184032
chr3	193185049	193185329
chr3	193188594	193188834
chr3	193201659	193201859
chr3	193207465	193207705
chr3	193209052	193209252
chr3	193210645	193211013
chr3	193220214	193220494
chr3	193232433	193232713
chr3	193272478	193272638
chr3	193879455	193879575
chr3	196898549	196898669
chr4	60097	60217
chr4	1791957	1792077
chr4	1793215	1793335
chr4	1795607	1795823
chr4	1796119	1796239
chr4	1797423	1797543
chr4	1797675	1797915
chr4	1798295	1798415
chr4	1799429	1799549
chr4	1800971	1801259
chr4	1801422	1801590
chr4	1802217	1802337
chr4	1803034	1803883
chr4	1803909	1804029

chr4	1804205	1804445
chr4	1804583	1804847
chr4	1805358	1805622
chr4	1806007	1806295
chr4	1806489	1806753
chr4	1807022	1807715
chr4	1807718	1808102
chr4	1808221	1808461
chr4	1808500	1808716
chr4	1808784	1809072
chr4	1816629	1816749
chr4	1902379	1902979
chr4	1904389	1904509
chr4	1905778	1906211
chr4	1907020	1907140
chr4	1913768	1913928
chr4	1918540	1918820
chr4	1918898	1919018
chr4	1919848	1920368
chr4	1925304	1925424
chr4	1925431	1925551
chr4	1932304	1932635
chr4	1936829	1937029
chr4	1940118	1940318
chr4	1941327	1941567
chr4	1944016	1944176
chr4	1946063	1946183
chr4	1947263	1947383
chr4	1952744	1952984
chr4	1953776	1954016
chr4	1954860	1955260
chr4	1956837	1957117
chr4	1957377	1957617
chr4	1957692	1957932
chr4	1959611	1959811
chr4	1961076	1961472
chr4	1962574	1962694
chr4	1962719	1962919
chr4	1963036	1963156
chr4	1971624	1971744
chr4	1976540	1976780
chr4	1976973	1977173
chr4	1978183	1978423

chr4	1978848	1978968
chr4	1979899	1980019
chr4	1980360	1980640
chr4	3089199	3089319
chr4	6090431	6090551
chr4	9599730	9599850
chr4	12664029	12664149
chr4	15712943	15713063
chr4	18722345	18722465
chr4	21726801	21726921
chr4	24779446	24779566
chr4	27790885	27791005
chr4	30796047	30796167
chr4	33879802	33879922
chr4	36979711	36979831
chr4	38776430	38776550
chr4	40187477	40187597
chr4	43199130	43199250
chr4	46238677	46238797
chr4	52728264	52728384
chr4	53219059	53219179
chr4	55743591	55743711
chr4	55946098	55946338
chr4	55948065	55948265
chr4	55948652	55948852
chr4	55953729	55953969
chr4	55954987	55955187
chr4	55955490	55955690
chr4	55955813	55956013
chr4	55956063	55956303
chr4	55958732	55958932
chr4	55960925	55961165
chr4	55961687	55961887
chr4	55962352	55962552
chr4	55963780	55963980
chr4	55964251	55964491
chr4	55964816	55965016
chr4	55968009	55968249
chr4	55968481	55968721
chr4	55970800	55971160
chr4	55971952	55972152
chr4	55972795	55973035
chr4	55973861	55974101

chr4	55976511	55976977
chr4	55979419	55979699
chr4	55980242	55980482
chr4	55980984	55981264
chr4	55981392	55981632
chr4	55984728	55985008
chr4	55987210	55987410
chr4	55991346	55991506
chr4	58744530	58744650
chr4	60833697	60833817
chr4	61771371	61771491
chr4	64948693	64948813
chr4	67961236	67961356
chr4	70974255	70974375
chr4	74208139	74208259
chr4	77228664	77228784
chr4	78269972	78270092
chr4	80352572	80352692
chr4	83378812	83378932
chr4	86384269	86384389
chr4	89389580	89389700
chr4	92454871	92454991
chr4	95472570	95472690
chr4	98567906	98568026
chr4	101579912	101580032
chr4	103431997	103432197
chr4	103443508	103443628
chr4	103446054	103446174
chr4	103446611	103446771
chr4	103450106	103450226
chr4	103450316	103450436
chr4	103450951	103451111
chr4	103454941	103455101
chr4	103457357	103457477
chr4	103458816	103458936
chr4	103458963	103459163
chr4	103465551	103465671
chr4	103481290	103481410
chr4	103481500	103481620
chr4	103486155	103486275
chr4	103488097	103488337
chr4	103497974	103498254
chr4	103499962	103500242

chr4	103501643	103501843
chr4	103503763	103503883
chr4	103503968	103504168
chr4	103505787	103506027
chr4	103506383	103506503
chr4	103510450	103510570
chr4	103511053	103511173
chr4	103514533	103514773
chr4	103515993	103516193
chr4	103517251	103517531
chr4	103518244	103518364
chr4	103518627	103518867
chr4	103522008	103522208
chr4	103527633	103527873
chr4	103528251	103528531
chr4	103528756	103528956
chr4	103531673	103531913
chr4	103533153	103533313
chr4	103533536	103533816
chr4	103534539	103534779
chr4	103537530	103537810
chr4	104640875	104640995
chr4	106111534	106111734
chr4	106155025	106158625
chr4	106162442	106162642
chr4	106163937	106164137
chr4	106164710	106164950
chr4	106180710	106180990
chr4	106182860	106183060
chr4	106190715	106190955
chr4	106193697	106194097
chr4	106196051	106196251
chr4	106196299	106197699
chr4	107685716	107685836
chr4	110708457	110708577
chr4	113715727	113715847
chr4	116804585	116804705
chr4	119817562	119817682
chr4	122824531	122824651
chr4	125840490	125840610
chr4	126237553	126242753
chr4	126272991	126273111
chr4	126279945	126280065

chr4	126281069	126281189
chr4	126281862	126281982
chr4	126286253	126286373
chr4	126286382	126286502
chr4	126315044	126315204
chr4	126319884	126320124
chr4	126328025	126328305
chr4	126329593	126329953
chr4	126333336	126333456
chr4	126336019	126336979
chr4	126337549	126337829
chr4	126352421	126352541
chr4	126355349	126355629
chr4	126367435	126367715
chr4	126369610	126373970
chr4	126378958	126379078
chr4	126380306	126380426
chr4	126384669	126384869
chr4	126387048	126387168
chr4	126389660	126389980
chr4	126397268	126397468
chr4	126398308	126398548
chr4	126400837	126401077
chr4	126402664	126402904
chr4	126408490	126408770
chr4	126411049	126412929
chr4	129024213	129024333
chr4	132052337	132052457
chr4	135137900	135138020
chr4	138161304	138161424
chr4	138313505	138313625
chr4	141194264	141194384
chr4	144220518	144220638
chr4	147260416	147260536
chr4	150280915	150281035
chr4	153244006	153244326
chr4	153245320	153245560
chr4	153247130	153247410
chr4	153249330	153249570
chr4	153250760	153251000
chr4	153251831	153252071
chr4	153253679	153253919
chr4	153258900	153259140

chr4	153268006	153268246
chr4	153269773	153269933
chr4	153271134	153271334
chr4	153273591	153273911
chr4	153303273	153303553
chr4	153332320	153332976
chr4	156352006	156352126
chr4	159430641	159430761
chr4	162460503	162460623
chr4	165498142	165498262
chr4	168545306	168545426
chr4	171592624	171592744
chr4	174604044	174604164
chr4	175896647	175899167
chr4	177605636	177605756
chr4	180653657	180653777
chr4	183655690	183655810
chr4	186658034	186658154
chr4	187509739	187510379
chr4	187511439	187511639
chr4	187511654	187511894
chr4	187513524	187513644
chr4	187513796	187513956
chr4	187516791	187517031
chr4	187517689	187518329
chr4	187518790	187518990
chr4	187519082	187519322
chr4	187520976	187521522
chr4	187522381	187522621
chr4	187524002	187524242
chr4	187524310	187525150
chr4	187525489	187525769
chr4	187527175	187527415
chr4	187527757	187527877
chr4	187530285	187530525
chr4	187530941	187531181
chr4	187532534	187532934
chr4	187534259	187534499
chr4	187535301	187535541
chr4	187538116	187538396
chr4	187538855	187542935
chr4	187549292	187549532
chr4	187549639	187549919

chr4	187554787	187555027
chr4	187557163	187557403
chr4	187557723	187558083
chr4	187560826	187560986
chr4	187574337	187574457
chr4	187580645	187580765
chr4	187584449	187584769
chr4	187593898	187594018
chr4	187627099	187627219
chr4	187627708	187630988
chr4	189687337	189687457
chr5	38079	38199
chr5	1295091	1295371
chr5	3042730	3042850
chr5	6043584	6043704
chr5	8828322	8828442
chr5	9044351	9044471
chr5	12060610	12060730
chr5	13692048	13692288
chr5	13700744	13700984
chr5	13701348	13701588
chr5	13708217	13708457
chr5	13714501	13714741
chr5	13716577	13716817
chr5	13717406	13717646
chr5	13718980	13719220
chr5	13721091	13721371
chr5	13727570	13727810
chr5	13729488	13729728
chr5	13735194	13735474
chr5	13735883	13736083
chr5	13737342	13737622
chr5	13751137	13751417
chr5	13752200	13752440
chr5	13753339	13753659
chr5	13754259	13754499
chr5	13758903	13759143
chr5	13762780	13763060
chr5	13766066	13766306
chr5	13769016	13769296
chr5	13769566	13769766
chr5	13770853	13771093
chr5	13776541	13776821

chr5	13777267	13777507
chr5	13780882	13781122
chr5	13786233	13786513
chr5	13788783	13789063
chr5	13792094	13792334
chr5	13793610	13793850
chr5	13793985	13794225
chr5	13807646	13807886
chr5	13809103	13809343
chr5	13810148	13810388
chr5	13811703	13811983
chr5	13814694	13814974
chr5	13817609	13817849
chr5	13820411	13820651
chr5	13823325	13823525
chr5	13824254	13824494
chr5	13829575	13829855
chr5	13830088	13830368
chr5	13830654	13830934
chr5	13839410	13839690
chr5	13841006	13841246
chr5	13841786	13842026
chr5	13844903	13845143
chr5	13850702	13850982
chr5	13859517	13859757
chr5	13862616	13862896
chr5	13864485	13864765
chr5	13865775	13866015
chr5	13866279	13866439
chr5	13867871	13868111
chr5	13870873	13871113
chr5	13871653	13871893
chr5	13876739	13876979
chr5	13882780	13883247
chr5	13885097	13885337
chr5	13886015	13886295
chr5	13891037	13891277
chr5	13894704	13894984
chr5	13900297	13900537
chr5	13901341	13901701
chr5	13902104	13902304
chr5	13911448	13911648
chr5	13913839	13914079

chr5	13914569	13914809
chr5	13916410	13916610
chr5	13917208	13917408
chr5	13919232	13919512
chr5	13920537	13920777
chr5	13922206	13922446
chr5	13923328	13923608
chr5	13928144	13928344
chr5	13931165	13931405
chr5	13944438	13944598
chr5	15093345	15093465
chr5	16784812	16784932
chr5	18099706	18099826
chr5	21143180	21143300
chr5	23509096	23509256
chr5	23509520	23509760
chr5	23509982	23510182
chr5	23517934	23518094
chr5	23521089	23521329
chr5	23522363	23522563
chr5	23522718	23522998
chr5	23523353	23523513
chr5	23524399	23524679
chr5	23526331	23527891
chr5	24163049	24163169
chr5	27198973	27199093
chr5	30209664	30209784
chr5	33212399	33212519
chr5	36221688	36221808
chr5	39224910	39225030
chr5	42398405	42398525
chr5	45400330	45400450
chr5	49820858	49820978
chr5	52828323	52828443
chr5	55828518	55828638
chr5	56111381	56111901
chr5	56152361	56152641
chr5	56155521	56155761
chr5	56160540	56160780
chr5	56161104	56161344
chr5	56161589	56161869
chr5	56167677	56167917
chr5	56168408	56168608

chr5	56168621	56168861
chr5	56170837	56171157
chr5	56174747	56174987
chr5	56176483	56176683
chr5	56176884	56177124
chr5	56177377	56177857
chr5	56177868	56178708
chr5	56179289	56179569
chr5	56180431	56180711
chr5	56181700	56181940
chr5	56183135	56183415
chr5	56183998	56184238
chr5	56189292	56189572
chr5	58831812	58831932
chr5	61866499	61866619
chr5	64870627	64870747
chr5	65892464	65892864
chr5	66055493	66055733
chr5	66084439	66084679
chr5	66124590	66124790
chr5	66166562	66166722
chr5	66195694	66195894
chr5	66198435	66198595
chr5	66212728	66212928
chr5	66226477	66226677
chr5	66255018	66255178
chr5	66259474	66259674
chr5	66259770	66259970
chr5	66282212	66282372
chr5	66300767	66300967
chr5	66350175	66350375
chr5	66385944	66386104
chr5	66389888	66390088
chr5	66391359	66391559
chr5	66396221	66396461
chr5	66398240	66398481
chr5	66400178	66400418
chr5	66405876	66406076
chr5	66409855	66410135
chr5	66414463	66414623
chr5	66416787	66416987
chr5	66426021	66426261
chr5	66427589	66427829

chr5	66429287	66429527
chr5	66430293	66430573
chr5	66432341	66432541
chr5	66437883	66438123
chr5	66438204	66438404
chr5	66440440	66440680
chr5	66440969	66441209
chr5	66445196	66445436
chr5	66448459	66448699
chr5	66449226	66449466
chr5	66456231	66456471
chr5	66458385	66458665
chr5	66458966	66462886
chr5	67880625	67880745
chr5	70948594	70948714
chr5	73950700	73950820
chr5	76967087	76967207
chr5	79970363	79970483
chr5	82977783	82977903
chr5	85993907	85994027
chr5	88999146	88999266
chr5	90151528	90151648
chr5	92019946	92020066
chr5	95036943	95037063
chr5	98058879	98058999
chr5	101075541	101075661
chr5	104123300	104123420
chr5	107139603	107139723
chr5	110143108	110143228
chr5	112024015	112024135
chr5	112032615	112032735
chr5	112043356	112043636
chr5	112047659	112047779
chr5	112072733	112072853
chr5	112090534	112090774
chr5	112100495	112100615
chr5	112101964	112102164
chr5	112102866	112103106
chr5	112111305	112111505
chr5	112116425	112116665
chr5	112125359	112125479
chr5	112128091	112128291
chr5	112136900	112137100

chr5	112151140	112151340
chr5	112154651	112155051
chr5	112157540	112157740
chr5	112158371	112158491
chr5	112162754	112162994
chr5	112163544	112163744
chr5	112164490	112164730
chr5	112170634	112170874
chr5	112172159	112172279
chr5	112173236	112179836
chr5	112192277	112192437
chr5	112198138	112198298
chr5	112212265	112212385
chr5	112223290	112223410
chr5	112230271	112230391
chr5	112364884	112365004
chr5	113143418	113143538
chr5	116153217	116153337
chr5	119165628	119165748
chr5	122169485	122169605
chr5	125173064	125173184
chr5	128173279	128173399
chr5	129194203	129194323
chr5	131288408	131288528
chr5	134331861	134331981
chr5	137493468	137493588
chr5	137791407	137791527
chr5	137792697	137792817
chr5	137795016	137795136
chr5	137797305	137797534
chr5	137799169	137799289
chr5	137799963	137800083
chr5	137801443	137801763
chr5	137802343	137803787
chr5	137805497	137805737
chr5	137807900	137808020
chr5	137808362	137808482
chr5	137810872	137810992
chr5	140527693	140527813
chr5	143540658	143540778
chr5	146554371	146554491
chr5	149495297	149495537
chr5	149497156	149497436

chr5	149498242	149498482
chr5	149498979	149499179
chr5	149499510	149499750
chr5	149500391	149500631
chr5	149500705	149500945
chr5	149501382	149501662
chr5	149502544	149502824
chr5	149503747	149503987
chr5	149504221	149504461
chr5	149504953	149505193
chr5	149506029	149506229
chr5	149509305	149509545
chr5	149510043	149510283
chr5	149511510	149511750
chr5	149512288	149512528
chr5	149513095	149513375
chr5	149513387	149513627
chr5	149514248	149514608
chr5	149515099	149515459
chr5	149516510	149516670
chr5	149568033	149568153
chr5	150885111	150885671
chr5	150886703	150887183
chr5	150887864	150887984
chr5	150889538	150889778
chr5	150891706	150892186
chr5	150897133	150897373
chr5	150900821	150901661
chr5	150903331	150903451
chr5	150905287	150905567
chr5	150906746	150906986
chr5	150907505	150907745
chr5	150908725	150908965
chr5	150911140	150911540
chr5	150913533	150913653
chr5	150913966	150914206
chr5	150917310	150917550
chr5	150918008	150918128
chr5	150920085	150920365
chr5	150921831	150925911
chr5	150928840	150929080
chr5	150930131	150930451
chr5	150930977	150931217

chr5	150932722	150932962
chr5	150933918	150934238
chr5	150935853	150936013
chr5	150942038	150942199
chr5	150942278	150942398
chr5	150942882	150943202
chr5	150945222	150948502
chr5	152588619	152588739
chr5	155603298	155603418
chr5	158679519	158679639
chr5	161684338	161684458
chr5	164710370	164710490
chr5	167715179	167715299
chr5	170726738	170726858
chr5	173743057	173743177
chr5	176747965	176748085
chr5	179663317	179663597
chr5	179665267	179665467
chr5	179666855	179667055
chr5	179667972	179668212
chr5	179669543	179669783
chr5	179674333	179674573
chr5	179674591	179674751
chr5	179674790	179674990
chr5	179675915	179676195
chr5	179688651	179688891
chr5	179691729	179691889
chr5	179696224	179696464
chr5	179706775	179707055
chr5	179707380	179707620
chr5	179757410	179757530
chr6	215363	215483
chr6	393140	393380
chr6	394773	395053
chr6	395573	395693
chr6	395790	395990
chr6	397059	397349
chr6	397360	397600
chr6	397876	397996
chr6	398181	398301
chr6	398714	398981
chr6	399018	399138
chr6	401420	401780

chr6	402026	402146
chr6	403543	403663
chr6	403738	403858
chr6	404571	404691
chr6	404973	405173
chr6	405333	405573
chr6	405839	405959
chr6	406675	406875
chr6	407406	407646
chr6	3258293	3258413
chr6	6260689	6260809
chr6	9283252	9283372
chr6	12286778	12286898
chr6	15246712	15246872
chr6	15305082	15305202
chr6	15374295	15374535
chr6	15401134	15401294
chr6	15410405	15410645
chr6	15452181	15452461
chr6	15468720	15469000
chr6	15487535	15487775
chr6	15496361	15497401
chr6	15501128	15501648
chr6	15504676	15504876
chr6	15507325	15507525
chr6	15507531	15507691
chr6	15508527	15508727
chr6	15509149	15509389
chr6	15511479	15511679
chr6	15512389	15512669
chr6	15513090	15513330
chr6	15513421	15513701
chr6	15517345	15517545
chr6	15520250	15520530
chr6	18330721	18330841
chr6	21332400	21332520
chr6	24342311	24342431
chr6	26055995	26056675
chr6	26156608	26157288
chr6	27367918	27368038
chr6	30368575	30368695
chr6	31539707	31539827
chr6	31539985	31540225

chr6	31540487	31540620
chr6	31540723	31540843
chr6	31542247	31542367
chr6	31546665	31546785
chr6	31547362	31547482
chr6	31548472	31548952
chr6	31549291	31549451
chr6	31549533	31549693
chr6	31549973	31550253
chr6	31555069	31555189
chr6	31555331	31555451
chr6	31555596	31555716
chr6	31556513	31556633
chr6	32163210	32163930
chr6	32164049	32164249
chr6	32164655	32164895
chr6	32165063	32165383
chr6	32166146	32166566
chr6	32166692	32166932
chr6	32168555	32168835
chr6	32168885	32169285
chr6	32169834	32170394
chr6	32171502	32171702
chr6	32171899	32172179
chr6	32178480	32178760
chr6	32180207	32180447
chr6	32180544	32180744
chr6	32180870	32181070
chr6	32181420	32181660
chr6	32181839	32182079
chr6	32182962	32183202
chr6	32184662	32185086
chr6	32185728	32185928
chr6	32187325	32187605
chr6	32187863	32188103
chr6	32188179	32188419
chr6	32188473	32188713
chr6	32188748	32189108
chr6	32190275	32190595
chr6	32190722	32190922
chr6	32191588	32191748
chr6	33398560	33398680
chr6	36437352	36437472

chr6	39464652	39464772
chr6	41898035	41898155
chr6	41898503	41898623
chr6	41903576	41903856
chr6	41904237	41904542
chr6	41904932	41905343
chr6	41906600	41906720
chr6	41907794	41907914
chr6	41908095	41908335
chr6	41908633	41908793
chr6	41909148	41909428
chr6	41913717	41913837
chr6	41914435	41914555
chr6	41915082	41915202
chr6	41915221	41915341
chr6	41915359	41915578
chr6	41915963	41916083
chr6	41918026	41918266
chr6	42478064	42478184
chr6	45296008	45296248
chr6	45296339	45296579
chr6	45390303	45390703
chr6	45399557	45399797
chr6	45405635	45405835
chr6	45459624	45459904
chr6	45479888	45480203
chr6	45481099	45481299
chr6	45512906	45513066
chr6	45514562	45515042
chr6	45630835	45631475
chr6	47708370	47708490
chr6	48488021	48488141
chr6	51483878	51484318
chr6	51490968	51491088
chr6	51491754	51491954
chr6	51497321	51497561
chr6	51503600	51503800
chr6	51512772	51512972
chr6	51513830	51514070
chr6	51523738	51524778
chr6	51586700	51586900
chr6	51609141	51609381
chr6	51611462	51611742

chr6	51612583	51613463
chr6	51617954	51618194
chr6	51619538	51619778
chr6	51637443	51637643
chr6	51640562	51640762
chr6	51655982	51656222
chr6	51695602	51695842
chr6	51701154	51701314
chr6	51712530	51712810
chr6	51720639	51720919
chr6	51732643	51732923
chr6	51735249	51735489
chr6	51747837	51748077
chr6	51750617	51750817
chr6	51751886	51752086
chr6	51768339	51768579
chr6	51768731	51768891
chr6	51770955	51771195
chr6	51774036	51774316
chr6	51776555	51776795
chr6	51777148	51777388
chr6	51798893	51799133
chr6	51824625	51824865
chr6	51875061	51875301
chr6	51882197	51882437
chr6	51887550	51887790
chr6	51889355	51890995
chr6	51892580	51892740
chr6	51892911	51893191
chr6	51897775	51898015
chr6	51900333	51900573
chr6	51907654	51907934
chr6	51908375	51908575
chr6	51909704	51909944
chr6	51910753	51911033
chr6	51913233	51913473
chr6	51914903	51915143
chr6	51917821	51918101
chr6	51918779	51919019
chr6	51920335	51920575
chr6	51921440	51921832
chr6	51923119	51923399
chr6	51924682	51924882

chr6	51927267	51927507
chr6	51929700	51929900
chr6	51930724	51930924
chr6	51934209	51934369
chr6	51935123	51935323
chr6	51935755	51935915
chr6	51936869	51937029
chr6	51938219	51938379
chr6	51941022	51941182
chr6	51944651	51944851
chr6	51947144	51947384
chr6	51947934	51948094
chr6	51949625	51949785
chr6	54509240	54509360
chr6	57516973	57517093
chr6	61967444	61967564
chr6	65000878	65000998
chr6	68033817	68033937
chr6	71036990	71037110
chr6	74041364	74041484
chr6	74608842	74608962
chr6	77059801	77059921
chr6	80085827	80085947
chr6	83170837	83170957
chr6	86268979	86269099
chr6	89271718	89271838
chr6	89790603	89791163
chr6	89793453	89793933
chr6	92320940	92321060
chr6	95403590	95403710
chr6	98517212	98517332
chr6	101540815	101540935
chr6	104554621	104554741
chr6	106534369	106534529
chr6	106536059	106536339
chr6	106537293	106537413
chr6	106537649	106537889
chr6	106538219	106538339
chr6	106538634	106538754
chr6	106541969	106542089
chr6	106543449	106543649
chr6	106543884	106544004
chr6	106544499	106544619

chr6	106545057	106545177
chr6	106545973	106546093
chr6	106546492	106546652
chr6	106546864	106547064
chr6	106547160	106547440
chr6	106548070	106548190
chr6	106548669	106548789
chr6	106551342	106551462
chr6	106551965	106552085
chr6	106552693	106553813
chr6	106554189	106554429
chr6	106554773	106555373
chr6	107637426	107637546
chr6	110659125	110659245
chr6	113682672	113682792
chr6	114262156	114262316
chr6	114262820	114262980
chr6	114264336	114264536
chr6	114264559	114264759
chr6	114265388	114265628
chr6	114266459	114266699
chr6	114267101	114267381
chr6	114270076	114270477
chr6	114274371	114274651
chr6	114277096	114277376
chr6	114277719	114277919
chr6	114279751	114279991
chr6	114281016	114281256
chr6	114291928	114292128
chr6	114292133	114292373
chr6	116698871	116698991
chr6	117609649	117609969
chr6	117619714	117619834
chr6	117620078	117620198
chr6	117622078	117622358
chr6	117624707	117624827
chr6	117628790	117628910
chr6	117629903	117630143
chr6	117631223	117631463
chr6	117632131	117632331
chr6	117633786	117633906
chr6	117638250	117638490
chr6	117639302	117639462

chr6	117639856	117639976
chr6	117640078	117640318
chr6	117640971	117641251
chr6	117642369	117642609
chr6	117645436	117645636
chr6	117646711	117646831
chr6	117647341	117647621
chr6	117649872	117650072
chr6	117650450	117650650
chr6	117658278	117658558
chr6	117662245	117662525
chr6	117662558	117662798
chr6	117663514	117663754
chr6	117665203	117665443
chr6	117674102	117674382
chr6	117677774	117678094
chr6	117678949	117679189
chr6	117680952	117681192
chr6	117681456	117681616
chr6	117683756	117684036
chr6	117684907	117685027
chr6	117686174	117686414
chr6	117686683	117686963
chr6	117687225	117687465
chr6	117700171	117700371
chr6	117704435	117704715
chr6	117706794	117707074
chr6	117708006	117708206
chr6	117708929	117709209
chr6	117710500	117711020
chr6	117714335	117714535
chr6	117715276	117715556
chr6	117715719	117715959
chr6	117716138	117716258
chr6	117717308	117717468
chr6	117718058	117718362
chr6	117724250	117724490
chr6	117725396	117725636
chr6	117728057	117728177
chr6	117730694	117730854
chr6	117735594	117735794
chr6	117737370	117737530
chr6	117739566	117739726

chr6	117741434	117741554
chr6	117746637	117746877
chr6	119713686	119713806
chr6	122744949	122745069
chr6	125748324	125748444
chr6	128291318	128291478
chr6	128294114	128294354
chr6	128294750	128295030
chr6	128297744	128297984
chr6	128298013	128298253
chr6	128302207	128302487
chr6	128303930	128304170
chr6	128304334	128304574
chr6	128306822	128307062
chr6	128311871	128312071
chr6	128312387	128312587
chr6	128313735	128313935
chr6	128316302	128316502
chr6	128316537	128316737
chr6	128317989	128318189
chr6	128319816	128320096
chr6	128322246	128322446
chr6	128324259	128324459
chr6	128326168	128326448
chr6	128327085	128327285
chr6	128329106	128329306
chr6	128330223	128330463
chr6	128385820	128386020
chr6	128388660	128388940
chr6	128399910	128400110
chr6	128403562	128403802
chr6	128404814	128405014
chr6	128410825	128411145
chr6	128505563	128505883
chr6	128540013	128540293
chr6	128561137	128561337
chr6	128563606	128563806
chr6	128642913	128643473
chr6	128718651	128718891
chr6	128730367	128730567
chr6	128812835	128813145
chr6	128841353	128841553
chr6	131879221	131879341

chr6	134943400	134943520
chr6	137947928	137948048
chr6	141001360	141001480
chr6	144004921	144005041
chr6	147006785	147006905
chr6	150144941	150145061
chr6	153168682	153168802
chr6	156198885	156199005
chr6	157099054	157100614
chr6	157144301	157144421
chr6	157150317	157150597
chr6	157153609	157153729
chr6	157161519	157161639
chr6	157188831	157188951
chr6	157192666	157192866
chr6	157196699	157196819
chr6	157197741	157197861
chr6	157199783	157199903
chr6	157218841	157218961
chr6	157222462	157222702
chr6	157242609	157242729
chr6	157256554	157256754
chr6	157338267	157338387
chr6	157357030	157357150
chr6	157389848	157389968
chr6	157405700	157406057
chr6	157431550	157431750
chr6	157432599	157432719
chr6	157442479	157442599
chr6	157443638	157443758
chr6	157454111	157454391
chr6	157469741	157470101
chr6	157488126	157488366
chr6	157495096	157495296
chr6	157495939	157496179
chr6	157502087	157502327
chr6	157505361	157505681
chr6	157507473	157507753
chr6	157510724	157510964
chr6	157511117	157511397
chr6	157517253	157517493
chr6	157519892	157520092
chr6	157521830	157522630

chr6	157524944	157525184
chr6	157527282	157529042
chr6	159210066	159210186
chr6	159425646	159425766
chr6	160390317	160390517
chr6	160412165	160412405
chr6	160429983	160430223
chr6	160431667	160431867
chr6	160445549	160445789
chr6	160448161	160448401
chr6	160450514	160450754
chr6	160453523	160453803
chr6	160453901	160454181
chr6	160455382	160455622
chr6	160461533	160461813
chr6	160464109	160464389
chr6	160465477	160465757
chr6	160466725	160466965
chr6	160467463	160467743
chr6	160468139	160468419
chr6	160468761	160469001
chr6	160469350	160469630
chr6	160471454	160471734
chr6	160477386	160477626
chr6	160478985	160479225
chr6	160479913	160480153
chr6	160481523	160481803
chr6	160482466	160483012
chr6	160483507	160483707
chr6	160484434	160484674
chr6	160485377	160485617
chr6	160485784	160485984
chr6	160489228	160489468
chr6	160490874	160491114
chr6	160492884	160493124
chr6	160493736	160493976
chr6	160494232	160494512
chr6	160494777	160495017
chr6	160496813	160497093
chr6	160499173	160499453
chr6	160500595	160500835
chr6	160501093	160501373
chr6	160504959	160505239

chr6	160505974	160506214
chr6	160509001	160509241
chr6	160510071	160510351
chr6	160510921	160511161
chr6	160517443	160517683
chr6	160523486	160523766
chr6	160524712	160524912
chr6	160525690	160526130
chr6	161412853	161413212
chr6	161455265	161455505
chr6	161469257	161469417
chr6	161469629	161471029
chr6	161491620	161491900
chr6	161494406	161494686
chr6	161501851	161502131
chr6	161505470	161505710
chr6	161507360	161507757
chr6	161508692	161509012
chr6	161510288	161510568
chr6	161512351	161512631
chr6	161512988	161513228
chr6	161513948	161514148
chr6	161514743	161514943
chr6	161518059	161518259
chr6	161519209	161519369
chr6	161519371	161519531
chr6	161522853	161523093
chr6	161523653	161523853
chr6	161527521	161527761
chr6	161528865	161529065
chr6	161529712	161529952
chr6	161530726	161531006
chr6	161532808	161533048
chr6	161533632	161533872
chr6	161536104	161536384
chr6	161537811	161537971
chr6	161551808	161552008
chr6	162237889	162238009
chr6	165248508	165248628
chr6	165557489	165557609
chr6	168289043	168289163
chr7	46179	46299
chr7	3082132	3082252

chr7	6098241	6098361
chr7	9110918	9111038
chr7	12115664	12115784
chr7	13935461	13935741
chr7	13940293	13940533
chr7	13945999	13946279
chr7	13947349	13947549
chr7	13949190	13949390
chr7	13950797	13950997
chr7	13971110	13971390
chr7	13975306	13975546
chr7	13978686	13978926
chr7	14016998	14017158
chr7	14025670	14025870
chr7	14026203	14026363
chr7	14027654	14027854
chr7	14028247	14028447
chr7	14028574	14028734
chr7	15126093	15126213
chr7	18156491	18156611
chr7	21163295	21163415
chr7	21582858	21583218
chr7	21584575	21584815
chr7	21598377	21598657
chr7	21599175	21599455
chr7	21600638	21600838
chr7	21603789	21604029
chr7	21609681	21609921
chr7	21611367	21611647
chr7	21621480	21621680
chr7	21627630	21627870
chr7	21628071	21628311
chr7	21628783	21629063
chr7	21630487	21630687
chr7	21630798	21631198
chr7	21639390	21639750
chr7	21640280	21640560
chr7	21640572	21640852
chr7	21641004	21641244
chr7	21646001	21646201
chr7	21646207	21646407
chr7	21654690	21654930
chr7	21655775	21655975

chr7	21657195	21657435
chr7	21658658	21658898
chr7	21659514	21659754
chr7	21675480	21675720
chr7	21677174	21677374
chr7	21678499	21678739
chr7	21695404	21695644
chr7	21698412	21698652
chr7	21721109	21721399
chr7	21723341	21723621
chr7	21726674	21726914
chr7	21726952	21727192
chr7	21730340	21730540
chr7	21737641	21737881
chr7	21742273	21742473
chr7	21744008	21744288
chr7	21745036	21745196
chr7	21747264	21747504
chr7	21750125	21750365
chr7	21751283	21751523
chr7	21757347	21757587
chr7	21760288	21760528
chr7	21765375	21765655
chr7	21775239	21775479
chr7	21778261	21778541
chr7	21779139	21779339
chr7	21781544	21781784
chr7	21783996	21784276
chr7	21784444	21784724
chr7	21788138	21788418
chr7	21789237	21789477
chr7	21789790	21790030
chr7	21804986	21805266
chr7	21813380	21813620
chr7	21818528	21818768
chr7	21824001	21824201
chr7	21826193	21826433
chr7	21826969	21827249
chr7	21828809	21829009
chr7	21840703	21840943
chr7	21847443	21847723
chr7	21856082	21856322
chr7	21857775	21858015

chr7	21882143	21882383
chr7	21891139	21891299
chr7	21892026	21892306
chr7	21893882	21894122
chr7	21901415	21901695
chr7	21904093	21904333
chr7	21906044	21906324
chr7	21907430	21907670
chr7	21908425	21908665
chr7	21912885	21913125
chr7	21920275	21920555
chr7	21923868	21924068
chr7	21932023	21932303
chr7	21934191	21934659
chr7	21938909	21939109
chr7	21939547	21939787
chr7	21940608	21940888
chr7	24166243	24166363
chr7	24213295	24213415
chr7	27167286	27167406
chr7	27203220	27203460
chr7	27204486	27205086
chr7	30194998	30195118
chr7	33197172	33197292
chr7	36211087	36211207
chr7	39213848	39213968
chr7	42221245	42221365
chr7	45222570	45222690
chr7	45932608	45932728
chr7	48238245	48238365
chr7	50305803	50305923
chr7	50311745	50311865
chr7	50313292	50313412
chr7	50332136	50332256
chr7	50350207	50350327
chr7	50358597	50358757
chr7	50367173	50367413
chr7	50424001	50424121
chr7	50430811	50430931
chr7	50435687	50436087
chr7	50438660	50438780
chr7	50444200	50444520
chr7	50449025	50449145

chr7	50450181	50450461
chr7	50452492	50452612
chr7	50454985	50455225
chr7	50459373	50459613
chr7	50460036	50460156
chr7	50466244	50466364
chr7	50467590	50468350
chr7	50474163	50474283
chr7	50475583	50475703
chr7	50481708	50481828
chr7	50486249	50486369
chr7	50493669	50493789
chr7	50507618	50507738
chr7	51252659	51252779
chr7	54252930	54253050
chr7	55086914	55087114
chr7	55088354	55088474
chr7	55090318	55090438
chr7	55127723	55127843
chr7	55144095	55144215
chr7	55154320	55154440
chr7	55160477	55160597
chr7	55172707	55172827
chr7	55179339	55179459
chr7	55192195	55192315
chr7	55209576	55209696
chr7	55209934	55210174
chr7	55210949	55211229
chr7	55214245	55214485
chr7	55218940	55219100
chr7	55220197	55220397
chr7	55221654	55221894
chr7	55223480	55223680
chr7	55224168	55224593
chr7	55225300	55225500
chr7	55227791	55228071
chr7	55229137	55229377
chr7	55231370	55231570
chr7	55232931	55233171
chr7	55236118	55236318
chr7	55237998	55238238
chr7	55238786	55238986
chr7	55240485	55240866

chr7	55241554	55241794
chr7	55242363	55242668
chr7	55247527	55247647
chr7	55248938	55249218
chr7	55251532	55251652
chr7	55253364	55253484
chr7	55259369	55259609
chr7	55259702	55259822
chr7	55260416	55260576
chr7	55266362	55266602
chr7	55267957	55268157
chr7	55268824	55269104
chr7	55269371	55269531
chr7	55270193	55270473
chr7	55272929	55273329
chr7	57256068	57256188
chr7	61074134	61074254
chr7	64149950	64150070
chr7	67159821	67159941
chr7	70161161	70161281
chr7	73176434	73176554
chr7	76208153	76208273
chr7	79221647	79221767
chr7	82222745	82222865
chr7	82387840	82388080
chr7	82389907	82390147
chr7	82390621	82390861
chr7	82430790	82430950
chr7	82434954	82435194
chr7	82451779	82452019
chr7	82453501	82453781
chr7	82455860	82456020
chr7	82457135	82457335
chr7	82464895	82465095
chr7	82467475	82467715
chr7	82470719	82470879
chr7	82474573	82474813
chr7	82475836	82475996
chr7	82476408	82476608
chr7	82478130	82478330
chr7	82497403	82497563
chr7	82508595	82508835
chr7	82531911	82532111

chr7	82538140	82538380
chr7	82543798	82546198
chr7	82578778	82580818
chr7	82581171	82586251
chr7	82595084	82595804
chr7	82763548	82764988
chr7	82784045	82785725
chr7	82791644	82791924
chr7	85280011	85280131
chr7	88294941	88295061
chr7	90773135	90773255
chr7	91440299	91440419
chr7	92244406	92244646
chr7	92247333	92247573
chr7	92252294	92252454
chr7	92300694	92300894
chr7	92354883	92355163
chr7	92403957	92404197
chr7	92462400	92462640
chr7	94463828	94463948
chr7	97464749	97464869
chr7	98627403	98627523
chr7	98628156	98628356
chr7	98630601	98630801
chr7	98633118	98633358
chr7	98633370	98633490
chr7	98634629	98634869
chr7	98635959	98636199
chr7	98637949	98638229
chr7	98639396	98639516
chr7	98639677	98639917
chr7	98643252	98643492
chr7	98645285	98645577
chr7	98647118	98647398
chr7	98648009	98648129
chr7	98648476	98648676
chr7	98648921	98649121
chr7	98649808	98650088
chr7	98652350	98652550
chr7	98653966	98654086
chr7	98654760	98654960
chr7	98654987	98655227
chr7	98658155	98658395

chr7	98659360	98659520
chr7	98676707	98676827
chr7	98690625	98690745
chr7	98697735	98697855
chr7	98731952	98732072
chr7	98739171	98739291
chr7	100468043	100468163
chr7	103488968	103489088
chr7	104681354	104681514
chr7	104682995	104683115
chr7	104685111	104685231
chr7	104687121	104687241
chr7	104688392	104688512
chr7	104696127	104696247
chr7	104700194	104700314
chr7	104702567	104702767
chr7	104703792	104704032
chr7	104704383	104704583
chr7	104707113	104707313
chr7	104710087	104710207
chr7	104714014	104714174
chr7	104715035	104715315
chr7	104716408	104716608
chr7	104717404	104717644
chr7	104717694	104717934
chr7	104719251	104719451
chr7	104722089	104722289
chr7	104729254	104729374
chr7	104730447	104730727
chr7	104731645	104731885
chr7	104737551	104737671
chr7	104741813	104742093
chr7	104742326	104742646
chr7	104745123	104745343
chr7	104745872	104746152
chr7	104746257	104746497
chr7	104746953	104747233
chr7	104747560	104747720
chr7	104747790	104748390
chr7	104748909	104749109
chr7	104749371	104749691
chr7	104750174	104750294
chr7	104750670	104751106

chr7	104751166	104751366
chr7	104752265	104753785
chr7	106500376	106500496
chr7	109568287	109568407
chr7	112579860	112579980
chr7	115584508	115584628
chr7	118634525	118634645
chr7	121513502	121513662
chr7	121568162	121568322
chr7	121607954	121608234
chr7	121612550	121612790
chr7	121616174	121616374
chr7	121616791	121616951
chr7	121623677	121623917
chr7	121623975	121624215
chr7	121636387	121636667
chr7	121637876	121638116
chr7	121644610	121644770
chr7	121650385	121653945
chr7	121659129	121659369
chr7	121668551	121668751
chr7	121671470	121671670
chr7	121674058	121674258
chr7	121674283	121674483
chr7	121676594	121676794
chr7	121678755	121678995
chr7	121679454	121679694
chr7	121680811	121681091
chr7	121682609	121682849
chr7	121684428	121684668
chr7	121691428	121691628
chr7	121691876	121692036
chr7	121693907	121694147
chr7	121694947	121695187
chr7	121698804	121699044
chr7	121699754	121699994
chr7	121701083	121701323
chr7	121890721	121890841
chr7	124462493	124462693
chr7	124463971	124464171
chr7	124465258	124465458
chr7	124467213	124467413
chr7	124469251	124469451

chr7	124475280	124475520
chr7	124481009	124481249
chr7	124482818	124483058
chr7	124486943	124487103
chr7	124491885	124492045
chr7	124492968	124493248
chr7	124498968	124499208
chr7	124503388	124503708
chr7	124510909	124511149
chr7	124532276	124532476
chr7	124537122	124537322
chr7	124893669	124893789
chr7	127953183	127953303
chr7	131071641	131071761
chr7	134083351	134083471
chr7	137098935	137099055
chr7	140108036	140108156
chr7	140426204	140426404
chr7	140432832	140432952
chr7	140434343	140434623
chr7	140439558	140439798
chr7	140447130	140447330
chr7	140449032	140449272
chr7	140453033	140453233
chr7	140453929	140454089
chr7	140455177	140455297
chr7	140456003	140456123
chr7	140473385	140473505
chr7	140476659	140476939
chr7	140477732	140477932
chr7	140481334	140481534
chr7	140482768	140483008
chr7	140487265	140487465
chr7	140494067	140494307
chr7	140499046	140499166
chr7	140500121	140500321
chr7	140501165	140501405
chr7	140505032	140505152
chr7	140505155	140505275
chr7	140506343	140506463
chr7	140507710	140507996
chr7	140508005	140508205
chr7	140508643	140508843

chr7	140511699	140511819
chr7	140534400	140534680
chr7	140549861	140550061
chr7	140558679	140558799
chr7	140567391	140567511
chr7	140614151	140614271
chr7	140617939	140618059
chr7	140624314	140624554
chr7	143134183	143134303
chr7	146168358	146168478
chr7	148504687	148504847
chr7	148506104	148506304
chr7	148506341	148506541
chr7	148507365	148507565
chr7	148508664	148508864
chr7	148510999	148511279
chr7	148511948	148512188
chr7	148512537	148512697
chr7	148513722	148513922
chr7	148514258	148514538
chr7	148514948	148515228
chr7	148516040	148516240
chr7	148516633	148516833
chr7	148523494	148523774
chr7	148524206	148524406
chr7	148525781	148526021
chr7	148526759	148526999
chr7	148529683	148529883
chr7	148533862	148534142
chr7	148543505	148543745
chr7	148544231	148544431
chr7	149190967	149191087
chr7	151833862	151834062
chr7	151835808	151836048
chr7	151836227	151836387
chr7	151836717	151836917
chr7	151841318	151841438
chr7	151841741	151842021
chr7	151842188	151842428
chr7	151842502	151842622
chr7	151842904	151843024
chr7	151843631	151843871
chr7	151845117	151846237

chr7	151847894	151848138
chr7	151848476	151848716
chr7	151849774	151850054
chr7	151850370	151850490
chr7	151851042	151851282
chr7	151851300	151851580
chr7	151852948	151853188
chr7	151853240	151853480
chr7	151854787	151855067
chr7	151855929	151856169
chr7	151859196	151860916
chr7	151864226	151864466
chr7	151866222	151866382
chr7	151868307	151868467
chr7	151871171	151871371
chr7	151873265	151875105
chr7	151876904	151877224
chr7	151877777	151879697
chr7	151880009	151880289
chr7	151882599	151882759
chr7	151884333	151884573
chr7	151884745	151884985
chr7	151891053	151891429
chr7	151891468	151891708
chr7	151892943	151893143
chr7	151893619	151893739
chr7	151896313	151896593
chr7	151899963	151900203
chr7	151902150	151902350
chr7	151904328	151904568
chr7	151917593	151917833
chr7	151919038	151919198
chr7	151919612	151919812
chr7	151921041	151921321
chr7	151921470	151921750
chr7	151926959	151927159
chr7	151927255	151927455
chr7	151932859	151933059
chr7	151935751	151935951
chr7	151944985	151945705
chr7	151946919	151947079
chr7	151947894	151948094
chr7	151948979	151949219

chr7	151949575	151949855
chr7	151955236	151955356
chr7	151960057	151960257
chr7	151962068	151962348
chr7	151965673	151965793
chr7	151970730	151971010
chr7	151981970	151982090
chr7	152007005	152007205
chr7	152008836	152009076
chr7	152009586	152009706
chr7	152012202	152012442
chr7	152027636	152027876
chr7	152031547	152031667
chr7	152050479	152050599
chr7	152055615	152055815
chr7	152132650	152132930
chr7	152198901	152199021
chr7	155201733	155201853
chr7	158215587	158215707
chr8	191016	191136
chr8	3199374	3199494
chr8	6209365	6209485
chr8	9217314	9217434
chr8	12598072	12598192
chr8	12957414	12957534
chr8	15598638	15598758
chr8	18618704	18618824
chr8	21668181	21668301
chr8	23060195	23060315
chr8	24736547	24736667
chr8	27740006	27740126
chr8	30784011	30784131
chr8	31445435	31445555
chr8	33903399	33903519
chr8	36911247	36911367
chr8	38225289	38225409
chr8	38226217	38226337
chr8	38236830	38236950
chr8	38239948	38240068
chr8	38241570	38241690
chr8	38243773	38243893
chr8	38271093	38271608
chr8	38271618	38271858

chr8	38272011	38272442
chr8	38273350	38273590
chr8	38274758	38274998
chr8	38275328	38275568
chr8	38275678	38275958
chr8	38277031	38277271
chr8	38279246	38279526
chr8	38280477	38280757
chr8	38282001	38282241
chr8	38283581	38283821
chr8	38285384	38285664
chr8	38285772	38286114
chr8	38287172	38287492
chr8	38297756	38297956
chr8	38299359	38299479
chr8	38314822	38315102
chr8	38318518	38318718
chr8	38322286	38322406
chr8	38361319	38361439
chr8	38369771	38369891
chr8	39911590	39911710
chr8	47823176	47823296
chr8	50834280	50834400
chr8	53834434	53834554
chr8	56866720	56866840
chr8	59873576	59873696
chr8	62919631	62919751
chr8	65922265	65922385
chr8	68927532	68927652
chr8	71932026	71932146
chr8	74954820	74954940
chr8	75822790	75822910
chr8	77616298	77618938
chr8	77619751	77620311
chr8	77690419	77690699
chr8	77694341	77694541
chr8	77745507	77745707
chr8	77754828	77755068
chr8	77761177	77761417
chr8	77761727	77761967
chr8	77762419	77762659
chr8	77763099	77764579
chr8	77764626	77765186

chr8	77765336	77766496
chr8	77766510	77768678
chr8	77775250	77775450
chr8	77775464	77776824
chr8	77819796	77819916
chr8	77998599	77998719
chr8	81002953	81003073
chr8	84219787	84219907
chr8	87232451	87232571
chr8	90236933	90237053
chr8	93247302	93247422
chr8	96256607	96256727
chr8	99258127	99258247
chr8	102261631	102261751
chr8	105261665	105261785
chr8	108269977	108270097
chr8	111280516	111280636
chr8	114290765	114290885
chr8	117293824	117293944
chr8	120294237	120294357
chr8	123304544	123304664
chr8	126311205	126311325
chr8	128747661	128752301
chr8	128752454	128753694
chr8	129312681	129312801
chr8	130459351	130459471
chr8	131024973	131025093
chr8	132328118	132328238
chr8	135328522	135328642
chr8	138333909	138334029
chr8	141339218	141339338
chr8	144345681	144345801
chr9	212701	212821
chr9	3214756	3214876
chr9	6215676	6215796
chr9	8317827	8317987
chr9	8319778	8320018
chr9	8331538	8331778
chr9	8338864	8339104
chr9	8340285	8340525
chr9	8341038	8341318
chr9	8341675	8341995
chr9	8375892	8376132

chr9	8376566	8376766
chr9	8389179	8389459
chr9	8404478	8404718
chr9	8436540	8436740
chr9	8437138	8437298
chr9	8449664	8449904
chr9	8454486	8454686
chr9	8460178	8460630
chr9	8465450	8465690
chr9	8470939	8471139
chr9	8484108	8484388
chr9	8485175	8485375
chr9	8485755	8486355
chr9	8492820	8493020
chr9	8497154	8497354
chr9	8499603	8499883
chr9	8500746	8501066
chr9	8504212	8504452
chr9	8507247	8507487
chr9	8517838	8518438
chr9	8521271	8521551
chr9	8523418	8523618
chr9	8524684	8525105
chr9	8526535	8526735
chr9	8527248	8527448
chr9	8528378	8528538
chr9	8528539	8528819
chr9	8633267	8633507
chr9	8636651	8636891
chr9	8733731	8733891
chr9	9228047	9228167
chr9	12229019	12229139
chr9	15239973	15240093
chr9	18261259	18261379
chr9	21405303	21405423
chr9	21968152	21968296
chr9	21968674	21968818
chr9	21969677	21969845
chr9	21970880	21971216
chr9	21972359	21972479
chr9	21973361	21973481
chr9	21974173	21974293
chr9	21974470	21974830

chr9	21974956	21975076
chr9	21975080	21975200
chr9	21975634	21975754
chr9	21978382	21978502
chr9	21978917	21979037
chr9	21986162	21986282
chr9	21986788	21986908
chr9	21988835	21988955
chr9	21990396	21990516
chr9	21991407	21991527
chr9	21991691	21991811
chr9	21991862	21991982
chr9	21993903	21994023
chr9	21994127	21994463
chr9	24478990	24479110
chr9	26531631	26531751
chr9	27483899	27484019
chr9	30500192	30500312
chr9	32516803	32516923
chr9	33524715	33524835
chr9	34614620	34614740
chr9	34619336	34619456
chr9	34619560	34619680
chr9	34621404	34621604
chr9	34621961	34622225
chr9	34622294	34622574
chr9	34622642	34622882
chr9	34623277	34623397
chr9	34623406	34623777
chr9	34623812	34624092
chr9	34624111	34624231
chr9	34625348	34625468
chr9	34625694	34625854
chr9	34626758	34626878
chr9	34627104	34627224
chr9	34627692	34628012
chr9	34631098	34631218
chr9	36639382	36639502
chr9	40585735	40585855
chr9	44863571	44863691
chr9	71034143	71034263
chr9	74048565	74048685
chr9	77114214	77114334

chr9	78831354	78831474
chr9	80118181	80118301
chr9	83121551	83121671
chr9	86127479	86127599
chr9	87285649	87285889
chr9	87317010	87317398
chr9	87322692	87322892
chr9	87325488	87325768
chr9	87338435	87338675
chr9	87339084	87339324
chr9	87342541	87342901
chr9	87356744	87356904
chr9	87359817	87360057
chr9	87366850	87367050
chr9	87425395	87425555
chr9	87475898	87476058
chr9	87482131	87482371
chr9	87486637	87486797
chr9	87549021	87549261
chr9	87563322	87563602
chr9	87570174	87570454
chr9	87635059	87635339
chr9	87636139	87636379
chr9	89157006	89157126
chr9	92158165	92158285
chr9	95271801	95271921
chr9	98273731	98273851
chr9	101278481	101278601
chr9	104291752	104291872
chr9	107293961	107294081
chr9	110311479	110311599
chr9	113328998	113329118
chr9	116363086	116363206
chr9	119419265	119419385
chr9	122426349	122426469
chr9	125426494	125426614
chr9	128435066	128435186
chr9	131452907	131453027
chr9	134465334	134465454
chr9	137475070	137475190
chr9	137828589	137828709
chr9	139258495	139258695
chr9	139258703	139259052

chr9	139259550	139259710
chr9	139261194	139261354
chr9	139261608	139261768
chr9	139262044	139262516
chr9	139264144	139264384
chr9	139264697	139265203
chr9	139265284	139265604
chr9	139265724	139265964
chr9	139266298	139266578
chr9	139390522	139392010
chr9	139393291	139393769
chr9	139394995	139395307
chr9	139396150	139396592
chr9	139396711	139396951
chr9	139397575	139397839
chr9	139399124	139399556
chr9	139399759	139400335
chr9	139400926	139401142
chr9	139401164	139401428
chr9	139401702	139401942
chr9	139402354	139402892
chr9	139403266	139403578
chr9	139404178	139404418
chr9	139405048	139405312
chr9	139405555	139405771
chr9	139407421	139407637
chr9	139407784	139408048
chr9	139408901	139409213
chr9	139409688	139409904
chr9	139409931	139410171
chr9	139410381	139410597
chr9	139411672	139411888
chr9	139412152	139412440
chr9	139412534	139412798
chr9	139413039	139413279
chr9	139413835	139414075
chr9	139417290	139417650
chr9	139418167	139418431
chr9	139438418	139438610
chr9	139440123	139440291
chr9	139793146	139793426
chr9	139793784	139793904
chr9	139794004	139794164

chr9	139794860	139795140
chr9	139795443	139795563
chr9	139796358	139796478
chr9	139797875	139798105
chr9	139802462	139802742
chr9	139803897	139804017
chr9	139804189	139804488
chr9	139807597	139807717
chr9	139808516	139808744
chr9	139810534	139810654
chr9	139810949	139811109
chr9	139814666	139814986
chr9	139814992	139815112
chr9	139815438	139815718
chr9	139816819	139816939
chr9	139816949	139817104
chr9	139817302	139817422
chr9	139818257	139818497
chr9	139819923	139820043
chr9	139820123	139820363
chr9	140477752	140477872
chr10	158886	159006
chr10	3165256	3165376
chr10	6181649	6181769
chr10	9192510	9192630
chr10	12195802	12195922
chr10	15199693	15199813
chr10	18229506	18229626
chr10	21240472	21240592
chr10	24244496	24244616
chr10	27253248	27253368
chr10	30272971	30273091
chr10	33281882	33282002
chr10	36282950	36283070
chr10	42827891	42828011
chr10	43572601	43572951
chr10	43595878	43596198
chr10	43597773	43598093
chr10	43600380	43600660
chr10	43601801	43602041
chr10	43604458	43604698
chr10	43606643	43606923
chr10	43607489	43607729

chr10	43608235	43608475
chr10	43608943	43609183
chr10	43609915	43610195
chr10	43611965	43612245
chr10	43613754	43613994
chr10	43614965	43615205
chr10	43615469	43615709
chr10	43617328	43617528
chr10	43619067	43619307
chr10	43620280	43620480
chr10	43621972	43622252
chr10	43623498	43623778
chr10	45829705	45829825
chr10	49419948	49420068
chr10	52499820	52499940
chr10	55507127	55507247
chr10	58687350	58687470
chr10	61726810	61726930
chr10	63661378	63661578
chr10	63661904	63662184
chr10	63669804	63669924
chr10	63688667	63688787
chr10	63699834	63700174
chr10	63718418	63718538
chr10	63727126	63727246
chr10	63759844	63760084
chr10	63800969	63801089
chr10	63803411	63803531
chr10	63809140	63809340
chr10	63809466	63809586
chr10	63810602	63810802
chr10	63811906	63812026
chr10	63816856	63817096
chr10	63818947	63819107
chr10	63829407	63829607
chr10	63832218	63832338
chr10	63832802	63832922
chr10	63834792	63834912
chr10	63835631	63835751
chr10	63841069	63841189
chr10	63845419	63845699
chr10	63850604	63852804
chr10	64745529	64745649

chr10	67759898	67760018
chr10	70800563	70800683
chr10	73819941	73820061
chr10	76821038	76821158
chr10	79825953	79826073
chr10	82857917	82858037
chr10	83757138	83757258
chr10	85889445	85889565
chr10	89175576	89175696
chr10	89624185	89624345
chr10	89653625	89653923
chr10	89657093	89657213
chr10	89674060	89674180
chr10	89675529	89675649
chr10	89682772	89682892
chr10	89684041	89684201
chr10	89685211	89685371
chr10	89686353	89686581
chr10	89689260	89689380
chr10	89690744	89690904
chr10	89692768	89693008
chr10	89700628	89700748
chr10	89705368	89705488
chr10	89707204	89707324
chr10	89711825	89712065
chr10	89716046	89716166
chr10	89717552	89717832
chr10	89719386	89719506
chr10	89720642	89720966
chr10	89723267	89723495
chr10	89724996	89725276
chr10	92195768	92195888
chr10	95202272	95202392
chr10	95790786	95792026
chr10	95848832	95849152
chr10	95891913	95892233
chr10	95930914	95931274
chr10	95986995	95987275
chr10	95993799	95994079
chr10	95995654	95995894
chr10	96005680	96006400
chr10	96012043	96012283
chr10	96013885	96014125

chr10	96014587	96014867
chr10	96018497	96018958
chr10	96022229	96022509
chr10	96025334	96025772
chr10	96028619	96028859
chr10	96030180	96030420
chr10	96033257	96033537
chr10	96039483	96039723
chr10	96043487	96043727
chr10	96044543	96044783
chr10	96053210	96053450
chr10	96058120	96058440
chr10	96064180	96064460
chr10	96066154	96066474
chr10	96068276	96068516
chr10	96072954	96073194
chr10	96076288	96076528
chr10	96081596	96081876
chr10	96084094	96084334
chr10	96084622	96084862
chr10	96087660	96087820
chr10	98205359	98205479
chr10	101213220	101213340
chr10	104149753	104149873
chr10	104150058	104150178
chr10	104150938	104151058
chr10	104152724	104152844
chr10	104155626	104155826
chr10	104155950	104156305
chr10	104156322	104156970
chr10	104157011	104157211
chr10	104157242	104157482
chr10	104157650	104157889
chr10	104157911	104158330
chr10	104158438	104158678
chr10	104159029	104159269
chr10	104159284	104159524
chr10	104159635	104159755
chr10	104159793	104159993
chr10	104160021	104160261
chr10	104160356	104160636
chr10	104160654	104160854
chr10	104160892	104161132

chr10	104161160	104161320
chr10	104161447	104161727
chr10	104161760	104162190
chr10	104162709	104162829
chr10	104164149	104164269
chr10	104170143	104170263
chr10	104170780	104170900
chr10	104239040	104239160
chr10	106022728	106022848
chr10	106039124	106039244
chr10	107252402	107252522
chr10	110261366	110261486
chr10	113267565	113267685
chr10	116292501	116292621
chr10	117612187	117612307
chr10	119295869	119295989
chr10	122301978	122302098
chr10	125327169	125327289
chr10	128332951	128333071
chr10	131346404	131346524
chr10	134442972	134443092
chr11	203728	203848
chr11	526377	526497
chr11	528514	528634
chr11	529906	530026
chr11	531163	531283
chr11	531443	531563
chr11	532595	532795
chr11	532976	533096
chr11	533246	533406
chr11	533412	533652
chr11	533714	533994
chr11	534166	534366
chr11	538471	538591
chr11	538879	539093
chr11	539689	539809
chr11	540097	540217
chr11	544036	544156
chr11	3204945	3205065
chr11	6217092	6217212
chr11	9230394	9230514
chr11	10819315	10819475
chr11	10820479	10820719

chr11	10820745	10820985
chr11	10821080	10821320
chr11	10821625	10821905
chr11	10821966	10822206
chr11	10822229	10822429
chr11	10822451	10822691
chr11	10823164	10823364
chr11	10823535	10824031
chr11	10824525	10824899
chr11	10824981	10825181
chr11	10825401	10826019
chr11	10826410	10826610
chr11	10827403	10827643
chr11	10828319	10828479
chr11	10828741	10828901
chr11	12232056	12232176
chr11	15232638	15232758
chr11	17408569	17408689
chr11	18235352	18235472
chr11	21251140	21251260
chr11	24267162	24267282
chr11	27334949	27335069
chr11	30342482	30342602
chr11	33362806	33362926
chr11	36376543	36376663
chr11	36608094	36608214
chr11	36608432	36608594
chr11	36612029	36612149
chr11	36613044	36613164
chr11	36613746	36613866
chr11	36614126	36615726
chr11	36616806	36616926
chr11	36618184	36618540
chr11	36618614	36618734
chr11	36620031	36620151
chr11	36621534	36621654
chr11	36622142	36622262
chr11	36622410	36622530
chr11	36625543	36625663
chr11	39436638	39436758
chr11	42443839	42443959
chr11	45458807	45458927
chr11	48548489	48548609

chr11	55091208	55091328
chr11	58113681	58113801
chr11	58978182	58980342
chr11	60878601	60878721
chr11	61067549	61067749
chr11	61068222	61068462
chr11	61069659	61069899
chr11	61069998	61070278
chr11	61070452	61070692
chr11	61071281	61071561
chr11	61076401	61076601
chr11	61077209	61077489
chr11	61077708	61077948
chr11	61079191	61079608
chr11	61080954	61081194
chr11	61081267	61082008
chr11	61083690	61084101
chr11	61088997	61089237
chr11	61089705	61089945
chr11	61090424	61090624
chr11	61091389	61091669
chr11	61093031	61093231
chr11	61094187	61094427
chr11	61096805	61097085
chr11	61097367	61097607
chr11	61098948	61099228
chr11	61100309	61100509
chr11	61126798	61126918
chr11	62559866	62560222
chr11	62561680	62561960
chr11	62562362	62562522
chr11	62563313	62563473
chr11	62563495	62563695
chr11	62563707	62563867
chr11	62563885	62564085
chr11	62564602	62564903
chr11	62565928	62566128
chr11	62567735	62568015
chr11	62568519	62568719
chr11	62568744	62568944
chr11	62568988	62569148
chr11	62569150	62569350
chr11	62569365	62569565

chr11	62569590	62569790
chr11	62570847	62571087
chr11	62571216	62571496
chr11	62571497	62571737
chr11	62572714	62572914
chr11	64156525	64156645
chr11	67174537	67174657
chr11	69455827	69469267
chr11	70253329	70253449
chr11	70318958	70319558
chr11	70331403	70331643
chr11	70331651	70333851
chr11	70335362	70335522
chr11	70336266	70336546
chr11	70338385	70338625
chr11	70341856	70342056
chr11	70343003	70343203
chr11	70346606	70346958
chr11	70347957	70348117
chr11	70348250	70348450
chr11	70348848	70349088
chr11	70505874	70506114
chr11	70507646	70507926
chr11	70544733	70544933
chr11	70644478	70644718
chr11	70653051	70653331
chr11	70666447	70666807
chr11	70672503	70672703
chr11	70719049	70719289
chr11	70742540	70742740
chr11	70757027	70757324
chr11	70779175	70779295
chr11	70788689	70789045
chr11	70789098	70789258
chr11	70789950	70790150
chr11	70798788	70799028
chr11	70803410	70803690
chr11	70805518	70805798
chr11	70820920	70821160
chr11	70824274	70824474
chr11	70829856	70830096
chr11	70858148	70858388
chr11	73276840	73276960

chr11	76139786	76139906
chr11	76157917	76158117
chr11	76161329	76161449
chr11	76162851	76163051
chr11	76163091	76163211
chr11	76164296	76164496
chr11	76165733	76165893
chr11	76169174	76169454
chr11	76170912	76171192
chr11	76174854	76175134
chr11	76183585	76183984
chr11	76207245	76207525
chr11	76224364	76224644
chr11	76227130	76227410
chr11	76234146	76234386
chr11	76236160	76236280
chr11	76237452	76237732
chr11	76239290	76239530
chr11	76246880	76247160
chr11	76248776	76249056
chr11	76250583	76250743
chr11	76253191	76253431
chr11	76255284	76255884
chr11	76256810	76257370
chr11	76260972	76261212
chr11	76313721	76313841
chr11	79322425	79322545
chr11	82324005	82324125
chr11	85436639	85436759
chr11	88445210	88445330
chr11	91448217	91448337
chr11	92085264	92088584
chr11	92219170	92219290
chr11	92225137	92225257
chr11	92229937	92230057
chr11	92234472	92234592
chr11	92244091	92244211
chr11	92257796	92258116
chr11	92259495	92259615
chr11	92272589	92272709
chr11	92289673	92289793
chr11	92292534	92292774
chr11	92301589	92301709

chr11	92325324	92325444
chr11	92335100	92335220
chr11	92340902	92341022
chr11	92353581	92353701
chr11	92416841	92416961
chr11	92430500	92430660
chr11	92495018	92495338
chr11	92498029	92498269
chr11	92507156	92507396
chr11	92523106	92523386
chr11	92525917	92526157
chr11	92530998	92535078
chr11	92538276	92538556
chr11	92539484	92539724
chr11	92543005	92543245
chr11	92564782	92565182
chr11	92568022	92568262
chr11	92569680	92569920
chr11	92570780	92571020
chr11	92573686	92573966
chr11	92577098	92577898
chr11	92590326	92590566
chr11	92592289	92592529
chr11	92599900	92600380
chr11	92613853	92614093
chr11	92615891	92616571
chr11	92620122	92620322
chr11	92622304	92622464
chr11	92622939	92623139
chr11	92623647	92624287
chr11	94153238	94153398
chr11	94168931	94169131
chr11	94170291	94170451
chr11	94178917	94179117
chr11	94180374	94180614
chr11	94189372	94189572
chr11	94192520	94192800
chr11	94194031	94194271
chr11	94197221	94197461
chr11	94200918	94201118
chr11	94203582	94203862
chr11	94204712	94204952
chr11	94208681	94208881

chr11	94209391	94209631
chr11	94211831	94212111
chr11	94212783	94212983
chr11	94219029	94219309
chr11	94223944	94224184
chr11	94225741	94226057
chr11	94479301	94479421
chr11	97494247	97494367
chr11	100509469	100509589
chr11	101981559	101981919
chr11	101984859	101985139
chr11	102033124	102033364
chr11	102056688	102056928
chr11	102076600	102076840
chr11	102080191	102080351
chr11	102094297	102094537
chr11	102098135	102098375
chr11	102100411	102100691
chr11	102188780	102188934
chr11	102190108	102190228
chr11	102195226	102196106
chr11	102196146	102196346
chr11	102196376	102196517
chr11	102197400	102197640
chr11	102198470	102198590
chr11	102198741	102198901
chr11	102199571	102199731
chr11	102199983	102200219
chr11	102201710	102201990
chr11	102202790	102202910
chr11	102204943	102205063
chr11	102206683	102206963
chr11	102207431	102207591
chr11	102207596	102207910
chr11	102209676	102209796
chr11	102211360	102211480
chr11	102212819	102212939
chr11	102213984	102214214
chr11	102215342	102215462
chr11	102219341	102219541
chr11	102220572	102221492
chr11	102221524	102221724
chr11	102231664	102231784

chr11	102231861	102231981
chr11	102233585	102233745
chr11	102234342	102234502
chr11	102235080	102235200
chr11	102237757	102237877
chr11	102238092	102238212
chr11	102238332	102238452
chr11	102239017	102239297
chr11	102247615	102247735
chr11	102248213	102248493
chr11	102248528	102248957
chr11	102253609	102253729
chr11	103512149	103512269
chr11	106517823	106517943
chr11	108098291	108098678
chr11	108099837	108100117
chr11	108106338	108106618
chr11	108114659	108114939
chr11	108115493	108115773
chr11	108117632	108117912
chr11	108119617	108119897
chr11	108121413	108121813
chr11	108122540	108122780
chr11	108123491	108123691
chr11	108124513	108124793
chr11	108126907	108127147
chr11	108128118	108128358
chr11	108129657	108129857
chr11	108137843	108138123
chr11	108139116	108139356
chr11	108141731	108142195
chr11	108143196	108143633
chr11	108150194	108150434
chr11	108151668	108151948
chr11	108153381	108153661
chr11	108154936	108155216
chr11	108158295	108158535
chr11	108159646	108159886
chr11	108160308	108160548
chr11	108163292	108163572
chr11	108164019	108164299
chr11	108165599	108165839
chr11	108167961	108168161

chr11	108170386	108170666
chr11	108172305	108172585
chr11	108173527	108173807
chr11	108175350	108175630
chr11	108178567	108178767
chr11	108180824	108181104
chr11	108183081	108183281
chr11	108186493	108186908
chr11	108188033	108188313
chr11	108190612	108190852
chr11	108191967	108192207
chr11	108196013	108196293
chr11	108196728	108197008
chr11	108198308	108198548
chr11	108199736	108199976
chr11	108200924	108201164
chr11	108202089	108202329
chr11	108202544	108202824
chr11	108203437	108203677
chr11	108204553	108204753
chr11	108205625	108205905
chr11	108206509	108206749
chr11	108213883	108214163
chr11	108216412	108216692
chr11	108217948	108218148
chr11	108224429	108224669
chr11	108225469	108225669
chr11	108235756	108235996
chr11	108236023	108236263
chr11	109575046	109575166
chr11	112580517	112580637
chr11	115618027	115618147
chr11	118307223	118307663
chr11	118309687	118309887
chr11	118317877	118317997
chr11	118318118	118318238
chr11	118318290	118318450
chr11	118320492	118320612
chr11	118339444	118339604
chr11	118341588	118341708
chr11	118342363	118345043
chr11	118347468	118347748
chr11	118348679	118348919

chr11	118349278	118349398
chr11	118350840	118351000
chr11	118351024	118351144
chr11	118352418	118352818
chr11	118353093	118353253
chr11	118354843	118355083
chr11	118355533	118355733
chr11	118359100	118359220
chr11	118359281	118359521
chr11	118360454	118360654
chr11	118360783	118361023
chr11	118361851	118362091
chr11	118362410	118362690
chr11	118362808	118362928
chr11	118363396	118363516
chr11	118363718	118363998
chr11	118364957	118365157
chr11	118365202	118365322
chr11	118365365	118365525
chr11	118366371	118366651
chr11	118366928	118367128
chr11	118368599	118368839
chr11	118369043	118369283
chr11	118369976	118370176
chr11	118370508	118370668
chr11	118371641	118371921
chr11	118372339	118372619
chr11	118373096	118377376
chr11	118377462	118377582
chr11	118378183	118378383
chr11	118379802	118379962
chr11	118380607	118380887
chr11	118381008	118381128
chr11	118382622	118382855
chr11	118390279	118390559
chr11	118390625	118390825
chr11	118391458	118391658
chr11	118391947	118392187
chr11	118392609	118392889
chr11	118618987	118619107
chr11	119077084	119077364
chr11	119078932	119079052
chr11	119085722	119085842

chr11	119086568	119086688
chr11	119088822	119088942
chr11	119096731	119096851
chr11	119099767	119099887
chr11	119103141	119103421
chr11	119105631	119105751
chr11	119107678	119107798
chr11	119142397	119142637
chr11	119142736	119142856
chr11	119144535	119144775
chr11	119145213	119145333
chr11	119145482	119145722
chr11	119146655	119146895
chr11	119147113	119147233
chr11	119148410	119148610
chr11	119148821	119149061
chr11	119149201	119149441
chr11	119155624	119155864
chr11	119155887	119156287
chr11	119158508	119158708
chr11	119165986	119166106
chr11	119167585	119167785
chr11	119168042	119168242
chr11	119169018	119169298
chr11	119170187	119170507
chr11	121628947	121629067
chr11	122713749	122713869
chr11	124635084	124635204
chr11	127636462	127636582
chr11	130637827	130637947
chr11	133656831	133656951
chr12	197781	197901
chr12	394604	394844
chr12	395270	395430
chr12	401909	402349
chr12	404728	404968
chr12	406166	406406
chr12	416063	416303
chr12	416615	417175
chr12	418909	419189
chr12	420000	420280
chr12	422170	422410
chr12	427269	427629

chr12	430117	430317
chr12	431537	431777
chr12	432189	432429
chr12	432716	432996
chr12	437957	438237
chr12	440944	441144
chr12	442593	442873
chr12	443357	443637
chr12	459745	459985
chr12	461330	461530
chr12	463200	463440
chr12	464269	464469
chr12	465550	465750
chr12	472075	472315
chr12	475044	475324
chr12	493137	493377
chr12	495021	495181
chr12	496326	496526
chr12	497352	497552
chr12	498034	498314
chr12	998304	998424
chr12	3200904	3201024
chr12	4383163	4383443
chr12	4385158	4385398
chr12	4387885	4388125
chr12	4388210	4388330
chr12	4388471	4388591
chr12	4389097	4389217
chr12	4392469	4392589
chr12	4394816	4394936
chr12	4397705	4397825
chr12	4397961	4398201
chr12	4399856	4399976
chr12	4402756	4402876
chr12	4403749	4403989
chr12	4404315	4404435
chr12	4405743	4405863
chr12	4406220	4406340
chr12	4406775	4407159
chr12	4408612	4408732
chr12	4408980	4409220
chr12	5168788	5168908
chr12	5674693	5674813

chr12	6169137	6169257
chr12	6202799	6202919
chr12	6484640	6484840
chr12	6484974	6485094
chr12	6485600	6485720
chr12	6488389	6488509
chr12	6490123	6490243
chr12	6490320	6490440
chr12	6490548	6490668
chr12	6490682	6490802
chr12	6491856	6492096
chr12	6492588	6492708
chr12	6493472	6493672
chr12	6493701	6493960
chr12	6494130	6494588
chr12	6495179	6495379
chr12	6495461	6495661
chr12	6496034	6496154
chr12	6496913	6497113
chr12	6497198	6497318
chr12	6497517	6497717
chr12	6497878	6498078
chr12	6498594	6498714
chr12	6499271	6499511
chr12	6499824	6500104
chr12	6554209	6554449
chr12	6554535	6554775
chr12	6559288	6559568
chr12	6559651	6559851
chr12	6560000	6560240
chr12	6560375	6560615
chr12	6653983	6654103
chr12	7049938	7050058
chr12	7600688	7600808
chr12	8090778	8090898
chr12	8687751	8687871
chr12	9174895	9175015
chr12	9212086	9212206
chr12	9739824	9739944
chr12	10251062	10251182
chr12	10743855	10743975
chr12	11802997	11803157
chr12	11905328	11905568

chr12	11992015	11992295
chr12	12006307	12006547
chr12	12022330	12022930
chr12	12037309	12037589
chr12	12038789	12039029
chr12	12043807	12044047
chr12	12161480	12161600
chr12	12223782	12223902
chr12	14538225	14538425
chr12	14576824	14578424
chr12	14587213	14587413
chr12	14588972	14589252
chr12	14591012	14591252
chr12	14599854	14600054
chr12	14609431	14609631
chr12	14610084	14610284
chr12	14613407	14614087
chr12	14619391	14619591
chr12	14628762	14628962
chr12	14631188	14631468
chr12	14633908	14634188
chr12	14649100	14649340
chr12	14650577	14651017
chr12	15227282	15227402
chr12	18233007	18233127
chr12	18434994	18435714
chr12	18439721	18439921
chr12	18443727	18444007
chr12	18446771	18447011
chr12	18466826	18467066
chr12	18473830	18474030
chr12	18477900	18478100
chr12	18491300	18491540
chr12	18496197	18496357
chr12	18499552	18499792
chr12	18515640	18515880
chr12	18524059	18524299
chr12	18534636	18534876
chr12	18544000	18544240
chr12	18552566	18552806
chr12	18573821	18574021
chr12	18576825	18577025
chr12	18641352	18641592

chr12	18644318	18644558
chr12	18648941	18649141
chr12	18650497	18650737
chr12	18656162	18656402
chr12	18658170	18658450
chr12	18691027	18691307
chr12	18699191	18699431
chr12	18715611	18715851
chr12	18716251	18716491
chr12	18719816	18720056
chr12	18747365	18747565
chr12	18762415	18762615
chr12	18793305	18793545
chr12	18800745	18801025
chr12	21274430	21274550
chr12	24284669	24284789
chr12	25362686	25362886
chr12	25368334	25368534
chr12	25369642	25369762
chr12	25371401	25371521
chr12	25375738	25375858
chr12	25377965	25378085
chr12	25378507	25378747
chr12	25380116	25380396
chr12	25382125	25382245
chr12	25382481	25382601
chr12	25383752	25383872
chr12	25385381	25385501
chr12	25388049	25388249
chr12	25391003	25391123
chr12	25391178	25391298
chr12	25391395	25391515
chr12	25392232	25392352
chr12	25392382	25392502
chr12	25393804	25393924
chr12	25398162	25398362
chr12	27285846	27285966
chr12	30289890	30290010
chr12	31184292	31184412
chr12	31195212	31195332
chr12	31225650	31225770
chr12	31231259	31231539
chr12	31236730	31237010

chr12	31237459	31237659
chr12	31237841	31238121
chr12	31238869	31239252
chr12	31240814	31240974
chr12	31241911	31242151
chr12	31242280	31242480
chr12	31242803	31243043
chr12	31244588	31244868
chr12	31245722	31245882
chr12	31246118	31246318
chr12	31247465	31247822
chr12	31249213	31249373
chr12	31249504	31249978
chr12	31250754	31250994
chr12	31253504	31253704
chr12	31253892	31254132
chr12	31254701	31254981
chr12	31255110	31255530
chr12	31255811	31256011
chr12	31256189	31256389
chr12	31256447	31256993
chr12	31292360	31292480
chr12	33293847	33293967
chr12	38475405	38475525
chr12	40618888	40619128
chr12	40619299	40619499
chr12	40626030	40626230
chr12	40629371	40629571
chr12	40631717	40631957
chr12	40634231	40634471
chr12	40637297	40637537
chr12	40643587	40643787
chr12	40644984	40645224
chr12	40645226	40645386
chr12	40646664	40646864
chr12	40650994	40651234
chr12	40653223	40653463
chr12	40657546	40657746
chr12	40668336	40668576
chr12	40668605	40668845
chr12	40671633	40672117
chr12	40677665	40677945
chr12	40681106	40681386

chr12	40687305	40687505
chr12	40688601	40688761
chr12	40689217	40689457
chr12	40692029	40692309
chr12	40692864	40693104
chr12	40694555	40694755
chr12	40696537	40696737
chr12	40697742	40697982
chr12	40699537	40699817
chr12	40702263	40702503
chr12	40702851	40703091
chr12	40704221	40704461
chr12	40707754	40707994
chr12	40708957	40709157
chr12	40713743	40714023
chr12	40714792	40715032
chr12	40715789	40716029
chr12	40716076	40716356
chr12	40716914	40717154
chr12	40722111	40722311
chr12	40728723	40729003
chr12	40734035	40734315
chr12	40740499	40740779
chr12	40742160	40742360
chr12	40745297	40745577
chr12	40748057	40748337
chr12	40749872	40750032
chr12	40753013	40753293
chr12	40757159	40757399
chr12	40758627	40758867
chr12	40760763	40760923
chr12	40761386	40761626
chr12	41497798	41497918
chr12	44508200	44508320
chr12	46123565	46123765
chr12	46123773	46123973
chr12	46124948	46125148
chr12	46134760	46134880
chr12	46154998	46155118
chr12	46175408	46175528
chr12	46192296	46192416
chr12	46200334	46200454
chr12	46205147	46205387

chr12	46211441	46211681
chr12	46215102	46215316
chr12	46227589	46227709
chr12	46230324	46230484
chr12	46230508	46230788
chr12	46231051	46231251
chr12	46231265	46231505
chr12	46231892	46232012
chr12	46233055	46233335
chr12	46238951	46239071
chr12	46240579	46240779
chr12	46242565	46242805
chr12	46243320	46243600
chr12	46243808	46246688
chr12	46248739	46248859
chr12	46254537	46254777
chr12	46274797	46274917
chr12	46275876	46275996
chr12	46285511	46285936
chr12	46287144	46287560
chr12	46298033	46298233
chr12	46298668	46298908
chr12	47509175	47509295
chr12	48317772	48317892
chr12	48325783	48325903
chr12	48343978	48344098
chr12	48358219	48358339
chr12	48359924	48360044
chr12	48366389	48366509
chr12	48367122	48367402
chr12	48367852	48368132
chr12	48368431	48368671
chr12	48369083	48369403
chr12	48369679	48369919
chr12	48370243	48370403
chr12	48370528	48370768
chr12	48370831	48370991
chr12	48371036	48371276
chr12	48371329	48371489
chr12	48371535	48371655
chr12	48371726	48371966
chr12	48372007	48372247
chr12	48372320	48372600

chr12	48373240	48373400
chr12	48373738	48373898
chr12	48374270	48374804
chr12	48375072	48375232
chr12	48375505	48375665
chr12	48375836	48375996
chr12	48376218	48376458
chr12	48376579	48376779
chr12	48376817	48376977
chr12	48377114	48377274
chr12	48377412	48377572
chr12	48377816	48377976
chr12	48378275	48378435
chr12	48378726	48378926
chr12	48379263	48379423
chr12	48379459	48379801
chr12	48379971	48380292
chr12	48380564	48380724
chr12	48380809	48381153
chr12	48381342	48381542
chr12	48382957	48383117
chr12	48383485	48383645
chr12	48385516	48385636
chr12	48386607	48386767
chr12	48387182	48387342
chr12	48387538	48387698
chr12	48387723	48387883
chr12	48388153	48388313
chr12	48388984	48389144
chr12	48389439	48389759
chr12	48390269	48390469
chr12	48390779	48390899
chr12	48391217	48391417
chr12	48391420	48391580
chr12	48391600	48392047
chr12	48392105	48392305
chr12	48393684	48393924
chr12	48397857	48398159
chr12	48403169	48403289
chr12	48410457	48410577
chr12	48418496	48418616
chr12	48444428	48444548
chr12	48446245	48446365

chr12	49415508	49415708
chr12	49415779	49415979
chr12	49416019	49416179
chr12	49416355	49416675
chr12	49416883	49417003
chr12	49417779	49417939
chr12	49418305	49418780
chr12	49419616	49419736
chr12	49419954	49421114
chr12	49421529	49421977
chr12	49422033	49422153
chr12	49422555	49423071
chr12	49423141	49423301
chr12	49424022	49424262
chr12	49424327	49424865
chr12	49424952	49427752
chr12	49427845	49428085
chr12	49428145	49428305
chr12	49428306	49428506
chr12	49428536	49428776
chr12	49429995	49430115
chr12	49430899	49432779
chr12	49432952	49433448
chr12	49433492	49435332
chr12	49435382	49435542
chr12	49435656	49435816
chr12	49435852	49436132
chr12	49436285	49436783
chr12	49436813	49437013
chr12	49437098	49437258
chr12	49437371	49437835
chr12	49437934	49438134
chr12	49438145	49438345
chr12	49438517	49438757
chr12	49439431	49439567
chr12	49439598	49440264
chr12	49440342	49440622
chr12	49441699	49441899
chr12	49442396	49442596
chr12	49442844	49443044
chr12	49443458	49444578
chr12	49444657	49446217
chr12	49446299	49446539

chr12	49446656	49446896
chr12	49446946	49447146
chr12	49447201	49447481
chr12	49447701	49447981
chr12	49448044	49448244
chr12	49448302	49448542
chr12	49448625	49448865
chr12	49449002	49449162
chr12	50514418	50514538
chr12	53514972	53515092
chr12	54466034	54466154
chr12	56474025	56474225
chr12	56477470	56477750
chr12	56478757	56479117
chr12	56480257	56480497
chr12	56481293	56481493
chr12	56481517	56482015
chr12	56482263	56482711
chr12	56486467	56486667
chr12	56486714	56486914
chr12	56487111	56487351
chr12	56487493	56487733
chr12	56487827	56488027
chr12	56488122	56488402
chr12	56488987	56489147
chr12	56489379	56489659
chr12	56490226	56490466
chr12	56490480	56490680
chr12	56490801	56491041
chr12	56491506	56491786
chr12	56492221	56492421
chr12	56492460	56492740
chr12	56493380	56493580
chr12	56493597	56493837
chr12	56493893	56494093
chr12	56494814	56495174
chr12	56495246	56495855
chr12	56630007	56630127
chr12	58142253	58142453
chr12	58142912	58143152
chr12	58143181	58143341
chr12	58144204	58144593
chr12	58144649	58144929

chr12	58144937	58145177
chr12	58145271	58145511
chr12	59723217	59723337
chr12	62745262	62745382
chr12	65748972	65749092
chr12	68751345	68751465
chr12	71822626	71822746
chr12	74843694	74843814
chr12	77873194	77873314
chr12	80888510	80888630
chr12	83895380	83895500
chr12	86965703	86965823
chr12	90147374	90147494
chr12	93165559	93165679
chr12	96168345	96168465
chr12	99173683	99173803
chr12	102182121	102182241
chr12	103772716	103772836
chr12	105230659	105230779
chr12	108233514	108233634
chr12	109937473	109937593
chr12	110719533	110719773
chr12	110720317	110720658
chr12	110729756	110729996
chr12	110734352	110734592
chr12	110760736	110760936
chr12	110764137	110764337
chr12	110765329	110765849
chr12	110770345	110770545
chr12	110770916	110771156
chr12	110771762	110772002
chr12	110777026	110777266
chr12	110777296	110777536
chr12	110778451	110778811
chr12	110780002	110780282
chr12	110781017	110781257
chr12	110782633	110782833
chr12	110783000	110783240
chr12	110783744	110784300
chr12	110784361	110784561
chr12	110788003	110788203
chr12	111235661	111235781
chr12	113495994	113496258

chr12	113515221	113515917
chr12	113530913	113531081
chr12	113531292	113531556
chr12	113531789	113531957
chr12	113532540	113533059
chr12	113533078	113533270
chr12	113534511	113534751
chr12	114238151	114238271
chr12	117256638	117256758
chr12	120257763	120257883
chr12	123258496	123258616
chr12	124809918	124810158
chr12	124810700	124810940
chr12	124811925	124812205
chr12	124813543	124813743
chr12	124815337	124815497
chr12	124816798	124817078
chr12	124817610	124817890
chr12	124818946	124819186
chr12	124819613	124819893
chr12	124819963	124820243
chr12	124821272	124821752
chr12	124821756	124821956
chr12	124824479	124824719
chr12	124824725	124825054
chr12	124825082	124825362
chr12	124826354	124826634
chr12	124827521	124827801
chr12	124829119	124829519
chr12	124831092	124831412
chr12	124832313	124832513
chr12	124832649	124832929
chr12	124835067	124835347
chr12	124838570	124838810
chr12	124838955	124839195
chr12	124839313	124839553
chr12	124839927	124840167
chr12	124841129	124841409
chr12	124846617	124846897
chr12	124848164	124848404
chr12	124856541	124857181
chr12	124858903	124859063
chr12	124862716	124862996

chr12	124870221	124870501
chr12	124873173	124873333
chr12	124882595	124882795
chr12	124884992	124885272
chr12	124886843	124887003
chr12	124887091	124887291
chr12	124904451	124904651
chr12	124906993	124907153
chr12	124911116	124911396
chr12	124914105	124914305
chr12	124915106	124915386
chr12	124922408	124922608
chr12	124934306	124934466
chr12	124941599	124941759
chr12	124950655	124950895
chr12	124957447	124957727
chr12	124968090	124968370
chr12	124970930	124971170
chr12	124979624	124979864
chr12	126265603	126265723
chr12	129271399	129271519
chr12	132022163	132022283
chr12	132272665	132272785
chr12	132445136	132446655
chr12	132448011	132448251
chr12	132464188	132464388
chr12	132466014	132466214
chr12	132466610	132467050
chr12	132471045	132471365
chr12	132472222	132472462
chr12	132474438	132474718
chr12	132475119	132475319
chr12	132475896	132476056
chr12	132476666	132476826
chr12	132479347	132479587
chr12	132489555	132489795
chr12	132490631	132490871
chr12	132491193	132491473
chr12	132495990	132496230
chr12	132497498	132497778
chr12	132497981	132498261
chr12	132498269	132498509
chr12	132502052	132502292

chr12	132502697	132502977
chr12	132504538	132504818
chr12	132505513	132505753
chr12	132505772	132505972
chr12	132508263	132508543
chr12	132510129	132510409
chr12	132511928	132512128
chr12	132512537	132512857
chr12	132514226	132514466
chr12	132514478	132514758
chr12	132516475	132516715
chr12	132522162	132522402
chr12	132522429	132522709
chr12	132527787	132528067
chr12	132528131	132528331
chr12	132528668	132528908
chr12	132529157	132529561
chr12	132529844	132530124
chr12	132530218	132530498
chr12	132534815	132535055
chr12	132535091	132535251
chr12	132537629	132537950
chr12	132538062	132538222
chr12	132539461	132539818
chr12	132546641	132546881
chr12	132546927	132547087
chr12	132547137	132547337
chr12	132549201	132549441
chr12	132551296	132551536
chr12	132551848	132552088
chr12	132553972	132554252
chr12	132560999	132561199
chr12	132561921	132562241
chr13	19273191	19273311
chr13	22274382	22274502
chr13	23732138	23732258
chr13	25285751	25285871
chr13	28293282	28293402
chr13	28578129	28578369
chr13	28588541	28588741
chr13	28589243	28589443
chr13	28589682	28589882
chr13	28592540	28592780

chr13	28597430	28597670
chr13	28598938	28599138
chr13	28599445	28599645
chr13	28601181	28601421
chr13	28602269	28602469
chr13	28607975	28608590
chr13	28609580	28609860
chr13	28610025	28610225
chr13	28611273	28611473
chr13	28622355	28622635
chr13	28623477	28623717
chr13	28623721	28623961
chr13	28624175	28624415
chr13	28626626	28626866
chr13	28631441	28631641
chr13	28635984	28636224
chr13	28644568	28644808
chr13	28674545	28674705
chr13	28877284	28877524
chr13	28880761	28880961
chr13	28882921	28883121
chr13	28885677	28885917
chr13	28886082	28886282
chr13	28891584	28891784
chr13	28893515	28893715
chr13	28895540	28895780
chr13	28896347	28896703
chr13	28896884	28897124
chr13	28901542	28901742
chr13	28903708	28903908
chr13	28908113	28908313
chr13	28913250	28913490
chr13	28919534	28919734
chr13	28931636	28931876
chr13	28942657	28942857
chr13	28958974	28959214
chr13	28963926	28964246
chr13	28971050	28971250
chr13	28973137	28973297
chr13	28979873	28980073
chr13	29001255	29001495
chr13	29001833	29002113
chr13	29004145	29004345

chr13	29005219	29005499
chr13	29007903	29008415
chr13	29012299	29012539
chr13	29041032	29041272
chr13	29041605	29041805
chr13	29068868	29069028
chr13	31310912	31311032
chr13	32890550	32890710
chr13	32893197	32893477
chr13	32899166	32899366
chr13	32900182	32900478
chr13	32900592	32900792
chr13	32903524	32903684
chr13	32905011	32905211
chr13	32906406	32907526
chr13	32910387	32915347
chr13	32918642	32918842
chr13	32920918	32921078
chr13	32928991	32929431
chr13	32930515	32930795
chr13	32931832	32932112
chr13	32936604	32936884
chr13	32937312	32937672
chr13	32944496	32944736
chr13	32945044	32945284
chr13	32949431	32949591
chr13	32950747	32950987
chr13	32953412	32953692
chr13	32953828	32954332
chr13	32968807	32969087
chr13	32970078	32970278
chr13	32970987	32971227
chr13	32972282	32972922
chr13	34318089	34318209
chr13	37351696	37351816
chr13	40385379	40385499
chr13	43408467	43408587
chr13	46413063	46413183
chr13	47505109	47505229
chr13	48877996	48878236
chr13	48881358	48881598
chr13	48916692	48916892
chr13	48917612	48917732

chr13	48918114	48918234
chr13	48919175	48919375
chr13	48921879	48922079
chr13	48923045	48923205
chr13	48934107	48934307
chr13	48936901	48937141
chr13	48938988	48939148
chr13	48941584	48941784
chr13	48942621	48942781
chr13	48943988	48944108
chr13	48947484	48947684
chr13	48951011	48951211
chr13	48953677	48953837
chr13	48954104	48954418
chr13	48955340	48955620
chr13	48964687	48964807
chr13	48974649	48974769
chr13	48986971	48987091
chr13	48997632	48997752
chr13	49011026	49011146
chr13	49012204	49012324
chr13	49016002	49016122
chr13	49027087	49027287
chr13	49030292	49030532
chr13	49033776	49034016
chr13	49037818	49038018
chr13	49038616	49038736
chr13	49039090	49039562
chr13	49047410	49047610
chr13	49050787	49051027
chr13	49051435	49051595
chr13	49054090	49054250
chr13	49417952	49418072
chr13	52418538	52418658
chr13	55429904	55430024
chr13	58503158	58503278
chr13	61517322	61517442
chr13	63071726	63071846
chr13	64540321	64540441
chr13	67588660	67588780
chr13	70592644	70592764
chr13	73333874	73334074
chr13	73334607	73334847

chr13	73334960	73335080
chr13	73335322	73335442
chr13	73335459	73335699
chr13	73335727	73336007
chr13	73336047	73336287
chr13	73337546	73337786
chr13	73338287	73338407
chr13	73338787	73338907
chr13	73340052	73340252
chr13	73340478	73340598
chr13	73341150	73341270
chr13	73341613	73341758
chr13	73342866	73343106
chr13	73343653	73343773
chr13	73344772	73344892
chr13	73344983	73345330
chr13	73345883	73346083
chr13	73346254	73346454
chr13	73346783	73347023
chr13	73347770	73348010
chr13	73348040	73348240
chr13	73349164	73349284
chr13	73349290	73349570
chr13	73350006	73350286
chr13	73351016	73351136
chr13	73351514	73351674
chr13	73352281	73352561
chr13	73354942	73355182
chr13	73355380	73355540
chr13	73355736	73355976
chr13	73641690	73641810
chr13	76678698	76678818
chr13	79703091	79703211
chr13	83101233	83101353
chr13	86109634	86109754
chr13	86230852	86230972
chr13	89146686	89146806
chr13	92051241	92051521
chr13	92052048	92052168
chr13	92100955	92101235
chr13	92126712	92126832
chr13	92149292	92149412
chr13	92172217	92172337

chr13	92290119	92290239
chr13	92345427	92346147
chr13	92380732	92380972
chr13	92408491	92408731
chr13	92560130	92560370
chr13	92643300	92643420
chr13	92792447	92792567
chr13	92797022	92797302
chr13	92835612	92835732
chr13	93057964	93058084
chr13	93071675	93071795
chr13	93141919	93142039
chr13	93230315	93230435
chr13	93279125	93279245
chr13	93319740	93319860
chr13	93360098	93360218
chr13	93477251	93477371
chr13	93518473	93518753
chr13	95226990	95227150
chr13	95228516	95228716
chr13	95229573	95229733
chr13	95230201	95230481
chr13	95230925	95231085
chr13	95232097	95232257
chr13	95233293	95233493
chr13	95233519	95233748
chr13	95235298	95235538
chr13	95235829	95235950
chr13	95236764	95236884
chr13	95238093	95238213
chr13	95238823	95238943
chr13	95242592	95242712
chr13	95243051	95243251
chr13	95243946	95244066
chr13	95244448	95244608
chr13	95246047	95246207
chr13	95246379	95246499
chr13	95246822	95246942
chr13	95248247	95248447
chr13	95254133	95254441
chr13	95257603	95257843
chr13	95258766	95259006
chr13	95259475	95259595

chr13	95260360	95260480
chr13	95261983	95262223
chr13	95263863	95263983
chr13	95264423	95264663
chr13	95271353	95271633
chr13	95271666	95271826
chr13	95273017	95273248
chr13	95273290	95273530
chr13	95275318	95275672
chr13	95278178	95278338
chr13	95279225	95279465
chr13	95353821	95353941
chr13	95356234	95356354
chr13	95356671	95356791
chr13	95363467	95364307
chr13	95365084	95365204
chr13	95366759	95366997
chr13	95367288	95367408
chr13	95367549	95367669
chr13	95367884	95368004
chr13	95368778	95368898
chr13	95369995	95370115
chr13	95371519	95371639
chr13	98272842	98272962
chr13	101286914	101287034
chr13	104309116	104309236
chr13	107325201	107325321
chr13	110333236	110333356
chr13	110818537	110818657
chr13	113343441	113343561
chr13	113863909	113864189
chr13	113864224	113864464
chr13	113873190	113873430
chr13	113882224	113882424
chr13	113883696	113883896
chr13	113887439	113887679
chr13	113888155	113888355
chr13	113889317	113889517
chr13	113891070	113891270
chr13	113893685	113893925
chr13	113897257	113897497
chr13	113898655	113898895
chr13	113899197	113899608

chr13	113900203	113900443
chr13	113907304	113907544
chr13	113908939	113909179
chr13	113909212	113909492
chr13	113914861	113915141
chr13	113917748	113917948
chr14	20213877	20213997
chr14	21853690	21854013
chr14	21854033	21854353
chr14	21859043	21859283
chr14	21859571	21859851
chr14	21859928	21860168
chr14	21860636	21860996
chr14	21861198	21861478
chr14	21861614	21862374
chr14	21862419	21862659
chr14	21863054	21863294
chr14	21863403	21863563
chr14	21863913	21864113
chr14	21865926	21866166
chr14	21867692	21867932
chr14	21868084	21868527
chr14	21868551	21868791
chr14	21869011	21869251
chr14	21869496	21869736
chr14	21870065	21870345
chr14	21870438	21870718
chr14	21871153	21871393
chr14	21871596	21871836
chr14	21873355	21873635
chr14	21873811	21874091
chr14	21874962	21875242
chr14	21876452	21876732
chr14	21876803	21877043
chr14	21877958	21878198
chr14	21881013	21881213
chr14	21882398	21882638
chr14	21883044	21883204
chr14	21883705	21884094
chr14	21894223	21894463
chr14	21896000	21896440
chr14	21897108	21897670
chr14	21898940	21899820

chr14	21899872	21900072
chr14	23218787	23218907
chr14	23495280	23495600
chr14	23496889	23497089
chr14	23502549	23502909
chr14	23503871	23504111
chr14	26280522	26280642
chr14	29384049	29384169
chr14	32389796	32389916
chr14	34195265	34195385
chr14	35390086	35390206
chr14	35862343	35862463
chr14	35868453	35868573
chr14	35869806	35869926
chr14	35870393	35870513
chr14	35871162	35871322
chr14	35871346	35871466
chr14	35871594	35871874
chr14	35871899	35872153
chr14	35872246	35872580
chr14	35872657	35873049
chr14	35873616	35873856
chr14	35874705	35874945
chr14	35880659	35880899
chr14	38428112	38428232
chr14	41444695	41444815
chr14	44462345	44462465
chr14	47493373	47493493
chr14	50497984	50498104
chr14	53499837	53499957
chr14	54123898	54124018
chr14	56511403	56511523
chr14	58766475	58766675
chr14	58768252	58768452
chr14	58770158	58770358
chr14	58771614	58771774
chr14	58771957	58772077
chr14	58772684	58772884
chr14	58779356	58779476
chr14	58785219	58785575
chr14	58790148	58790388
chr14	58793940	58794060
chr14	58794914	58795074

chr14	58796203	58796363
chr14	58796663	58796943
chr14	58797586	58797706
chr14	58797892	58798012
chr14	58799300	58799420
chr14	58811368	58811528
chr14	58813075	58813275
chr14	58813686	58813886
chr14	58814360	58814600
chr14	58815925	58816045
chr14	58817775	58818055
chr14	58820334	58820614
chr14	58821868	58821988
chr14	58822972	58823092
chr14	58825791	58825991
chr14	58827569	58827809
chr14	58830872	58832032
chr14	58832187	58832387
chr14	58832724	58832964
chr14	58833553	58833793
chr14	58834331	58834451
chr14	58838555	58838755
chr14	59534777	59534897
chr14	62536626	62536746
chr14	65544390	65544510
chr14	68609259	68609379
chr14	71197078	71197598
chr14	71199233	71200113
chr14	71201038	71201278
chr14	71202611	71202811
chr14	71204898	71205178
chr14	71206699	71206939
chr14	71209047	71209327
chr14	71215535	71215815
chr14	71216583	71216863
chr14	71227690	71227930
chr14	71267370	71267810
chr14	71275463	71275783
chr14	71275789	71275909
chr14	71632929	71633049
chr14	74673703	74673823
chr14	77722407	77722527
chr14	80773310	80773430

chr14	83790199	83790319
chr14	85125226	85125346
chr14	86817101	86817221
chr14	89696458	89696578
chr14	89844754	89844874
chr14	90194030	90194150
chr14	90715990	90716110
chr14	91689437	91689557
chr14	92199497	92199617
chr14	92717989	92718109
chr14	92846234	92846354
chr14	93715813	93715933
chr14	94197412	94197532
chr14	94680595	94680715
chr14	95174205	95174325
chr14	95556777	95557057
chr14	95557328	95557760
chr14	95560218	95560498
chr14	95562099	95563059
chr14	95566074	95566314
chr14	95569672	95570472
chr14	95571355	95571635
chr14	95571967	95572167
chr14	95572328	95572608
chr14	95573901	95574141
chr14	95574203	95574443
chr14	95574610	95574890
chr14	95577603	95577843
chr14	95578466	95578626
chr14	95579374	95579614
chr14	95581960	95582200
chr14	95582770	95583050
chr14	95583904	95584144
chr14	95590528	95591008
chr14	95592860	95593140
chr14	95595748	95596028
chr14	95596341	95596581
chr14	95597790	95598030
chr14	95598792	95599072
chr14	95599603	95599843
chr14	95686016	95686136
chr14	95846907	95847027
chr14	96161492	96161612

chr14	96660275	96660395
chr14	97173332	97173452
chr14	97239155	97239275
chr14	97667526	97667646
chr14	98175406	98175526
chr14	98680648	98680768
chr14	98863667	98863787
chr14	99172319	99172439
chr14	99671727	99671847
chr14	100166238	100166358
chr14	100659905	100660025
chr14	101170479	101170599
chr14	101668540	101668660
chr14	101872239	101872359
chr14	102673932	102674052
chr14	103209104	103209224
chr14	103336520	103336800
chr14	103337192	103337312
chr14	103338199	103338359
chr14	103341912	103342112
chr14	103342638	103342918
chr14	103344246	103344366
chr14	103344965	103345085
chr14	103345406	103345526
chr14	103348682	103348802
chr14	103349115	103349235
chr14	103351468	103351588
chr14	103352105	103352225
chr14	103352465	103352665
chr14	103354212	103354332
chr14	103355853	103356013
chr14	103357249	103357369
chr14	103357542	103357807
chr14	103361359	103361519
chr14	103361675	103361795
chr14	103361956	103362076
chr14	103363547	103363787
chr14	103369538	103369818
chr14	103371535	103372135
chr14	103707356	103707476
chr14	104212888	104213008
chr14	104711427	104711547
chr14	104888451	104888571

chr14	105212338	105212458
chr14	105235862	105236022
chr14	105236637	105236797
chr14	105237032	105237232
chr14	105238645	105238845
chr14	105239201	105239441
chr14	105239531	105239974
chr14	105240202	105240362
chr14	105241227	105241598
chr14	105241942	105242182
chr14	105242951	105243151
chr14	105246368	105246608
chr14	105258877	105259037
chr14	105684660	105684780
chr14	105994186	106001746
chr14	106002282	106005476
chr14	106005596	106005936
chr14	106006235	106006475
chr14	106007030	106011199
chr14	106011285	106011405
chr14	106011439	106011559
chr14	106011637	106014175
chr14	106014298	106014658
chr14	106015376	106017297
chr14	106017389	106017509
chr14	106017858	106018430
chr14	106018778	106019099
chr14	106019219	106019485
chr14	106019843	106019969
chr14	106020065	106020665
chr14	106020905	106021446
chr14	106021700	106021976
chr14	106022083	106022760
chr14	106023117	106023717
chr14	106023937	106024537
chr14	106024748	106039560
chr14	106039680	106049464
chr14	106049477	106056715
chr14	106057476	106063236
chr14	106063540	106075753
chr14	106075955	106086328
chr14	106086354	106103126
chr14	106103201	106105390

chr14	106105403	106121392
chr14	106121580	106123260
chr14	106123454	106124852
chr14	106124928	106129049
chr14	106129069	106136629
chr14	106136889	106137009
chr14	106137132	106141816
chr14	106142075	106142228
chr14	106142450	106143177
chr14	106143495	106144215
chr14	106144239	106144959
chr14	106145166	106159869
chr14	106160151	106164746
chr14	106164816	106184233
chr14	106185180	106190700
chr14	106190889	106191009
chr14	106191250	106192810
chr14	106193005	106194383
chr14	106194441	106202174
chr14	106202297	106211057
chr14	106211224	106220465
chr14	106220584	106222176
chr14	106222378	106223757
chr14	106223837	106230334
chr14	106230442	106244803
chr14	106244930	106245117
chr14	106245141	106245381
chr14	106245679	106253279
chr14	106253549	106253789
chr14	106253883	106255452
chr14	106255562	106255829
chr14	106255978	106256380
chr14	106256446	106259086
chr14	106259208	106262053
chr14	106262171	106262387
chr14	106262795	106264235
chr14	106264321	106267859
chr14	106268239	106268719
chr14	106268790	106270125
chr14	106270486	106271758
chr14	106272025	106275927
chr14	106276171	106277131
chr14	106277335	106277455

chr14	106277659	106279067
chr14	106279334	106280414
chr14	106280575	106287851
chr14	106287971	106291172
chr14	106291455	106291575
chr14	106292003	106292235
chr14	106292665	106292786
chr14	106293049	106293259
chr14	106293726	106294110
chr14	106294324	106294729
chr14	106294903	106295263
chr14	106295327	106295517
chr14	106295637	106295824
chr14	106296047	106296167
chr14	106296238	106296478
chr14	106296543	106300326
chr14	106300582	106309141
chr14	106309364	106336327
chr14	106336395	106336949
chr14	106337189	106337651
chr14	106337770	106337891
chr14	106337985	106338346
chr14	106338389	106338629
chr14	106338636	106338876
chr14	106338985	106339225
chr14	106339335	106339779
chr14	106339836	106340471
chr14	106340817	106344769
chr14	106345082	106362301
chr14	106362330	106399935
chr14	106400053	106400173
chr14	106400263	106402183
chr14	106402229	106408349
chr14	106408641	106408826
chr14	106409012	106409732
chr14	106409822	106412976
chr14	106413188	106413308
chr14	106413359	106414079
chr14	106414282	106414642
chr14	106414736	106415816
chr14	106415880	106418880
chr14	106418910	106421644
chr14	106421749	106421989

chr14	106427767	106427896
chr14	106428097	106428217
chr14	106428305	106429109
chr14	106429607	106429727
chr14	106429918	106430044
chr14	106430128	106430488
chr14	106430680	106432124
chr14	106432947	106438899
chr14	106438997	106451717
chr14	106451983	106452159
chr14	106452367	106455348
chr14	106455534	106458414
chr14	106458556	106459756
chr14	106459957	106464103
chr14	106464292	106470532
chr14	106470535	106472815
chr14	106472912	106475230
chr14	106475469	106478949
chr14	106479207	106486047
chr14	106486182	106487502
chr14	106487729	106487849
chr14	106488092	106488219
chr14	106488487	106496407
chr14	106496585	106498745
chr14	106499116	106500459
chr14	106500646	106506189
chr14	106506491	106506868
chr14	106507015	106513464
chr14	106513803	106531812
chr14	106532088	106532208
chr14	106535142	106542942
chr14	106543114	106562020
chr14	106563965	106564271
chr14	106564352	106564472
chr14	106565135	106568161
chr14	106568166	106568286
chr14	106569631	106571676
chr14	106571793	106588172
chr14	106588175	106588895
chr14	106589016	106590971
chr14	106591053	106594777
chr14	106594971	106596051
chr14	106596442	106602082

chr14	106602287	106606197
chr14	106606289	106607992
chr14	106608089	106615169
chr14	106616066	106616186
chr14	106616332	106616457
chr14	106617135	106617297
chr14	106617344	106617464
chr14	106617772	106618010
chr14	106618143	106618348
chr14	106618525	106618765
chr14	106618889	106623883
chr14	106624003	106626604
chr14	106626707	106633907
chr14	106634126	106637726
chr14	106637921	106640365
chr14	106640485	106646567
chr14	106646765	106647419
chr14	106647539	106652319
chr14	106652509	106653797
chr14	106653984	106663651
chr14	106663762	106669482
chr14	106669754	106677261
chr14	106677586	106687763
chr14	106687839	106699454
chr14	106701254	106701442
chr14	106701714	106701847
chr14	106702483	106702606
chr14	106704232	106704485
chr14	106704605	106706677
chr14	106706797	106707923
chr14	106708087	106710894
chr14	106710953	106713760
chr14	106713879	106719519
chr14	106719630	106723586
chr14	106723687	106737005
chr14	106737123	106737243
chr14	106737245	106748918
chr14	106749819	106753716
chr14	106754175	106754734
chr14	106754854	106754974
chr14	106755842	106762248
chr14	106762331	106768691
chr14	106768866	106769055

chr14	106769156	106773716
chr14	106773809	106783142
chr14	106783262	106793128
chr14	106793132	106793922
chr14	106794348	106794988
chr14	106795019	106796939
chr14	106796991	106807777
chr14	106808105	106818656
chr14	106819092	106819523
chr14	106819643	106821526
chr14	106821743	106821983
chr14	106822151	106837851
chr14	106837944	106838958
chr14	106839096	106843384
chr14	106843693	106844293
chr14	106844370	106847250
chr14	106847570	106854050
chr14	106854171	106854291
chr14	106854308	106855868
chr14	106856208	106856364
chr14	106856750	106861430
chr14	106863813	106864293
chr14	106864356	106865076
chr14	106865143	106870058
chr14	106870238	106871798
chr14	106871972	106875050
chr14	106875091	106883131
chr14	106886181	106891889
chr14	106892134	106894045
chr14	106894575	106897575
chr14	106897741	106898514
chr14	106898727	106900767
chr14	106901009	106903458
chr14	106903553	106903793
chr14	106903934	106909291
chr14	106909627	106913208
chr14	106913533	106914613
chr14	106914963	106915443
chr14	106915539	106918179
chr14	106918861	106928461
chr14	106928519	106935479
chr14	106935540	106935660
chr14	106935789	106935909

chr14	106936079	106937469
chr14	106937757	106947357
chr14	106947460	106947944
chr14	106948050	106948170
chr14	106948364	106949564
chr14	106949744	106950824
chr14	106951082	106952042
chr14	106952218	106954054
chr14	106954207	106954327
chr14	106954664	106955498
chr14	106955588	106955792
chr14	106955912	106956724
chr14	106956944	106960664
chr14	106960934	106965974
chr14	106966017	106972314
chr14	106972522	106978625
chr14	106978721	106979990
chr14	106980082	106983769
chr14	106983917	106991086
chr14	106991205	106995510
chr14	106996059	106996419
chr14	106996724	106999695
chr14	107000026	107006114
chr14	107006208	107009378
chr14	107009468	107012174
chr14	107012282	107017890
chr14	107018202	107019642
chr14	107019977	107023577
chr14	107023940	107025287
chr14	107025491	107025820
chr14	107025907	107044977
chr14	107045273	107047193
chr14	107048198	107072624
chr14	107072868	107085604
chr14	107085743	107085863
chr14	107086057	107090781
chr14	107090881	107092681
chr14	107092820	107097482
chr14	107097615	107097735
chr14	107097936	107100782
chr14	107101048	107102828
chr14	107102948	107105029
chr14	107105121	107110041

chr14	107110250	107115782
chr14	107115862	107115982
chr14	107116345	107127073
chr14	107127177	107128847
chr14	107129037	107129680
chr14	107129774	107149078
chr14	107149259	107165850
chr14	107166247	107176087
chr14	107176092	107185906
chr14	107186020	107186140
chr14	107186260	107188239
chr14	107188360	107188927
chr14	107189197	107191063
chr14	107191262	107191382
chr14	107191552	107191912
chr14	107192265	107196542
chr14	107196579	107203163
chr14	107203755	107205085
chr14	107206546	107214868
chr14	107215204	107226874
chr14	107226962	107227202
chr14	107228052	107239268
chr14	107239630	107242750
chr14	107242960	107258431
chr14	107258654	107264117
chr14	107264378	107264661
chr14	107264814	107264934
chr14	107265242	107265362
chr14	107266347	107266467
chr14	107266642	107266800
chr14	107266883	107267073
chr14	107267123	107267282
chr14	107267402	107271568
chr14	107272847	107273129
chr14	107273479	107274799
chr14	107275078	107279474
chr14	107279764	107280490
chr14	107280676	107284756
chr14	107284960	107287165
chr14	107287246	107288072
chr15	20161312	20161432
chr15	23648732	23648852
chr15	26653158	26653278

chr15	29653229	29653349
chr15	32922887	32923007
chr15	35927595	35927715
chr15	38930463	38930583
chr15	40453358	40453518
chr15	40457185	40457465
chr15	40462193	40462433
chr15	40462669	40462949
chr15	40466057	40466257
chr15	40468655	40468895
chr15	40475859	40476139
chr15	40477352	40477592
chr15	40477697	40477897
chr15	40488720	40489000
chr15	40491751	40491991
chr15	40492382	40492622
chr15	40493076	40493236
chr15	40494528	40494962
chr15	40498361	40498681
chr15	40500784	40501024
chr15	40501765	40502045
chr15	40502240	40502480
chr15	40504634	40504914
chr15	40505463	40505743
chr15	40509642	40509922
chr15	40510589	40510829
chr15	40512742	40512982
chr15	41961064	41962184
chr15	41964813	41964933
chr15	41988246	41989246
chr15	41990999	41991392
chr15	41999871	42000111
chr15	42000233	42000473
chr15	42002877	42003557
chr15	42005332	42005612
chr15	42005665	42005865
chr15	42019350	42019630
chr15	42021334	42021574
chr15	42026655	42026855
chr15	42028357	42028917
chr15	42032185	42032465
chr15	42034716	42035396
chr15	42040819	42041139

chr15	42041280	42042840
chr15	42046579	42046819
chr15	42049931	42050091
chr15	42052503	42052743
chr15	42053872	42054112
chr15	42054303	42054583
chr15	42057031	42057311
chr15	42058179	42059499
chr15	45048591	45048711
chr15	48051592	48051712
chr15	51207145	51207265
chr15	52027716	52027836
chr15	54209462	54209582
chr15	57213223	57213343
chr15	60222870	60222990
chr15	63242223	63242343
chr15	66253057	66253177
chr15	67358475	67358715
chr15	67430301	67430501
chr15	67457209	67457449
chr15	67457536	67457776
chr15	67459053	67459253
chr15	67462836	67462996
chr15	67473564	67473804
chr15	67477013	67477253
chr15	67479634	67479914
chr15	67482692	67482932
chr15	69282208	69282328
chr15	72299971	72300091
chr15	74836273	74836833
chr15	74843949	74844069
chr15	74845684	74845804
chr15	74851355	74851475
chr15	74853620	74853740
chr15	74860624	74860744
chr15	74865158	74865588
chr15	74868039	74868159
chr15	74869377	74869497
chr15	74871830	74871950
chr15	74873618	74873738
chr15	74882112	74882392
chr15	74883473	74883793
chr15	74883887	74884167

chr15	74885002	74885122
chr15	74885427	74885627
chr15	74887893	74888173
chr15	75304980	75305100
chr15	78311283	78311403
chr15	79696640	79696760
chr15	81316033	81316153
chr15	84321369	84321489
chr15	86123927	86124047
chr15	86124494	86124614
chr15	87326491	87326611
chr15	90327747	90327867
chr15	90627441	90627641
chr15	90627993	90628381
chr15	90628442	90628682
chr15	90630279	90630559
chr15	90630618	90630858
chr15	90631522	90632038
chr15	90633653	90633933
chr15	90634730	90634930
chr15	90645444	90645684
chr15	91290571	91290771
chr15	91292566	91293326
chr15	91294956	91295236
chr15	91297984	91298224
chr15	91303322	91303562
chr15	91303794	91304514
chr15	91306171	91306411
chr15	91308464	91308704
chr15	91310037	91310277
chr15	91312311	91312511
chr15	91312601	91312881
chr15	91325984	91326224
chr15	91328090	91328370
chr15	91333856	91334096
chr15	91337371	91337611
chr15	91341353	91341633
chr15	91346730	91346970
chr15	91347372	91347612
chr15	91352307	91352547
chr15	91354415	91354655
chr15	91358280	91358560
chr15	93331223	93331343

chr15	96338303	96338423
chr15	99192759	99192959
chr15	99230203	99230323
chr15	99250763	99251363
chr15	99256510	99256630
chr15	99263214	99263334
chr15	99284620	99284740
chr15	99301475	99301595
chr15	99341914	99342034
chr15	99364310	99364430
chr15	99409488	99409608
chr15	99429402	99429522
chr15	99434529	99434889
chr15	99439919	99440199
chr15	99442637	99442917
chr15	99451900	99452140
chr15	99453503	99453623
chr15	99454486	99454726
chr15	99456251	99456531
chr15	99459136	99459416
chr15	99459882	99460122
chr15	99465358	99465678
chr15	99467052	99467292
chr15	99467693	99467973
chr15	99472717	99472957
chr15	99473398	99473598
chr15	99478027	99478307
chr15	99478479	99478719
chr15	99482369	99482649
chr15	99486096	99486336
chr15	99491749	99492105
chr15	99500260	99500700
chr15	99504377	99504497
chr15	102341944	102342064
chr16	105260	105380
chr16	3108972	3109092
chr16	3777716	3779876
chr16	3780270	3780390
chr16	3781173	3781493
chr16	3781717	3781997
chr16	3785980	3786260
chr16	3786593	3786914
chr16	3788516	3788716

chr16	3789529	3789769
chr16	3790246	3790594
chr16	3794848	3795008
chr16	3795236	3795396
chr16	3795885	3796005
chr16	3799575	3799735
chr16	3801667	3801867
chr16	3807232	3807432
chr16	3807809	3808049
chr16	3808813	3809013
chr16	3811495	3811615
chr16	3817675	3817955
chr16	3819124	3819404
chr16	3820558	3820998
chr16	3823704	3823944
chr16	3824511	3824751
chr16	3827555	3827715
chr16	3827957	3828237
chr16	3828287	3828407
chr16	3828659	3828859
chr16	3830685	3830925
chr16	3831155	3831355
chr16	3832410	3832530
chr16	3832665	3832945
chr16	3838201	3838425
chr16	3841938	3842138
chr16	3843366	3843646
chr16	3860551	3860831
chr16	3865049	3865169
chr16	3867020	3867140
chr16	3871225	3871345
chr16	3900293	3901013
chr16	3922931	3923051
chr16	3929774	3929974
chr16	6110487	6110607
chr16	8987807	8988007
chr16	8988127	8988247
chr16	8988351	8988551
chr16	8988596	8988756
chr16	8988847	8989047
chr16	8989448	8989648
chr16	8990805	8991005
chr16	8992175	8992335

chr16	8992341	8992541
chr16	8992767	8992887
chr16	8992931	8993091
chr16	8993417	8993657
chr16	8994336	8994536
chr16	8994800	8995144
chr16	8995891	8996091
chr16	8996188	8996418
chr16	8996739	8996859
chr16	8997071	8997311
chr16	8998236	8998476
chr16	8998995	8999235
chr16	8999487	8999607
chr16	8999923	9000163
chr16	9000240	9000480
chr16	9002152	9002352
chr16	9004542	9004742
chr16	9008405	9008525
chr16	9009055	9009441
chr16	9010305	9010465
chr16	9010827	9011067
chr16	9012841	9013041
chr16	9014159	9014444
chr16	9014744	9014864
chr16	9014962	9015202
chr16	9017030	9017310
chr16	9023296	9023496
chr16	9024101	9024301
chr16	9027368	9027488
chr16	9030354	9030554
chr16	9035063	9035183
chr16	9035323	9035443
chr16	9050238	9050358
chr16	9056250	9056450
chr16	9057022	9057182
chr16	9127446	9127566
chr16	9653995	9654115
chr16	12157840	12157960
chr16	15166831	15166951
chr16	18799555	18799675
chr16	21951355	21951475
chr16	24958925	24959045
chr16	27963289	27963409

chr16	28889855	28890135
chr16	28890328	28890528
chr16	28890762	28890962
chr16	28892167	28892407
chr16	28893720	28893960
chr16	28895835	28896035
chr16	28898452	28898652
chr16	28898734	28899054
chr16	28900050	28900330
chr16	28905419	28905619
chr16	28905756	28905996
chr16	28906088	28906328
chr16	28909263	28909503
chr16	28909542	28909782
chr16	28911889	28912249
chr16	28913153	28913433
chr16	28913485	28913725
chr16	28913843	28914043
chr16	28914045	28914285
chr16	28914289	28914529
chr16	28914582	28914822
chr16	28914922	28915122
chr16	28915433	28915593
chr16	28915670	28915830
chr16	30964880	30965000
chr16	34197432	34197552
chr16	46723538	46723658
chr16	47276777	47276897
chr16	47754505	47754625
chr16	48358307	48358427
chr16	48867487	48867607
chr16	49364995	49365115
chr16	49733003	49733123
chr16	49874690	49874810
chr16	50376735	50376855
chr16	50783601	50784121
chr16	50785505	50785825
chr16	50787086	50787206
chr16	50787422	50787542
chr16	50788182	50788382
chr16	50791189	50791309
chr16	50792207	50792327
chr16	50803569	50803721

chr16	50807495	50807615
chr16	50807718	50807838
chr16	50808980	50809180
chr16	50810034	50810234
chr16	50811693	50811893
chr16	50813565	50813965
chr16	50815099	50815379
chr16	50816186	50816426
chr16	50817158	50817278
chr16	50818180	50818420
chr16	50819051	50819171
chr16	50819505	50819625
chr16	50819849	50819969
chr16	50820711	50820911
chr16	50821649	50821809
chr16	50822903	50823023
chr16	50825414	50825654
chr16	50826461	50826661
chr16	50827415	50827660
chr16	50828115	50828395
chr16	50829406	50829566
chr16	50830186	50830466
chr16	50831195	50831315
chr16	50834641	50834841
chr16	50900250	50900370
chr16	51428895	51429015
chr16	51920030	51920150
chr16	52742198	52742318
chr16	52919505	52919625
chr16	53489644	53489764
chr16	53981360	53981480
chr16	54469574	54469694
chr16	55033633	55033753
chr16	55527817	55527937
chr16	55778085	55778205
chr16	56038407	56038527
chr16	56510238	56510358
chr16	57012279	57012399
chr16	58803486	58803606
chr16	61807196	61807316
chr16	64847480	64847600
chr16	68307387	68307507
chr16	71319586	71319706

chr16	72821042	72821602
chr16	72821642	72822002
chr16	72822037	72822557
chr16	72822567	72822847
chr16	72827130	72831370
chr16	72831398	72832638
chr16	72833856	72834096
chr16	72845455	72845695
chr16	72845750	72845990
chr16	72863617	72863817
chr16	72906998	72907118
chr16	72923605	72923885
chr16	72931025	72931145
chr16	72981170	72981290
chr16	72984355	72984875
chr16	72991304	72991704
chr16	72991714	72992594
chr16	72992659	72994059
chr16	73029100	73029220
chr16	73045789	73045909
chr16	73073748	73073868
chr16	73612059	73612179
chr16	74125305	74125425
chr16	74348983	74349103
chr16	74686105	74686225
chr16	75231624	75231744
chr16	75717869	75717989
chr16	76224988	76225108
chr16	76629599	76629719
chr16	77134182	77134302
chr16	77353913	77354033
chr16	77637031	77637151
chr16	78133610	78133850
chr16	78142251	78142451
chr16	78143546	78143783
chr16	78148821	78149101
chr16	78198012	78198252
chr16	78312446	78312606
chr16	78420700	78420900
chr16	78458739	78458979
chr16	78466356	78466676
chr16	78623946	78624066
chr16	78790655	78790855

chr16	79116818	79116938
chr16	79245487	79246047
chr16	79610275	79610395
chr16	79619721	79619921
chr16	79619985	79620105
chr16	79620377	79620497
chr16	79620996	79621116
chr16	79623024	79623144
chr16	79624788	79624908
chr16	79625506	79625626
chr16	79626745	79626865
chr16	79627004	79627124
chr16	79627221	79627341
chr16	79628055	79628295
chr16	79628303	79628503
chr16	79628741	79628861
chr16	79629731	79629851
chr16	79632377	79632497
chr16	79632658	79633818
chr16	80111081	80111201
chr16	80356988	80357108
chr16	80631360	80631480
chr16	81143395	81143515
chr16	81644228	81644348
chr16	82135450	82135570
chr16	82637697	82637817
chr16	83145289	83145409
chr16	83377141	83377261
chr16	83643873	83643993
chr16	84132472	84132592
chr16	84657050	84657170
chr16	86384877	86384997
chr16	89386748	89386868
chr16	89804942	89805182
chr16	89805235	89805435
chr16	89805478	89805758
chr16	89805823	89806023
chr16	89806282	89806522
chr16	89807142	89807342
chr16	89809156	89809396
chr16	89811302	89811542
chr16	89812923	89813163
chr16	89813188	89813348

chr16	89815000	89815240
chr16	89816083	89816363
chr16	89818487	89818687
chr16	89824950	89825190
chr16	89828313	89828513
chr16	89831245	89831525
chr16	89833496	89833696
chr16	89836218	89836458
chr16	89836520	89836720
chr16	89836906	89837106
chr16	89838033	89838273
chr16	89839615	89839855
chr16	89842086	89842286
chr16	89845153	89845480
chr16	89846220	89846420
chr16	89849216	89849562
chr16	89851196	89851436
chr16	89857757	89857997
chr16	89858265	89858545
chr16	89858816	89859016
chr16	89862249	89862489
chr16	89865396	89865706
chr16	89865949	89866109
chr16	89869607	89869807
chr16	89871623	89871863
chr16	89874597	89874797
chr16	89877062	89877262
chr16	89877267	89877547
chr16	89880874	89881074
chr16	89882219	89882459
chr16	89882883	89883083
chr17	76450	76570
chr17	598224	598344
chr17	1098672	1098792
chr17	1588899	1589019
chr17	2095651	2095771
chr17	2599211	2599331
chr17	3093818	3093938
chr17	3118321	3118441
chr17	3480386	3480506
chr17	3615768	3615888
chr17	3822775	3822975
chr17	3827987	3828187

chr17	3828603	3828803
chr17	3831223	3831383
chr17	3831450	3831690
chr17	3831895	3832135
chr17	3832560	3832800
chr17	3833572	3833812
chr17	3838428	3838628
chr17	3839541	3839781
chr17	3840679	3840959
chr17	3844252	3844612
chr17	3844718	3844958
chr17	3845842	3846082
chr17	3846630	3846870
chr17	3847928	3848168
chr17	3848314	3848514
chr17	3850656	3851176
chr17	3853747	3854126
chr17	3854506	3854746
chr17	3854806	3855046
chr17	3856581	3856781
chr17	3856895	3857095
chr17	3867472	3867632
chr17	4638502	4638622
chr17	5113362	5113482
chr17	5616518	5616638
chr17	6114812	6114933
chr17	6593861	6593981
chr17	7101230	7101350
chr17	7565214	7565374
chr17	7566342	7566462
chr17	7569442	7569757
chr17	7570167	7570287
chr17	7570755	7570995
chr17	7571391	7571511
chr17	7572867	7573067
chr17	7573879	7574079
chr17	7576480	7576740
chr17	7576809	7577206
chr17	7577296	7577653
chr17	7578132	7578725
chr17	7579310	7579590
chr17	7579610	7579955
chr17	7614538	7614658

chr17	7748820	7749060
chr17	7749138	7749338
chr17	7749385	7749625
chr17	7749663	7750375
chr17	7750416	7750776
chr17	7750854	7753054
chr17	7753081	7753321
chr17	7753342	7753542
chr17	7754321	7754561
chr17	7754599	7754911
chr17	7754919	7755159
chr17	7755225	7755700
chr17	7755763	7756003
chr17	7756260	7756500
chr17	7756523	7756843
chr17	8090847	8090967
chr17	8595032	8595152
chr17	9095621	9095741
chr17	9120810	9120930
chr17	9613623	9613743
chr17	10614381	10614501
chr17	11135641	11135761
chr17	11501803	11502243
chr17	11511403	11511683
chr17	11513671	11513911
chr17	11514919	11515199
chr17	11515802	11516042
chr17	11520713	11520953
chr17	11522861	11523101
chr17	11532677	11532957
chr17	11535861	11536061
chr17	11539905	11540145
chr17	11543543	11543743
chr17	11547901	11548061
chr17	11550331	11550571
chr17	11554373	11554653
chr17	11556058	11556338
chr17	11568097	11568337
chr17	11572338	11572618
chr17	11572678	11573118
chr17	11583064	11583304
chr17	11583982	11584262
chr17	11592877	11593757

chr17	11597129	11597369
chr17	11597580	11597820
chr17	11603003	11603243
chr17	11604381	11604621
chr17	11607519	11607759
chr17	11608281	11608561
chr17	11622608	11622848
chr17	11631086	11631286
chr17	11642155	11642395
chr17	11645440	11645680
chr17	11648097	11648417
chr17	11650857	11651097
chr17	11656093	11656333
chr17	11659838	11660038
chr17	11660815	11661055
chr17	11666703	11666983
chr17	11671716	11671996
chr17	11672422	11672662
chr17	11684268	11684548
chr17	11686913	11687113
chr17	11687616	11687856
chr17	11696760	11697040
chr17	11700871	11701151
chr17	11710989	11711269
chr17	11713504	11713744
chr17	11725187	11725427
chr17	11725695	11725975
chr17	11726114	11726354
chr17	11737944	11738224
chr17	11757312	11757752
chr17	11772410	11772610
chr17	11774868	11775148
chr17	11778263	11778503
chr17	11783335	11783575
chr17	11784507	11784747
chr17	11786844	11787124
chr17	11790091	11790331
chr17	11795038	11795318
chr17	11797631	11797871
chr17	11805992	11806272
chr17	11808931	11809171
chr17	11827074	11827314
chr17	11833176	11833416

chr17	11835286	11835566
chr17	11837152	11837432
chr17	11840613	11840893
chr17	11845570	11845850
chr17	11865178	11865578
chr17	11872610	11872850
chr17	11924041	11924401
chr17	11935518	11935678
chr17	11956932	11957052
chr17	11958136	11958376
chr17	11984619	11984899
chr17	11985514	11985634
chr17	11998673	11999071
chr17	12011046	12011286
chr17	12013637	12013797
chr17	12016493	12016733
chr17	12028549	12028749
chr17	12031049	12031169
chr17	12032389	12032669
chr17	12043098	12043258
chr17	12043513	12043633
chr17	12044400	12044640
chr17	12134126	12134246
chr17	15135070	15135190
chr17	15935583	15935823
chr17	15938028	15938308
chr17	15942717	15942997
chr17	15943701	15943861
chr17	15950195	15950475
chr17	15952090	15952370
chr17	15960799	15961079
chr17	15961159	15961439
chr17	15961728	15961968
chr17	15964701	15965221
chr17	15965361	15965641
chr17	15967281	15967441
chr17	15967447	15967607
chr17	15968118	15968398
chr17	15968783	15969023
chr17	15971181	15971461
chr17	15973458	15973858
chr17	15974706	15974986
chr17	15975400	15975600

chr17	15976672	15976952
chr17	15978791	15979071
chr17	15983207	15983447
chr17	15983660	15984100
chr17	15989535	15989815
chr17	15990432	15990712
chr17	15995150	15995390
chr17	16001625	16001865
chr17	16004541	16005141
chr17	16012042	16012282
chr17	16021151	16021391
chr17	16022667	16022867
chr17	16024354	16024594
chr17	16029482	16029682
chr17	16040543	16040783
chr17	16041418	16041578
chr17	16042301	16042581
chr17	16046864	16047064
chr17	16049635	16049915
chr17	16052697	16052897
chr17	16054836	16054996
chr17	16055205	16055365
chr17	16056600	16056760
chr17	16062010	16062250
chr17	16068263	16068503
chr17	16075092	16075332
chr17	16089814	16090054
chr17	16097709	16097949
chr17	18135693	18135813
chr17	21151046	21151166
chr17	25402103	25402223
chr17	28434908	28435028
chr17	28731354	28731474
chr17	29422166	29422446
chr17	29423928	29424048
chr17	29461838	29461958
chr17	29473292	29473412
chr17	29482817	29482937
chr17	29482952	29483192
chr17	29485969	29486169
chr17	29490158	29490438
chr17	29496861	29497061
chr17	29508393	29508553

chr17	29508685	29508845
chr17	29509484	29509724
chr17	29519995	29520115
chr17	29527386	29527666
chr17	29527995	29528235
chr17	29528385	29528545
chr17	29533203	29533443
chr17	29541415	29541655
chr17	29545979	29546179
chr17	29548817	29549057
chr17	29550403	29550643
chr17	29552070	29552310
chr17	29553437	29553717
chr17	29554192	29554352
chr17	29554482	29554682
chr17	29556022	29556502
chr17	29556802	29557042
chr17	29557218	29557458
chr17	29557801	29558001
chr17	29559048	29559248
chr17	29559668	29559948
chr17	29560005	29560245
chr17	29562569	29562849
chr17	29562887	29563087
chr17	29575949	29576189
chr17	29578299	29578419
chr17	29579906	29580066
chr17	29584620	29584820
chr17	29585320	29585560
chr17	29585998	29586198
chr17	29587339	29587579
chr17	29588681	29588921
chr17	29592201	29592401
chr17	29612349	29612469
chr17	29626475	29626675
chr17	29644791	29644911
chr17	29652833	29653273
chr17	29654506	29654866
chr17	29657294	29657534
chr17	29661634	29661794
chr17	29661812	29662092
chr17	29663300	29663540
chr17	29663652	29663932

chr17	29664372	29664612
chr17	29664786	29664946
chr17	29664968	29665208
chr17	29665672	29665872
chr17	29667472	29667712
chr17	29669969	29670209
chr17	29676083	29676323
chr17	29677148	29677388
chr17	29679185	29679473
chr17	29683418	29683658
chr17	29683922	29684162
chr17	29684236	29684436
chr17	29685448	29685688
chr17	29685929	29686089
chr17	29687496	29687736
chr17	29694189	29694349
chr17	29700981	29701221
chr17	29703941	29704061
chr17	29705847	29706007
chr17	31438398	31438518
chr17	34444060	34444180
chr17	37453234	37453354
chr17	37884174	37884294
chr17	37895472	37895592
chr17	37907419	37907539
chr17	37916239	37916359
chr17	37922014	37922774
chr17	37925833	37925953
chr17	37933841	37934081
chr17	37938034	37938154
chr17	37943671	37943791
chr17	37944448	37944688
chr17	37947612	37947892
chr17	37948895	37949215
chr17	37949864	37949984
chr17	37957257	37957377
chr17	37964376	37964496
chr17	37970305	37970425
chr17	37985565	37985805
chr17	37988297	37988457
chr17	38004414	38004534
chr17	38020275	38020475
chr17	38040703	38040823

chr17	38045665	38045785
chr17	38051288	38051408
chr17	38064227	38064347
chr17	38069889	38070009
chr17	40498213	40498333
chr17	41197636	41197876
chr17	41199609	41199769
chr17	41201094	41201254
chr17	41201993	41202193
chr17	41203026	41203186
chr17	41209010	41209210
chr17	41215289	41215449
chr17	41215849	41216009
chr17	41219568	41219768
chr17	41222939	41223259
chr17	41226302	41226582
chr17	41228447	41228687
chr17	41231303	41231463
chr17	41234366	41234646
chr17	41242904	41243104
chr17	41243444	41246884
chr17	41247820	41247980
chr17	41249203	41249363
chr17	41251744	41251944
chr17	41256088	41256328
chr17	41256828	41257028
chr17	41258431	41258591
chr17	41262494	41262654
chr17	41267689	41267849
chr17	41275993	41276153
chr17	41277100	41277300
chr17	43341944	43342224
chr17	43342459	43342699
chr17	43343835	43344115
chr17	43344391	43344631
chr17	43344749	43345149
chr17	43347714	43347994
chr17	43348280	43348600
chr17	43350801	43351041
chr17	43351435	43351675
chr17	43351775	43352015
chr17	43362127	43362367
chr17	43363784	43364424

chr17	43364504	43364744
chr17	43366536	43366736
chr17	43367843	43368123
chr17	43513381	43513501
chr17	46521968	46522088
chr17	47677671	47677951
chr17	47679157	47679437
chr17	47684552	47684792
chr17	47685183	47685343
chr17	47688590	47688870
chr17	47695816	47696016
chr17	47696286	47696526
chr17	47696531	47696811
chr17	47697931	47698131
chr17	47699248	47699488
chr17	47699536	47699696
chr17	47700033	47700233
chr17	49537702	49537822
chr17	52548065	52548185
chr17	55550872	55550992
chr17	56082290	56082490
chr17	56082723	56083003
chr17	56083100	56083340
chr17	56083655	56083935
chr17	56084259	56084539
chr17	58677751	58678271
chr17	58678886	58679006
chr17	58700855	58701135
chr17	58711155	58711395
chr17	58725227	58725467
chr17	58733940	58734220
chr17	58734228	58734388
chr17	58740334	58740934
chr17	59760638	59761518
chr17	59763181	59763541
chr17	59770731	59770931
chr17	59793204	59793444
chr17	59820314	59820554
chr17	59821732	59822012
chr17	59853702	59853982
chr17	59857545	59857825
chr17	59858143	59858423
chr17	59861567	59861847

chr17	59870903	59871143
chr17	59876440	59876680
chr17	59878584	59878864
chr17	59885812	59886132
chr17	59924401	59924641
chr17	59926433	59926673
chr17	59934365	59934645
chr17	59937092	59937332
chr17	59938753	59938953
chr17	61805341	61805461
chr17	62006534	62006894
chr17	62007088	62007288
chr17	62007429	62007749
chr17	62008641	62008801
chr17	62009507	62009667
chr17	62121333	62121573
chr17	62122153	62122353
chr17	62122660	62122900
chr17	62125161	62125401
chr17	62126348	62126628
chr17	62130119	62130359
chr17	62130581	62130781
chr17	62131584	62131824
chr17	62132042	62132242
chr17	62132959	62133381
chr17	62135137	62135377
chr17	62137768	62138008
chr17	62141288	62141568
chr17	62142507	62142707
chr17	62144001	62144321
chr17	62145480	62145720
chr17	62149281	62149521
chr17	62152430	62152630
chr17	62156957	62157157
chr17	62158071	62158231
chr17	62175420	62175660
chr17	62207165	62207556
chr17	64831597	64831717
chr17	67857570	67857690
chr17	70117507	70117987
chr17	70118846	70119126
chr17	70119666	70120026
chr17	70120117	70120557

chr17	70864365	70864485
chr17	73911655	73911775
chr17	74732234	74732554
chr17	74732861	74733261
chr17	76923366	76923486
chr17	78237428	78237628
chr17	78246981	78247261
chr17	78252495	78252775
chr17	78261435	78262192
chr17	78262372	78262612
chr17	78263406	78263686
chr17	78264307	78264587
chr17	78265406	78265646
chr17	78268500	78268820
chr17	78269344	78269624
chr17	78272099	78272339
chr17	78280039	78280279
chr17	78280864	78281064
chr17	78282754	78283034
chr17	78286749	78287029
chr17	78290932	78291132
chr17	78292974	78293294
chr17	78298773	78299053
chr17	78301562	78301842
chr17	78302062	78302342
chr17	78305778	78306458
chr17	78307847	78308127
chr17	78309932	78310332
chr17	78311315	78311854
chr17	78312978	78314178
chr17	78316908	78317148
chr17	78317595	78317875
chr17	78318461	78322101
chr17	78323517	78323757
chr17	78324048	78324248
chr17	78325463	78325623
chr17	78326679	78326919
chr17	78327248	78327528
chr17	78327752	78328032
chr17	78328188	78328428
chr17	78332063	78332303
chr17	78333793	78334073
chr17	78335470	78335750

chr17	78336887	78337127
chr17	78337379	78337619
chr17	78338172	78338412
chr17	78341477	78341717
chr17	78341731	78341971
chr17	78343246	78343717
chr17	78345609	78345849
chr17	78346307	78346547
chr17	78346749	78346989
chr17	78348203	78348443
chr17	78349494	78349734
chr17	78350081	78350361
chr17	78350622	78350822
chr17	78351498	78351658
chr17	78353366	78353566
chr17	78354568	78354848
chr17	78355316	78355556
chr17	78356160	78356320
chr17	78356724	78356924
chr17	78357462	78357742
chr17	78358784	78359024
chr17	78359279	78359479
chr17	78360019	78360259
chr17	78360470	78360710
chr17	78362350	78362550
chr17	78362949	78363189
chr17	78363572	78363772
chr17	78363783	78364023
chr17	78367081	78367361
chr17	79477627	79478183
chr17	79478212	79478652
chr17	79478728	79478888
chr17	79478928	79479168
chr17	79479198	79479438
chr17	79924371	79924491
chr18	212696	212816
chr18	3215170	3215290
chr18	6300875	6300995
chr18	8080296	8080416
chr18	9420444	9420564
chr18	12422829	12422949
chr18	12703054	12703214
chr18	12706494	12706774

chr18	12712649	12712809
chr18	12718454	12718694
chr18	12720455	12720735
chr18	12724437	12724677
chr18	12725383	12725583
chr18	18540793	18540913
chr18	19728584	19728704
chr18	19739441	19739561
chr18	19742808	19742928
chr18	19743466	19743586
chr18	19743994	19744114
chr18	19746757	19746877
chr18	19751078	19751836
chr18	19751846	19752324
chr18	19756831	19757111
chr18	19761356	19761596
chr18	19762661	19763072
chr18	19765679	19765799
chr18	19778868	19778988
chr18	19780562	19780842
chr18	19786794	19786914
chr18	19809409	19809529
chr18	19814736	19814856
chr18	19821510	19821630
chr18	19827413	19827533
chr18	21495422	21495542
chr18	21543956	21544076
chr18	23877114	23877234
chr18	24546576	24546696
chr18	27571974	27572094
chr18	30783164	30783284
chr18	33790953	33791073
chr18	36805319	36805439
chr18	39859977	39860097
chr18	42866490	42866610
chr18	45881427	45881547
chr18	48885359	48885479
chr18	51932956	51933076
chr18	54949022	54949142
chr18	57962921	57963041
chr18	60027180	60027300
chr18	60795804	60796044
chr18	60964997	60965117

chr18	60985270	60985910
chr18	63969531	63969651
chr18	67021940	67022060
chr18	70029362	70029482
chr18	72923751	72923951
chr18	72997399	73000599
chr18	73056218	73056338
chr18	76062879	76062999
chr19	266979	267099
chr19	929520	929904
chr19	930813	930933
chr19	932411	932747
chr19	933961	934081
chr19	939564	939804
chr19	941513	941633
chr19	960031	960325
chr19	960993	961113
chr19	962100	962220
chr19	963760	963880
chr19	964195	964483
chr19	964824	965088
chr19	966563	966875
chr19	967650	967770
chr19	968347	968563
chr19	971827	972115
chr19	1611656	1611896
chr19	1612198	1612438
chr19	1615275	1615827
chr19	1619050	1619525
chr19	1619719	1619911
chr19	1620910	1621244
chr19	1621782	1622470
chr19	1623888	1624056
chr19	1625520	1625760
chr19	1627307	1627475
chr19	1631886	1632174
chr19	1632271	1632463
chr19	1646293	1646485
chr19	1650127	1650295
chr19	3274544	3274664
chr19	4090530	4090770
chr19	4094393	4094553
chr19	4095316	4095516

chr19	4097214	4097414
chr19	4099185	4099425
chr19	4100958	4101332
chr19	4102312	4102512
chr19	4110439	4110719
chr19	4117401	4117641
chr19	4123698	4123898
chr19	6278637	6278757
chr19	8968488	8968608
chr19	9282318	9282438
chr19	12291550	12291670
chr19	15271470	15272526
chr19	15273115	15273427
chr19	15276120	15276384
chr19	15276593	15276905
chr19	15278008	15278272
chr19	15280842	15281034
chr19	15281132	15281372
chr19	15281426	15281690
chr19	15284876	15285212
chr19	15288330	15288906
chr19	15289584	15289800
chr19	15289832	15290096
chr19	15290120	15290360
chr19	15290830	15291118
chr19	15291433	15291697
chr19	15291716	15292028
chr19	15292379	15292619
chr19	15295051	15295315
chr19	15295665	15295881
chr19	15296011	15296549
chr19	15297635	15297851
chr19	15297912	15298152
chr19	15298210	15298330
chr19	15298640	15298856
chr19	15298994	15299210
chr19	15299748	15300293
chr19	15302231	15302471
chr19	15302496	15302736
chr19	15302759	15303119
chr19	15303138	15303378
chr19	15308253	15308445
chr19	15311549	15311765

chr19	18315569	18315689
chr19	20741528	20741648
chr19	21326298	21326418
chr19	24338222	24338342
chr19	28246682	28246802
chr19	31078158	31078278
chr19	31246795	31246915
chr19	31767428	31770228
chr19	31770244	31770684
chr19	34253339	34253459
chr19	36208901	36209301
chr19	36210326	36210486
chr19	36210675	36212715
chr19	36213217	36213664
chr19	36213896	36214176
chr19	36214296	36214456
chr19	36214630	36214910
chr19	36215484	36215684
chr19	36215838	36216277
chr19	36216332	36216771
chr19	36217095	36217295
chr19	36217900	36218213
chr19	36218290	36218933
chr19	36218955	36219115
chr19	36219148	36219268
chr19	36219632	36220248
chr19	36220369	36220489
chr19	36220826	36221110
chr19	36221182	36221827
chr19	36221962	36222082
chr19	36222802	36223042
chr19	36223102	36224422
chr19	36224442	36224818
chr19	36224912	36225032
chr19	36225559	36225679
chr19	36225938	36226058
chr19	36227539	36227929
chr19	36227935	36228215
chr19	36228413	36228693
chr19	36228701	36229147
chr19	36229180	36229460
chr19	37326224	37326344
chr19	40649942	40650062

chr19	41869331	41869451
chr19	42381333	42381493
chr19	42383051	42383731
chr19	42384690	42384850
chr19	42384890	42385090
chr19	42788789	42788989
chr19	42790857	42791414
chr19	42791416	42791656
chr19	42791667	42792184
chr19	42793020	42793260
chr19	42793305	42793585
chr19	42793931	42794171
chr19	42794361	42795641
chr19	42795683	42795923
chr19	42796178	42796674
chr19	42796704	42797024
chr19	42797090	42797450
chr19	42797725	42798005
chr19	42798023	42798510
chr19	42798701	42799146
chr19	42799176	42799416
chr19	43805671	43805791
chr19	46877345	46877465
chr19	47177765	47178005
chr19	47178232	47178432
chr19	47181611	47181931
chr19	47184855	47185055
chr19	47188665	47188825
chr19	47192734	47193014
chr19	47193809	47194009
chr19	47194947	47195107
chr19	47195135	47195295
chr19	47197120	47197400
chr19	47200372	47200532
chr19	47200948	47201148
chr19	47204012	47204410
chr19	47207416	47207656
chr19	47207684	47207964
chr19	47214109	47214349
chr19	47217068	47217308
chr19	47219387	47219627
chr19	49945790	49945910
chr19	51315763	51315883

chr19	52946089	52946209
chr19	55964660	55964780
chr19	56166414	56166574
chr19	56170182	56170382
chr19	56170523	56170763
chr19	56171484	56171644
chr19	56171833	56172033
chr19	56172359	56172599
chr19	56173037	56173237
chr19	56173825	56174025
chr19	56174920	56175160
chr19	56179832	56180216
chr19	56180397	56180597
chr19	56180793	56181073
chr19	56185246	56185486
chr19	57318613	57318733
chr19	57323214	57323334
chr19	57325034	57328954
chr19	57329059	57329259
chr19	57329322	57329442
chr19	57329918	57330118
chr19	57330708	57330828
chr19	57331851	57331971
chr19	57332970	57333170
chr19	57334062	57334262
chr19	57334459	57334699
chr19	57334902	57335102
chr19	57335118	57335238
chr19	57335626	57336026
chr19	57339145	57339265
chr19	57344818	57345058
chr19	57640027	57642827
chr19	57642985	57643105
chr19	57643108	57643228
chr19	57644387	57644507
chr19	57644897	57645017
chr19	57646509	57646629
chr19	57646954	57647074
chr19	57647175	57647295
chr19	57648099	57648219
chr19	57650167	57650287
chr19	57652226	57652346
chr19	57739186	57739306

chr19	58965600	58965720
chr20	68689	68809
chr20	3081578	3081698
chr20	4312092	4312212
chr20	6083433	6083553
chr20	9110699	9110819
chr20	12175418	12175538
chr20	15183833	15183953
chr20	18187539	18187659
chr20	21233493	21233613
chr20	22562413	22563811
chr20	22564772	22564972
chr20	23038980	23039100
chr20	23347492	23347612
chr20	24242078	24242198
chr20	29615755	29615875
chr20	29618231	29618351
chr20	29619445	29619685
chr20	29620155	29620275
chr20	29623139	29623393
chr20	29623981	29624141
chr20	29625314	29625434
chr20	29625828	29626028
chr20	29628178	29628504
chr20	29629995	29630235
chr20	29630602	29630802
chr20	29631350	29631470
chr20	29631483	29631683
chr20	29632565	29632765
chr20	29633622	29633742
chr20	29633803	29634003
chr20	29846342	29846462
chr20	30946526	30946686
chr20	30947491	30947651
chr20	30954127	30954327
chr20	30955430	30955630
chr20	30956771	30956971
chr20	30959483	30959683
chr20	30959869	30960069
chr20	31015870	31016276
chr20	31017087	31017287
chr20	31017659	31017899
chr20	31018862	31019062

chr20	31019065	31019533
chr20	31020635	31020835
chr20	31021083	31021723
chr20	31022227	31025147
chr20	33170692	33170812
chr20	36202288	36202408
chr20	39210244	39210364
chr20	39307602	39307722
chr20	39307753	39307873
chr20	39311502	39311622
chr20	39313629	39313749
chr20	39314609	39314729
chr20	39316504	39317504
chr20	39318442	39318682
chr20	39319124	39319244
chr20	39320690	39320810
chr20	39321241	39321361
chr20	39323256	39323496
chr20	39323756	39323980
chr20	39325127	39325247
chr20	39327195	39327337
chr20	39327435	39327555
chr20	39657602	39657762
chr20	39657978	39658138
chr20	39689981	39690181
chr20	39704646	39704846
chr20	39704906	39705106
chr20	39706169	39706329
chr20	39708672	39708872
chr20	39709742	39709942
chr20	39713034	39713274
chr20	39721049	39721289
chr20	39725800	39726040
chr20	39726795	39727035
chr20	39728669	39728909
chr20	39729780	39730060
chr20	39741353	39741633
chr20	39742582	39742822
chr20	39743944	39744144
chr20	39744854	39745094
chr20	39746752	39746992
chr20	39750282	39750482
chr20	39750580	39750860

chr20	39751765	39752005
chr20	39797404	39797524
chr20	40709433	40709593
chr20	40710469	40710709
chr20	40713263	40713543
chr20	40714310	40714550
chr20	40727006	40727246
chr20	40730710	40730990
chr20	40733143	40733423
chr20	40735373	40735613
chr20	40738936	40739216
chr20	40743747	40743987
chr20	40744610	40744770
chr20	40746991	40747191
chr20	40748512	40748672
chr20	40757331	40757531
chr20	40770498	40770698
chr20	40789966	40790206
chr20	40827818	40828098
chr20	40864803	40864963
chr20	40877274	40877514
chr20	40884615	40884735
chr20	40898984	40899144
chr20	40911066	40911226
chr20	40944339	40944659
chr20	40979198	40979438
chr20	40980704	40980944
chr20	41076794	41077034
chr20	41100893	41101213
chr20	41306492	41306812
chr20	41385048	41385328
chr20	41400012	41400252
chr20	41408798	41408998
chr20	41419810	41420130
chr20	41514389	41514629
chr20	41818224	41818424
chr20	42215414	42215534
chr20	44746927	44747145
chr20	44747249	44747369
chr20	44747886	44748006
chr20	44748038	44748158
chr20	44748489	44748609
chr20	44750363	44750577

chr20	44750814	44751165
chr20	44751201	44751441
chr20	44751711	44751911
chr20	44752243	44752363
chr20	44754847	44754967
chr20	44755050	44755170
chr20	44755229	44755389
chr20	44756719	44757082
chr20	44757152	44757272
chr20	44757346	44757466
chr20	44757479	44757719
chr20	45258580	45258700
chr20	48260143	48260263
chr20	51261835	51261955
chr20	52950986	52951106
chr20	54275842	54275962
chr20	57280938	57281058
chr20	57415150	57415910
chr20	57428294	57430414
chr20	57430555	57430755
chr20	57466792	57466912
chr20	57470602	57470802
chr20	57473937	57474097
chr20	57474906	57475106
chr20	57478531	57478906
chr20	57480386	57480586
chr20	57484163	57484323
chr20	57484341	57484918
chr20	57484950	57485190
chr20	57485322	57485522
chr20	57485670	57485950
chr20	60282500	60282620
chr21	14687511	14687631
chr21	17713137	17713257
chr21	19842516	19842636
chr21	20716221	20716341
chr21	23759335	23759455
chr21	26798029	26798149
chr21	29803963	29804083
chr21	32818692	32818812
chr21	35819003	35819123
chr21	36164429	36164909
chr21	36171538	36171818

chr21	36193878	36194078
chr21	36206662	36206942
chr21	36228626	36228826
chr21	36231722	36231922
chr21	36252811	36253051
chr21	36259134	36259414
chr21	36261923	36262083
chr21	36265140	36265340
chr21	36421087	36421247
chr21	38860080	38860200
chr21	41897312	41897432
chr21	43718422	43718542
chr21	44513165	44513405
chr21	44514526	44514951
chr21	44515496	44515696
chr21	44515748	44515908
chr21	44520515	44520675
chr21	44521428	44521588
chr21	44524368	44524568
chr21	44527502	44527662
chr21	44898266	44898386
chr21	47917499	47917619
chr22	16114184	16114304
chr22	19118641	19118761
chr22	19960427	19960819
chr22	21062280	21062440
chr22	21063525	21063725
chr22	21064140	21064340
chr22	21064926	21065206
chr22	21065576	21065816
chr22	21066716	21067152
chr22	21067503	21067743
chr22	21068680	21069051
chr22	21071930	21072130
chr22	21072925	21073165
chr22	21075525	21075765
chr22	21080722	21080882
chr22	21081461	21081741
chr22	21082006	21082206
chr22	21083577	21083817
chr22	21083859	21084059
chr22	21084119	21084359
chr22	21087202	21087442

chr22	21087957	21088197
chr22	21088266	21088546
chr22	21088621	21088901
chr22	21096459	21096699
chr22	21096833	21097113
chr22	21098861	21099061
chr22	21101810	21102050
chr22	21104137	21104337
chr22	21105522	21105722
chr22	21105902	21106102
chr22	21107132	21107532
chr22	21115532	21115732
chr22	21119051	21119291
chr22	21119335	21119575
chr22	21119806	21120046
chr22	21147425	21147585
chr22	21150377	21150657
chr22	21152803	21153043
chr22	21153352	21153592
chr22	21153906	21154106
chr22	21156206	21156446
chr22	21157423	21157663
chr22	21158517	21158757
chr22	21159237	21159477
chr22	21161597	21161797
chr22	21165187	21165387
chr22	21167579	21167859
chr22	21172703	21172903
chr22	21173918	21174198
chr22	21174762	21174962
chr22	21178557	21178757
chr22	21188763	21188963
chr22	21192840	21193080
chr22	21212763	21212963
chr22	21212992	21213192
chr22	22142978	22143098
chr22	24129302	24129502
chr22	24133891	24134131
chr22	24135690	24135930
chr22	24143106	24143346
chr22	24145425	24145665
chr22	24158899	24159179
chr22	24167386	24167626

chr22	24175704	24175944
chr22	24176267	24176427
chr22	25144852	25144972
chr22	28156728	28156848
chr22	29182009	29182129
chr22	29182439	29182559
chr22	29183072	29183192
chr22	29185052	29185172
chr22	29185716	29185836
chr22	29189385	29189505
chr22	29191161	29191721
chr22	29191818	29191938
chr22	29191987	29192227
chr22	29192609	29192729
chr22	29193008	29193248
chr22	29194985	29195185
chr22	29195905	29196065
chr22	29196278	29196518
chr22	29196696	29196816
chr22	29198090	29198210
chr22	29204871	29204991
chr22	31160479	31160599
chr22	34171319	34171439
chr22	37196620	37196740
chr22	40257931	40258051
chr22	41488955	41489155
chr22	41489859	41489979
chr22	41507339	41507459
chr22	41513187	41513827
chr22	41519301	41519421
chr22	41521714	41522095
chr22	41523481	41523761
chr22	41525850	41526050
chr22	41527374	41527654
chr22	41529996	41530116
chr22	41531763	41531963
chr22	41533605	41533845
chr22	41536102	41536302
chr22	41536998	41537293
chr22	41537528	41537648
chr22	41540963	41541083
chr22	41541466	41541586
chr22	41542701	41542861

chr22	41543795	41544042
chr22	41544990	41545230
chr22	41545763	41546203
chr22	41547786	41548066
chr22	41548161	41548401
chr22	41549860	41549980
chr22	41550957	41551157
chr22	41553172	41553412
chr22	41554359	41554559
chr22	41556585	41556785
chr22	41558171	41558291
chr22	41558674	41558834
chr22	41560015	41560175
chr22	41562556	41562716
chr22	41564407	41564647
chr22	41564677	41564917
chr22	41565463	41565663
chr22	41566352	41566632
chr22	41568444	41568724
chr22	41569548	41569847
chr22	41570684	41570804
chr22	41572231	41572551
chr22	41572768	41574968
chr22	43278088	43278208
chr22	46422878	46422998
chr22	49430856	49430976
chrX	2710780	2710900
chrX	5713062	5713182
chrX	8761565	8761685
chrX	11761973	11762093
chrX	14795879	14795999
chrX	15808558	15808718
chrX	15809016	15809176
chrX	15817935	15818135
chrX	15819376	15819656
chrX	15821764	15821964
chrX	15822176	15822376
chrX	15826274	15826474
chrX	15827281	15827481
chrX	15833786	15834026
chrX	15836657	15836817
chrX	15838284	15838484
chrX	15840849	15841369

chrX	17797849	17797969
chrX	20871651	20871771
chrX	23884277	23884397
chrX	26955981	26956101
chrX	29959841	29959961
chrX	32966447	32966567
chrX	35993426	35993546
chrX	39007235	39007355
chrX	39893161	39893281
chrX	39908656	39908776
chrX	39909126	39909286
chrX	39911347	39911667
chrX	39913074	39913354
chrX	39913467	39913627
chrX	39914126	39914246
chrX	39914573	39914813
chrX	39915658	39915778
chrX	39916350	39916630
chrX	39917339	39917459
chrX	39918301	39918421
chrX	39921378	39921658
chrX	39921981	39922341
chrX	39922852	39923212
chrX	39923580	39923860
chrX	39925268	39925388
chrX	39928322	39928442
chrX	39930178	39930458
chrX	39930836	39930996
chrX	39931597	39934547
chrX	39935665	39935825
chrX	39937039	39937239
chrX	39938164	39938284
chrX	39944875	39944995
chrX	40982829	40983029
chrX	40988185	40988465
chrX	40990649	40990849
chrX	40993913	40994153
chrX	40996045	40996285
chrX	40999846	41000086
chrX	41000204	41000484
chrX	41000494	41000734
chrX	41002479	41002759
chrX	41003706	41003946

chrX	41007604	41007844
chrX	41010121	41010361
chrX	41012147	41012387
chrX	41021986	41022186
chrX	41025095	41025495
chrX	41026682	41026882
chrX	41027245	41027485
chrX	41029227	41029507
chrX	41029657	41029937
chrX	41030845	41031005
chrX	41031030	41031270
chrX	41043175	41043415
chrX	41043628	41043948
chrX	41045712	41045952
chrX	41047187	41047427
chrX	41048504	41048784
chrX	41055087	41055327
chrX	41055437	41055677
chrX	41055777	41056057
chrX	41056549	41056829
chrX	41057751	41058031
chrX	41060282	41060562
chrX	41064530	41064770
chrX	41069708	41069988
chrX	41073751	41074031
chrX	41075128	41075928
chrX	41076414	41076654
chrX	41077597	41077877
chrX	41078299	41078539
chrX	41082442	41082682
chrX	41083964	41084244
chrX	41084245	41084445
chrX	41088443	41088723
chrX	41088789	41089109
chrX	41089701	41089901
chrX	41091588	41091828
chrX	41193447	41193607
chrX	41193793	41194073
chrX	41196609	41196769
chrX	41198232	41198392
chrX	41200682	41200922
chrX	41201706	41202139
chrX	41202416	41202656

chrX	41202932	41203132
chrX	41203231	41203711
chrX	41204384	41204848
chrX	41205432	41205712
chrX	41205716	41205916
chrX	41206068	41206308
chrX	41206514	41206754
chrX	41206852	41207012
chrX	42009351	42009471
chrX	44732737	44733017
chrX	44733121	44733281
chrX	44782251	44782371
chrX	44785917	44786201
chrX	44786276	44786396
chrX	44794289	44794409
chrX	44811016	44811136
chrX	44820482	44820682
chrX	44833780	44834015
chrX	44870154	44870314
chrX	44872730	44872850
chrX	44879264	44879384
chrX	44879794	44880034
chrX	44886643	44886763
chrX	44887389	44887509
chrX	44894122	44894282
chrX	44896816	44897016
chrX	44910900	44911100
chrX	44913016	44913256
chrX	44918199	44918399
chrX	44918481	44918721
chrX	44919213	44919453
chrX	44919811	44920051
chrX	44920516	44920716
chrX	44921842	44922042
chrX	44922664	44923064
chrX	44928812	44929612
chrX	44931636	44931756
chrX	44935886	44936126
chrX	44937597	44937797
chrX	44938373	44938613
chrX	44941772	44942076
chrX	44942658	44942898
chrX	44945066	44945266

chrX	44948941	44949221
chrX	44949918	44950158
chrX	44953143	44953263
chrX	44965740	44965940
chrX	44966597	44966837
chrX	44969268	44969548
chrX	44970541	44970741
chrX	45013037	45013157
chrX	48178898	48179018
chrX	51344449	51344569
chrX	51637342	51637502
chrX	51637666	51637946
chrX	51638142	51638862
chrX	51639498	51640178
chrX	51640255	51640415
chrX	51640602	51640762
chrX	51640836	51641036
chrX	51641169	51641481
chrX	51641627	51641787
chrX	51643234	51643434
chrX	51644637	51645037
chrX	53221876	53222068
chrX	53222139	53222523
chrX	53222562	53222874
chrX	53222897	53223089
chrX	53223320	53223920
chrX	53224061	53224301
chrX	53224358	53224646
chrX	53225046	53225286
chrX	53225866	53226226
chrX	53226897	53227113
chrX	53227613	53227877
chrX	53227887	53228393
chrX	53230672	53230984
chrX	53230987	53231203
chrX	53239544	53240092
chrX	53240625	53240889
chrX	53240920	53241136
chrX	53243817	53244081
chrX	53244923	53245437
chrX	53246271	53246511
chrX	53246918	53247206
chrX	53247398	53247638

chrX	53247774	53247942
chrX	53249963	53250155
chrX	53250841	53251033
chrX	53253864	53254128
chrX	54731288	54731408
chrX	63749094	63749214
chrX	66865436	66865556
chrX	70022838	70022958
chrX	70338553	70338753
chrX	70339166	70339406
chrX	70339511	70339751
chrX	70339801	70340081
chrX	70340791	70341031
chrX	70341111	70341351
chrX	70341398	70341678
chrX	70341982	70342262
chrX	70342307	70342507
chrX	70342535	70342775
chrX	70342890	70343130
chrX	70343386	70343626
chrX	70343983	70344263
chrX	70344553	70344753
chrX	70344770	70345050
chrX	70345132	70345412
chrX	70345457	70345617
chrX	70345824	70346064
chrX	70346122	70346402
chrX	70346760	70347040
chrX	70347131	70347371
chrX	70347716	70347996
chrX	70348077	70348357
chrX	70348387	70348627
chrX	70348894	70349342
chrX	70349477	70349757
chrX	70349834	70350114
chrX	70351335	70351535
chrX	70351869	70352109
chrX	70352167	70352447
chrX	70352671	70352911
chrX	70352917	70353117
chrX	70354141	70354381
chrX	70354510	70354750
chrX	70354882	70355162

chrX	70356117	70356517
chrX	70356663	70356943
chrX	70357019	70357259
chrX	70357346	70357813
chrX	70360407	70360607
chrX	70360679	70360879
chrX	70360896	70361096
chrX	70361217	70361337
chrX	70361673	70361873
chrX	70361966	70362126
chrX	73152927	73153047
chrX	76812080	76812200
chrX	79924013	79924133
chrX	82937006	82937126
chrX	85939024	85939144
chrX	88941105	88941225
chrX	92504100	92504220
chrX	95633558	95633678
chrX	98655997	98656117
chrX	100604828	100604988
chrX	100608140	100608380
chrX	100608816	100609016
chrX	100609569	100609729
chrX	100611027	100611267
chrX	100611717	100611997
chrX	100612453	100612613
chrX	100613241	100613481
chrX	100613564	100613724
chrX	100614227	100614387
chrX	100615026	100615186
chrX	100615509	100615789
chrX	100617114	100617274
chrX	100617492	100617732
chrX	100624926	100625126
chrX	100626574	100626734
chrX	100629472	100629672
chrX	100630081	100630321
chrX	101751388	101751508
chrX	104751474	104751594
chrX	107781637	107781757
chrX	111161141	111161261
chrX	114186055	114186175
chrX	117346792	117346912

chrX	119660563	119660763
chrX	119663892	119664172
chrX	119666222	119666502
chrX	119668268	119668508
chrX	119669621	119669861
chrX	119670715	119670955
chrX	119671921	119672121
chrX	119672449	119672689
chrX	119673054	119673294
chrX	119674200	119674440
chrX	119675395	119675635
chrX	119676754	119676954
chrX	119677522	119677722
chrX	119677913	119678113
chrX	119678276	119678556
chrX	119679235	119679435
chrX	119680336	119680536
chrX	119680922	119681162
chrX	119691716	119691956
chrX	119693598	119693758
chrX	119693855	119694589
chrX	119695155	119695315
chrX	119708338	119708538
chrX	120377072	120377192
chrX	123408705	123408825
chrX	123514384	123515144
chrX	123516470	123516710
chrX	123517473	123518713
chrX	123519520	123519920
chrX	123525889	123526209
chrX	123538878	123539118
chrX	123540112	123540392
chrX	123554155	123554675
chrX	123556104	123556504
chrX	123587143	123587383
chrX	123615577	123615817
chrX	123620968	123621168
chrX	123630850	123631130
chrX	123637361	123637601
chrX	123654360	123654640
chrX	123657204	123657484
chrX	123663673	123663873
chrX	123680711	123680951

chrX	123695473	123695713
chrX	123697510	123697710
chrX	123699177	123699457
chrX	123775620	123775860
chrX	123778949	123779229
chrX	123780509	123780709
chrX	123785748	123785988
chrX	123787393	123787673
chrX	123805488	123805728
chrX	123838861	123839101
chrX	123870786	123871066
chrX	124028092	124028252
chrX	124029819	124030099
chrX	124097373	124097613
chrX	126410083	126410203
chrX	129474247	129474367
chrX	132475062	132475182
chrX	135476623	135476743
chrX	138485263	138485383
chrX	141598059	141598179
chrX	144612599	144612719
chrX	147628033	147628153
chrX	150654784	150654904
chrX	153659539	153659659
chrY	2655120	2655240
chrY	5755490	5755610
chrY	8775118	8775238
chrY	13315335	13315455
chrY	16331498	16331618
chrY	19360582	19360702
chrY	21867875	21868235
chrY	21868270	21868582
chrY	21868630	21868798
chrY	21869032	21869632
chrY	21869768	21870008
chrY	21870075	21870363
chrY	21870709	21870949
chrY	21871335	21871695
chrY	21872008	21872200
chrY	21872201	21872417
chrY	21877179	21877943
chrY	21878105	21878417
chrY	21878433	21878649

chrY	21882706	21883250
chrY	21885176	21885368
chrY	21893604	21893868
chrY	21893883	21894099
chrY	21894416	21894680
chrY	21897178	21897442
chrY	21897451	21897691
chrY	21901360	21901600
chrY	21903144	21903432
chrY	21903561	21903801
chrY	21904990	21905182
chrY	21906213	21906477
chrY	22597046	22597166
chrY	28498294	28498414
