# Supplementary material for: A DNA target-enrichment approach to detect mutations, copy number changes and immunoglobulin translocations in multiple myeloma
Source: Blood Cancer J. 2016 Sep 2;6(9):e467–. doi: 10.1038/bcj.2016.72 (PMC5056967; doi:10.1038/bcj.2016.72)
Supplement: Supplementary Table 3 [file bcj201672x6.pdf]

Supplementary table S3.

one read mapping in				and the other in any of the following			
CHR	START	END	Comment	CHR	START	END	Comment
14	105994256	107288051	IGH	4	1800000	1980000	t(4;14)
				6	41750000	42000000	t(6;14)
				8	125000000	130000000	t(8;14)
				11	64456000	69456000	t(11;14)
				16	78000000	79630000	t(14;16)
				20	38119089	39119089	t(14;20)
