# Supplementary material for: A DNA target-enrichment approach to detect mutations, copy number changes and immunoglobulin translocations in multiple myeloma
Source: Blood Cancer J. 2016 Sep 2;6(9):e467–. doi: 10.1038/bcj.2016.72 (PMC5056967; doi:10.1038/bcj.2016.72)
Supplement: Supplementary Table 4 [file bcj201672x7.pdf]

CHR	POS	WT	MT	cell_line	cell_type	type	Gene	cDNA	Prot	effect	Targeted_depth	Targeted_AF
12	18800877	T	G	ARH-77	MM	sub	PIK3C2G	c.4253T>G	p.V1418G	missense	143	0.5
16	72831276	A	G	ARH-77	MM	sub	ZFHX3	c.5305T>C	p.S1769P	missense	67	0.45
19	15302285	C	A	ARH-77	MM	sub	NOTCH3	c.986G>T	p.C329F	missense	74	0.55
2	141083421	G	A	ARH-77	MM	sub	LRP1B	c.12250C>T	p.R4084C	missense	106	0.49
11	102195846	A	T	BALL-1	non_MM	sub	BIRC3	c.606A>T	p.R202S	missense	355	0.99
12	18656350	A	T	BALL-1	non_MM	sub	PIK3C2G	c.3029A>T	p.K1010M	missense	231	0.52
12	40646786	C	T	BALL-1	non_MM	sub	LRRK2	c.1256C>T	p.A419V	missense	252	1
13	32910842	A	G	BALL-1	non_MM	sub	BRCA2	c.2350A>G	p.M784V	missense	415	0.992771084
13	92051394	G	A	BALL-1	non_MM	sub	GPC5	c.94G>A	p.E32K	missense	1582	0.049
13	92345954	G	A	BALL-1	non_MM	sub	GPC5	c.839G>A	p.G280D	missense	386	0.31
16	89858913	C	T	BALL-1	non_MM	sub	FANCA	c.1049G>A	p.R350Q	missense	109	0.43
17	15978874	T	G	BALL-1	non_MM	sub	NCOR1	c.3644A>C	p.K1215T	missense	122	0.99
17	41203106	T	C	BALL-1	non_MM	sub	BRCA1	c.5306A>G	p.Y1769C	missense	146	0.51
17	7577096	T	C	BALL-1	non_MM	sub	TP53	c.842A>G	p.D281G	missense	83	0.975903614
17	78341626	A	G	BALL-1	non_MM	sub	RNF213	c.6169A>G	p.T2057A	missense	317	0.057
6	51735401	T	G	BALL-1	non_MM	sub	PKHD1	c.7387A>C	p.K2463Q	missense	353	0.062
7	124487025	A	G	BALL-1	non_MM	sub	POT1	c.977T>C	p.V326A	missense	236	1
7	82581918	A	G	BALL-1	non_MM	sub	PCLO	c.8351T>C	p.I2784T	missense	457	0.044
8	77768069	G	A	BALL-1	non_MM	sub	ZFHX4	c.8777G>A	p.R2926H	missense	326	0.49
8	77776592	C	T	BALL-1	non_MM	sub	ZFHX4	c.10507C>T	p.L3503F	missense	290	0.083
X	70342119	G	A	BALL-1	non_MM	sub	MED12	c.1171G>A	p.G391S	missense	160	0.15
11	102201850	G	A	CESS	non_MM	sub	BIRC3	c.1202G>A	p.R401K	missense	365	0.446575342
15	90630768	T	C	CESS	non_MM	sub	IDH2	c.718A>G	p.I240V	missense	256	0.52
19	31770175	G	A	CESS	non_MM	sub	TSHZ3	c.524C>T	p.T175M	missense	139	0.47
2	152359878	T	G	CESS	non_MM	sub	NEB	c.18717A>C	p.Q6239H	missense	211	0.62
3	193201770	C	T	CESS	non_MM	sub	ATP13A4	c.763G>A	p.V255I	missense	351	0.48
5	112176429	T	C	CESS	non_MM	sub	APC	c.5138T>C	p.L1713S	missense	349	0.55
5	13864684	A	G	CESS	non_MM	sub	DNAH5	c.4418T>C	p.I1473T	missense	208	0.45
7	82583068	T	A	CESS	non_MM	sub	PCLO	c.7201A>T	p.I2401L	missense	121	0.42
8	77690582	C	T	CESS	non_MM	sub	ZFHX4	c.3154C>T	p.R1052W	missense	248	0.5
9	8465638	C	T	CESS	non_MM	sub	PTPRD	c.3542G>A	p.R1181H	missense	262	0.43
1	16259531	G	A	CTV-1	MM	sub	SPEN	c.6796G>A	p.D2266N	missense	96	0.46
1	16260138	C	T	CTV-1	MM	sub	SPEN	c.7403C>T	p.P2468L	missense	123	0.48
1	27099036	G	A	CTV-1	MM	sub	ARID1A	c.3452G>A	p.S1151N	missense	97	0.53
1	27106662	G	T	CTV-1	MM	sub	ARID1A	c.6273G>T	p.W2091C	missense	132	0.39
10	104161574	G	A	CTV-1	MM	sub	NFKB2	c.2366G>A	p.G789D	missense	108	0.5
10	43610015	G	T	CTV-1	MM	sub	RET	c.1967G>T	p.C656F	missense	130	0.46
11	102195549	C	A	CTV-1	MM	sub	BIRC3	c.309C>A	p.N103K	missense	210	0.5
11	61081381	G	C	CTV-1	MM	sub	DDB1	c.1814C>G	p.A605G	missense	109	0.39
11	62571390	C	T	CTV-1	MM	sub	NXF1	c.89G>A	p.R30Q	missense	179	0.54
11	70332590	G	A	CTV-1	MM	sub	SHANK2	c.3790C>T	p.R1264W	missense	107	0.3
11	92085985	G	A	CTV-1	MM	sub	FAT3	c.707G>A	p.R236Q	missense	120	0.52
12	48376668	C	T	CTV-1	MM	sub	COL2A1	c.2156G>A	p.R719H	missense	116	0.47
12	6495274	C	T	CTV-1	MM	sub	LTBR	c.515C>T	p.A172V	missense	46	0.65
13	28592653	C	T	CTV-1	MM	sub	FLT3	c.2492G>A	p.G831E	missense	175	0.5
13	73346926	C	A	CTV-1	MM	sub	DIS3	c.1291G>T	p.E431*	nonsense	115	0.33
14	71199721	C	A	CTV-1	MM	sub	MAP3K9	c.2407G>T	p.E803*	nonsense	115	0.46
15	42028660	C	T	CTV-1	MM	sub	MGA	c.4198C>T	p.P1400S	missense	153	0.46
15	42040883	C	G	CTV-1	MM	sub	MGA	c.5408C>G	p.T1803R	missense	95	0.4
15	91292901	G	A	CTV-1	MM	sub	BLM	c.403G>A	p.A135T	missense	185	0.48
16	28909648	C	T	CTV-1	MM	sub	ATP2A1	c.1640C>T	p.A547V	missense	132	0.48
16	89871784	C	T	CTV-1	MM	sub	FANCA	c.613G>A	p.A205T	missense	131	0.44
16	8993456	A	C	CTV-1	MM	sub	USP7	c.2463+5T>G	p.?	ess splice	107	0.49
17	11572991	T	G	CTV-1	MM	sub	DNAH9	c.3233T>G	p.I1078S	missense	146	0.49
17	11833393	C	A	CTV-1	MM	sub	DNAH9	c.12088C>A	p.L4030M	missense	54	0.35
17	29588745	G	A	CTV-1	MM	sub	NF1	c.4594G>A	p.G1532R	missense	141	0.44
17	3851047	C	T	CTV-1	MM	sub	ATP2A3	c.733G>A	p.E245K	missense	137	0.53
17	3851063	C	A	CTV-1	MM	sub	ATP2A3	c.717G>T	p.M239I	missense	129	0.43
17	43348402	C	T	CTV-1	MM	sub	MAP3K14	c.1190G>A	p.G397D	missense	120	0.49
17	43362195	T	C	CTV-1	MM	sub	MAP3K14	c.1271A>G	p.Q424R	missense	88	0.44
17	59937249	C	T	CTV-1	MM	sub	BRIP1	c.113G>A	p.S38N	missense	125	0.45
17	7752773	C	T	CTV-1	MM	sub	KDM6B	c.3167C>T	p.P1056L	missense	124	0.52
17	7752983	G	A	CTV-1	MM	sub	KDM6B	c.3377G>A	p.R1126Q	missense	129	0.54
18	60985887	C	A	CTV-1	MM	sub	BCL2	c.13G>T	p.G5W	missense	84	0.46
19	47177931	G	A	CTV-1	MM	sub	PRKD2	c.2486C>T	p.T829M	missense	137	0.48
2	152396917	G	A	CTV-1	MM	sub	NEB	c.15730C>T	p.P5244S	missense	98	0.41
2	25469134	C	A	CTV-1	MM	sub	DNMT3A	c.1324G>T	p.E442*	nonsense	109	0.51
2	9347281	G	T	CTV-1	MM	sub	ASAP2	c.48G>T	p.E16D	missense	125	0.34
20	39317132	G	A	CTV-1	MM	sub	MAFB	c.359C>T	p.P120L	missense	101	0.49
3	138374245	C	T	CTV-1	MM	sub	PIK3CB	c.3199G>A	p.D1067N	missense	118	0.49
3	170800100	G	A	CTV-1	MM	sub	TNIK	c.3253C>T	p.R1085W	missense	123	0.47
3	52685795	T	C	CTV-1	MM	sub	PBRM1	c.677A>G	p.E226G	missense	124	0.3
4	153247289	G	A	CTV-1	MM	sub	FBXW7	c.1513C>T	p.R505C	missense	152	0.47
4	153247373	C	T	CTV-1	MM	sub	FBXW7	c.1429G>A	p.G477S	missense	102	0.48
4	187558064	G	A	CTV-1	MM	sub	FAT1	c.3647C>T	p.T1216I	missense	87	0.38
4	187627816	C	T	CTV-1	MM	sub	FAT1	c.3166G>A	p.V1056M	missense	133	0.53
5	13769218	G	T	CTV-1	MM	sub	DNAH5	c.9748C>A	p.Q3250K	missense	113	0.55
5	149501471	C	T	CTV-1	MM	sub	PDGFRB	c.2316G>A	p.M772I	missense	113	0.44

5	56177668	C	T	CTV-1	MM	sub	MAP3K1	c.2641C>T	p.Q881*	nonsense	110	0.47
6	117631340	T	C	CTV-1	MM	sub	ROS1	c.6338A>G	p.D2113G	missense	151	0.21
6	117683833	T	C	CTV-1	MM	sub	ROS1	c.3314A>G	p.N1105S	missense	197	0.23
6	128302281	T	C	CTV-1	MM	sub	PTPRK	c.3709A>G	p.I1237V	missense	190	0.24
6	15512580	G	A	CTV-1	MM	sub	JARID2	c.3094G>A	p.A1032T	missense	112	0.46
6	157100553	G	A	CTV-1	MM	sub	ARID1B	c.1316G>A	p.S439N	missense	131	0.22
6	31548874	C	T	CTV-1	MM	sub	LTB	c.347G>A	p.G116E	missense	143	0.46
7	148544344	C	T	CTV-1	MM	sub	EZH2	c.47G>A	p.R16Q	missense	89	0.53
7	82579156	G	A	CTV-1	MM	sub	PCLO	c.10748C>T	p.A3583V	missense	128	0.55
7	82785556	C	A	CTV-1	MM	sub	PCLO	c.401G>T	p.S134I	missense	99	0.43
9	139390623	G	T	CTV-1	MM	sub	NOTCH1	c.7568C>A	p.S2523*	nonsense	160	0.43
9	8486271	G	A	CTV-1	MM	sub	PTPRD	c.2546C>T	p.T849I	missense	121	0.55
X	39932305	G	T	CTV-1	MM	sub	BCOR	c.2294C>A	p.S765Y	missense	75	1
X	39932813	G	A	CTV-1	MM	sub	BCOR	c.1786C>T	p.Q596*	nonsense	67	1
X	70341449	G	A	CTV-1	MM	sub	MED12	c.884G>A	p.R295H	missense	34	1
X	70349177	G	C	CTV-1	MM	sub	MED12	c.3589G>C	p.V1197L	missense	54	0.98
3	47098571	C	G	EHEB	non_MM	sub	SETD2	c.6703G>C	p.V2235L	missense	107	0.45
1	115256529	T	A	HL-60	non_MM	sub	NRAS	c.182A>T	p.Q61L	missense	347	0.54
9	21971120	G	A	HL-60	non_MM	sub	CDKN2A	c.238C>T	p.R80*	nonsense	114	1
1	115256530	G	T	IM-9	MM	sub	NRAS	c.181C>A	p.Q61K	missense	190	0.46
13	32907075	C	A	IM-9	MM	sub	BRCA2	c.1460C>A	p.A487E	missense	212	0.53
19	31770055	C	T	IM-9	MM	sub	TSHZ3	c.644G>A	p.R215H	missense	146	0.44
2	152515585	C	T	IM-9	MM	sub	NEB	c.6069G>A	p.M2023I	missense	186	0.483870968
2	152536299	T	C	IM-9	MM	sub	NEB	c.3191A>G	p.Y1064C	missense	113	0.522123894
22	21080786	C	G	IM-9	MM	sub	PI4KA	c.4811G>C	p.R1604T	missense	57	0.37
4	187541228	G	A	IM-9	MM	sub	FAT1	c.6512C>T	p.P2171L	missense	185	0.45
8	77766214	A	G	IM-9	MM	sub	ZFHX4	c.6922A>G	p.M2308V	missense	222	0.53
17	12044528	T	C	JVM-3	non_MM	sub	MAP2K4	c.1151T>C	p.I384T	missense	179	0.49
2	178096390	T	A	JVM-3	non_MM	sub	NFE2L2	c.941A>T	p.N314I	missense	225	0.56
3	47147516	A	C	JVM-3	non_MM	sub	SETD2	c.4810T>G	p.Y1604D	missense	208	0.37
3	47162351	C	T	JVM-3	non_MM	sub	SETD2	c.3775G>A	p.G1259S	missense	276	0.032608696
4	187525619	T	A	JVM-3	non_MM	sub	FAT1	c.10460A>T	p.K3487M	missense	143	0.29
7	140453132	T	A	JVM-3	non_MM	sub	BRAF	c.1803A>T	p.K601N	missense	121	0.45
11	119169190	G	C	K-562	non_MM	sub	CBL	c.2374G>C	p.D792H	missense	183	0.31
12	56488312	C	T	K-562	non_MM	sub	ERBB3	c.1831C>T	p.R611W	missense	139	0.61
19	42795793	G	A	K-562	non_MM	sub	CIC	c.2782G>A	p.A928T	missense	103	0.62
2	178096531	G	C	K-562	non_MM	sub	NFE2L2	c.800C>G	p.T267R	missense	391	0.49
20	31022288	C	A	K-562	non_MM	sub	ASXL1	c.1773C>A	p.Y591*	nonsense	173	0.32
3	3194255	G	A	K-562	non_MM	sub	CRBN	c.1033C>T	p.P345S	missense	266	0.29
4	187524859	T	C	K-562	non_MM	sub	FAT1	c.10821A>G	p.I3607M	missense	260	0.67
4	1941422	C	T	K-562	non_MM	sub	WHSC1	c.1798C>T	p.R600*	nonsense	267	0.33
5	13794109	C	A	K-562	non_MM	sub	DNAH5	c.7946G>T	p.G2649V	missense	307	0.47
6	117715410	C	A	K-562	non_MM	sub	ROS1	c.1079G>T	p.R360I	missense	273	0.29
6	160450640	A	G	K-562	non_MM	sub	IGF2R	c.835A>G	p.S279G	missense	225	0.49
7	82544404	C	T	K-562	non_MM	sub	PCLO	c.12898G>A	p.G4300R	missense	269	0.17
7	82581620	C	A	K-562	non_MM	sub	PCLO	c.8649G>T	p.W2883C	missense	280	0.19
8	77618486	C	A	K-562	non_MM	sub	ZFHX4	c.2163C>A	p.D721E	missense	212	0.33
1	120483279	C	A	KARPAS-45	non_MM	sub	NOTCH2	c.3082G>T	p.E1028*	nonsense	221	0.23
1	16256495	G	A	KARPAS-45	non_MM	sub	SPEN	c.3760G>A	p.D1254N	missense	448	0.074
1	16260017	C	A	KARPAS-45	non_MM	sub	SPEN	c.7282C>A	p.P2428T	missense	264	0.24
1	193219834	A	T	KARPAS-45	non_MM	sub	CDC73	c.1588A>T	p.R530*	nonsense	257	0.089
1	23885511	C	T	KARPAS-45	non_MM	sub	ID3	c.301-1G>A	p.?	ess splice	257	0.23
1	27106003	G	A	KARPAS-45	non_MM	sub	ARID1A	c.5614G>A	p.A1872T	missense	313	0.11
1	43812513	C	G	KARPAS-45	non_MM	sub	MPL	c.1216C>G	p.L406V	missense	202	0.23
1	99161244	A	G	KARPAS-45	non_MM	sub	SNX7	c.810A>G	p.I270M	missense	277	0.051
10	43615557	A	G	KARPAS-45	non_MM	sub	RET	c.2636A>G	p.N879S	missense	183	0.18
10	63829505	G	T	KARPAS-45	non_MM	sub	ARID5B	c.1148G>T	p.G383V	missense	266	0.29
10	89720852	C	T	KARPAS-45	non_MM	sub	PTEN	c.1003C>T	p.R335*	nonsense	378	0.99
10	96006280	A	G	KARPAS-45	non_MM	sub	PLCE1	c.2998A>G	p.S1000G	missense	350	0.24
10	96058279	A	G	KARPAS-45	non_MM	sub	PLCE1	c.5311A>G	p.R1771G	missense	336	0.45
11	108128318	G	T	KARPAS-45	non_MM	sub	ATM	c.2361G>T	p.L787F	missense	206	0.21
11	108173611	A	G	KARPAS-45	non_MM	sub	ATM	c.5351A>G	p.N1784S	missense	307	0.21
11	108173686	T	C	KARPAS-45	non_MM	sub	ATM	c.5426T>C	p.L1809P	missense	302	0.24
11	119156061	G	A	KARPAS-45	non_MM	sub	CBL	c.1726G>A	p.D576N	missense	319	0.52
11	533833	C	G	KARPAS-45	non_MM	sub	HRAS	c.223G>C	p.G75R	missense	284	0.049
11	61068301	C	A	KARPAS-45	non_MM	sub	DDB1	c.3319G>T	p.E1107*	nonsense	245	0.54
11	61094313	T	C	KARPAS-45	non_MM	sub	DDB1	c.602A>G	p.E201G	missense	369	0.49
11	62563623	C	T	KARPAS-45	non_MM	sub	NXF1	c.1376G>A	p.R459H	missense	322	0.071
11	62563815	C	A	KARPAS-45	non_MM	sub	NXF1	c.1289G>T	p.S430I	missense	343	0.061
12	113531466	G	A	KARPAS-45	non_MM	sub	DTX1	c.1126G>A	p.G376R	missense	273	0.42
12	12022876	C	T	KARPAS-45	non_MM	sub	ETV6	c.982C>T	p.H328Y	missense	120	0.19
12	124817676	C	A	KARPAS-45	non_MM	sub	NCOR2	c.6776G>T	p.S2259I	missense	175	0.21
12	132446413	T	C	KARPAS-45	non_MM	sub	EP400	c.1249T>C	p.Y417H	missense	448	0.11
12	132505701	G	A	KARPAS-45	non_MM	sub	EP400	c.4522G>A	p.A1508T	missense	168	0.5
12	132561107	G	T	KARPAS-45	non_MM	sub	EP400	c.9065G>T	p.S3022I	missense	247	0.065
12	18439812	T	C	KARPAS-45	non_MM	sub	PIK3C2G	c.710T>C	p.V237A	missense	266	0.24
12	40688680	C	T	KARPAS-45	non_MM	sub	LRRK2	c.2842C>T	p.R948*	nonsense	188	0.43
12	40704267	G	C	KARPAS-45	non_MM	sub	LRRK2	c.4352G>C	p.G1451A	missense	223	0.49
12	4383403	T	C	KARPAS-45	non_MM	sub	CCND2	c.195+2T>C	p.?	ess splice	230	0.091

12	4385295	T	C	KARPAS-45	non_MM	sub	CCND2	c.320T>C	p.F107S	missense	231	0.082
12	48368054	G	A	KARPAS-45	non_MM	sub	COL2A1	c.4135C>T	p.R1379C	missense	209	0.091
12	56481806	C	T	KARPAS-45	non_MM	sub	ERBB3	c.734C>T	p.A245V	missense	325	0.052
12	56486791	G	A	KARPAS-45	non_MM	sub	ERBB3	c.1205G>A	p.W402*	nonsense	302	0.076
12	6500019	G	T	KARPAS-45	non_MM	sub	LTBR	c.1224G>T	p.K408N	missense	277	0.22
12	6559401	C	T	KARPAS-45	non_MM	sub	CD27	c.331C>T	p.Q111*	nonsense	205	0.25
13	32906445	A	G	KARPAS-45	non_MM	sub	BRCA2	c.830A>G	p.N277S	missense	275	0.51
13	32914236	C	T	KARPAS-45	non_MM	sub	BRCA2	c.5744C>T	p.T1915M	missense	495	0.484848485
13	92101156	G	T	KARPAS-45	non_MM	sub	GPC5	c.305G>T	p.R102L	missense	278	0.46
14	21860815	A	C	KARPAS-45	non_MM	sub	CHD8	c.5785T>G	p.Y1929D	missense	236	0.25
14	58830888	A	G	KARPAS-45	non_MM	sub	ARID4A	c.2081A>G	p.D694G	missense	176	0.43
14	71199856	C	T	KARPAS-45	non_MM	sub	MAP3K9	c.2272G>A	p.A758T	missense	326	0.058
15	40504699	G	A	KARPAS-45	non_MM	sub	BUB1B	c.2386-1G>A	p.?	ess splice	345	0.12
15	40512907	G	A	KARPAS-45	non_MM	sub	BUB1B	c.3100G>A	p.A1034T	missense	316	0.028481013
15	42003059	C	T	KARPAS-45	non_MM	sub	MGA	c.2596C>T	p.R866*	nonsense	449	0.45
15	42058807	G	A	KARPAS-45	non_MM	sub	MGA	c.8674G>A	p.G2892S	missense	504	0.51
15	67457723	G	A	KARPAS-45	non_MM	sub	SMAD3	c.532+1G>A	p.?	ess splice	213	0.21
15	90630465	C	A	KARPAS-45	non_MM	sub	IDH2	c.846G>T	p.K282N	missense	354	0.076
15	91304297	A	G	KARPAS-45	non_MM	sub	BLM	c.1694A>G	p.D565G	missense	494	0.14
15	91334041	C	T	KARPAS-45	non_MM	sub	BLM	c.2986C>T	p.H996Y	missense	314	0.099
15	91346897	G	A	KARPAS-45	non_MM	sub	BLM	c.3505G>A	p.A1169T	missense	383	0.22
15	91347499	G	T	KARPAS-45	non_MM	sub	BLM	c.3661G>T	p.E1221*	nonsense	442	0.086
15	99440060	C	T	KARPAS-45	non_MM	sub	IGF1R	c.1028C>T	p.S343F	missense	364	0.51
16	3779548	G	A	KARPAS-45	non_MM	sub	CREBBP	c.5500C>T	p.Q1834*	nonsense	304	0.52
16	72828719	T	C	KARPAS-45	non_MM	sub	ZFHX3	c.7862A>G	p.K2621R	missense	283	0.28
16	72993849	C	T	KARPAS-45	non_MM	sub	ZFHX3	c.196G>A	p.A66T	missense	223	0.52
16	8998350	C	A	KARPAS-45	non_MM	sub	USP7	c.1646G>T	p.R549M	missense	184	0.15
16	9009156	C	T	KARPAS-45	non_MM	sub	USP7	c.1033G>A	p.E345K	missense	341	0.13
16	9014262	C	A	KARPAS-45	non_MM	sub	USP7	c.565G>T	p.V189F	missense	407	0.076
16	9017144	C	T	KARPAS-45	non_MM	sub	USP7	c.311G>A	p.R104H	missense	247	0.13
17	11523060	C	T	KARPAS-45	non_MM	sub	DNAH9	c.1312C>T	p.R438*	nonsense	183	0.51
17	11593344	A	G	KARPAS-45	non_MM	sub	DNAH9	c.4205A>G	p.D1402G	missense	324	0.27
17	12032498	A	G	KARPAS-45	non_MM	sub	MAP2K4	c.934A>G	p.S312G	missense	261	0.073
17	15935786	A	G	KARPAS-45	non_MM	sub	NCOR1	c.7147T>C	p.F2383L	missense	143	0.26
17	29654769	C	A	KARPAS-45	non_MM	sub	NF1	c.5521C>A	p.Q1841K	missense	383	0.1
17	37922668	C	T	KARPAS-45	non_MM	sub	IKZF3	c.905G>A	p.R302H	missense	398	0.23
17	3833729	C	T	KARPAS-45	non_MM	sub	ATP2A3	c.2611-1G>A	p.?	ess splice	248	0.073
17	3840839	G	A	KARPAS-45	non_MM	sub	ATP2A3	c.2192C>T	p.A731V	missense	209	0.057
17	3840876	C	T	KARPAS-45	non_MM	sub	ATP2A3	c.2155G>A	p.A719T	missense	179	0.13
17	3844321	C	T	KARPAS-45	non_MM	sub	ATP2A3	c.2044G>A	p.A682T	missense	238	0.21
17	3844949	C	T	KARPAS-45	non_MM	sub	ATP2A3	c.1546-1G>A	p.?	ess splice	150	0.3
17	3848035	C	T	KARPAS-45	non_MM	sub	ATP2A3	c.1250G>A	p.C417Y	missense	226	0.11
17	47685270	G	A	KARPAS-45	non_MM	sub	SPOP	c.680C>T	p.A227V	missense	205	0.46
17	59760659	A	G	KARPAS-45	non_MM	sub	BRIP1	c.3748T>C	p.*1250Q	stop-lost	222	0.095
17	62121353	G	T	KARPAS-45	non_MM	sub	ERN1	c.2929C>A	p.L977I	missense	174	0.49
17	62158169	C	T	KARPAS-45	non_MM	sub	ERN1	c.176-1G>A	p.?	ess splice	285	0.06
17	70117896	A	C	KARPAS-45	non_MM	sub	SOX9	c.364A>C	p.K122Q	missense	142	0.22
17	7577121	G	A	KARPAS-45	non_MM	sub	TP53	c.817C>T	p.R273C	missense	242	0.479338843
17	7578406	C	T	KARPAS-45	non_MM	sub	TP53	c.524G>A	p.R175H	missense	280	0.48
17	7579313	G	A	KARPAS-45	non_MM	sub	TP53	c.374C>T	p.T125M	missense	145	0.082758621
17	7749937	T	C	KARPAS-45	non_MM	sub	KDM6B	c.590T>C	p.V197A	missense	258	0.28
17	7751546	G	A	KARPAS-45	non_MM	sub	KDM6B	c.1940G>A	p.G647D	missense	270	0.21
17	78327398	G	T	KARPAS-45	non_MM	sub	RNF213	c.4729G>T	p.E1577*	nonsense	202	0.084
17	78341569	G	A	KARPAS-45	non_MM	sub	RNF213	c.6112G>A	p.V2038M	missense	309	0.52
17	79477969	C	A	KARPAS-45	non_MM	sub	ACTG1	c.968G>T	p.S323I	missense	217	0.092
17	79478564	A	G	KARPAS-45	non_MM	sub	ACTG1	c.452T>C	p.I151T	missense	275	0.5
17	79479302	G	T	KARPAS-45	non_MM	sub	ACTG1	c.79C>A	p.P27T	missense	254	0.075
18	19751259	C	T	KARPAS-45	non_MM	sub	GATA6	c.154C>T	p.R52W	missense	294	0.47
18	72998423	G	A	KARPAS-45	non_MM	sub	TSHZ1	c.926G>A	p.R309Q	missense	236	0.5
19	15290294	T	C	KARPAS-45	non_MM	sub	NOTCH3	c.3341A>G	p.Y1114C	missense	291	0.21
19	15302905	C	T	KARPAS-45	non_MM	sub	NOTCH3	c.545G>A	p.R182H	missense	284	0.25
19	4102376	C	T	KARPAS-45	non_MM	sub	MAP2K2	c.526G>A	p.A176T	missense	214	0.4
19	4110526	C	A	KARPAS-45	non_MM	sub	MAP2K2	c.431G>T	p.S144I	missense	365	0.23
19	42383293	G	A	KARPAS-45	non_MM	sub	CD79A	c.313G>A	p.V105M	missense	311	0.28
19	47177813	C	T	KARPAS-45	non_MM	sub	PRKD2	c.2604G>A	p.M868I	missense	262	0.26
2	102507646	C	T	KARPAS-45	non_MM	sub	MAP4K4	c.3898C>T	p.R1300W	missense	181	0.28
2	141986892	G	A	KARPAS-45	non_MM	sub	LRP1B	c.710C>T	p.T237I	missense	248	0.29
2	152426672	A	G	KARPAS-45	non_MM	sub	NEB	c.12250T>C	p.Y4084H	missense	180	0.29
2	152512752	T	C	KARPAS-45	non_MM	sub	NEB	c.6410A>G	p.Y2137C	missense	228	0.34
2	178096504	G	A	KARPAS-45	non_MM	sub	NFE2L2	c.827C>T	p.T276I	missense	364	0.32
2	25471040	C	A	KARPAS-45	non_MM	sub	DNMT3A	c.721G>T	p.E241*	nonsense	81	0.25
2	29416573	C	A	KARPAS-45	non_MM	sub	ALK	c.4380G>T	p.E1460D	missense	263	0.34
2	61706076	T	A	KARPAS-45	non_MM	sub	XPO1	c.3095A>T	p.D1032V	missense	247	0.032388664
20	22563629	G	A	KARPAS-45	non_MM	sub	FOXA2	c.251C>T	p.A84V	missense	232	0.25
20	41101119	C	T	KARPAS-45	non_MM	sub	PTPRT	c.1237G>A	p.A413T	missense	229	0.59
21	44514778	G	A	KARPAS-45	non_MM	sub	U2AF1	c.469C>T	p.Q157*	nonsense	332	0.23
22	21104218	G	T	KARPAS-45	non_MM	sub	PI4KA	c.3044C>A	p.P1015H	missense	140	0.086
22	24167480	C	A	KARPAS-45	non_MM	sub	SMARCB1	c.864C>A	p.N288K	missense	251	0.46
3	138433507	C	T	KARPAS-45	non_MM	sub	PIK3CB	c.1105G>A	p.V369I	missense	275	0.31

3	141231095	G	A	KARPAS-45	non_MM	sub	RASA2	c.224G>A	p.R75H	missense	303	0.63
3	141327331	A	G	KARPAS-45	non_MM	sub	RASA2	c.2017A>G	p.M673V	missense	168	0.35
3	142215353	G	T	KARPAS-45	non_MM	sub	ATR	c.5748C>A	p.Y1916*	nonsense	190	0.079
3	170841384	C	T	KARPAS-45	non_MM	sub	TNIK	c.2120+1G>A	p.?	ess splice	127	0.14
3	170843927	A	G	KARPAS-45	non_MM	sub	TNIK	c.1787T>C	p.V596A	missense	106	0.26
3	178917489	G	A	KARPAS-45	non_MM	sub	PIK3CA	c.364G>A	p.G122S	missense	191	0.32
3	47098564	C	T	KARPAS-45	non_MM	sub	SETD2	c.6710G>A	p.S2237N	missense	201	0.1
3	52595799	C	A	KARPAS-45	non_MM	sub	PBRM1	c.4176G>T	p.K1392N	missense	166	0.084
3	78685048	A	G	KARPAS-45	non_MM	sub	ROBO1	c.3131T>C	p.L1044P	missense	211	0.33
3	78688924	C	T	KARPAS-45	non_MM	sub	ROBO1	c.2890G>A	p.D964N	missense	86	0.36
3	89457249	A	G	KARPAS-45	non_MM	sub	EPHA3	c.1730A>G	p.D577G	missense	239	0.35
3	8955369	T	A	KARPAS-45	non_MM	sub	RAD18	c.925A>T	p.K309*	nonsense	241	0.32
4	126370690	G	A	KARPAS-45	non_MM	sub	FAT4	c.8519G>A	p.R2840H	missense	292	0.2
4	126411835	T	C	KARPAS-45	non_MM	sub	FAT4	c.13858T>C	p.Y4620H	missense	264	0.27
4	153247289	G	A	KARPAS-45	non_MM	sub	FBXW7	c.1513C>T	p.R505C	missense	303	0.65
4	175897971	C	T	KARPAS-45	non_MM	sub	ADAM29	c.1295C>T	p.T432I	missense	257	0.34
4	187510360	T	C	KARPAS-45	non_MM	sub	FAT1	c.13153A>G	p.T4385A	missense	105	0.076
4	187524399	T	C	KARPAS-45	non_MM	sub	FAT1	c.11281A>G	p.T3761A	missense	155	0.15
4	187524995	C	T	KARPAS-45	non_MM	sub	FAT1	c.10685G>A	p.G3562D	missense	243	0.11
4	187525064	C	T	KARPAS-45	non_MM	sub	FAT1	c.10616G>A	p.S3539N	missense	241	0.14
4	187549484	A	G	KARPAS-45	non_MM	sub	FAT1	c.4634T>C	p.F1545S	missense	183	0.28
4	1918621	T	C	KARPAS-45	non_MM	sub	WHSC1	c.784T>C	p.Y262H	missense	452	0.25
4	55961055	C	T	KARPAS-45	non_MM	sub	KDR	c.2885G>A	p.R962H	missense	253	0.53
5	112177617	A	G	KARPAS-45	non_MM	sub	APC	c.6326A>G	p.N2109S	missense	397	0.055
5	112177872	T	C	KARPAS-45	non_MM	sub	APC	c.6581T>C	p.V2194A	missense	380	0.23
5	13752344	G	T	KARPAS-45	non_MM	sub	DNAH5	c.10927C>A	p.L3643I	missense	233	0.39
5	137802832	G	A	KARPAS-45	non_MM	sub	EGR1	c.694G>A	p.A232T	missense	306	0.12
5	137803735	G	T	KARPAS-45	non_MM	sub	EGR1	c.1597G>T	p.A533S	missense	245	0.082
5	149502651	C	T	KARPAS-45	non_MM	sub	PDGFRB	c.2137G>A	p.A713T	missense	319	0.6
5	149513252	G	T	KARPAS-45	non_MM	sub	PDGFRB	c.831C>A	p.H277Q	missense	255	0.51
5	150923606	C	G	KARPAS-45	non_MM	sub	FAT2	c.7082G>C	p.G2361A	missense	329	0.53
5	66460423	G	T	KARPAS-45	non_MM	sub	MAST4	c.4849G>T	p.D1617Y	missense	265	0.11
5	66462230	C	T	KARPAS-45	non_MM	sub	MAST4	c.6656C>T	p.A2219V	missense	330	0.48
6	106552838	G	A	KARPAS-45	non_MM	sub	PRDM1	c.803G>A	p.R268H	missense	514	0.59
6	106555015	C	T	KARPAS-45	non_MM	sub	PRDM1	c.2132C>T	p.A711V	missense	461	0.2
6	114270421	T	C	KARPAS-45	non_MM	sub	HDAC2	c.925A>G	p.I309V	missense	400	0.44
6	117725565	A	G	KARPAS-45	non_MM	sub	ROS1	c.316T>C	p.F106L	missense	378	0.37
6	160464302	G	A	KARPAS-45	non_MM	sub	IGF2R	c.1603G>A	p.A535T	missense	311	0.061
6	161455467	G	A	KARPAS-45	non_MM	sub	MAP3K4	c.329G>A	p.R110Q	missense	312	0.62
6	32166763	C	A	KARPAS-45	non_MM	sub	NOTCH4	c.4475G>T	p.R1492L	missense	362	0.046961326
6	41903806	G	T	KARPAS-45	non_MM	sub	CCND3	c.751C>A	p.L251I	missense	243	0.23
6	45459768	T	C	KARPAS-45	non_MM	sub	RUNX2	c.980T>C	p.V327A	missense	329	0.21
6	51774167	A	G	KARPAS-45	non_MM	sub	PKHD1	c.6596T>C	p.V2199A	missense	397	0.071
7	121698987	T	A	KARPAS-45	non_MM	sub	PTPRZ1	c.6662T>A	p.V2221D	missense	281	0.078
7	148526829	C	T	KARPAS-45	non_MM	sub	EZH2	c.475G>A	p.G159R	missense	275	0.44
7	21695478	A	G	KARPAS-45	non_MM	sub	DNAH11	c.4988A>G	p.D1663G	missense	307	0.068
7	21726869	A	G	KARPAS-45	non_MM	sub	DNAH11	c.5795A>G	p.Y1932C	missense	257	0.51
7	50444234	G	A	KARPAS-45	non_MM	sub	IKZF1	c.164G>A	p.S55N	missense	287	0.13
7	55210117	T	C	KARPAS-45	non_MM	sub	EGFR	c.227T>C	p.L76P	missense	247	0.48
7	82390800	T	C	KARPAS-45	non_MM	sub	PCLO	c.15017A>G	p.Q5006R	missense	197	0.47
7	82595436	T	A	KARPAS-45	non_MM	sub	PCLO	c.3668A>T	p.E1223V	missense	363	0.088
7	82785230	G	A	KARPAS-45	non_MM	sub	PCLO	c.727C>T	p.Q243*	nonsense	302	0.24
7	92247491	C	T	KARPAS-45	non_MM	sub	CDK6	c.729G>A	p.W243*	nonsense	326	0.071
8	128750966	A	G	KARPAS-45	non_MM	sub	MYC	c.503A>G	p.Q168R	missense	323	0.22
8	77765405	T	C	KARPAS-45	non_MM	sub	ZFHX4	c.6113T>C	p.M2038T	missense	141	0.23
8	77767027	T	A	KARPAS-45	non_MM	sub	ZFHX4	c.7735T>A	p.Y2579N	missense	391	0.24
9	139391719	C	T	KARPAS-45	non_MM	sub	NOTCH1	c.6472G>A	p.V2158I	missense	337	0.2
9	139391892	A	G	KARPAS-45	non_MM	sub	NOTCH1	c.6299T>C	p.I2100T	missense	286	0.34
9	139393398	C	T	KARPAS-45	non_MM	sub	NOTCH1	c.6133G>A	p.V2045I	missense	278	0.2
9	139395053	C	T	KARPAS-45	non_MM	sub	NOTCH1	c.5885G>A	p.R1962H	missense	281	0.39
9	139395161	C	T	KARPAS-45	non_MM	sub	NOTCH1	c.5777G>A	p.R1926H	missense	317	0.05
9	139396284	C	T	KARPAS-45	non_MM	sub	NOTCH1	c.5554G>A	p.D1852N	missense	229	0.17
9	139397675	A	G	KARPAS-45	non_MM	sub	NOTCH1	c.5126T>C	p.L1709P	missense	230	0.33
9	139397775	C	T	KARPAS-45	non_MM	sub	NOTCH1	c.5026G>A	p.V1676I	missense	197	0.19
9	139818415	C	T	KARPAS-45	non_MM	sub	TRAF2	c.1250C>T	p.P417L	missense	159	0.69
9	21971120	G	A	KARPAS-45	non_MM	sub	CDKN2A	c.238C>T	p.R80*	nonsense	230	0.991304348
9	8317920	C	T	KARPAS-45	non_MM	sub	PTPRD	c.5693G>A	p.R1898H	missense	186	0.28
9	8319926	T	C	KARPAS-45	non_MM	sub	PTPRD	c.5575A>G	p.I1859V	missense	168	0.64
9	87339190	G	A	KARPAS-45	non_MM	sub	NTRK2	c.772G>A	p.D258N	missense	185	0.69
9	87342797	T	C	KARPAS-45	non_MM	sub	NTRK2	c.1082T>C	p.L361P	missense	277	0.12
X	41203649	G	A	KARPAS-45	non_MM	sub	DDX3X	c.1022G>A	p.C341Y	missense	180	0.48
X	53224108	C	T	KARPAS-45	non_MM	sub	KDM5C	c.3438+5G>A	p.?	ess splice	111	0.099
X	70341238	G	A	KARPAS-45	non_MM	sub	MED12	c.797G>A	p.R266H	missense	180	0.23
X	70348159	G	A	KARPAS-45	non_MM	sub	MED12	c.3223G>A	p.A1075T	missense	191	0.49
X	70352988	C	T	KARPAS-45	non_MM	sub	MED12	c.4543C>T	p.R1515*	nonsense	182	0.24
X	70360544	C	T	KARPAS-45	non_MM	sub	MED12	c.6104C>T	p.A2035V	missense	130	0.45
13	95273424	A	G	KASUMI-1	non_MM	sub	GPR180	c.829A>G	p.R277G	missense	270	0.43
15	74836779	G	C	KASUMI-1	non_MM	sub	ARID3B	c.502G>C	p.V168L	missense	356	0.412921348
15	99251007	C	T	KASUMI-1	non_MM	sub	IGF1R	c.311C>T	p.T104M	missense	287	0.47

17	7577538	C	T	KASUMI-1	non_MM	sub	TP53	c.743G>A	p.R248Q	missense	88	1
19	1612406	T	A	KASUMI-1	non_MM	sub	TCF3	c.1613A>T	p.E538V	missense	203	0.5
4	175899083	C	T	KASUMI-1	non_MM	sub	ADAM29	c.2407C>T	p.Q803*	nonsense	253	0.3
6	117642495	C	T	KASUMI-1	non_MM	sub	ROS1	c.5704G>A	p.E1902K	missense	355	0.523943662
7	21658769	C	T	KASUMI-1	non_MM	sub	DNAH11	c.4321C>T	p.R1441W	missense	387	0.542635659
7	21698468	A	G	KASUMI-1	non_MM	sub	DNAH11	c.5162A>G	p.H1721R	missense	193	0.5
1	43818443	A	G	KMS-11	MM	sub	MPL	c.1908A>G	p.*636W	stop-lost	72	0.61
11	119148988	C	T	KMS-11	MM	sub	CBL	c.1208C>T	p.S403F	missense	98	0.082
12	124824918	G	A	KMS-11	MM	sub	NCOR2	c.5431C>T	p.R1811W	missense	105	0.49
12	31253975	C	T	KMS-11	MM	sub	DDX11	c.1963C>T	p.P655S	missense	105	0.057142857
17	62157063	T	C	KMS-11	MM	sub	ERN1	c.241A>G	p.S81G	missense	177	0.2
19	15295205	G	T	KMS-11	MM	sub	NOTCH3	c.2467C>A	p.H823N	missense	102	0.31
19	31770405	C	A	KMS-11	MM	sub	TSHZ3	c.294G>T	p.E98D	missense	90	0.19
2	102482984	C	T	KMS-11	MM	sub	MAP4K4	c.2299C>T	p.Q767*	nonsense	55	0.31
2	141299461	C	T	KMS-11	MM	sub	LRP1B	c.7274G>A	p.R2425K	missense	72	0.47
2	152347020	C	T	KMS-11	MM	sub	NEB	c.19595G>A	p.R6532H	missense	218	0.62
2	152373056	C	T	KMS-11	MM	sub	NEB	c.17719G>A	p.E5907K	missense	242	0.11
2	152422269	G	C	KMS-11	MM	sub	NEB	c.13119C>G	p.D4373E	missense	250	0.4
2	152512444	C	T	KMS-11	MM	sub	NEB	c.6589G>A	p.E2197K	missense	220	0.22
4	1806099	A	G	KMS-11	MM	sub	FGFR3	c.1118A>G	p.Y373C	missense	111	0.45
4	187584654	T	C	KMS-11	MM	sub	FAT1	c.3379A>G	p.I1127V	missense	202	0.52
4	187628521	C	A	KMS-11	MM	sub	FAT1	c.2461G>T	p.E821*	nonsense	254	0.15
5	112176660	G	A	KMS-11	MM	sub	APC	c.5369G>A	p.R1790K	missense	183	0.34
5	149515232	C	T	KMS-11	MM	sub	PDGFRB	c.250G>A	p.V84M	missense	69	0.97
5	66459026	C	T	KMS-11	MM	sub	MAST4	c.3452C>T	p.P1151L	missense	196	0.12
7	21784584	G	A	KMS-11	MM	sub	DNAH11	c.8434G>A	p.E2812K	missense	233	0.06
7	82435118	T	C	KMS-11	MM	sub	PCLO	c.14819A>G	p.E4940G	missense	77	0.091
9	8485274	G	C	KMS-11	MM	sub	PTPRD	c.3106C>G	p.L1036V	missense	145	0.29
11	92533998	G	A	KMS-12-BM	MM	sub	FAT3	c.7819G>A	p.A2607T	missense	216	0.17
12	31244771	C	T	KMS-12-BM	MM	sub	DDX11	c.1208C>T	p.T403M	missense	195	0.41
12	430171	C	T	KMS-12-BM	MM	sub	KDM5A	c.2531G>A	p.R844Q	missense	128	0.42
15	91326152	C	A	KMS-12-BM	MM	sub	BLM	c.2656C>A	p.H886N	missense	218	0.48
3	10108987	A	C	KMS-12-BM	MM	sub	FANCD2	c.2480A>C	p.E827A	missense	134	0.46
3	89456423	C	A	KMS-12-BM	MM	sub	EPHA3	c.1599C>A	p.F533L	missense	158	0.54
6	31549372	C	G	KMS-12-BM	MM	sub	LTB	c.244G>C	p.D82H	missense	68	0.57
6	31549398	T	C	KMS-12-BM	MM	sub	LTB	c.218A>G	p.K73R	missense	56	0.62
7	82764569	A	T	KMS-12-BM	MM	sub	PCLO	c.2297T>A	p.L766H	missense	180	0.46
7	82784113	G	T	KMS-12-BM	MM	sub	PCLO	c.1844C>A	p.T615N	missense	144	0.46
1	115256528	T	G	L-363	MM	sub	NRAS	c.183A>C	p.Q61H	missense	109	0.47
1	27057949	C	G	L-363	MM	sub	ARID1A	c.1657C>G	p.Q553E	missense	70	0.4
12	56487289	G	A	L-363	MM	sub	ERBB3	c.1435G>A	p.E479K	missense	71	0.52
15	99482568	G	A	L-363	MM	sub	IGF1R	c.3436G>A	p.D1146N	missense	150	0.68
16	3828071	A	C	L-363	MM	sub	CREBBP	c.2054T>G	p.L685*	nonsense	73	0.096
16	89809206	G	A	L-363	MM	sub	FANCA	c.3765+2C>T	p.?	ess splice	73	0.51
16	8994457	G	A	L-363	MM	sub	USP7	c.2239C>T	p.Q747*	nonsense	101	0.47
17	78351591	G	T	L-363	MM	sub	RNF213	c.7759G>T	p.G2587*	nonsense	35	0.23
17	78360674	C	G	L-363	MM	sub	RNF213	c.9124C>G	p.P3042A	missense	31	0.23
20	29633907	T	A	L-363	MM	sub	FRG1B	c.546T>A	p.N182K	missense	603	0.035
3	178936091	G	A	L-363	MM	sub	PIK3CA	c.1633G>A	p.E545K	missense	157	0.45
3	78649435	G	T	L-363	MM	sub	ROBO1	c.4652C>A	p.P1551H	missense	101	0.49
6	32163297	C	T	L-363	MM	sub	NOTCH4	c.5929G>A	p.E1977K	missense	79	0.63
7	21628186	G	A	L-363	MM	sub	DNAH11	c.1905G>A	p.M635I	missense	150	0.31
7	82582600	G	T	L-363	MM	sub	PCLO	c.7669C>A	p.P2557T	missense	171	0.32
11	69456169	G	A	LP-1	MM	sub	CCND1	c.88G>A	p.A30T	missense	139	0.55
16	72831992	C	A	LP-1	MM	sub	ZFHX3	c.4589G>T	p.G1530V	missense	156	0.49
2	141643868	T	C	LP-1	MM	sub	LRP1B	c.3803A>G	p.H1268R	missense	86	0.91
2	202137633	G	A	LP-1	MM	sub	CASP8	c.740G>A	p.S247N	missense	138	0.4
20	40713407	G	A	LP-1	MM	sub	PTPRT	c.4051C>T	p.R1351C	missense	117	0.3
5	13769721	C	T	LP-1	MM	sub	DNAH5	c.9609G>A	p.M3203I	missense	130	0.28
6	157522211	C	T	LP-1	MM	sub	ARID1B	c.4429C>T	p.R1477C	missense	88	0.84
7	21630542	G	A	LP-1	MM	sub	DNAH11	c.2176G>A	p.A726T	missense	103	0.53
X	44922760	C	T	LP-1	MM	sub	KDM6A	c.1621C>T	p.Q541*	nonsense	65	0.89
10	104160777	C	T	MC-CAR	MM	sub	NFKB2	c.2042C>T	p.P681L	missense	76	0.58
11	533874	T	C	MC-CAR	MM	sub	HRAS	c.182A>G	p.Q61R	missense	161	0.43
16	89838148	C	T	MC-CAR	MM	sub	FANCA	c.2089G>A	p.V697I	missense	145	0.49
19	1627422	T	C	MC-CAR	MM	sub	TCF3	c.302A>G	p.K101R	missense	94	0.521276596
4	106164897	C	A	MC-CAR	MM	sub	TET2	c.3765C>A	p.Y1255*	nonsense	70	0.4
5	13716742	C	A	MC-CAR	MM	sub	DNAH5	c.12763G>T	p.V4255F	missense	148	0.47
6	31549637	C	T	MC-CAR	MM	sub	LTB	c.163-1G>A	p.?	ess splice	122	0.54
7	21657328	C	G	MC-CAR	MM	sub	DNAH11	c.4202C>G	p.A1401G	missense	125	0.44
1	118166298	A	G	MM1S	MM	sub	FAM46C	c.808A>G	p.M270V	missense	75	1
12	25398284	C	G	MM1S	MM	sub	KRAS	c.35G>C	p.G12A	missense	132	0.45
15	42046714	A	G	MM1S	MM	sub	MGA	c.7235A>G	p.N2412S	missense	117	0.44
17	62125301	C	A	MM1S	MM	sub	ERN1	c.2446G>T	p.D816Y	missense	159	0.056603774
19	42798179	C	G	MM1S	MM	sub	CIC	c.4133C>G	p.S1378C	missense	128	0.21
3	77657042	C	G	MM1S	MM	sub	ROBO2	c.3230C>G	p.P1077R	missense	92	0.51
4	1962801	G	A	MM1S	MM	sub	WHSC1	c.3295G>A	p.E1099K	missense	132	0.55
6	31549377	T	C	MM1S	MM	sub	LTB	c.239A>G	p.E80G	missense	54	0.56
7	55266457	G	C	MM1S	MM	sub	EGFR	c.2749G>C	p.G917R	missense	136	0.43

9	139794917	C	T	MM1S	MM	sub	TRAF2	c.311C>T	p.P104L	missense	76	0.28
1	120471739	C	T	MN-60	non_MM	sub	NOTCH2	c.3752G>A	p.R1251H	missense	140	0.45
1	154574829	C	T	MN-60	non_MM	sub	ADAR	c.289G>A	p.V97M	missense	328	0.33
1	23885728	G	A	MN-60	non_MM	sub	ID3	c.190C>T	p.L64F	missense	113	1
1	27105553	C	T	MN-60	non_MM	sub	ARID1A	c.5164C>T	p.R1722*	nonsense	164	0.46
10	96076338	C	T	MN-60	non_MM	sub	PLCE1	c.6167C>T	p.T2056I	missense	197	0.32
11	70331800	T	A	MN-60	non_MM	sub	SHANK2	c.4580A>T	p.Y1527F	missense	189	0.058
11	92533581	A	G	MN-60	non_MM	sub	FAT3	c.7402A>G	p.N2468D	missense	188	0.41
12	18466970	G	T	MN-60	non_MM	sub	PIK3C2G	c.1109G>T	p.R370M	missense	213	0.36
13	73335914	G	A	MN-60	non_MM	sub	DIS3	c.2381C>T	p.A794V	missense	352	0.2
15	41988349	A	G	MN-60	non_MM	sub	MGA	c.1141A>G	p.I381V	missense	257	0.49
15	91347446	C	T	MN-60	non_MM	sub	BLM	c.3608C>T	p.A1203V	missense	200	0.42
17	41244703	C	A	MN-60	non_MM	sub	BRCA1	c.2845G>T	p.G949C	missense	327	0.39
19	31767689	G	A	MN-60	non_MM	sub	TSHZ3	c.3010C>T	p.R1004W	missense	221	0.43
2	152383461	C	T	MN-60	non_MM	sub	NEB	c.16813G>A	p.A5605T	missense	203	0.45
2	152507108	C	T	MN-60	non_MM	sub	NEB	c.7207G>A	p.V2403I	missense	211	0.43
20	44757522	C	G	MN-60	non_MM	sub	CD40	c.677C>G	p.A226G	missense	122	0.48
20	57415192	C	T	MN-60	non_MM	sub	GNAS	c.31C>T	p.R11C	missense	184	0.41
3	187446979	G	T	MN-60	non_MM	sub	BCL6	c.1214C>A	p.P405Q	missense	171	0.32
3	37056036	G	A	MN-60	non_MM	sub	MLH1	c.790+1G>A	p.?	ess splice	158	0.98
3	78680332	T	C	MN-60	non_MM	sub	ROBO1	c.3488A>G	p.Y1163C	missense	123	0.3
3	78683102	C	T	MN-60	non_MM	sub	ROBO1	c.3347G>A	p.R1116Q	missense	175	0.41
4	103528412	G	A	MN-60	non_MM	sub	NFKB1	c.2060G>A	p.R687H	missense	129	0.52
4	126370746	A	G	MN-60	non_MM	sub	FAT4	c.8575A>G	p.T2859A	missense	257	0.082
4	1807533	C	T	MN-60	non_MM	sub	FGFR3	c.1702C>T	p.R568W	missense	218	0.4
4	187509894	T	C	MN-60	non_MM	sub	FAT1	c.13619A>G	p.Y4540C	missense	180	0.094
4	187629487	T	C	MN-60	non_MM	sub	FAT1	c.1495A>G	p.T499A	missense	295	0.38
4	1918645	G	A	MN-60	non_MM	sub	WHSC1	c.808G>A	p.A270T	missense	327	0.42
5	13708249	G	A	MN-60	non_MM	sub	DNAH5	c.13321C>T	p.P4441S	missense	129	0.41
5	150922248	T	C	MN-60	non_MM	sub	FAT2	c.8440A>G	p.T2814A	missense	193	0.45
5	179676027	T	A	MN-60	non_MM	sub	MAPK9	c.562A>T	p.T188S	missense	208	0.048
5	66459305	C	T	MN-60	non_MM	sub	MAST4	c.3731C>T	p.A1244V	missense	251	0.39
6	117678064	G	A	MN-60	non_MM	sub	ROS1	c.3869C>T	p.T1290I	missense	71	0.76
6	15513159	G	A	MN-60	non_MM	sub	JARID2	c.3149G>A	p.R1050H	missense	197	0.41
6	157454280	C	A	MN-60	non_MM	sub	ARID1B	c.2277C>A	p.S759R	missense	222	0.5
6	161491676	T	C	MN-60	non_MM	sub	MAP3K4	c.1744T>C	p.Y582H	missense	78	0.09
7	121651238	A	G	MN-60	non_MM	sub	PTPRZ1	c.2138A>G	p.Y713C	missense	247	0.47
7	82595791	G	A	MN-60	non_MM	sub	PCLO	c.3313C>T	p.L1105F	missense	111	0.37
8	128750844	C	G	MN-60	non_MM	sub	MYC	c.381C>G	p.N127K	missense	262	0.58
8	128750921	T	C	MN-60	non_MM	sub	MYC	c.458T>C	p.F153S	missense	266	0.52
8	77616672	A	C	MN-60	non_MM	sub	ZFHX4	c.349A>C	p.S117R	missense	265	0.41
9	21971120	G	A	MN-60	non_MM	sub	CDKN2A	c.238C>T	p.R80*	nonsense	213	0.990610329
9	87425481	C	T	MN-60	non_MM	sub	NTRK2	c.1421C>T	p.P474L	missense	111	0.33
1	115258744	C	T	NCI-H929	MM	sub	NRAS	c.38G>A	p.G13D	missense	150	0.52
11	108117828	G	A	NCI-H929	MM	sub	ATM	c.1039G>A	p.E347K	missense	296	0.28
12	459807	T	C	NCI-H929	MM	sub	KDM5A	c.1288A>G	p.K430E	missense	107	0.5
13	32937341	A	G	NCI-H929	MM	sub	BRCA2	c.8002A>G	p.R2668G	missense	53	1
15	42059075	C	G	NCI-H929	MM	sub	MGA	c.8942C>G	p.S2981C	missense	164	0.61
19	47193921	A	G	NCI-H929	MM	sub	PRKD2	c.1745T>C	p.I582T	missense	59	0.61
2	61761029	G	C	NCI-H929	MM	sub	XPO1	c.4C>G	p.P2A	missense	158	0.4
6	117622256	C	A	NCI-H929	MM	sub	ROS1	c.6614G>T	p.R2205I	missense	117	0.5
6	51523918	G	A	NCI-H929	MM	sub	PKHD1	c.11006C>T	p.S3669L	missense	121	0.53
7	121651510	G	A	NCI-H929	MM	sub	PTPRZ1	c.2410G>A	p.D804N	missense	150	0.51
X	39916496	C	T	NCI-H929	MM	sub	BCOR	c.4507G>A	p.E1503K	missense	65	0.65
1	118166023	A	C	OPM-2	MM	sub	FAM46C	c.533A>C	p.E178A	missense	58	0.98
11	92531941	G	T	OPM-2	MM	sub	FAT3	c.5762G>T	p.S1921I	missense	180	0.23
12	124817000	T	C	OPM-2	MM	sub	NCOR2	c.6790A>G	p.K2264E	missense	96	0.49
13	73355008	T	G	OPM-2	MM	sub	DIS3	c.362A>C	p.Y121S	missense	81	1
17	11865517	C	T	OPM-2	MM	sub	DNAH9	c.13177C>T	p.R4393W	missense	129	0.52
17	16049706	G	C	OPM-2	MM	sub	NCOR1	c.1066C>G	p.Q356E	missense	194	0.18
17	7578406	C	T	OPM-2	MM	sub	TP53	c.524G>A	p.R175H	missense	62	0.97
2	141115674	A	G	OPM-2	MM	sub	LRP1B	c.11269T>C	p.Y3757H	missense	114	0.13
2	97215067	G	A	OPM-2	MM	sub	ARID5A	c.130G>A	p.E44K	missense	55	0.69
20	31022469	G	A	OPM-2	MM	sub	ASXL1	c.1954G>A	p.G652S	missense	129	0.643410853
3	193201748	G	A	OPM-2	MM	sub	ATP13A4	c.785C>T	p.T262M	missense	164	0.69
4	126402711	A	G	OPM-2	MM	sub	FAT4	c.12634A>G	p.K4212E	missense	180	0.17
4	1807889	A	G	OPM-2	MM	sub	FGFR3	c.1948A>G	p.K650E	missense	168	0.49
9	21971111	G	A	OPM-2	MM	sub	CDKN2A	c.247C>T	p.H83Y	missense	115	0.3
1	16255925	A	G	RPMI-8226	MM	sub	SPEN	c.3190A>G	p.K1064E	missense	201	0.43
1	45797105	C	T	RPMI-8226	MM	sub	MUTYH	c.1310G>A	p.R437Q	missense	119	0.58
12	25398284	C	G	RPMI-8226	MM	sub	KRAS	c.35G>C	p.G12A	missense	118	0.67
12	40697769	A	G	RPMI-8226	MM	sub	LRRK2	c.3610A>G	p.S1204G	missense	89	0.37
12	4409098	C	T	RPMI-8226	MM	sub	CCND2	c.793C>T	p.Q265*	nonsense	106	0.27
17	7577085	C	T	RPMI-8226	MM	sub	TP53	c.853G>A	p.E285K	missense	62	0.983870968
2	152507259	C	G	RPMI-8226	MM	sub	NEB	c.7056G>C	p.K2352N	missense	190	0.28
21	44527564	T	C	RPMI-8226	MM	sub	U2AF1	c.41A>G	p.D14G	missense	109	0.17
6	31549590	C	T	RPMI-8226	MM	sub	LTB	c.208+1G>A	p.?	ess splice	131	0.65
6	31549591	C	T	RPMI-8226	MM	sub	LTB	c.208G>A	p.G70R	missense	128	0.66
6	395889	G	A	RPMI-8226	MM	sub	IRF4	c.446G>A	p.S149N	missense	118	0.330508475

7	55242482	C	T	RPMI-8226	MM	sub	EGFR	c.2252C>T	p.T751I	missense	192	0.26
7	82581655	C	T	RPMI-8226	MM	sub	PCLO	c.8614G>A	p.V2872I	missense	214	0.39
9	139391079	G	C	RPMI-8226	MM	sub	NOTCH1	c.7112C>G	p.T2371S	missense	167	0.5
10	104156071	A	C	RPMI-8866	non_MM	sub	NFKB2	c.83A>C	p.K28T	missense	174	0.48
11	102196238	C	T	RPMI-8866	non_MM	sub	BIRC3	c.895C>T	p.L299F	missense	216	0.47
17	29684329	A	G	RPMI-8866	non_MM	sub	NF1	c.7912A>G	p.I2638V	missense	207	0.49
17	78320643	G	T	RPMI-8866	non_MM	sub	RNF213	c.2727G>T	p.Q909H	missense	133	0.5
2	140997075	C	T	RPMI-8866	non_MM	sub	LRP1B	c.13351G>A	p.V4451I	missense	184	0.45
2	141739747	C	A	RPMI-8866	non_MM	sub	LRP1B	c.2869G>T	p.D957Y	missense	202	0.44
2	152387587	G	T	RPMI-8866	non_MM	sub	NEB	c.16448C>A	p.A5483D	missense	195	0.44
2	152515585	C	T	RPMI-8866	non_MM	sub	NEB	c.6069G>A	p.M2023I	missense	282	0.489361702
2	152536299	T	C	RPMI-8866	non_MM	sub	NEB	c.3191A>G	p.Y1064C	missense	150	0.493333333
22	21088073	G	A	RPMI-8866	non_MM	sub	PI4KA	c.3937C>T	p.R1313C	missense	146	0.48
3	193175245	A	T	RPMI-8866	non_MM	sub	ATP13A4	c.1684T>A	p.F562I	missense	252	0.31
3	77614164	C	A	RPMI-8866	non_MM	sub	ROBO2	c.1742C>A	p.T581N	missense	232	0.49
4	126239529	T	C	RPMI-8866	non_MM	sub	FAT4	c.1963T>C	p.Y655H	missense	178	0.44
4	175897949	T	C	RPMI-8866	non_MM	sub	ADAM29	c.1273T>C	p.C425R	missense	242	0.48
4	187629135	C	G	RPMI-8866	non_MM	sub	FAT1	c.1847G>C	p.S616T	missense	217	0.5
4	55948163	T	G	RPMI-8866	non_MM	sub	KDR	c.3808A>C	p.K1270Q	missense	177	0.44
5	112179398	A	G	RPMI-8866	non_MM	sub	APC	c.8107A>G	p.K2703E	missense	242	0.49
5	13920635	C	T	RPMI-8866	non_MM	sub	DNAH5	c.752G>A	p.G251E	missense	234	0.48
5	23526849	T	C	RPMI-8866	non_MM	sub	PRDM9	c.1652T>C	p.L551P	missense	311	0.048
5	66396399	G	A	RPMI-8866	non_MM	sub	MAST4	c.482G>A	p.R161H	missense	151	0.55
6	26056599	C	T	RPMI-8866	non_MM	sub	HIST1H1C	c.58G>A	p.V20I	missense	130	0.57
6	26157099	C	T	RPMI-8866	non_MM	sub	HIST1H1E	c.481C>T	p.P161S	missense	131	0.48
7	82785518	C	T	RPMI-8866	non_MM	sub	PCLO	c.439G>A	p.E147K	missense	212	0.49
X	41205841	T	A	RPMI-8866	non_MM	sub	DDX3X	c.1581T>A	p.H527Q	missense	202	0.52
12	124862822	C	T	SK-MM-2	MM	sub	NCOR2	c.2128G>A	p.V710M	missense	97	0.51
12	132512718	C	T	SK-MM-2	MM	sub	EP400	c.5263C>T	p.R1755C	missense	101	0.53
13	92408666	A	G	SK-MM-2	MM	sub	GPC5	c.1272A>G	p.I424M	missense	76	0.052631579
17	15968292	G	T	SK-MM-2	MM	sub	NCOR1	c.4993C>A	p.P1665T	missense	75	0.55
2	152506848	T	A	SK-MM-2	MM	sub	NEB	c.7273A>T	p.S2425C	missense	102	0.4
2	152525618	G	T	SK-MM-2	MM	sub	NEB	c.4534C>A	p.L1512M	missense	103	0.019417476
3	141248570	G	C	SK-MM-2	MM	sub	RASA2	c.376G>C	p.E126Q	missense	85	0.47
6	32190369	G	A	SK-MM-2	MM	sub	NOTCH4	c.370C>T	p.P124S	missense	65	0.45
X	15840962	C	A	SK-MM-2	MM	sub	ZRSR2	c.1046C>A	p.S349Y	missense	53	0.11
13	48934228	A	G	U-266	MM	sub	RB1	c.683A>G	p.K228R	missense	90	1
13	48951156	G	T	U-266	MM	sub	RB1	c.1318G>T	p.E440*	nonsense	51	0.98
15	91328204	G	A	U-266	MM	sub	BLM	c.2716G>A	p.D906N	missense	174	0.49
17	7578449	C	T	U-266	MM	sub	TP53	c.481G>A	p.A161T	missense	47	1
4	126370177	T	C	U-266	MM	sub	FAT4	c.8006T>C	p.F2669S	missense	215	0.49
6	26056416	T	A	U-266	MM	sub	HIST1H1C	c.241A>T	p.K81*	nonsense	156	0.52
7	140453132	T	A	U-266	MM	sub	BRAF	c.1803A>T	p.K601N	missense	162	0.58
15	91292768	TA	T	CTV-1	MM	ind	BLM	NA	p.N92fs*37	frameshift_d	218	0.279816514
13	32910800	AT	A	CTV-1	MM	ind	BRCA2	NA	p.L771fs*1	frameshift_d	170	0.370588235
6	160485487	CG	C	CTV-1	MM	ind	IGF2R	NA	p.D1317fs*2	frameshift_d	176	NO
14	95596485	AT	A	CTV-1	MM	ind	DICER1	NA	p.N161fs*22	frameshift_d	120	0.391666667
12	56492627	AG	A	CTV-1	MM	ind	ERBB3	NA	p.E928fs*16	frameshift_d	136	0.397058824
12	6499901	TG	T	CTV-1	MM	ind	LTBR	NA	p.G371fs*42	frameshift_d	109	0.403669725
5	66460200	GC	G	CTV-1	MM	ind	MAST4	NA	p.P1544fs*1	frameshift_d	142	0.443661972
15	42032391	A	AA	CTV-1	MM	ind	MGA	NA	p.R1575fs*2	frameshift_ir	142	0.387323944
11	58979362	AG	A	CTV-1	MM	ind	MPEG1	NA	p.L326fs*2	frameshift_d	111	0.423423423
3	170781744	CA	C	CTV-1	MM	ind	TNIK	NA	p.F1336fs*1	frameshift_d	100	0.51
16	72984639	TC	T	CTV-1	MM	ind	ZFHX3	NA	p.D982fs*87	frameshift_d	119	0.428571429
16	72991665	AG	A	CTV-1	MM	ind	ZFHX3	NA	p.S794fs*30	frameshift_d	101	0.425742574
2	9484896	TA	T	KARPAS-45	non_MM	ind	ASAP2	NA	p.N332fs*4	frameshift_d	209	0.234449761
13	32913836	CA	C	KARPAS-45	non_MM	ind	BRCA2	NA	p.N1784fs*7	frameshift_d	469	0.198294243
6	15496721	TG	T	KARPAS-45	non_MM	ind	JARID2	NA	p.R425fs*36	frameshift_d	380	0.286842105
6	15496720	GT	G	KARPAS-45	non_MM	ind	JARID2	NA	p.V422fs*39	frameshift_d	385	0.301298701
6	51618047	TC	T	KARPAS-45	non_MM	ind	PKHD1	NA	p.R2968fs*7	frameshift_d	457	0.043763676
9	139793211	AC	A	KARPAS-45	non_MM	ind	TRAF2	NA	p.P9fs*77	frameshift_d	129	0.07751938
10	63759905	GTACCGCTC	G	LP-1	MM	ind	ARID5B	NA	p.Y187_D19	inframe_del	109	0.211009174
14	103363634	AAGAGCATA	A	LP-1	MM	ind	TRAF3	NA	p.K286fs*7	frameshift_d	60	0.766666667
14	103372020	GTCTTTGTG	G	MM1S	MM	ind	TRAF3	NA	p.V536_N54	complex sub	45	0.8
1	27099013	GC	G	MN-60	non_MM	ind	ARID1A	NA	p.Q1145fs*1	frameshift_d	200	0.37
14	58814450	G	GA	MN-60	non_MM	ind	ARID4A	NA	p.D423fs*24	frameshift_ir	218	0.403669725
6	41903745	C	CG	MN-60	non_MM	ind	CCND3	NA	p.R271fs*53	frameshift_ir	211	0.379146919
5	150885188	TG	T	MN-60	non_MM	ind	FAT2	NA	p.N4330fs*6	frameshift_d	246	0.345528455
4	153303351	CA	C	MN-60	non_MM	ind	FBXW7	NA	p.C46fs*5	frameshift_d	223	0.255605381
6	15496728	G	GG	MN-60	non_MM	ind	JARID2	NA	p.R425fs*99	frameshift_ir	233	0.248927039
15	42042482	GA	G	MN-60	non_MM	ind	MGA	NA	p.S2277fs*1	frameshift_d	300	0.363333333
17	58740623	CA	C	MN-60	non_MM	ind	PPM1D	NA	p.N512fs*2	frameshift_d	300	0.436666667
1	118165768	T	TC	NCI-H929	MM	ind	FAM46C	NA	p.I94fs*16	frameshift_ir	71	0.85915493
X	39932207	C	CA	RPMI-8866	non_MM	ind	BCOR	NA	p.V798fs*19	frameshift_ir	253	0.438735178
14	103372062	A	AT	U-266	MM	ind	TRAF3	NA	p.K550fs*3	frameshift_ir	110	0.854545455
