# Supplementary material for: A DNA target-enrichment approach to detect mutations, copy number changes and immunoglobulin translocations in multiple myeloma
Source: Blood Cancer J. 2016 Sep 2;6(9):e467–. doi: 10.1038/bcj.2016.72 (PMC5056967; doi:10.1038/bcj.2016.72)
Supplement: Supplementary Table 5 [file bcj201672x8.pdf]

cell lines	chr_h	h5	h3	chr_l	l5	l3	ID	reads_h	dir_h	dir_l	reads_l	qual_h	qual_l	high_confidence
ARH-77	11	65433560	65433635	14	106828541	106828616	11+_14+	2	+	+	2	60	60	
ARH-77	16	78131369	78131396	14	107174448	107174616	16+_14+	2	+	+	2	4	60	
ARH-77	8	125184242	125184279	14	106052731	106919195	8+_14+	2	+	+	2	0	30	
ARH-77	11	64929102	64929177	14	106901486	106901561	11+_14-	2	+	-	2	51	60	
ARH-77	11	68137820	68137840	14	106088599	106131326	11-_14+	2	-	+	2	3	51	
ARH-77	11	68291169	68291196	14	106198255	106623102	11-_14+	2	-	+	2	3.5	60	
ARH-77	6	41757783	41757806	14	107229651	107229726	6-_14+	2	-	+	2	17	60	
ARH-77	8	125184242	125184279	14	106211284	107270964	8-_14-	2	-	-	2	0	0.33333333	
KMS-12-BM	8	125184244	125184278	14	106014274	106172979	8+_14+	3	+	+	3	1.33333333	28.2	
KMS-12-BM	11	69139800	69139884	14	106112683	106211011	11-_14+	2	-	+	2	36	22	
KMS-12-BM	11	69159770	69159854	14	106060283	106181176	11-_14+	5	-	+	5	60	1	TRUE
KMS-12-BM	11	69002107	69002146	14	106016528	106773049	11-_14-	2	-	-	2	1.5	60	
L-363	11	64748631	64748852	14	106091338	106091551	11-_14+	21	-	+	21	49.0952381	31.3428571	TRUE
LP-1	11	67056821	67057103	14	106127994	107113173	11+_14+	2	+	+	2	0	30	
LP-1	11	67978737	67978757	14	106988052	106988072	11+_14+	2	+	+	2	3	60	
LP-1	8	125184242	125184275	14	106052728	107055364	8+_14+	3	+	+	3	2	23	
LP-1	8	125570135	125570157	14	106019031	107064917	8+_14+	2	+	+	2	0	60	
LP-1	8	125969371	125969446	14	106987424	106987499	8+_14+	2	+	+	2	39	50.5	
LP-1	8	129023667	129023985	14	106048402	106167495	8+_14+	27	+	+	27	54.8888889	25.6571429	TRUE
LP-1	11	67204669	67204689	14	106150602	106150645	11+_14-	2	+	-	2	3.5	12	
LP-1	11	67668494	67668514	14	107154856	107183508	11+_14-	2	+	-	2	0	35.5	
LP-1	4	1905624	1905902	14	106212840	106213119	4+_14-	44	+	-	44	55.2954546	44.9090909	TRUE
LP-1	8	125184247	125184281	14	107273956	107279160	8+_14-	2	+	-	2	0	60	
LP-1	16	78211919	78211994	14	106607734	106607809	16-_14+	2	-	+	2	60	9	
LP-1	16	78349451	78349570	14	106211553	106598380	16-_14+	4	-	+	4	34.25	24.8	
LP-1	4	1857160	1857268	14	106324063	106324213	4-_14+	2	-	+	2	60	60	
LP-1	8	127426547	127426580	14	107190664	107190911	8-_14+	4	-	+	4	0	60	
LP-1	4	1857460	1857546	14	106324912	106325062	4-_14-	2	-	-	2	60	60	
LP-1	4	1905892	1906146	14	106211454	106212712	4-_14-	52	-	-	52	55.3846154	17.3529412	TRUE
LP-1	8	125903029	125903050	14	107045405	107045504	8-_14-	2	-	-	2	0	36.5	
LP-1	8	129008973	129009078	14	106023113	106143769	8-_14-	33	-	-	33	53.1212121	10.4901961	TRUE
OPM-2	11	65225709	65225736	14	107034309	107073292	11+_14+	2	+	+	2	0	60	
OPM-2	4	1914633	1914819	14	106112630	106176478	4+_14+	31	+	+	31	38.0645161	37.8085106	TRUE
OPM-2	8	125184242	125184269	14	106052684	106172976	8+_14+	2	+	+	2	1.5	0	
OPM-2	8	129799390	129799598	14	106232473	106232847	8+_14+	76	+	+	76	59.2894737	52.1584158	TRUE
OPM-2	8	127606999	127607018	14	106059977	106181930	8+_14-	2	+	-	2	0	0	
OPM-2	11	68291165	68291200	14	106734169	107200085	11-_14+	2	-	+	2	7.5	60	
OPM-2	11	68667309	68667384	14	106201607	106229839	11-_14-	2	-	-	2	60	0	
OPM-2	4	1914907	1915167	14	106176444	106327196	4-_14-	25	-	-	25	36.76	48.0882353	TRUE
SK-MM-2	11	67154878	67154897	14	106112457	106112476	11+_14+	2	+	+	2	0	11	
SK-MM-2	16	79135951	79136001	14	106056667	106172984	16+_14+	2	+	+	2	0	0	
SK-MM-2	8	125184242	125184277	14	106052719	106172886	8+_14+	3	+	+	3	1	21	
SK-MM-2	8	127606997	127607018	14	106059948	106181375	8+_14-	2	+	-	2	0	0	
SK-MM-2	11	69265829	69266031	14	106211080	106239618	11-_14+	30	-	+	30	50.8	6.175	TRUE
SK-MM-2	8	127878728	127878750	14	106155367	106155389	8-_14-	2	-	-	2	0	60	
U-266	11	69443711	69443838	14	106068843	106069009	11+_14-	11	+	-	11	59.3636364	51.4285714	TRUE
U-266	11	69443862	69444017	14	106176784	106176971	11-_14+	16	-	+	16	53.8125	60	TRUE
U-266	20	38497661	38497685	14	106719837	106719942	20-_14-	2	-	-	2	0	60	
CTV-1	11	65389047	65389376	14	106481569	106793432	11+_14+	2	+	+	2	30	30	
CTV-1	11	66614890	66614923	14	106172945	106734227	11+_14+	2	+	+	2	6.5	14.6666667	
CTV-1	16	78739944	78739975	14	106137492	106137588	16+_14-	2	+	-	2	0	0	
IM-9	8	125184249	125184279	14	106547404	107282897	8+_14+	2	+	+	2	0	60	
IM-9	11	67204669	67204689	14	106031038	106150601	11+_14-	2	+	-	2	2	13	
IM-9	11	64647646	64647684	14	107020026	107020236	11-_14-	4	-	-	4	0	54.25	
IM-9	11	65243025	65243052	14	106011838	106011929	11-_14-	2	-	-	2	4.5	12	
KMS-11	8	125184242	125184278	14	106014358	106919196	8+_14+	4	+	+	4	0.25	42	
KMS-11	16	78349451	78349698	14	106211275	106598396	16-_14+	19	-	+	19	41.1578947	18.6521739	TRUE
KMS-11	4	1857159	1857347	14	106323733	106324247	4-_14+	60	-	+	60	57.95	55.5340909	TRUE
KMS-11	11	64856007	64856029	14	106097935	106117903	11-_14-	2	-	-	2	0.5	11	
KMS-11	4	1857431	1857611	14	106324881	106325049	4-_14-	17	-	-	17	53.9411765	56.16	TRUE
MC-CAR	11	67204667	67204691	14	106030988	106150586	11+_14-	5	+	-	5	2.6	8.5	
MC-CAR	16	78777326	78777345	14	106363330	106363349	16+_14-	2	+	-	2	0	60	
MC-CAR	8	125184243	125184277	14	106089573	106158865	8+_14-	3	+	-	3	0.33333333	9.25	
MC-CAR	8	125184242	125184280	14	105994568	106358672	8-_14+	3	-	+	3	0	24	
MC-CAR	8	129414257	129414277	14	106164535	106164629	8-_14+	3	-	+	3	0	21.3333333	
MC-CAR	8	125184241	125184277	14	106041840	106094637	8-_14-	3	-	-	3	5	17.6	
MM1S	8	125184242	125184276	14	106014361	106173009	8+_14+	2	+	+	2	0	40	
MM1S	8	129689173	129689338	14	106063756	106185713	8+_14+	41	+	+	41	32.195122	3.625	TRUE
MM1S	16	78802660	78802771	14	106324819	106324986	16+_14-	13	+	-	13	55.3846154	60	TRUE
MM1S	16	78802777	78802927	14	106324559	106324793	16-_14+	26	-	+	26	57.4615385	46.7647059	TRUE
MM1S	8	127426547	127426581	14	107190840	107190952	8-_14+	3	-	+	3	0	60	
NCI-H929	11	65225705	65225736	14	106035582	107119344	11+_14+	2	+	+	2	0	60	
NCI-H929	4	1904551	1904702	14	106176040	106176253	4+_14+	21	+	+	21	49.9047619	59.7142857	TRUE
NCI-H929	4	1904708	1904921	14	106325082	106325696	4-_14-	34	-	-	34	51.9117647	23.4081633	TRUE
NCI-H929	4	1949430	1949505	14	106399493	106399568	4-_14-	2	-	-	2	60	60	
EHEB	8	125184242	125184278	14	106052692	106815537	8+_14+	6	+	+	6	0.66666667	3.63636364	
EHEB	8	127426547	127426580	14	107190671	107190907	8-_14+	2	-	+	2	0	60	
EHEB	11	65628949	65629024	14	106696111	106696186	11-_14-	2	-	-	2	60	60	
EHEB	11	65851249	65851268	14	106838237	106838266	11-_14-	2	-	-	2	0	60	
EHEB	8	125184242	125184278	14	106094650	107155300	8-_14-	3	-	-	3	0	31.75	
JVM-3	11	67203007	67203058	14	106093300	106093375	11+_14+	2	+	+	2	60	0	
JVM-3	11	67204669	67204689	14	106031022	106150597	11+_14-	2	+	-	2	0.5	8.33333333	
JVM-3	11	67346374	67346453	14	107100649	107100745	11-_14+	2	-	+	2	0	56	
HL-60	11	66537207	66537282	14	107189508	107189583	11+_14+	2	+	+	2	20	60	
HL-60	8	125184245	125184277	14	106014359	106714466	8+_14+	4	+	+	4	0	22.6666667	
HL-60	8	126895552	126895577	14	106640951	106640976	8+_14+	2	+	+	2	13	29	
HL-60	8	128188035	128188110	14	106221621	106221696	8+_14+	2	+	+	2	60	12	
HL-60	8	129082879	129082899	14	106265735	106434294	8+_14+	2	+	+	2	3	44.5	
HL-60	8	129465161	129465230	14	106929955	106934125	8+_14+	2	+	+	2	1	44	
HL-60	11	67204669	67204690	14	106031086	106150576	11+_14-	2	+	-	2	4.5	7.75	
HL-60	11	67388713	67388754	14	106223050	106223091	11+_14-	3	+	-	3	0	0	
HL-60	8	126391240	126391299	14	106649564	106649639	8+_14-	2	+	-	2	60	41	
HL-60	8	126479333	126479408	14	106937027	106937102	8+_14-	2	+	-	2	60	12	
HL-60	8	126794679	126795049	14	106314348	107028335	8+_14-	3	+	-	3	60	47.6666667	
HL-60	8	126860014	126860033	14	106442559	106442578	8+_14-	2	+	-	2	0	0	
HL-60	8	128212187	128212231	14	107238283	107238358	8+_14-	2	+	-	2	60	60	

HL-60	8	128753228	128753311	14	107103400	107199267	8+_14-	2	+	-	2	60	30	
HL-60	11	65074123	65074173	14	107211453	107211528	11-_14+	2	-	+	2	60	60	
HL-60	11	65225707	65225733	14	106133216	106418089	11-_14+	3	-	+	3	0	15.2	
HL-60	11	67985250	67985290	14	107072463	107072581	11-_14+	2	-	+	2	0	37	
HL-60	11	68137816	68137840	14	106088520	106088677	11-_14+	2	-	+	2	6	49.5	
HL-60	11	68291173	68291200	14	106120241	106252041	11-_14+	2	-	+	2	0	20	
HL-60	8	126651324	126651399	14	106187990	106188065	8-_14+	2	-	+	2	60	47.5	
HL-60	8	127179949	127179974	14	106521236	106521261	8-_14+	2	-	+	2	36	60	
HL-60	8	128703386	128703461	14	106411601	106411676	8-_14+	2	-	+	2	60	60	
HL-60	8	128751592	128751844	14	106781813	106971219	8-_14+	2	-	+	2	30	30.5	
HL-60	8	129801810	129801864	14	107212014	107212089	8-_14+	2	-	+	2	60	37.5	
HL-60	11	64647649	64647685	14	107020110	107020222	11-_14-	2	-	-	2	0	60	
HL-60	11	67015283	67015336	14	106875494	106875569	11-_14-	2	-	-	2	60	60	
HL-60	11	69002107	69002226	14	106054431	107176871	11-_14-	2	-	-	2	0	21.5	
HL-60	20	38673106	38673126	14	106719830	106719894	20-_14-	2	-	-	2	0	60	
HL-60	8	125184242	125184274	14	106094660	106418601	8-_14-	2	-	-	2	1	14.5	
HL-60	8	126744556	126744631	14	106932134	106932209	8-_14-	2	-	-	2	60	0	
HL-60	8	126923562	126923637	14	106828057	106828132	8-_14-	2	-	-	2	60	60	
HL-60	8	127101659	127101734	14	106538747	106538822	8-_14-	2	-	-	2	60	60	
HL-60	8	128122886	128122961	14	106910968	106911043	8-_14-	2	-	-	2	60	60	
HL-60	8	128394506	128394563	14	106109136	106235245	8-_14-	2	-	-	2	60	0	
HL-60	8	128765289	128765314	14	106832256	106832281	8-_14-	2	-	-	2	30	45	
HL-60	8	129047842	129047902	14	106652904	106652979	8-_14-	2	-	-	2	60	29	
HL-60	8	129186158	129186233	14	106693645	106693720	8-_14-	2	-	-	2	60	60	
KASUMI-1	11	65225705	65225734	14	106405131	106876398	11+_14+	2	+	+	2	0	41.5	
KASUMI-1	11	65780135	65780170	14	106088756	106371277	11+_14+	2	+	+	2	0	47.5	
KASUMI-1	8	125184242	125184274	14	106014355	106800726	8+_14+	3	+	+	3	1	30	
KASUMI-1	8	126058435	126058510	14	106520122	106520197	8+_14+	2	+	+	2	60	32	
KASUMI-1	8	128947625	128947681	14	106878325	106878400	8+_14+	2	+	+	2	60	47	
KASUMI-1	11	64604172	64604200	14	106102671	106299147	11+_14-	3	+	-	3	0.66666667	23	
KASUMI-1	11	65686674	65686697	14	106108339	106317267	11+_14-	2	+	-	2	7.5	36	
KASUMI-1	8	125184245	125184279	14	106032764	107176717	8+_14-	3	+	-	3	0	16.6666667	
KASUMI-1	8	128951237	128951312	14	107000791	107000866	8+_14-	2	+	-	2	60	60	
KASUMI-1	11	65225705	65225724	14	106089970	106317155	11-_14+	2	-	+	2	0	0	
KASUMI-1	11	68439837	68439912	14	107194290	107194365	11-_14+	2	-	+	2	60	28	
KASUMI-1	8	125852721	125852766	14	106405520	106405595	8-_14+	2	-	+	2	60	60	
KASUMI-1	8	127426547	127426580	14	107190765	107190938	8-_14+	4	-	+	4	0	60	
KASUMI-1	11	64647646	64647681	14	107020055	107020222	11-_14-	4	-	-	4	0	60	
KASUMI-1	11	65225706	65225736	14	106492275	106597930	11-_14-	2	-	-	2	10.5	60	
KASUMI-1	11	68375954	68376029	14	106087188	106129975	11-_14-	2	-	-	2	60	0	
KASUMI-1	16	79092697	79092717	14	107196375	107196395	16-_14-	2	-	-	2	6	60	
KASUMI-1	8	126621022	126621062	14	106352418	106352493	8-_14-	2	-	-	2	60	41	
KASUMI-1	8	126741486	126741561	14	106720689	106720764	8-_14-	2	-	-	2	60	11	
KASUMI-1	8	129465139	129465202	14	106831478	107165256	8-_14-	2	-	-	2	0	30	
CESS	8	125184242	125184272	14	106014370	106173017	8+_14+	4	+	+	4	0.75	24	
CESS	8	128204452	128204472	14	106783277	106783337	8+_14+	2	+	+	2	0	14.5	
CESS	8	127426547	127426580	14	107190759	107190953	8-_14+	5	-	+	5	0	58.6666667	
CESS	8	129414258	129414277	14	106164529	106164591	8-_14+	2	-	+	2	0	22.5	
CESS	11	68291167	68291202	14	106034183	106141595	11-_14-	3	-	-	3	0	25.5	
CESS	11	69002207	69002227	14	106094467	106239587	11-_14-	2	-	-	2	3	32.5	
MN-60	8	125184242	125184279	14	106172892	106657348	8+_14+	4	+	+	4	0	31.2	
MN-60	11	67204669	67204689	14	106031060	106150646	11+_14-	2	+	-	2	0.5	12.6666667	
MN-60	8	127606998	127607018	14	106060391	106180770	8+_14-	2	+	-	2	0	0	
MN-60	8	128748776	128749043	14	106326987	106909413	8+_14-	72	+	-	72	57.5	56.967033	TRUE
MN-60	11	67057085	67057108	14	106052712	106172965	11-_14+	2	-	+	2	2.5	0	
MN-60	16	78996199	78996239	14	106121225	106593381	16-_14+	2	-	+	2	0	9.5	
MN-60	8	125184241	125184278	14	106088704	106207472	8-_14+	3	-	+	3	0	33.6	
MN-60	8	125241819	125241873	14	106756777	107025253	8-_14+	3	-	+	3	0	35.3333333	
MN-60	8	127426547	127426580	14	107190845	107190966	8-_14+	2	-	+	2	0	60	
MN-60	8	128749046	128749271	14	106326794	106327016	8-_14+	79	-	+	79	57.3291139	57.9207921	TRUE
MN-60	8	129414258	129414277	14	106164401	106164604	8-_14+	3	-	+	3	0	43	
MN-60	11	67415171	67415190	14	106173504	106173559	11-_14-	2	-	-	2	0	18	
MN-60	8	125184242	125184279	14	106041824	106804371	8-_14-	6	-	-	6	1.16666667	21.75	
MN-60	8	128267386	128267411	14	106719817	106719884	8-_14-	2	-	-	2	1.5	60	
BALL-1	11	64929043	64929097	14	107280273	107280470	11+_14+	2	+	+	2	0.5	60	
BALL-1	8	125184242	125184277	14	106014334	106173029	8+_14+	6	+	+	6	1.66666667	20.5714286	
BALL-1	8	128204452	128204472	14	106783222	106783357	8+_14+	2	+	+	2	0	44.5	
BALL-1	11	67204667	67204691	14	106150625	106232384	11+_14-	2	+	-	2	3	34	
BALL-1	11	69438790	69439060	14	106346891	106347354	11+_14-	86	+	-	86	57.6162791	57.2857143	TRUE
BALL-1	16	78739945	78739974	14	106137362	106137627	16+_14-	3	+	-	3	0	4	
BALL-1	16	78996201	78996239	14	106221704	106288366	16+_14-	2	+	-	2	0	25	
BALL-1	4	1913310	1913351	14	106719801	106719948	4+_14-	2	+	-	2	0	60	
BALL-1	8	125184245	125184274	14	106165742	106228079	8+_14-	2	+	-	2	0	30	
BALL-1	8	129013893	129014155	14	106027069	106147680	8+_14-	88	+	-	88	55.4659091	11.0857143	TRUE
BALL-1	8	129465161	129465221	14	106283294	106887763	8+_14-	2	+	-	2	0	60	
BALL-1	11	65936240	65936315	14	106150301	106150376	11-_14+	2	-	+	2	60	0	
BALL-1	11	67057085	67057114	14	106172940	106825104	11-_14+	3	-	+	3	1.33333333	11	
BALL-1	11	68137818	68137839	14	106088629	106131995	11-_14+	2	-	+	2	0	51	
BALL-1	11	69439057	69439224	14	106330268	106330455	11-_14+	13	-	+	13	55.8461539	60	TRUE
BALL-1	8	125184244	125184281	14	106130631	106305494	8-_14+	2	-	+	2	3.5	39.3333333	
BALL-1	8	126208433	126208489	14	106855745	106855829	8-_14+	2	-	+	2	1	12	
BALL-1	11	64647649	64647681	14	107020085	107020236	11-_14-	5	-	-	5	0	60	
BALL-1	11	65225706	65225731	14	106691869	107034232	11-_14-	2	-	-	2	0	52	
BALL-1	8	125517092	125517151	14	107279816	107279891	8-_14-	2	-	-	2	0	60	
BALL-1	8	126222947	126222970	14	106569911	106569993	8-_14-	2	-	-	2	0	29	
BALL-1	8	128188765	128188796	14	107069810	107069841	8-_14-	2	-	-	2	15	60	
KARPAS-45	11	64496786	64496812	14	106069256	106743767	11+_14+	3	+	+	3	0	24	
KARPAS-45	11	64929044	64929097	14	107280274	107280431	11+_14+	4	+	+	4	1	60	
KARPAS-45	11	67204667	67204689	14	106056820	107048901	11+_14+	3	+	+	3	1	2	
KARPAS-45	8	125184245	125184278	14	106014327	106938761	8+_14+	4	+	+	4	0	52	
KARPAS-45	8	129414258	129414277	14	106039447	106160114	8+_14+	4	+	+	4	0	0	
KARPAS-45	11	67149204	67149279	14	106168993	106169068	11+_14-	2	+	-	2	60	60	
KARPAS-45	8	125184242	125184279	14	106234787	106667681	8+_14-	2	+	-	2	0.5	30	
KARPAS-45	8	129414258	129414277	14	106044940	106045062	8+_14-	2	+	-	2	0	0	
KARPAS-45	11	68137816	68137840	14	106088634	106233358	11-_14+	2	-	+	2	7.5	29	
KARPAS-45	16	78804548	78804571	14	106052652	106172960	16-_14+	3	-	+	3	0	29.6666667	
KARPAS-45	16	79287919	79287944	14	106600892	106677181	16-_14+	2	-	+	2	0	28.5	

KARPAS-45	8	125184244	125184279	14	106032718	106307018	8-_14+	2	-	+	2	0	30
KARPAS-45	8	129414258	129414277	14	106164486	106164557	8-_14+	4	-	+	4	0	16.5
KARPAS-45	11	65555114	65555254	14	106217536	107150979	11-_14-	2	-	-	2	0	59.3333333
KARPAS-45	11	67529282	67529357	14	107090627	107090702	11-_14-	2	-	-	2	34	60
KARPAS-45	11	68362658	68362696	14	106475773	107230031	11-_14-	2	-	-	2	0	60
KARPAS-45	8	125184242	125184277	14	106094679	106938501	8-_14-	2	-	-	2	0	10.5
K-562	16	78131369	78131394	14	107174421	107174535	16+_14+	3	+	+	3	0	60
K-562	8	125819524	125819546	14	106405297	106405384	8-_14+	2	-	+	2	0	60
RPMI-8866	11	64929042	64929097	14	107280275	107280480	11+_14+	2	+	+	2	0	60
RPMI-8866	8	125184242	125184273	14	106172984	106225713	8+_14+	2	+	+	2	0	1
RPMI-8866	16	79223819	79223841	14	106214795	107105825	16+_14-	2	+	-	2	0	30
RPMI-8866	8	127426547	127426580	14	107190968	107191057	8+_14-	2	+	-	2	0	42.25
RPMI-8866	8	129414258	129414277	14	106164547	106164566	8-_14+	2	-	+	2	0	11.5
RPMI-8866	8	125184244	125184276	14	106025319	106211272	8-_14-	2	-	-	2	0	9.66666667
