# Supplementary material for: A DNA target-enrichment approach to detect mutations, copy number changes and immunoglobulin translocations in multiple myeloma
Source: Blood Cancer J. 2016 Sep 2;6(9):e467–. doi: 10.1038/bcj.2016.72 (PMC5056967; doi:10.1038/bcj.2016.72)
Supplement: Supplementary Table 6 [file bcj201672x9.pdf]

cell_lines	chr_h	h5	h3	chr_l	l5	l3	sample	normal	qual_h	qual_l	size	quality	depth	coord	n_of_duplicates	high_confidence
ARH-77	14	106072000	106076000	14	106076000	106080000	204	29	24.2401961	24.7647059	4000	FALSE	FALSE	106072000_106076000_106076000_106080000	17	
ARH-77	14	106082500	106086500	14	106103500	106107500	22	0	11.1818182	3.68181818	21000	FALSE	TRUE	106082500_106086500_106103500_106107500	6	
ARH-77	14	106098500	106102500	14	106192000	106196000	1	0	60	0	93500	FALSE	FALSE	106098500_106102500_106192000_106196000	2	
ARH-77	14	106173000	106177000	14	106324500	106328500	1	0	0	35	151500	FALSE	FALSE	106173000_106177000_106324500_106328500	1	
ARH-77	14	106208000	106212000	14	106324500	106328500	49	0	48.244898	60	116500	TRUE	TRUE	106208000_106212000_106324500_106328500	2	
ARH-77	14	106328500	106332500	14	106827500	106831500	11	0	55.5454545	9	499000	TRUE	TRUE	106328500_106332500_106827500_106831500	1	TRUE
ARH-77	14	106072000	106076000	14	106118927	106119413	8	0	13.625	2.375	45170	FALSE	FALSE	106072000_106076000_106118927_106119413	1	
ARH-77	14	106072000	106076000	14	106120824	106121164	2	1	22	23.5	46994	FALSE	FALSE	106072000_106076000_106120824_106121164	1	
ARH-77	14	106072000	106076000	14	106217823	106218246	5	0	8.2	3.6	144034.5	FALSE	FALSE	106072000_106076000_106217823_106218246	1	
ARH-77	14	106082500	106086500	14	106102226	106102293	8	1	44.625	1	17759.5	TRUE	FALSE	106082500_106086500_106102226_106102293	1	
ARH-77	14	106082500	106086500	14	106102498	106102570	3	0	14.3333333	7.33333333	18034	FALSE	FALSE	106082500_106086500_106102498_106102570	1	
ARH-77	14	106082500	106086500	14	106128151	106128496	3	1	0	15.3333333	43823.5	FALSE	FALSE	106082500_106086500_106128151_106128496	1	
ARH-77	14	106082500	106086500	14	106129171	106129433	2	0	0	0	44802	FALSE	FALSE	106082500_106086500_106129171_106129433	1	
ARH-77	14	106082500	106086500	14	106198232	106198347	2	0	3.5	5	113789.5	FALSE	FALSE	106082500_106086500_106198232_106198347	1	
ARH-77	14	106082500	106086500	14	106200472	106200820	13	0	11	0	116146	FALSE	TRUE	106082500_106086500_106200472_106200820	1	
ARH-77	14	106082500	106086500	14	106201082	106201493	5	0	0	10.4	116787.5	FALSE	FALSE	106082500_106086500_106201082_106201493	1	
ARH-77	14	106082500	106086500	14	106201967	106202350	3	0	31	0	117658.5	FALSE	FALSE	106082500_106086500_106201967_106202350	1	
ARH-77	14	106082500	106086500	14	106227198	106227616	35	6	44.8285714	53.0285714	142907	TRUE	FALSE	106082500_106086500_106227198_106227616	1	
ARH-77	14	106082500	106086500	14	106230100	106230425	3	0	3	0	145762.5	FALSE	FALSE	106082500_106086500_106230100_106230425	1	
ARH-77	14	106094675	106094749	14	106095000	106099000	2	0	55.5	52.75	2288	TRUE	FALSE	106094675_106094749_106095000_106099000	1	
ARH-77	14	106098500	106102500	14	106197182	106197327	4	0	1.5	18.75	96754.5	FALSE	FALSE	106098500_106102500_106197182_106197327	1	
ARH-77	14	106098500	106102500	14	106225521	106225595	2	0	10	14	125058	FALSE	FALSE	106098500_106102500_106225521_106225595	1	
ARH-77	14	106098500	106102500	14	106226346	106226919	5	1	3.4	12.4	126132.5	FALSE	FALSE	106098500_106102500_106226346_106226919	1	
ARH-77	14	106088147	106088361	14	106103500	106107500	3	0	9	0	17246	FALSE	FALSE	106088147_106088361_106103500_106107500	1	
ARH-77	14	106143000	106147000	14	106147205	106147311	13	0	14.0769231	14.0769231	2258	FALSE	TRUE	106143000_106147000_106147205_106147311	1	
ARH-77	14	106045694	106045800	14	106163500	106167500	3	1	0	0	119753	FALSE	FALSE	106045694_106045800_106163500_106167500	1	
ARH-77	14	106046126	106046522	14	106163500	106167500	10	0	4	6.1	119176	FALSE	TRUE	106046126_106046522_106163500_106167500	1	
ARH-77	14	106163158	106163299	14	106163500	106167500	5	0	16.0714286	16.0714286	2271.5	FALSE	FALSE	106163158_106163299_106163500_106167500	1	
ARH-77	14	106173000	106177000	14	106177211	106177379	8	0	59	60	2295	TRUE	FALSE	106173000_106177000_106177211_106177379	1	
ARH-77	14	106123981	106124118	14	106192000	106196000	2	0	0	0	69950.5	FALSE	FALSE	106123981_106124118_106192000_106196000	1	
ARH-77	14	106191673	106191795	14	106192000	106196000	5	1	25.9230769	22.9230769	2266	FALSE	FALSE	106191673_106191795_106192000_106196000	1	
ARH-77	14	106196000	106200000	14	106200214	106200376	6	1	60	60	2295	TRUE	FALSE	106196000_106200000_106200214_106200376	1	
ARH-77	14	106196000	106200000	14	106227200	106227350	2	0	11	26	29275	FALSE	FALSE	106196000_106200000_106227200_106227350	1	
ARH-77	14	106196000	106200000	14	106227598	106227698	3	0	19.6666667	1	29648	FALSE	FALSE	106196000_106200000_106227598_106227698	1	
ARH-77	14	106208000	106212000	14	106238230	106238481	7	0	35.2857143	45.8571429	28355.5	TRUE	FALSE	106208000_106212000_106238230_106238481	1	
ARH-77	14	106208000	106212000	14	106239103	106239203	6	0	19.5	8	29153	FALSE	FALSE	106208000_106212000_106239103_106239203	1	
ARH-77	14	106796134	106796285	14	106796500	106800500	4	7	32.6	31.6	2290.5	TRUE	FALSE	106796134_106796285_106796500_106800500	1	
ARH-77	14	106941000	106945000	14	106945210	106945413	12	6	60	60	2311.5	TRUE	FALSE	106941000_106945000_106945210_106945413	1	
ARH-77	14	107076500	107080500	14	107083254	107083462	25	0	37.68	0	4858	FALSE	TRUE	107076500_107080500_107083254_107083462	1	
ARH-77	14	107076500	107080500	14	107095124	107095282	30	0	37.6333333	0	16703	FALSE	TRUE	107076500_107080500_107095124_107095282	1	
ARH-77	14	107076500	107080500	14	107153649	107153766	2	0	0	60	75207.5	FALSE	FALSE	107076500_107080500_107153649_107153766	1	
ARH-77	14	107086685	107086976	14	107097000	107101000	14	12	0	6.28571429	12169.5	FALSE	FALSE	107086685_107086976_107097000_107101000	1	
ARH-77	14	107087195	107087296	14	107097000	107101000	4	3	56.6666667	4	11754.5	TRUE	FALSE	107087195_107087296_107097000_107101000	1	
ARH-77	14	107096689	107096763	14	107097000	107101000	3	5	38.1666667	36.8333333	2274	TRUE	FALSE	107096689_107096763_107097000_107101000	1	
ARH-77	14	107093118	107093393	14	107271500	107275500	2	0	48	25	180244.5	TRUE	FALSE	107093118_107093393_107271500_107275500	1	
ARH-77	14	107271107	107271285	14	107271500	107275500	4	14	51.6875	53.625	2304	TRUE	FALSE	107271107_107271285_107271500_107275500	1	
KMS-12-BM	14	106022500	106026500	14	106143500	106147500	22	0	5.31818182	5.45454545	121000	FALSE	TRUE	106022500_106026500_106143500_106147500	1	
KMS-12-BM	14	106047500	106051500	14	106164000	106168000	1	0	50	0	116500	FALSE	FALSE	106047500_106051500_106164000_106168000	3	
KMS-12-BM	14	106078500	106082500	14	106223500	106227500	23	3	26.6956522	44.0869565	145000	TRUE	FALSE	106078500_106082500_106223500_106227500	1	
KMS-12-BM	14	106090500	106094500	14	106092500	106096500	3100	29	28.5383871	30.2536302	2000	TRUE	FALSE	106090500_106094500_106092500_106096500	1	
KMS-12-BM	14	106090500	106094500	14	106108000	106112000	7	0	2.28571429	4.57142857	17500	FALSE	FALSE	106090500_106094500_106108000_106112000	1	
KMS-12-BM	14	106102000	106106000	14	106104500	106108500	2311	15	14.4521852	14.3601732	2500	FALSE	FALSE	106102000_106106000_106104500_106108500	4	
KMS-12-BM	14	106106500	106110500	14	106108000	106112000	4968	31	43.218599	43.2055567	1500	TRUE	FALSE	106106500_106110500_106108000_106112000	2	
KMS-12-BM	14	106209000	106213000	14	106238500	106242500	16	0	9.1875	36.625	29500	TRUE	TRUE	106209000_106213000_106238500_106242500	2	
KMS-12-BM	14	106820000	106824000	14	107265500	107269500	1	0	0	60	445500	FALSE	FALSE	106820000_106824000_107265500_107269500	1	
KMS-12-BM	14	107262000	107266000	14	107265500	107269500	11	14	47.4545455	49.8181818	3500	TRUE	FALSE	107262000_107266000_107265500_107269500	15	
KMS-12-BM	14	107268500	107272500	14	107271500	107275500	249	372	47.4297189	53.3815261	3000	TRUE	FALSE	107268500_107272500_107271500_107275500	15	
KMS-12-BM	14	106026686	106026796	14	106027000	106031000	3	0	0	3	2259	FALSE	FALSE	106026686_106026796_106027000_106031000	1	
KMS-12-BM	14	106031133	106031292	14	106031500	106035500	2	0	23.2857143	24.4285714	2287.5	FALSE	FALSE	106031133_106031292_106031500_106035500	1	
KMS-12-BM	14	106036500	106040500	14	106040702	106040819	18	2	30.6666667	30.2777778	2260.5	TRUE	FALSE	106036500_106040500_106040702_106040819	1	
KMS-12-BM	14	106036500	106040500	14	106157594	106157680	2	0	42.5	0	119137	FALSE	FALSE	106036500_106040500_106157594_106157680	1	
KMS-12-BM	14	106036500	106040500	14	106158163	106158499	6	0	24.1666667	1	119831	FALSE	FALSE	106036500_106040500_106158163_106158499	1	
KMS-12-BM	14	106036500	106040500	14	106159182	106159471	2	0	4.5	0	120826.5	FALSE	FALSE	106036500_106040500_106159182_106159471	1	
KMS-12-BM	14	106047500	106051500	14	106168134	106168218	4	0	40	8	118676	TRUE	FALSE	106047500_106051500_106168134_106168218	1	
KMS-12-BM	14	106047500	106051500	14	106169507	106169746	9	0	32.3333333	0	120126.5	FALSE	FALSE	106047500_106051500_106169507_106169746	1	
KMS-12-BM	14	106047500	106051500	14	106170425	106170505	2	0	0	0	120965	FALSE	FALSE	106047500_106051500_106170425_106170505	1	
KMS-12-BM	14	106047500	106051500	14	106171694	106171801	4	0	7.25	22.75	122247.5	FALSE	FALSE	106047500_106051500_106171694_106171801	1	
KMS-12-BM	14	106078500	106082500	14	106082703	106082842	16	0	47.1875	49.6875	2272.5	TRUE	TRUE	106078500_106082500_106082703_106082842	1	
KMS-12-BM	14	106078500	106082500	14	106099723	106100053	3	1	9.66666667	24.3333333	19388	FALSE	FALSE	106078500_106082500_106099723_106100053	1	
KMS-12-BM	14	106078500	106082500	14	106100378	106100642	3	0	3	13	20010	FALSE	FALSE	106078500_106082500_106100378_106100642	1	
KMS-12-BM	14	106078500	106082500	14	106101005	106101740	9	0	4.22222222	1.44444444	20872.5	FALSE	FALSE	106078500_106082500_106101005_106101740	1	
KMS-12-BM	14	106078500	106082500	14	106125631	106125845	2	0	29.5	0	45238	FALSE	FALSE	106078500_106082500_106125631_106125845	1	
KMS-12-BM	14	106078500	106082500	14	106195313	106195555	3	0	35	4.33333333	114934	TRUE	FALSE	106078500_106082500_106195313_106195555	1	
KMS-12-BM	14	106090500	106094500	14	106112500	106112925	12	0	13.8333333	17.5833333	20212.5	FALSE	TRUE	106090500_106094500_106112500_106112925	1	
KMS-12-BM	14	106090500	106094500	14	106135761	106135978	2	0	16	0	43369.5	FALSE	FALSE	106090500_106094500_106135761_106135978	1	
KMS-12-BM	14	106090500	106094500	14	106208542	106208615	2	0	12.5	11	116078.5	FALSE	FALSE	106090500_106094500_106208542_106208615	1	
KMS-12-BM	14	106090500	106094500	14	106209878	106211007	23	0	3.91304348	15.2173913	117942.5	FALSE	TRUE	106090500_106094500_106209878_106211007	1	
KMS-12-BM	14	106090500	106094500	14	106238005	106238082	3	0	35	0	145543.5	FALSE	FALSE	106090500_106094500_106238005_106238082	1	
KMS-12-BM	14	106102000	106106000	14	106128035	106128497	5	0	0	3	24266	FALSE	FALSE	106102000_106106000_106128035_106128497	1	
KMS-12-BM	14	106102000	106106000	14	106129409	106129500	2	0	5	6	25454.5	FALSE	FALSE	106102000_106106000_106129409_106129500	1	
KMS-12-BM	14	106102000	106106000	14	106200454	106200611	2	0	46.5	0	96532.5	FALSE	FALSE	106102000_106106000_106200454_106200611	1	
KMS-12-BM	14	106102000	106106000	14	106200815	106201388	5	1	15.2	8	97101.5	FALSE	FALSE	106102000_106106000_106200815_106201388	1	
KMS-12-BM	14	106102000	106106000	14	106202512	106202712	3	0	1.66666667	2	98612	FALSE	FALSE	106102000_106106000_106202512_106202712	1	
KMS-12-BM	14	106102000	106106000	14	106229317	106229527	3	0	0	0	125422	FALSE	FALSE	106102000_106106000_106229317_106229527	1	
KMS-12-BM	14	106102000	106106000	14	106230306	106230400	2	0	5.5	0	126353	FALSE	FALSE	106102000_106106000_106230306_106230400	1	
KMS-12-BM	14	106102000	106106000	14	106230728	106231087	4	0	8.5	4.5	126907.5	FALSE	FALSE	106102000_106106000_106230728_106231087	1	
KMS-12-BM	14	106086157	106086752	14	106104500	106108500	11	1	5.27272727	3.09090909	20045.5	FALSE	FALSE	106086157_106086752_106104500_106108500	1	
KMS-12-BM	14	106087144	106087413	14	106104500	106108500	2	0	0	0	19221.5	FALSE	FALSE	106087144_106087413_106104500_106108500	1	
KMS-12-BM	14	106106500	106110500	14	106133193	106133313	3	0	45	0	24753	FALSE	FALSE	106106500_106110500_106133193_106133313	1	
KMS-12-BM	14	106106500	106110500	14	106204163	106204281	2	0	23.5	6.5	95722	FALSE	FALSE	106106500_106110500_106204163_106204281	1	
KMS-12-BM	14	106106500	106110500	14	106206905	106207097	6	0	0	0	98501	FALSE	FALSE	106106500_106110500_106206905_106207097	1	
KMS-12-BM	14	106106500	106110500	14	106207448	106207663	4	1	0	7.25	99055.5	FALSE	FALSE	106106500_106110500_106207448_106207663	1	
KMS-12-BM	14	106106500	106110500	14	106208603	106208698	2	0	41.5	0	100150.5	FALSE	FALSE	106106500_106110500_106208603_106208698	1	
KMS-12-BM	14	106106500	106110500	14	106231774	106231940	3	0	0	0	123357	FALSE	FALSE	106106500_106110500_106231774_106231940	1	
KMS-12-BM	14	106106500	106110500	14	106234655	106234863	9	0	9.77777778	0.66666667	126259	FALSE	FALSE	106106500_106110500_106234655_106234863	1	
KMS-12-BM	14	106089636	106089923	14	106108000	106112000	4	0	25.5	22	20220.5	FALSE	FALSE	106089636_106089923_106108000_106112000	1	
KMS-12-BM	14	106090169	106090371	14	106108000	106112000	2	0	0	0	19730	FALSE	FALSE	106090169_106090371_106108000_106112000	1	
KMS-12-BM	14	106111000	106115000	14	106209761	106210098	5	0	0	9.8	96929.5	FALSE	FALSE	106111000_106115000_106209761_106210098	1	
KMS-12-BM	14	106111000	106115000	14	106210736	106211061	4	0	12.5	4	97898.5	FALSE	FALSE	106111000_106115000_106210736_106211061	1	
KMS-12-BM	14	106111000	106115000	14	106237881	106238095	7	0	7.42857143	15.8571429	124988	FALSE	FALSE	106111000_106115000_106237881_106238095	1	
KMS-12-BM	14	106040697	106040771	14	106161000	106165000	2	1	27	12	122266	FALSE	FALSE	106040697_106040771_106161000_106165000	1	
KMS-12-BM	14	106045312	106046656	14	106164000	106168000	19	1	2.10526316	3.26315789	120016	FALSE	FALSE	106045312_106046656_106164000_106168000	1	
KMS-12-BM	14	106056729	106057174	14	106176000	106180000	3	0	6.66666667	4	121048.5	FALSE	FALSE	106056729_106057174_106176000_106180000	1	
KMS-12-BM	14	106057711	106057785	14	106176000	106180000	2	0	50	0	120252	FALSE	FALSE	106057711_106057785_106176000_106180000	1	
KMS-12-BM	14	106058367	106058859	14	106176000	106180000	8	0	11.2857143	5.57142857	119387	FALSE	FALSE	106058367_106058859_106176000_106180000	1	
KMS-12-BM	14	106063406	106064078	14	106184500	106188500	9	0	0	10.5555556	122758	FALSE	FALSE	106063406_106064078_106184500_106188500	1	
KMS-12-BM	14	106064294	106064947	14	106184500	106188500	11	0	4.9	11.2	121879.5	FALSE	TRUE	106064294_106064947_106184500_106188500	1	
KMS-12-BM	14	106065217	106066149	14	106184500	106188500	7	0	5.42857143	6.71428571	120817	FALSE	FALSE	106065217_106066149_106184500_106188500	1	
KMS-12-BM	14	106184167	106184263	14	106184500	106188500	2	0	29	0	2285	FALSE	FALSE	106184167_106184263_106184500_106188500	1	
KMS-12-BM	14	106100884	106101456	14	106223500	106227500	6	0	14.5	2.5	124330	FALSE	FALSE	106100884_106101456_106223500_106227500	1	
KMS-12-BM	14	106096088	106096319	14	106238500	106242500	2	0	27	5.5	144296.5	FALSE	FALSE	106096088_106096319_106238500_106242500	1	
KMS-12-BM	14	106213097	106213320	14	106238500	106242500	10	0	28	0	27291.5	FALSE	TRUE	106213097_106213320_106238500_106242500	1	
KMS-12-BM	14	106213910	106213995	14	106238500	106242500	3	0	3.33333333	39	26547.5	TRUE	FALSE	106213910_106213995_106238500_106242500	1	
KMS-12-BM	14	106240872	106241110	14	106325000	106329000	7	0	0	60	86009	FALSE	FALSE	106240872_106241110_106325000_106329000	1	
KMS-12-BM	14	106328000	106332000	14	106845396	106845457	2	0	48	0	515426.5	FALSE	FALSE	106328000_106332000_106845396_106845457	1	
KMS-12-BM	14	106355500	106359500	14	106539077	106539153	5	0	42.6	0	181615	FALSE	FALSE	106355500_106359500_106539077_106539153	1	
KMS-12-BM	14	106355500	106359500	14	106967049	106967123	3	0	54.6666667	0	609586	FALSE	FALSE	106355500_106359500_106967049_106967123	1	
KMS-12-BM	14	106774681	106774769	14	106775000	106779000	2	3	60	60	2275	TRUE	FALSE	106774681_106774769_106775000_106779000	1	
KMS-12-BM	14	106788500	106792500	14	106813362	106813951	12	7	12.6666667	9.66666667	23156.5	FALSE	FALSE	106788500_106792500_106813362_106813951	1	
KMS-12-BM	14	106788500	106792500	14	106814425	106814554	2	2	0	35.5	23989.5	FALSE	FALSE	106788500_106792500_106814425_106814554	1	
KMS-12-BM	14	106788500	106792500	14	106814760	106815128	3	7	0	0	24444	FALSE	FALSE	106788500_106792500_106814760_106815128	1	
KMS-12-BM	14	106788500	106792500	14	106816000	106816074	2	4	0	0	25537	FALSE	FALSE	106788500_106792500_106816000_106816074	1	
KMS-12-BM	14	106820000	106824000	14	106824210	106824324	16	16	21.75	21.75	2267	FALSE	FALSE	106820000_106824000_106824210_106824324	1	

KMS-12-BM	14	106820000	106824000	14	107104826	107104900	2	2	5	5	282863	FALSE	FALSE	106820000_106824000_107104826_107104900	1	
KMS-12-BM	14	106539079	106539163	14	107168000	107172000	4	0	0	27.4	630879	FALSE	FALSE	106539079_106539163_107168000_107172000	1	
KMS-12-BM	14	106967049	106967123	14	107168000	107172000	2	0	0	42.6666667	202914	FALSE	FALSE	106967049_106967123_107168000_107172000	1	
KMS-12-BM	14	107093188	107093528	14	107271500	107275500	8	0	55.125	42.125	180142	TRUE	FALSE	107093188_107093528_107271500_107275500	1	
L-363	14	106098500	106102500	14	106125666	106125849	2	0	29.5	6.5	25257.5	FALSE	FALSE	106098500_106102500_106125666_106125849	1	
L-363	14	106098500	106102500	14	106226593	106226663	2	0	0	0	126128	FALSE	FALSE	106098500_106102500_106226593_106226663	1	
L-363	14	106084261	106084456	14	106103500	106107500	11	0	10.8181818	6.45454545	21141.5	FALSE	TRUE	106084261_106084456_106103500_106107500	1	
L-363	14	106085150	106085333	14	106103500	106107500	3	0	0	0	20258.5	FALSE	FALSE	106085150_106085333_106103500_106107500	1	
L-363	14	106087739	106088028	14	106103500	106107500	2	0	14.5	4	17616.5	FALSE	FALSE	106087739_106088028_106103500_106107500	1	
L-363	14	106070603	106070688	14	106189000	106193000	6	1	11.8	0	120354.5	FALSE	FALSE	106070603_106070688_106189000_106193000	1	
L-363	14	106121422	106121909	14	106189000	106193000	3	2	10.3333333	12.3333333	69334.5	FALSE	FALSE	106121422_106121909_106189000_106193000	1	
L-363	14	106203000	106207000	14	106234757	106234844	2	0	0	0	29800.5	FALSE	FALSE	106203000_106207000_106234757_106234844	1	
L-363	14	106778220	106778294	14	106778500	106782500	2	13	60	60	2243	TRUE	FALSE	106778220_106778294_106778500_106782500	1	
LP-1	14	106072000	106076000	14	106076000	106080000	171	29	26.7777778	27.0818713	4000	FALSE	FALSE	106072000_106076000_106076000_106080000	17	
LP-1	14	106072000	106076000	14	106095000	106099000	1	0	0	60	23000	FALSE	FALSE	106072000_106076000_106095000_106099000	1	
LP-1	14	106134500	106138500	14	106137500	106141500	1164	84	28.8075601	27.083405	3000	FALSE	FALSE	106134500_106138500_106137500_106141500	3	
LP-1	14	107262000	107266000	14	107265500	107269500	5	14	60	48	3500	TRUE	FALSE	107262000_107266000_107265500_107269500	15	
LP-1	14	107268500	107272500	14	107271500	107275500	394	372	46.5888325	52.2918782	3000	TRUE	FALSE	107268500_107272500_107271500_107275500	15	
LP-1	14	106072000	106076000	14	106119118	106119413	9	0	10.1111111	5	45265.5	FALSE	FALSE	106072000_106076000_106119118_106119413	1	
LP-1	14	106072000	106076000	14	106120731	106120992	2	0	0	4	46861.5	FALSE	FALSE	106072000_106076000_106120731_106120992	1	
LP-1	14	106072000	106076000	14	106121249	106121935	4	2	15	6	47592	FALSE	FALSE	106072000_106076000_106121249_106121935	1	
LP-1	14	106072000	106076000	14	106218011	106218073	2	0	19	0	144042	FALSE	FALSE	106072000_106076000_106218011_106218073	1	
LP-1	14	106072000	106076000	14	106218639	106218770	2	1	0	5	144704.5	FALSE	FALSE	106072000_106076000_106218639_106218770	1	
LP-1	14	106094679	106094796	14	106095000	106099000	4	0	54.0769231	52.2307692	2262.5	TRUE	FALSE	106094679_106094796_106095000_106099000	1	
LP-1	14	106098500	106102500	14	106125521	106125859	4	0	45	0	25190	FALSE	FALSE	106098500_106102500_106125521_106125859	1	
LP-1	14	106098500	106102500	14	106197023	106197592	9	0	5.77777778	10.1111111	96807.5	FALSE	FALSE	106098500_106102500_106197023_106197592	1	
LP-1	14	106098500	106102500	14	106198115	106198251	2	0	12.5	20	97683	FALSE	FALSE	106098500_106102500_106198115_106198251	1	
LP-1	14	106098500	106102500	14	106226571	106226631	2	0	3.5	14.5	126101	FALSE	FALSE	106098500_106102500_106226571_106226631	1	
LP-1	14	106085758	106086065	14	106129000	106133000	2	0	30	0	45088.5	FALSE	FALSE	106085758_106086065_106129000_106133000	1	
LP-1	14	106088193	106088271	14	106129000	106133000	4	0	0	12.1	42768	FALSE	FALSE	106088193_106088271_106129000_106133000	1	
LP-1	14	106012072	106013265	14	106137500	106141500	16	7	11.0625	0.8125	126831.5	FALSE	FALSE	106012072_106013265_106137500_106141500	1	
LP-1	14	106013638	106013917	14	106137500	106141500	2	1	5	22	125722.5	FALSE	FALSE	106013638_106013917_106137500_106141500	1	
LP-1	14	106035708	106035977	14	106154000	106158000	25	19	21.2916667	33	120157.5	TRUE	FALSE	106035708_106035977_106154000_106158000	1	
LP-1	14	106153657	106153794	14	106154000	106158000	4	0	58.2	42	2274.5	TRUE	FALSE	106153657_106153794_106154000_106158000	1	
LP-1	14	106045369	106047015	14	106164000	106168000	27	1	1.37037037	6.5	119808	FALSE	FALSE	106045369_106047015_106164000_106168000	1	
LP-1	14	106048403	106048458	14	106164000	106168000	3	0	0	47	117569.5	FALSE	FALSE	106048403_106048458_106164000_106168000	1	
LP-1	14	106163723	106163799	14	106164000	106168000	2	1	12.0909091	11.7272727	2239	FALSE	FALSE	106163723_106163799_106164000_106168000	1	
LP-1	14	106165500	106169500	14	106169728	106169883	5	0	33.2	41	2305.5	TRUE	FALSE	106165500_106169500_106169728_106169883	1	
LP-1	14	106173000	106177000	14	106177657	106177852	10	0	55.7	49.1	2754.5	TRUE	TRUE	106173000_106177000_106177657_106177852	1	
LP-1	14	106070612	106070780	14	106189000	106193000	9	1	4.375	0	120304	FALSE	FALSE	106070612_106070780_106189000_106193000	1	
LP-1	14	106094181	106094259	14	106325000	106329000	2	0	0	60	232780	FALSE	FALSE	106094181_106094259_106325000_106329000	1	
IM-9	14	106072000	106076000	14	106076000	106080000	206	29	22.6990291	22.5048544	4000	FALSE	FALSE	106072000_106076000_106076000_106080000	17	
IM-9	14	106072000	106076000	14	106119129	106119407	5	0	8.2	5.2	45268	FALSE	FALSE	106072000_106076000_106119129_106119407	1	
LP-1	14	106328000	106332000	14	106815758	106815842	7	0	60	0	485800	FALSE	FALSE	106328000_106332000_106815758_106815842	1	
LP-1	14	106328000	106332000	14	107131035	107131109	3	0	60	0	801072	FALSE	FALSE	106328000_106332000_107131035_107131109	1	
LP-1	14	106431142	106431294	14	106431500	106435500	3	5	40.6666667	37.3333333	2282	TRUE	FALSE	106431142_106431294_106431500_106435500	1	
LP-1	14	106701695	106701769	14	106702000	106706000	2	6	58	46.3333333	2268	TRUE	FALSE	106701695_106701769_106702000_106706000	1	
LP-1	14	106725500	106729500	14	106729707	106729791	8	5	60	60	2249	TRUE	FALSE	106725500_106729500_106729707_106729791	1	
LP-1	14	106777638	106777785	14	106778000	106782000	4	6	60	56.5833333	2288.5	TRUE	FALSE	106777638_106777785_106778000_106782000	1	
LP-1	14	106799182	106799231	14	107271500	107275500	2	0	17	2	474293.5	FALSE	FALSE	106799182_106799231_107271500_107275500	1	
LP-1	14	107093177	107093536	14	107271500	107275500	7	0	57.2857143	40.7142857	180143.5	TRUE	FALSE	107093177_107093536_107271500_107275500	1	
OPM-2	14	106023000	106027000	14	106143959	106144215	3	0	0	1.66666667	119087	FALSE	FALSE	106023000_106027000_106143959_106144215	1	
OPM-2	14	106023000	106027000	14	106144606	106144887	3	0	20.3333333	0	119746.5	FALSE	FALSE	106023000_106027000_106144606_106144887	1	
OPM-2	14	106023000	106027000	14	106145525	106146453	13	0	12.6923077	2.66666667	120989	FALSE	TRUE	106023000_106027000_106145525_106146453	1	
OPM-2	14	106023000	106027000	14	106147149	106147410	3	0	0	6	122279.5	FALSE	FALSE	106023000_106027000_106147149_106147410	1	
OPM-2	14	106047500	106051500	14	106169521	106169581	2	0	26	0	120051	FALSE	FALSE	106047500_106051500_106169521_106169581	1	
OPM-2	14	106047500	106051500	14	106171477	106171778	2	0	6	7	122127.5	FALSE	FALSE	106047500_106051500_106171477_106171778	1	
OPM-2	14	106053000	106057000	14	106057339	106057578	2	0	0	30.5	2458.5	FALSE	FALSE	106053000_106057000_106057339_106057578	1	
OPM-2	14	106053000	106057000	14	106174529	106174603	2	0	43.5	0	119566	FALSE	FALSE	106053000_106057000_106174529_106174603	1	
OPM-2	14	106053000	106057000	14	106188881	106188906	3	0	6.33333333	10.3333333	133893.5	FALSE	FALSE	106053000_106057000_106188881_106188906	1	
OPM-2	14	106063239	106063492	14	106184500	106188500	2	0	20	0	123134.5	FALSE	FALSE	106063239_106063492_106184500_106188500	1	
OPM-2	14	106063852	106064086	14	106184500	106188500	2	0	8.5	0	122531	FALSE	FALSE	106063852_106064086_106184500_106188500	1	
OPM-2	14	106099117	106099230	14	106192000	106196000	2	0	0	39	94826.5	FALSE	FALSE	106099117_106099230_106192000_106196000	1	
OPM-2	14	106106500	106110500	14	106133811	106133912	3	0	0	5.33333333	25361.5	FALSE	FALSE	106106500_106110500_106133811_106133912	1	
OPM-2	14	106106500	106110500	14	106206904	106207010	2	0	0	0	98457	FALSE	FALSE	106106500_106110500_106206904_106207010	1	
OPM-2	14	106111000	106115000	14	106209775	106209957	3	0	0	11	96866	FALSE	FALSE	106111000_106115000_106209775_106209957	1	
OPM-2	14	106111000	106115000	14	106210737	106211063	3	0	9.66666667	4.66666667	97900	FALSE	FALSE	106111000_106115000_106210737_106211063	1	
IM-9	14	106211258	106211923	14	106324500	106328500	79	0	19.220339	60	114909.5	TRUE	TRUE	106211258_106211923_106324500_106328500	1	TRUE
IM-9	14	106328500	106332500	14	106550000	106554000	30	0	58.4666667	31	221500	TRUE	TRUE	106328500_106332500_106550000_106554000	1	TRUE
OPM-2	14	106184109	106184219	14	106184500	106188500	2	0	37	0	2336	FALSE	FALSE	106184109_106184219_106184500_106188500	1	
OPM-2	14	106191672	106191797	14	106192000	106196000	7	1	30.6363636	24.6363636	2265.5	TRUE	FALSE	106191672_106191797_106192000_106196000	1	
OPM-2	14	106328500	106332500	14	106377500	106381500	16	0	27.6875	20.5	49000	TRUE	TRUE	106328500_106332500_106377500_106381500	1	TRUE
SK-MM-2	14	106070583	106070788	14	106189000	106193000	10	1	4	0	120314.5	FALSE	FALSE	106070583_106070788_106189000_106193000	1	
SK-MM-2	14	106072000	106076000	14	106076000	106080000	164	29	23.4268293	23.3780488	4000	FALSE	FALSE	106072000_106076000_106076000_106080000	17	
SK-MM-2	14	106072000	106076000	14	106119240	106119361	3	0	16.3333333	0	45300.5	FALSE	FALSE	106072000_106076000_106119240_106119361	1	
SK-MM-2	14	106072000	106076000	14	106119659	106119869	2	0	0	13	45764	FALSE	FALSE	106072000_106076000_106119659_106119869	1	
SK-MM-2	14	106072000	106076000	14	106120385	106120672	2	0	3	5	46528.5	FALSE	FALSE	106072000_106076000_106120385_106120672	1	
SK-MM-2	14	106072000	106076000	14	106121196	106121435	3	0	0	0	47315.5	FALSE	FALSE	106072000_106076000_106121196_106121435	1	
SK-MM-2	14	106072000	106076000	14	106218004	106218260	3	0	2.66666667	6	144132	FALSE	FALSE	106072000_106076000_106218004_106218260	1	
SK-MM-2	14	106122013	106122259	14	106189000	106193000	2	1	0	21	68864	FALSE	FALSE	106122013_106122259_106189000_106193000	1	
SK-MM-2	14	106134500	106138500	14	106237903	106238129	3	0	6	15.3333333	101516	FALSE	FALSE	106134500_106138500_106237903_106238129	1	
SK-MM-2	14	106529500	106533500	14	107008913	107008962	2	0	4.5	7	477437.5	FALSE	FALSE	106529500_106533500_107008913_107008962	1	
U-266	14	106175000	106179000	14	106324000	106328000	16	0	32	56.5	149000	TRUE	TRUE	106175000_106179000_106324000_106328000	2	
U-266	14	106327500	106331500	14	106451000	106455000	4	0	58.5	26.75	123500	TRUE	FALSE	106327500_106331500_106451000_106455000	1	
U-266	14	107262000	107266000	14	107265500	107269500	1	14	60	60	3500	TRUE	FALSE	107262000_107266000_107265500_107269500	15	
U-266	14	107268500	107272500	14	107271500	107275500	208	372	47.3701923	52.5865385	3000	TRUE	FALSE	107268500_107272500_107271500_107275500	15	
U-266	14	106057500	106061500	14	106061701	106061775	5	0	0	0	2238	FALSE	FALSE	106057500_106061500_106061701_106061775	1	
U-266	14	106057500	106061500	14	106178860	106179010	2	0	50.5	5.5	119435	TRUE	FALSE	106057500_106061500_106178860_106179010	1	
U-266	14	106057500	106061500	14	106179652	106179940	2	0	7.5	10	120296	FALSE	FALSE	106057500_106061500_106179652_106179940	1	
U-266	14	106057500	106061500	14	106180850	106180991	3	0	1.33333333	0	121420.5	FALSE	FALSE	106057500_106061500_106180850_106180991	1	
U-266	14	106082500	106086500	14	106103682	106103859	9	0	10.5555556	8.44444444	19270.5	FALSE	FALSE	106082500_106086500_106103682_106103859	1	
U-266	14	106082500	106086500	14	106200470	106200597	7	0	12	0	116033.5	FALSE	FALSE	106082500_106086500_106200470_106200597	1	
U-266	14	106082500	106086500	14	106227202	106227312	17	5	46.8823529	55.3529412	142757	TRUE	FALSE	106082500_106086500_106227202_106227312	1	
U-266	14	106082500	106086500	14	106228383	106228641	3	0	60	29.3333333	144012	TRUE	FALSE	106082500_106086500_106228383_106228641	1	
U-266	14	106089653	106089786	14	106104500	106108500	3	0	43.3333333	27	16780.5	TRUE	FALSE	106089653_106089786_106104500_106108500	1	
U-266	14	106465180	106465274	14	106465500	106469500	2	1	53.7142857	60	2273	TRUE	FALSE	106465180_106465274_106465500_106469500	1	
U-266	14	106788500	106792500	14	106813332	106813810	9	6	9.33333333	3	23071	FALSE	FALSE	106788500_106792500_106813332_106813810	1	
U-266	14	106788500	106792500	14	106814067	106814845	8	12	11.5	8.375	23956	FALSE	FALSE	106788500_106792500_106814067_106814845	1	
U-266	14	106788500	106792500	14	106815073	106815176	2	0	14.5	0	24624.5	FALSE	FALSE	106788500_106792500_106815073_106815176	1	
U-266	14	106788500	106792500	14	106815960	106816254	2	5	6	0	25607	FALSE	FALSE	106788500_106792500_106815960_106816254	1	
U-266	14	106788500	106792500	14	106816505	106816621	3	9	0	11.6666667	26063	FALSE	FALSE	106788500_106792500_106816505_106816621	1	
U-266	14	106788500	106792500	14	106816920	106817203	4	1	0	15.8	26561.5	FALSE	FALSE	106788500_106792500_106816920_106817203	1	
U-266	14	106788500	106792500	14	107099857	107099931	2	1	0	60	309394	FALSE	FALSE	106788500_106792500_107099857_107099931	1	
U-266	14	106796135	106796280	14	106796500	106800500	3	4	47.5	39.2	2292.5	TRUE	FALSE	106796135_106796280_106796500_106800500	1	
U-266	14	106852000	106856000	14	106935319	106935531	5	10	35.8	10	81425	TRUE	FALSE	106852000_106856000_106935319_106935531	1	
U-266	14	106852000	106856000	14	106935811	106935954	2	0	6	6	81882.5	FALSE	FALSE	106852000_106856000_106935811_106935954	1	
U-266	14	106852000	106856000	14	106936366	106936462	3	0	10.3333333	16.6666667	82414	FALSE	FALSE	106852000_106856000_106936366_106936462	1	
U-266	14	106852000	106856000	14	106936787	106936964	2	0	34.5	26	82875.5	TRUE	FALSE	106852000_106856000_106936787_106936964	1	
U-266	14	107186000	107190000	14	107190218	107190297	7	16	60	60	2257.5	TRUE	FALSE	107186000_107190000_107190218_107190297	1	
U-266	14	107106958	107107101	14	107271500	107275500	2	0	42.5	31	166470.5	TRUE	FALSE	107106958_107107101_107271500_107275500	1	
IM-9	14	106072000	106076000	14	106120666	106120839	2	0	0	0	46752.5	FALSE	FALSE	106072000_106076000_106120666_106120839	1	
IM-9	14	106072000	106076000	14	106121361	106121542	2	0	1	0.5	47451.5	FALSE	FALSE	106072000_106076000_106121361_106121542	1	
IM-9	14	106072000	106076000	14	106217491	106218187	6	1	4.83333333	5.42857143	143839	FALSE	FALSE	106072000_106076000_106217491_106218187	1	
IM-9	14	106072000	106076000	14	106218697	106218978	3	1	0	0	144837.5	FALSE	FALSE	106072000_106076000_106218697_106218978	1	
IM-9	14	106072000	106076000	14	106219398	106219584	3	0	0	0	145491	FALSE	FALSE	106072000_106076000_106219398_106219584	1	
IM-9	14	106092189	106092454	14	106110500	106114500	3	0	7.33333333	2.33333333	20178.5	FALSE	FALSE	106092189_106092454_106110500_106114500	1	
IM-9	14	106092785	106093111	14	106110500	106114500	3	0	0	0	19552	FALSE	FALSE	106092785_106093111_106110500_106114500	1	
IM-9	14	106093578	106093675	14	106110500	106114500	3	0	3	9	18873.5	FALSE	FALSE	106093578_106093675_106110500_106114500	1	
IM-9	14	106094558	106094843	14	106110500	106114500	3	0	17.6666667	0	17799.5	FALSE	FALSE	106094558_106094843_106110500_106114500	1	
IM-9	14	106134500	106138500	14	106235664	106235857	2	0	36	2.5	99260.5	TRUE	FALSE	106134500_106138500_106235664_106235857	1	
IM-9	14	106134500	106138500	14	106237728	106237989	4	0	8	19	101358.5	FALSE	FALSE	106134500_106138500_106237728_106237989	1	
IM-9	14	106220000	106224000	14	106224202	106224308	18	1	40.8333333	58.2777778	2255	TRUE	FALSE	106220000_106224000_106224202_106224308	1	
IM-9	14	106328500	106332500	14	106348000	106352000	27	0	54.0740741	42.6666667	19500	TRUE	TRUE	106328500_106332500_106348000_106352000	2	
IM-9	14	106328500	106332500	14	106359434	106359516	4	0	57	0	28975	FALSE	FALSE	106328500_106332500_106359434_106359516	1	

IM-9	14	106328500	106332500	14	106667613	106667675	2	0	60	0	337144	FALSE	FALSE	106328500_106332500_106667613_106667675	1	
IM-9	14	106549693	106549798	14	106550000	106554000	2	29	60	60	2254.5	TRUE	FALSE	106549693_106549798_106550000_106554000	1	
IM-9	14	106711685	106711771	14	106712000	106716000	2	17	60	60	2272	TRUE	FALSE	106711685_106711771_106712000_106716000	1	
IM-9	14	106714179	106714293	14	106714500	106718500	2	19	41.2222222	44.5555556	2264	TRUE	FALSE	106714179_106714293_106714500_106718500	1	
IM-9	14	106725500	106729500	14	106729748	106729844	6	1	58.5	60	2296	TRUE	FALSE	106725500_106729500_106729748_106729844	1	
IM-9	14	106777589	106777741	14	106778000	106782000	2	1	60	57.7142857	2335	TRUE	FALSE	106777589_106777741_106778000_106782000	1	
IM-9	14	106789039	106789222	14	106814500	106818500	3	2	0.6	0	27369.5	FALSE	FALSE	106789039_106789222_106814500_106818500	1	
IM-9	14	106789500	106793500	14	106814500	106818500	19	39	4.21052632	5.15	25000	FALSE	FALSE	106789500_106793500_106814500_106818500	1	
IM-9	14	106789500	106793500	14	106817000	106821000	7	11	1.42857143	4	27500	FALSE	FALSE	106789500_106793500_106817000_106821000	1	
IM-9	14	106789500	106793500	14	106793701	106793882	32	4	9.5625	9.5625	2291.5	FALSE	FALSE	106789500_106793500_106793701_106793882	1	
IM-9	14	106794532	106794606	14	106817000	106821000	3	0	38.6666667	7.66666667	24431	TRUE	FALSE	106794532_106794606_106817000_106821000	1	
IM-9	14	106795014	106795078	14	106817000	106821000	2	0	12	0	23954	FALSE	FALSE	106795014_106795078_106817000_106821000	1	
IM-9	14	106795367	106795539	14	106817000	106821000	4	3	0	5	23547	FALSE	FALSE	106795367_106795539_106817000_106821000	1	
IM-9	14	106796152	106796291	14	106796500	106800500	7	9	45.3428571	38.9117647	2278.5	TRUE	FALSE	106796152_106796291_106796500_106800500	1	
IM-9	14	106811000	106815000	14	106814500	106818500	182	476	5.16483516	5.15934066	3500	FALSE	FALSE	106811000_106815000_106814500_106818500	1	
IM-9	14	106815500	106819500	14	106817000	106821000	3665	1713	20.6016371	20.1200546	1500	FALSE	FALSE	106815500_106819500_106817000_106821000	4	
IM-9	14	106815500	106819500	14	106917000	106921000	1	0	0	57	101500	FALSE	FALSE	106815500_106819500_106917000_106921000	1	
IM-9	14	106815500	106819500	14	107101150	107101418	3	1	20.6666667	5.33333333	283784	FALSE	FALSE	106815500_106819500_107101150_107101418	1	
IM-9	14	106859500	106863500	14	106861500	106865500	85	80	40.2235294	48.4588235	2000	TRUE	FALSE	106859500_106863500_106861500_106865500	7	
IM-9	14	106859500	106863500	14	106862500	106866500	1	3	60	40	3000	TRUE	FALSE	106859500_106863500_106862500_106866500	7	
IM-9	14	106859500	106863500	14	106940635	106940922	3	1	0	13.6666667	79278.5	FALSE	FALSE	106859500_106863500_106940635_106940922	1	
IM-9	14	106881000	106885000	14	106884000	106888000	3	20	0	0	3000	FALSE	FALSE	106881000_106885000_106884000_106888000	5	
IM-9	14	106940500	106944500	14	106944701	106944990	9	2	60	60	2345.5	TRUE	FALSE	106940500_106944500_106944701_106944990	1	
IM-9	14	107093204	107093369	14	107271500	107275500	4	0	58.75	31	180213.5	TRUE	FALSE	107093204_107093369_107271500_107275500	1	
IM-9	14	107146970	107147297	14	107147500	107151500	5	4	60	60	2366.5	TRUE	FALSE	107146970_107147297_107147500_107151500	1	
IM-9	14	107182500	107186500	14	107186722	107186854	13	7	60	60	2288	TRUE	FALSE	107182500_107186500_107186722_107186854	1	
IM-9	14	107262000	107266000	14	107265500	107269500	5	14	60	44	3500	TRUE	FALSE	107262000_107266000_107265500_107269500	15	
IM-9	14	107268500	107272500	14	107271500	107275500	255	372	45.7215686	52.1098039	3000	TRUE	FALSE	107268500_107272500_107271500_107275500	15	
KMS-11	14	106042500	106046500	14	106045000	106049000	1515	46	4.31551155	4.14521452	2500	FALSE	FALSE	106042500_106046500_106045000_106049000	2	
KMS-11	14	106042500	106046500	14	106046500	106050500	57	0	2.80701754	2.98245614	4000	FALSE	TRUE	106042500_106046500_106046500_106050500	1	
KMS-11	14	106042500	106046500	14	106161500	106165500	1	0	0	0	119000	FALSE	FALSE	106042500_106046500_106161500_106165500	2	
KMS-11	14	106042500	106046500	14	106164000	106168000	18	1	1.11111111	1.83333333	121500	FALSE	FALSE	106042500_106046500_106164000_106168000	1	
KMS-11	14	106047500	106051500	14	106164000	106168000	3	0	9.66666667	40	116500	TRUE	FALSE	106047500_106051500_106164000_106168000	3	
KMS-11	14	106209000	106213000	14	106238500	106242500	1	0	0	0	29500	FALSE	FALSE	106209000_106213000_106238500_106242500	2	
KMS-11	14	106371000	106375000	14	106380500	106384500	9	0	35.4444444	47.4444444	9500	TRUE	FALSE	106371000_106375000_106380500_106384500	1	
KMS-11	14	106781000	106785000	14	106807000	106811000	2	12	0	14.5	26000	FALSE	FALSE	106781000_106785000_106807000_106811000	2	
KMS-11	14	106859500	106863500	14	106861500	106865500	50	80	40.74	48.88	2000	TRUE	FALSE	106859500_106863500_106861500_106865500	7	
KMS-11	14	106859500	106863500	14	106862500	106866500	1	3	44	27	3000	TRUE	FALSE	106859500_106863500_106862500_106866500	7	
KMS-11	14	106881000	106885000	14	106884000	106888000	2	20	5	5	3000	FALSE	FALSE	106881000_106885000_106884000_106888000	5	
KMS-11	14	106022500	106026500	14	106026707	106026782	12	0	0	0	2244.5	FALSE	TRUE	106022500_106026500_106026707_106026782	1	
KMS-11	14	106022500	106026500	14	106144119	106144193	2	0	0	43	119656	FALSE	FALSE	106022500_106026500_106144119_106144193	1	
KMS-11	14	106022500	106026500	14	106144827	106144901	2	0	27	0	120364	FALSE	FALSE	106022500_106026500_106144827_106144901	1	
KMS-11	14	106022500	106026500	14	106145552	106145628	2	0	6	0	121090	FALSE	FALSE	106022500_106026500_106145552_106145628	1	
KMS-11	14	106022500	106026500	14	106146135	106146481	4	0	5.25	0	121808	FALSE	FALSE	106022500_106026500_106146135_106146481	1	
KMS-11	14	106022500	106026500	14	106146827	106147077	3	0	0	0	122452	FALSE	FALSE	106022500_106026500_106146827_106147077	1	
KMS-11	14	106047500	106051500	14	106169515	106169585	6	0	29	0	120050	FALSE	FALSE	106047500_106051500_106169515_106169585	1	
KMS-11	14	106047500	106051500	14	106170003	106170142	3	0	2.33333333	11	120572.5	FALSE	FALSE	106047500_106051500_106170003_106170142	1	
KMS-11	14	106047500	106051500	14	106171136	106171254	6	0	12.1666667	0	121695	FALSE	FALSE	106047500_106051500_106171136_106171254	1	
KMS-11	14	106112000	106116000	14	106116400	106116546	3	0	51.3333333	38.6666667	2473	TRUE	FALSE	106112000_106116000_106116400_106116546	1	
KMS-11	14	106112000	106116000	14	106210697	106210773	3	0	2	26	96735	FALSE	FALSE	106112000_106116000_106210697_106210773	1	
KMS-11	14	106112000	106116000	14	106210987	106211193	4	0	18.25	0	97090	FALSE	FALSE	106112000_106116000_106210987_106211193	1	
KMS-11	14	106080651	106080898	14	106192500	106196500	3	0	9.66666667	11.3333333	113725.5	FALSE	FALSE	106080651_106080898_106192500_106196500	1	
KMS-11	14	106112000	106116000	14	106238500	106242500	114	0	33.5438596	56.8	126500	TRUE	TRUE	106112000_106116000_106238500_106242500	1	TRUE
KMS-11	14	106093621	106093678	14	106238500	106242500	2	0	5.5	0	146850.5	FALSE	FALSE	106093621_106093678_106238500_106242500	1	
KMS-11	14	106094864	106094938	14	106238500	106242500	2	0	0	60	145599	FALSE	FALSE	106094864_106094938_106238500_106242500	1	
KMS-11	14	106328500	106332500	14	106351911	106351992	4	0	42	0	21451.5	FALSE	FALSE	106328500_106332500_106351911_106351992	1	
KMS-11	14	106328500	106332500	14	106361519	106361600	4	0	48.75	0	31059.5	FALSE	FALSE	106328500_106332500_106361519_106361600	1	
KMS-11	14	106351918	106351992	14	106368500	106372500	2	0	0	60	18545	FALSE	FALSE	106351918_106351992_106368500_106372500	1	
KMS-11	14	106361527	106361601	14	106368500	106372500	2	0	0	53.6666667	8936	FALSE	FALSE	106361527_106361601_106368500_106372500	1	
KMS-11	14	106711722	106711796	14	106712000	106716000	2	26	60	60	2241	TRUE	FALSE	106711722_106711796_106712000_106716000	1	
KMS-11	14	106725500	106729500	14	106729711	106729819	9	5	59.5555556	60	2265	TRUE	FALSE	106725500_106729500_106729711_106729819	1	
KMS-11	14	106820681	106820790	14	106821000	106825000	3	7	28.5555556	26.6666667	2264.5	FALSE	FALSE	106820681_106820790_106821000_106825000	1	
KMS-11	14	106940500	106944500	14	106944715	106944823	8	1	60	60	2269	TRUE	FALSE	106940500_106944500_106944715_106944823	1	
KMS-11	14	106536863	106536960	14	106960500	106964500	2	0	38.5	30	425588.5	TRUE	FALSE	106536863_106536960_106960500_106964500	1	
MC-CAR	14	106072000	106076000	14	106076000	106080000	221	29	22.1809955	22.0452489	4000	FALSE	FALSE	106072000_106076000_106076000_106080000	17	
MC-CAR	14	106082500	106086500	14	106084000	106088000	3790	114	30.4994723	28.8976253	1500	TRUE	FALSE	106082500_106086500_106084000_106088000	5	
MC-CAR	14	106082500	106086500	14	106104500	106108500	5	0	12	2.4	22000	FALSE	FALSE	106082500_106086500_106104500_106108500	2	
KMS-11	14	106328500	106332500	14	106368500	106372500	11	0	32.5454545	45.3636364	40000	TRUE	TRUE	106328500_106332500_106368500_106372500	1	TRUE
KMS-11	14	106209000	106213000	14	106598306	106598385	12	0	7.33333333	43.5	387345.5	TRUE	TRUE	106209000_106213000_106598306_106598385	1	TRUE
KMS-12-BM	14	106090500	106094500	14	106238500	106242500	16	0	19.25	37.875	148000	TRUE	TRUE	106090500_106094500_106238500_106242500	1	TRUE
KMS-12-BM	14	106111000	106115000	14	106176000	106180000	23	0	51.9565217	40.826087	65000	TRUE	TRUE	106111000_106115000_106176000_106180000	1	TRUE
MC-CAR	14	107262000	107266000	14	107265500	107269500	4	14	60	45	3500	TRUE	FALSE	107262000_107266000_107265500_107269500	15	
MC-CAR	14	107268500	107272500	14	107271500	107275500	254	372	47.2322835	53.4606299	3000	TRUE	FALSE	107268500_107272500_107271500_107275500	15	
MC-CAR	14	106072000	106076000	14	106119139	106119723	7	0	3.85714286	7	45431	FALSE	FALSE	106072000_106076000_106119139_106119723	1	
MC-CAR	14	106072000	106076000	14	106120758	106121010	3	0	0	16.6666667	46884	FALSE	FALSE	106072000_106076000_106120758_106121010	1	
MC-CAR	14	106072000	106076000	14	106218037	106218089	2	0	14	0	144063	FALSE	FALSE	106072000_106076000_106218037_106218089	1	
MC-CAR	14	106072000	106076000	14	106218592	106218770	3	1	0	16.3333333	144681	FALSE	FALSE	106072000_106076000_106218592_106218770	1	
MC-CAR	14	106082500	106086500	14	106101954	106102277	3	1	20	40	17615.5	TRUE	FALSE	106082500_106086500_106101954_106102277	1	
MC-CAR	14	106082500	106086500	14	106103662	106103891	15	0	10.8666667	11.8666667	19276.5	FALSE	TRUE	106082500_106086500_106103662_106103891	1	
MC-CAR	14	106082500	106086500	14	106127900	106128109	3	0	0.33333333	18.3333333	43504.5	FALSE	FALSE	106082500_106086500_106127900_106128109	1	
MC-CAR	14	106082500	106086500	14	106129048	106129135	2	0	1	0	44591.5	FALSE	FALSE	106082500_106086500_106129048_106129135	1	
MC-CAR	14	106082500	106086500	14	106198101	106198399	6	1	12	7.5	113750	FALSE	FALSE	106082500_106086500_106198101_106198399	1	
MC-CAR	14	106082500	106086500	14	106200441	106200566	6	0	11.8333333	0	116003.5	FALSE	FALSE	106082500_106086500_106200441_106200566	1	
MC-CAR	14	106082500	106086500	14	106200795	106201370	8	0	0.125	14.5	116582.5	FALSE	FALSE	106082500_106086500_106200795_106201370	1	
MC-CAR	14	106082500	106086500	14	106202076	106202384	4	0	45	0	117730	FALSE	FALSE	106082500_106086500_106202076_106202384	1	
MC-CAR	14	106082500	106086500	14	106227198	106227320	44	5	49.3409091	56.75	142759	TRUE	FALSE	106082500_106086500_106227198_106227320	1	
MC-CAR	14	106082500	106086500	14	106228380	106228719	6	0	40.5	11.6666667	144049.5	TRUE	FALSE	106082500_106086500_106228380_106228719	1	
MC-CAR	14	106082500	106086500	14	106229453	106229711	2	0	0	22	145082	FALSE	FALSE	106082500_106086500_106229453_106229711	1	
MC-CAR	14	106082500	106086500	14	106230274	106230511	2	0	0	0	145892.5	FALSE	FALSE	106082500_106086500_106230274_106230511	1	
MC-CAR	14	106098500	106102500	14	106125476	106125835	4	1	22.5	8.5	25155.5	FALSE	FALSE	106098500_106102500_106125476_106125835	1	
MC-CAR	14	106098500	106102500	14	106197139	106197659	10	0	2.6	17.4	96899	FALSE	TRUE	106098500_106102500_106197139_106197659	1	
MC-CAR	14	106098500	106102500	14	106226537	106226736	6	0	2.33333333	4.83333333	126136.5	FALSE	FALSE	106098500_106102500_106226537_106226736	1	
MC-CAR	14	106087954	106088307	14	106104500	106108500	3	0	3.33333333	2.33333333	18369.5	FALSE	FALSE	106087954_106088307_106104500_106108500	1	
MC-CAR	14	106089604	106089809	14	106104500	106108500	4	0	8	18.5	16793.5	FALSE	FALSE	106089604_106089809_106104500_106108500	1	
MC-CAR	14	106134500	106138500	14	106138720	106138811	8	0	6.75	7.71428571	2265.5	FALSE	FALSE	106134500_106138500_106138720_106138811	1	
MC-CAR	14	106134500	106138500	14	106237904	106237988	2	0	15	16	101446	FALSE	FALSE	106134500_106138500_106237904_106237988	1	
MC-CAR	14	106063703	106064078	14	106185500	106189500	6	0	0	4.66666667	123609.5	FALSE	FALSE	106063703_106064078_106185500_106189500	1	
MC-CAR	14	106065769	106066769	14	106185500	106189500	8	0	7.375	6.375	121231	FALSE	FALSE	106065769_106066769_106185500_106189500	1	
MC-CAR	14	106070223	106070386	14	106185500	106189500	3	0	0	0	117195.5	FALSE	FALSE	106070223_106070386_106185500_106189500	1	
MC-CAR	14	106185210	106185298	14	106185500	106189500	2	0	48.9375	36.9375	2246	TRUE	FALSE	106185210_106185298_106185500_106189500	1	
MC-CAR	14	38408547	38408621	14	106192000	106196000	2	0	60	11	67785416	TRUE	FALSE	38408547_38408621_106192000_106196000	1	
MC-CAR	14	106077009	106077260	14	106192000	106196000	2	0	5	0	116865.5	FALSE	FALSE	106077009_106077260_106192000_106196000	1	
MC-CAR	14	106078180	106078611	14	106192000	106196000	3	1	36	0	115604.5	FALSE	FALSE	106078180_106078611_106192000_106196000	1	
MC-CAR	14	106080339	106080413	14	106192000	106196000	2	0	0	35	113624	FALSE	FALSE	106080339_106080413_106192000_106196000	1	
MC-CAR	14	106191667	106191798	14	106192000	106196000	7	1	24.862069	21.7241379	2267.5	FALSE	FALSE	106191667_106191798_106192000_106196000	1	
MC-CAR	14	106196000	106200000	14	106200205	106200346	14	1	60	60	2275.5	TRUE	FALSE	106196000_106200000_106200205_106200346	1	
KMS-12-BM	14	106111000	106115000	14	106238500	106242500	15	0	20.3333333	43.1333333	127500	TRUE	TRUE	106111000_106115000_106238500_106242500	1	TRUE
MC-CAR	14	106208000	106212000	14	106239121	106239203	3	0	21	3.66666667	29162	FALSE	FALSE	106208000_106212000_106239121_106239203	1	
MC-CAR	14	106208000	106212000	14	106348167	106348294	8	0	14.375	3.75	138230.5	FALSE	FALSE	106208000_106212000_106348167_106348294	1	
MC-CAR	14	106238500	106242500	14	106242710	106242823	8	1	23.25	23.25	2266.5	FALSE	FALSE	106238500_106242500_106242710_106242823	1	
MC-CAR	14	106113674	106113854	14	106324500	106328500	6	0	0	60	212736	FALSE	FALSE	106113674_106113854_106324500_106328500	1	
MC-CAR	14	106213171	106213245	14	106324500	106328500	3	0	0	60	113292	FALSE	FALSE	106213171_106213245_106324500_106328500	1	
MC-CAR	14	106529500	106533500	14	107008913	107008988	2	0	49	0	477450.5	FALSE	FALSE	106529500_106533500_107008913_107008988	2	
MC-CAR	14	106518106	106518220	14	106570000	106574000	4	0	53.5	0	53837	FALSE	FALSE	106518106_106518220_106570000_106574000	1	
MC-CAR	14	106711689	106711785	14	106712000	106716000	3	22	60	58.8571429	2263	TRUE	FALSE	106711689_106711785_106712000_106716000	1	
MC-CAR	14	106777710	106777798	14	106778000	106782000	4	9	60	60	2246	TRUE	FALSE	106777710_106777798_106778000_106782000	1	
MC-CAR	14	69644244	69644318	14	106782000	106786000	2	0	60	9	37139719	TRUE	FALSE	69644244_69644318_106782000_106786000	1	
MC-CAR	14	106781679	106781791	14	106782000	106786000	2	7	47.25	58.5	2265	TRUE	FALSE	106781679_106781791_106782000_106786000	1	
MC-CAR	14	106789500	106793500	14	106793738	106793835	3	0	27	27	2286.5	FALSE	FALSE	106789500_106793500_106793738_106793835	1	
MC-CAR	14	106789500	106793500	14	106814368	106814430	2	1	23.5	36	22899	TRUE	FALSE	106789500_106793500_106814368_106814430	1	
MC-CAR	14	106789500	106793500	14	106814720	106814974	3	5	22	9	23347	FALSE	FALSE	106789500_106793500_106814720_106814974	1	
MC-CAR	14	106789500	106793500	14	106815259	106815468	3	1	0	0	23863.5	FALSE	FALSE	106789500_106793500_106815259_106815468	1	
MC-CAR	14	106789500	106793500	14	106815965	106816086	3	4	17	0	24525.5	FALSE	FALSE	106789500_106793500_106815965_106816086	1	
MC-CAR	14	106789500	106793500	14	106816718	106816926	3	1	0	21.3333333	25322	FALSE	FALSE	106789500_106793500_106816718_106816926	1	

MC-CAR	14	106789500	106793500	14	106818005	106818080	2	0	0	0	26542.5	FALSE	FALSE	106789500_106793500_106818005_106818080	1	
MC-CAR	14	106789500	106793500	14	107048552	107048772	2	0	0	11.5	257162	FALSE	FALSE	106789500_106793500_107048552_107048772	1	
MC-CAR	14	106789500	106793500	14	107083254	107083380	10	0	0.3	0	291817	FALSE	TRUE	106789500_106793500_107083254_107083380	1	
MC-CAR	14	106789500	106793500	14	107095138	107095232	5	0	0	0	303685	FALSE	FALSE	106789500_106793500_107095138_107095232	1	
MC-CAR	14	106796193	106796287	14	106796500	106800500	2	7	42.5	36	2260	TRUE	FALSE	106796193_106796287_106796500_106800500	1	
MC-CAR	14	106712815	106712943	14	107011000	107015000	4	18	50.5	28.5	300121	TRUE	FALSE	106712815_106712943_107011000_107015000	1	
MC-CAR	14	107045000	107049000	14	107083254	107083414	12	0	5	0	36334	FALSE	TRUE	107045000_107049000_107083254_107083414	1	
MC-CAR	14	107045000	107049000	14	107095124	107095282	16	0	19.9375	0	48203	FALSE	TRUE	107045000_107049000_107095124_107095282	1	
MC-CAR	14	107084605	107084782	14	107085000	107089000	3	3	44.25	54.1875	2306.5	TRUE	FALSE	107084605_107084782_107085000_107089000	1	
MC-CAR	14	107147203	107147285	14	107147500	107151500	2	3	60	60	2256	TRUE	FALSE	107147203_107147285_107147500_107151500	1	
MC-CAR	14	106811205	106811378	14	107271500	107275500	3	0	0	3.33333333	462208.5	FALSE	FALSE	106811205_106811378_107271500_107275500	1	
KMS-12-BM	14	106316000	106320000	14	106325000	106329000	40	0	54.4	58.575	9000	TRUE	TRUE	106316000_106320000_106325000_106329000	1	TRUE
MM1S	14	106327500	106331500	14	106382689	106382763	4	0	60	12	53226	TRUE	FALSE	106327500_106331500_106382689_106382763	1	
KMS-12-BM	14	106328000	106332000	14	106345500	106349500	16	0	43.5	42.8125	17500	TRUE	TRUE	106328000_106332000_106345500_106349500	1	TRUE
MM1S	14	106327500	106331500	14	106815720	106815857	3	0	60	0	486288.5	FALSE	FALSE	106327500_106331500_106815720_106815857	1	
MM1S	14	106327500	106331500	14	107131031	107131154	3	0	60	0	801592.5	FALSE	FALSE	106327500_106331500_107131031_107131154	1	
NCI-H929	14	106072000	106076000	14	106076000	106080000	224	29	23.0848214	23.46875	4000	FALSE	FALSE	106072000_106076000_106076000_106080000	17	
NCI-H929	14	106082500	106086500	14	106103500	106107500	24	0	9.375	8.54166667	21000	FALSE	TRUE	106082500_106086500_106103500_106107500	6	
KMS-12-BM	14	106355500	106359500	14	107168000	107172000	25	0	32.12	25.48	812500	TRUE	TRUE	106355500_106359500_107168000_107172000	1	TRUE
NCI-H929	14	106859500	106863500	14	106861500	106865500	68	80	35.0441176	41.1029412	2000	TRUE	FALSE	106859500_106863500_106861500_106865500	7	
NCI-H929	14	106859500	106863500	14	106862500	106866500	4	3	32.5	32.5	3000	TRUE	FALSE	106859500_106863500_106862500_106866500	7	
NCI-H929	14	107262000	107266000	14	107265500	107269500	8	14	54	53.25	3500	TRUE	FALSE	107262000_107266000_107265500_107269500	15	
NCI-H929	14	107268500	107272500	14	107271500	107275500	216	372	47.1296296	51.9166667	3000	TRUE	FALSE	107268500_107272500_107271500_107275500	15	
NCI-H929	14	106072000	106076000	14	106118857	106119326	4	0	10.25	0	45091.5	FALSE	FALSE	106072000_106076000_106118857_106119326	1	
NCI-H929	14	106072000	106076000	14	106119571	106119768	2	0	6.5	13	45669.5	FALSE	FALSE	106072000_106076000_106119571_106119768	1	
NCI-H929	14	106072000	106076000	14	106217204	106217278	2	0	30	0	143241	FALSE	FALSE	106072000_106076000_106217204_106217278	2	
NCI-H929	14	106072000	106076000	14	106218011	106218208	5	0	9	0	144109.5	FALSE	FALSE	106072000_106076000_106218011_106218208	1	
NCI-H929	14	106082500	106086500	14	106086707	106086852	23	0	22.7826087	28.0454545	2279.5	FALSE	TRUE	106082500_106086500_106086707_106086852	1	
NCI-H929	14	106082500	106086500	14	106102228	106102577	20	1	25.6	4.55	17902.5	FALSE	FALSE	106082500_106086500_106102228_106102577	1	
NCI-H929	14	106082500	106086500	14	106128940	106129249	3	0	50.6666667	3	44594.5	TRUE	FALSE	106082500_106086500_106128940_106129249	1	
NCI-H929	14	106082500	106086500	14	106198106	106198184	3	1	56.3333333	7.66666667	113645	TRUE	FALSE	106082500_106086500_106198106_106198184	1	
NCI-H929	14	106082500	106086500	14	106200437	106200972	16	0	10.4375	0.5625	116204.5	FALSE	TRUE	106082500_106086500_106200437_106200972	1	
NCI-H929	14	106082500	106086500	14	106201246	106201494	5	0	0	0	116870	FALSE	FALSE	106082500_106086500_106201246_106201494	1	
NCI-H929	14	106082500	106086500	14	106202014	106202240	3	0	3	0	117627	FALSE	FALSE	106082500_106086500_106202014_106202240	1	
NCI-H929	14	106082500	106086500	14	106227198	106227310	58	5	45.9310345	55.7758621	142754	TRUE	FALSE	106082500_106086500_106227198_106227310	1	
KMS-12-BM	14	106078500	106082500	14	106197130	106197315	16	0	0.5625	32.875	116722.5	TRUE	TRUE	106078500_106082500_106197130_106197315	1	TRUE
NCI-H929	14	106082500	106086500	14	106229302	106229503	3	0	0	0	144902.5	FALSE	FALSE	106082500_106086500_106229302_106229503	1	
NCI-H929	14	106094634	106094792	14	106095000	106099000	7	0	54.3888889	52.5555556	2287	TRUE	FALSE	106094634_106094792_106095000_106099000	1	
NCI-H929	14	106098500	106102500	14	106197250	106197353	3	0	6	9	96801.5	FALSE	FALSE	106098500_106102500_106197250_106197353	1	
NCI-H929	14	106098500	106102500	14	106197554	106197628	2	0	0	6	97091	FALSE	FALSE	106098500_106102500_106197554_106197628	1	
NCI-H929	14	106098500	106102500	14	106225680	106226014	3	0	3.33333333	2.33333333	125347	FALSE	FALSE	106098500_106102500_106225680_106226014	1	
NCI-H929	14	106098500	106102500	14	106226582	106226895	5	1	0.2	11	126238.5	FALSE	FALSE	106098500_106102500_106226582_106226895	1	
NCI-H929	14	106086562	106086914	14	106103500	106107500	3	1	2.66666667	7	18762	FALSE	FALSE	106086562_106086914_106103500_106107500	1	
NCI-H929	14	106087401	106087680	14	106103500	106107500	2	0	1.33333333	0.5	17959.5	FALSE	FALSE	106087401_106087680_106103500_106107500	1	
NCI-H929	14	106088160	106088242	14	106103500	106107500	2	0	0	0	17299	FALSE	FALSE	106088160_106088242_106103500_106107500	1	
NCI-H929	14	106134500	106138500	14	106138719	106138793	6	0	0	0	2256	FALSE	FALSE	106134500_106138500_106138719_106138793	1	
NCI-H929	14	106143000	106147000	14	106147203	106147376	9	0	15	15	2289.5	FALSE	FALSE	106143000_106147000_106147203_106147376	1	
NCI-H929	14	106045961	106046424	14	106163000	106167000	6	0	2.83333333	11.6666667	118807.5	FALSE	FALSE	106045961_106046424_106163000_106167000	1	
NCI-H929	14	106162692	106162766	14	106163000	106167000	2	0	0.5	0.5	2271	FALSE	FALSE	106162692_106162766_106163000_106167000	1	
NCI-H929	14	106070536	106070738	14	106189000	106193000	14	1	7.64285714	0	120363	FALSE	FALSE	106070536_106070738_106189000_106193000	1	
NCI-H929	14	106329000	106333000	14	106392755	106392841	2	0	60	60	61798	TRUE	FALSE	106329000_106333000_106392755_106392841	1	
NCI-H929	14	106030415	106030722	14	106580000	106584000	4	0	9	5	551431.5	FALSE	FALSE	106030415_106030722_106580000_106584000	1	
KMS-12-BM	14	106213076	106213403	14	106325000	106329000	40	0	22.8918919	60	113760.5	TRUE	TRUE	106213076_106213403_106325000_106329000	1	TRUE
NCI-H929	14	106579608	106579682	14	106580000	106584000	2	0	18	18	2355	FALSE	FALSE	106579608_106579682_106580000_106584000	1	
NCI-H929	14	106711658	106711779	14	106712000	106716000	2	20	60	60	2281.5	TRUE	FALSE	106711658_106711779_106712000_106716000	1	
NCI-H929	14	106714156	106714296	14	106714500	106718500	6	22	48.1538462	41.6923077	2274	TRUE	FALSE	106714156_106714296_106714500_106718500	1	
NCI-H929	14	106725500	106729500	14	106729718	106729871	13	4	60	60	2294.5	TRUE	FALSE	106725500_106729500_106729718_106729871	1	
NCI-H929	14	106475954	106476175	14	106778000	106782000	2	1	4.33333333	49.3333333	303935.5	TRUE	FALSE	106475954_106476175_106778000_106782000	1	
NCI-H929	14	106788500	106792500	14	106792735	106792826	11	1	21.8181818	21.8181818	2280.5	FALSE	FALSE	106788500_106792500_106792735_106792826	1	
NCI-H929	14	106788500	106792500	14	106813523	106813618	9	4	14.1111111	0	23070.5	FALSE	FALSE	106788500_106792500_106813523_106813618	1	
NCI-H929	14	106788500	106792500	14	106815718	106816201	4	5	10.25	0	25459.5	FALSE	FALSE	106788500_106792500_106815718_106816201	1	
NCI-H929	14	106788500	106792500	14	106816750	106816933	2	0	13.5	15	26341.5	FALSE	FALSE	106788500_106792500_106816750_106816933	1	
NCI-H929	14	106788500	106792500	14	106817263	106817374	2	0	0	0	26818.5	FALSE	FALSE	106788500_106792500_106817263_106817374	1	
NCI-H929	14	106796137	106796284	14	106796500	106800500	5	5	41.5454545	37.8181818	2289.5	TRUE	FALSE	106796137_106796284_106796500_106800500	1	
NCI-H929	14	106835500	106839500	14	106839720	106839835	9	7	60	60	2277.5	TRUE	FALSE	106835500_106839500_106839720_106839835	1	
NCI-H929	14	107093202	107093535	14	107271500	107275500	9	0	54	39.7777778	180131.5	TRUE	FALSE	107093202_107093535_107271500_107275500	1	
L-363	14	106203000	106207000	14	106340000	106344000	29	0	60	60	137000	TRUE	TRUE	106203000_106207000_106340000_106344000	1	TRUE
LP-1	14	106211119	106211906	14	106325000	106329000	51	0	5.36734694	60	115487.5	TRUE	TRUE	106211119_106211906_106325000_106329000	1	TRUE
LP-1	14	106328000	106332000	14	106791007	106791179	10	0	60	3.9	461093	TRUE	TRUE	106328000_106332000_106791007_106791179	1	TRUE
MC-CAR	14	106208000	106212000	14	106356000	106360000	29	0	19.4137931	42.1724138	148000	TRUE	TRUE	106208000_106212000_106356000_106360000	1	TRUE
MC-CAR	14	106238500	106242500	14	106324500	106328500	38	0	13.0263158	58.5526316	86000	TRUE	TRUE	106238500_106242500_106324500_106328500	1	TRUE
MC-CAR	14	106241000	106245000	14	106324500	106328500	27	0	10.8518519	60	83500	TRUE	TRUE	106241000_106245000_106324500_106328500	1	TRUE
MC-CAR	14	106328500	106332500	14	107011000	107015000	22	0	36.5909091	30.7272727	682500	TRUE	TRUE	106328500_106332500_107011000_107015000	1	TRUE
MC-CAR	14	106208000	106212000	14	106238314	106238503	10	0	22.7	35.8181818	28408.5	TRUE	TRUE	106208000_106212000_106238314_106238503	1	TRUE
MM1S	14	106210000	106214000	14	106322000	106326000	48	0	11.9375	40.0625	112000	TRUE	TRUE	106210000_106214000_106322000_106326000	1	TRUE
MM1S	14	106327500	106331500	14	106791003	106791192	10	0	60	11.2	461597.5	TRUE	TRUE	106327500_106331500_106791003_106791192	1	TRUE
NCI-H929	14	106329000	106333000	14	106383500	106387500	39	0	54.6153846	58.6153846	54500	TRUE	TRUE	106329000_106333000_106383500_106387500	1	TRUE
NCI-H929	14	106082500	106086500	14	106228380	106228726	12	0	56	15.5	144053	TRUE	TRUE	106082500_106086500_106228380_106228726	1	TRUE
NCI-H929	14	106150017	106150274	14	106580000	106584000	24	0	33.5151515	20.0606061	431854.5	TRUE	TRUE	106150017_106150274_106580000_106584000	1	TRUE
OPM-2	14	106053000	106057000	14	106175500	106179500	46	0	48.8043478	47.1304348	122500	TRUE	TRUE	106053000_106057000_106175500_106179500	1	TRUE
OPM-2	14	106111000	106115000	14	106175500	106179500	27	0	27.6296296	58.6296296	64500	TRUE	TRUE	106111000_106115000_106175500_106179500	1	TRUE
OPM-2	14	106176445	106176686	14	106325000	106329000	39	0	52.6666667	60	150434.5	TRUE	TRUE	106176445_106176686_106325000_106329000	1	TRUE
OPM-2	14	106328500	106332500	14	106378500	106382500	16	0	27.6875	20.5	50000	TRUE	TRUE	106328500_106332500_106378500_106382500	1	TRUE
OPM-2	14	106797000	106801000	14	106821500	106825500	6	44	10.5	13.8333333	24500	FALSE	FALSE	106797000_106801000_106821500_106825500	1	
OPM-2	14	106821200	106821279	14	106821500	106825500	2	3	22.8	16	2260.5	FALSE	FALSE	106821200_106821279_106821500_106825500	1	
SK-MM-2	14	106329000	106333000	14	106352500	106356500	14	0	53.5714286	51.7142857	23500	TRUE	TRUE	106329000_106333000_106352500_106356500	1	TRUE
SK-MM-2	14	106356000	106360000	14	106450500	106454500	15	0	47.5333333	32.7333333	94500	TRUE	TRUE	106356000_106360000_106450500_106454500	1	TRUE
SK-MM-2	14	106859500	106863500	14	106862500	106866500	1	3	0	0	3000	FALSE	FALSE	106859500_106863500_106862500_106866500	7	
SK-MM-2	14	106881000	106885000	14	106884000	106888000	3	20	15.3333333	13.3333333	3000	FALSE	FALSE	106881000_106885000_106884000_106888000	5	
U-266	14	106067500	106071500	14	106092000	106096000	42	0	56	46.547619	24500	TRUE	TRUE	106067500_106071500_106092000_106096000	1	TRUE
U-266	14	106067500	106071500	14	106324000	106328000	20	0	55	57.2	256500	TRUE	TRUE	106067500_106071500_106324000_106328000	1	TRUE
U-266	14	106067500	106071500	14	106177025	106177106	12	0	60	41.5833333	107565.5	TRUE	TRUE	106067500_106071500_106177025_106177106	1	TRUE
U-266	14	107093220	107093541	14	107271500	107275500	13	0	56.5384615	36.3846154	180119.5	TRUE	TRUE	107093220_107093541_107271500_107275500	1	TRUE
